# Glutantβase: a database for improving the rational design of glucose-tolerant β-glucosidases

**DOI:** 10.1186/s12860-020-00293-y

**Published:** 2020-07-01

**Authors:** Diego Mariano, Naiara Pantuza, Lucianna H. Santos, Rafael E. O. Rocha, Leonardo H. F. de Lima, Lucas Bleicher, Raquel Cardoso de Melo-Minardi

**Affiliations:** 1 Laboratory of Bioinformatics and Systems. Department of Computer Science, Universidade Federal de Minas Gerais, Belo Horizonte, 31270-901 Brazil; 2 Laboratory of Molecular Modelling and Bioinformatics (LAMMB), Department of Physical and Biological Sciences, Universidade Federal de São João Del-Rei, Campus Sete Lagoas, Sete Lagoas, 35701-970 Brazil; 3 Protein Computational Biology Laboratory, Department of Biochemistry and Immunology, Universidade Federal de Minas Gerais, Belo Horizonte, 31270-901 Brazil

**Keywords:** β-Glucosidases, Biofuel, Rational design of enzymes, Glucose-tolerant, Thermostability, GH1

## Abstract

Β-glucosidases are key enzymes used in second-generation biofuel production. They act in the last step of the lignocellulose saccharification, converting cellobiose in glucose. However, most of the β-glucosidases are inhibited by high glucose concentrations, which turns it a limiting step for industrial production. Thus, β-glucosidases have been targeted by several studies aiming to understand the mechanism of glucose tolerance, pH and thermal resistance for constructing more efficient enzymes. In this paper, we present a database of β-glucosidase structures, called Glutantβase. Our database includes 3842 GH1 β-glucosidase sequences collected from UniProt. We modeled the sequences by comparison and predicted important features in the 3D-structure of each enzyme. Glutantβase provides information about catalytic and conserved amino acids, residues of the coevolution network, protein secondary structure, and residues located in the channel that guides to the active site. We also analyzed the impact of beneficial mutations reported in the literature, predicted in analogous positions, for similar enzymes. We suggested these mutations based on six previously described mutants that showed high catalytic activity, glucose tolerance, or thermostability (A404V, E96K, H184F, H228T, L441F, and V174C). Then, we used molecular docking to verify the impact of the suggested mutations in the affinity of protein and ligands (substrate and product). Our results suggest that only mutations based on the H228T mutant can reduce the affinity for glucose (product) and increase affinity for cellobiose (substrate), which indicates an increment in the resistance to product inhibition and agrees with computational and experimental results previously reported in the literature. More resistant β-glucosidases are essential to saccharification in industrial applications. However, thermostable and glucose-tolerant β-glucosidases are rare, and their glucose tolerance mechanisms appear to be related to multiple and complex factors. We gather here, a set of information, and made predictions aiming to provide a tool for supporting the rational design of more efficient β-glucosidases. We hope that Glutantβase can help improve second-generation biofuel production. Glutantβase is available at http://bioinfo.dcc.ufmg.br/glutantbase.

## Background

Biofuels are a clean and renewable source of energy, rising as an alternative to fossil fuels, such as those derived from petroleum [1, 2]. They are produced from agricultural materials, for example, sugarcane, corn, soil, seaweed, and so on [3]. Second-generation biofuel production occurs in several steps, such as pre-processing, saccharification, and fermentation. The saccharification step occurs by the synergistic action of three types of enzymes: endoglucanases (E.C. 3.2.1.4), exoglucanases, also called cellobiohydrolases (E.C. 3.2.1.91), and β-glucosidases (E.C. 3.2.1.21) [4, 5]. Endoglucanases act in the cellulose structure, releasing oligosaccharides of different lengths. Cellobiohydrolases hydrolyzes the terminal of these oligosaccharides, releasing mainly cellobiose molecules. Then, β-glucosidases hydrolyzes the cellobiose glycosidic bond, releasing two glucose molecules [4–7]. However, most β-glucosidases are strongly inhibited by high glucose concentrations [8–10]. Thus, these enzymes have been considered by several studies as targets to improve high glucose concentrations tolerance by site-direct mutagenesis or the design of new enzymes [8–42]. Also, many reviews have reported the importance of glucose tolerance for improving the saccharification process [4, 7, 43].

Recently, Salgado et al. [43] proposed a β-glucosidase classification system divided into four groups: (i) β-glucosidases strongly inhibited by glucose (most of them); (ii) β-glucosidases tolerant to glucose; (iii) β-glucosidases stimulated by low glucose concentrations but inhibited in high concentrations; and (iv) β-glucosidases not inhibited by high glucose concentrations. To the best of our knowledge, the groups ii, iii, and iv are composed of few enzymes. Therefore, many studies aimed to transfer their characteristics to other non-efficient enzymes for biomass hydrolysis. For example, Yang et al. [9] evaluated the importance of a set of amino acid positions through site-direct mutagenesis. They reported that H228T and N301Q/V302F mutations could lead a marine non-resistant β-glucosidase to glucose tolerance. Also, Giuseppe et al. [10] reported that shape and the presence of hydrophobic residues in the middle of the substrate channel could be related to the structural basis of glucose tolerance. Furthermore, mutations in the positions 174, 404, and 441 of a β-glucosidase extracted from the Turpan Depression metagenome, have been reported as necessary for increasing the optimal temperature and reduce the optimal pH [12]. The study of Cao et al. [12] demonstrated that the β-glucosidase of the Turpan Depression metagenome could be classified as glucose-tolerant. However, the wild enzyme presented a low K_cat_/K_m_ value when using cellobiose as substrate. Also, the half-life of the wild enzyme at 50 °C was only 1 h. Therefore, this could hinder the employment of this enzyme in cellulose hydrolysis. The combination of three beneficial mutations (W174C/A404V/L441F) was essential to extending the half-life to 48 h, keeping the IC_50_ and, consequently, the glucose tolerance. The use of the mutant enzyme allowed an improvement of the sugarcane bagasse conversion by 14–35%, which demonstrated that multiple aspects should be considered to propose mutations that improve the activity of β-glucosidases.

Computational approaches have also been used in the search for crucial amino acids to convert non-tolerant to tolerant β-glucosidases. For instance, a set of 15 mutations have been proposed to improve the activity of a non-tolerant β-glucosidase from a marine metagenome [44]. From these 15 proposed mutations, a previous study has provided experimental evidence of enhancing β-glucosidase activity even in high glucose concentrations for three of them: H228C, H228T, and H228V [9]. The residues mutated V302F, N301Q/V302F, F172I, V227M, G246S, T299S, and H228T were also the target of other computational studies that used classic and accelerated molecular dynamics simulation to highlight their role in glucose releasing [45, 46]. Despite all these efforts, the rational design of more efficient β-glucosidases is still a challenge.

Previously, a database containing structures of glucose-tolerant β-glucosidases, called Betagdb, has been proposed [4]. Betagdb database was developed based on papers that reported glucose-tolerant β-glucosidases with experimental validations and structural data from public databases (only 23 occurrences were found at that moment). With the rising and popularization of next-generation sequencing platforms, thousands of β-glucosidase from several organisms were stored in sequence databases, such as UniProt. These data could be better explored to bring new insights into β-glucosidase mechanisms. In this paper, we propose a database of β-glucosidases enzymes called Glutantβase. Our database includes 3842 sequences collected from UniProt of β-glucosidases from the GH1 family (Glycoside Hydrolase Family 1), the most promising family for second-generation biofuel production. For all sequences, we performed comparative modeling, predicted their secondary structure, detected the residues involved in coevolution networks, detailed the conserved residues, the catalytic glutamates, and the residues present in the substrate channel that guides to the active site. Also, we hypothesized that mutations described in the literature as beneficial for improving β-glucosidase activity could be extrapolated to other β-glucosidases. To verify this, we modeled 5607 mutant proteins based on analogous positions of six beneficial mutations described in the literature: H228T [9], V174C [12], A404V [12], L441F [12], H184F [27], and E96K [47]. We performed molecular docking of glucose and cellobiose in the wild and mutant proteins to verify the affinity score variation. Our results show that only mutations in analogous positions of H228T impact in the interactions of glucose and cellobiose, which agree with previous computational and experimental studies [9, 44, 45]. We hope Glutantβase might help engineering tolerant β-glucosidase enzymes to bring improvements in second-generation biofuel production.

## Construction and content

### Sequence collection

Β-glucosidases sequences were collected from UniProt (http://www.uniprot.org/). We collected sequences classified with the E. C. number 3.2.1.21 and from the GH1 family. Then, the sequences were submitted to comparative modeling (see next section) to create their three-dimensional structures. However, the sequences with less than 25% identity to a three-dimensional structure template were removed. Three thousand eight hundred forty-two sequences were included in Glutantβase. For each sequence, we also collected: (i) protein name; (ii) organism of origin; (iii) sequence length; and (iv) UniProt ID.

### Comparative modeling

We performed comparative modeling for each GH1 β-glucosidase sequence collected from UniProt. Comparative modeling has been used to obtain three-dimensional structures from sequences in many β-glucosidases studies [9, 11, 13, 15, 16, 26, 48]. We used an adapted version of Bitar & Franco’s protocol [49] to perform comparative modeling. Three-dimensional structures for templates were collected from the Protein Data Bank (PDB) [50]. To automatize the process, we constructed a pipeline using in-house Python scripts and Biopython [51]. Our pipeline was divided into four steps:

(i) **Template’s definition**: we defined a 3D-structure template for each sequence. For this, we performed sequence alignment against sequences obtained from three-dimensional structures collected from PDB using the blastp tool [52]. The 3D-structure with the highest identity was defined as the template (only if the identity was higher than 25%);
(ii) **Pairwise sequence alignment**: target and template sequences were aligned using the Clustal W v2.1 tool [53]. The sequence alignments (and template’s 3D-structure) were used as input for model’s construction step;
(iii) **Models’ construction**: we performed comparative modeling using the MODELLER tool [54]. We constructed 100 models for each protein using default parameters;
(iv) **Assessment and definition of the best model**: we defined the best 3D-model for each protein using the DOPE score [55]. We also constructed Ramachandran plots for each model selected using the standard PSI and PHI preferences [56] implemented in PyRAMA script (https://github.com/gerdos/PyRAMA). List of templates used and DOPE scores for best models are available at additional files (Tables S1-S2).

### Multiple sequence alignment

We performed multiple sequence alignment of GH1 β-glucosidases using the Clustal Omega tool (default parameters) [57]. We used Clustal Omega since it can deal with a vast number of sequences [58]. We collected the most representative amino acid for each position of the multiple sequence alignment. Then, we constructed a corresponding position table, the so-called “global position table”, for each amino acid of all sequences. From this table, we determined amino acids conserved in over 50 and 80% of the 3842 Glutantβase’s sequences. We also used the global position table to detect, for each sequence, the two glutamates described as catalytic amino acids in the literature [26, 47, 59, 60].

### Substrate channel residues

To define which amino acid residues from the substrate channel, i.e.*,* residues that possibly interact with ligands in their way to the active site, we used the Betagdb definition of catalytic pocket [4]. Betagdb uses the β-glucosidase of the termite *Neotermes koshunensis* in complex to cellobiose (PDB ID: 3VIK) to detect the residues at 6.5 Å from any atom of the ligand (in this case, the substrate cellobiose). This value was defined based on the method proposed by [61] to construct representative fingerprints of protein pockets. For *N. koshunensis* β-glucosidase, 24 residues in the substrate channel were detected. Once again, we used the global position table to identify analogous positions for each Glutantβase sequence.

### Secondary structure prediction

We used the DSSP command line tool [62, 63] to predict the secondary structure of each sequence. DSSP calculates the most likely secondary structure assignment from each three-dimensional structure model. DSSP returns an H character for amino acid residues in α-helix, B for residue in isolated β-bridge, E for an extended strand participant of a β ladder, G for 3-helix, I for π-helix, T for a hydrogen-bonded turn, and S for a bend.

### Coevolution analysis

We performed the family-wide sequence coevolution analysis using the Decomposition of Residue Coevolution Networks (DRCN) method with PFstats software [64, 65]. A multiple sequence alignment from glycosyl hydrolases (family 1) was obtained from Pfam [66], entry PF00232. The alignment was filtered to remove fragments (a minimum 80% size of the hidden Markov model used for this protein family) and redundancy (80% maximum identity), resulting in a final alignment of 4084 sequences. Residue-specific correlations were calculated as described previously [64], with a minimum score of 10, minimum sub-alignment size of 15%, and Δf = 0.2. The resulting coevolution network obtained from these pairwise correlation signals was decomposed into communities using a standard connected components algorithm.

### Extrapolated mutations

In the last years, several studies have proposed mutations through site-direct mutagenesis to improve the activity of β-glucosidases enzymes [4, 9, 10, 12, 27, 43, 44, 67, 68]. Hence, many mutation sites have been described as responsible for leading to beneficial characteristics, such as glucose tolerance and thermostability. We wondered if the same effects could be extrapolated to other β-glucosidases by mutating analogous sites. To verify this hypothesis, we chose six mutations described in the literature as beneficial to improve β-glucosidase activity, glucose resistance, or thermostability (Table 1).

**Table 1:** Mutations reported in the literature for improving the activity, glucose tolerance, and stability of β-glucosidases

#	Mutation	Effect	Source
**1**	H228T	Responsible for attracting glucose to the middle of the substrate channel and, then, to the exit, which improves the resistance to product inhibition.	[9]
**2**	V174C	Mutations were described to increase the optimal temperature from 50 ° C to 60°, reduce the optimal pH from 6 to 5.5, and increases the half-life from 1 to 2-20 h.	[12]
**3**	A404V		
**4**	L441F		
**5**	H184F	This mutation has been reported as responsible for promoting an increase in the inhibition constant for glucose.	[27]
**6**	E96K	Described as responsible for improving the protein thermostability.	[47]

We scanned the global position table for sequences with the same amino acid mutated in an analogous position. Then, we suggested a mutation for the amino acid residue based on the mutation described in the literature. For example, for the β-glucosidase of a marine metagenome (UniProt ID: D0VEC8), the mutation H228T has been experimentally described as responsible for improving the glucose tolerance and, thus, improving the β-glucosidase’s catalytic activity even in high concentrations of the product. Based on global position table, the position 228 of D0VEC8 is analogous to the position 235 in the β-glucosidase of *Microbacterium sp. Leaf320* (UniProt ID: A0A0Q5FWL5)*.* Also, both present a histidine in this position. Hence, we hypothesized that the mutant H235T of the β-glucosidase of *Microbacterium sp. Leaf320* should present similar characteristics that the mutant H228T of the marine metagenome β-glucosidase. To prove this, we extrapolated possible mutations from the six mutations described in Table 1 to all Glutantβase sequences using a similar strategy to the earlier described. A total of 5607 mutations were suggested for the 3842 Glutantβase sequences (an average of 1.45 mutations per sequence). Then, we performed comparative modeling for each mutant using the point-mutation script of the MODELLER software [54]. To estimate if the mutations impact the ligand interaction in the substrate channel, we performed molecular docking analysis.

### Molecular docking

Before molecular docking, we performed a minimization step for the 3842 model structures (from now on, called wild) and for the 5607 mutant modeled structures (from now on, called mutants) using AMBER16 [69]. This was performed to minimize the potential energy of the modeled structures. We used 750 steps of the steepest descent algorithm, and then we switched to the conjugate gradient algorithm for another 250 steps.

We performed docking for glucose and cellobiose for wild and mutant structures using Autodock Vina [70]. Based on in-house protocols, we generated ten binding modes and defined the exhaustiveness parameter as 20. The docking region was defined by a cubic box of 15x15x15 Å. The box center was calculated using in-house Python scripts based on the average of the atom coordinates of the two catalytic glutamates. Glucose and cellobiose structures were collected from the Zinc database [71]. To compare the docking results, we used the affinity score (Kcal/mol) calculated by Vina. A higher negative value indicates a better affinity for a determined ligand, while a positive value shows a lower affinity for the ligand.

We expected that a high affinity for glucose indicates an inhibition by it, which may show a lower catalytic activity. On the other hand, a high affinity for cellobiose (i.e.*,* the substrate) may indicate higher catalytic activity. As Autodock Vina uses the Monte Carlo algorithm, a nondeterministic algorithm, the same docking experiment performed two or more times could get different results. To reduce the random impact of the Monte Carlo algorithm, we performed each docking experiment in triplicate. We analyzed average affinity scores for (i) the first docked pose in triplicate, (ii) poses one to three in triplicate, and (iii) poses one to ten in triplicate.

To evaluate if affinity score changes were statistically significant, we carried out the student’s t-test for paired samples using in-house R scripts (5% of significance). For this hypothesis test, we verified the statistical relevance for each of the six possible mutations (Table 1).

### Web-based tool

We incorporate the results into a webtool available at http://bioinfo.dcc.ufmg.br/glutantbase. The web-based tool was constructed using the same framework structure of [44, 72–74], and the database was built using the MySQL Database Management System (https://mysql.com). For each β-glucosidase structure, a three-dimensional visualization was constructed using 3Dmol [75]. Furthermore, we used BLAST [52] to perform searches for similar sequences inside Glutantβase.

## Utility and discussion

### Glutantβase webtool

**Fig. 1:**
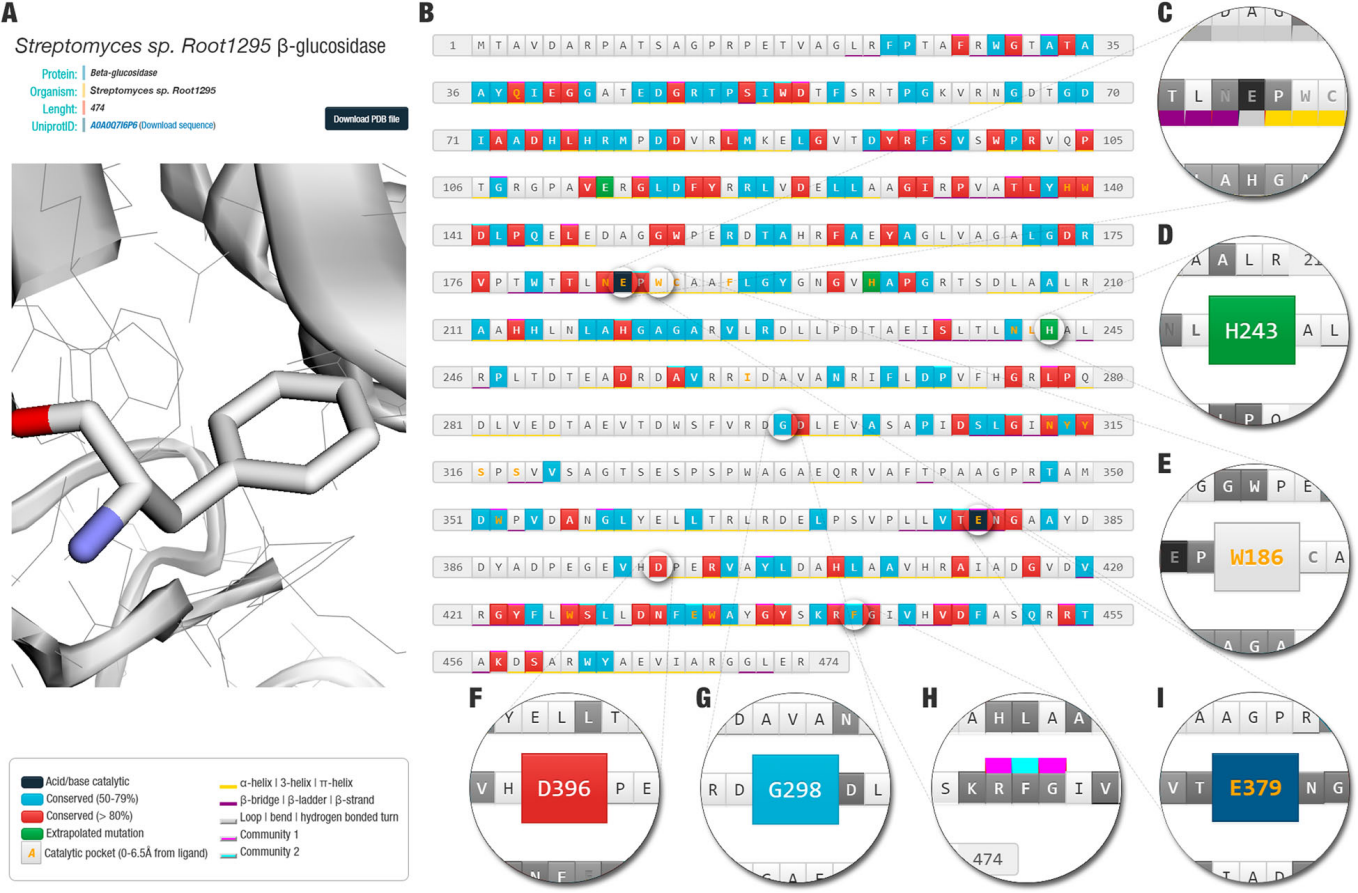
Glutantβase’s interface overview. (**a**) Protein’s details and three-dimensional visualization (for this example, we used the β-glucosidase of *Streptomyces sp. Root1295* - UniProt ID: A0A0Q7I6P6). (**b**) Protein sequence. Some amino acids are colored according to their predicted role: (**c**) secondary structure (border-bottom is colored of yellow for an alpha-helix region, purple for a β-strand region, and grey for a loop region); (**d**) predicted mutation (green); (**e**) residue located in the substrate channel (orange letters); (**f**) amino acids conserved in more than 80% of the GH1 β-glucosidases (red); (**g**) amino acids conserved in more than 50% of the GH1 β-glucosidases (blue); (**h**) amino acids present in the coevolution network (border-top is colored of magenta for community 1 and colored of cyan for community 2; a click on the button shows details about correlated residues); and (**i**) acid/base catalytic or nucleophile (black)

To make Glutantβase a reference into the design of improved β-glucosidase enzymes, essential features to help researchers decide site-directed mutagenesis was included. Glutantβase includes 3842 structures of β-glucosidase enzymes. For each β-glucosidase, we constructed an individual page with a 3D-model and classified the role of some amino acids (Fig. 1a-b).

The β-glucosidase’s active site is composed of a glutamate pair, acid/base catalytic and nucleophile [60, 76]. We predicted and showed the position of both glutamic acid amino acids in all sequences (Fig. 1c). The literature has described that mutations in these residues lead to loss of activity [59, 77]. Therefore, to design improved β-glucosidases, mutations in these residues are not recommended. The same recommendation can be assigned for conserved positions (Fig. 1f-g), although the impact of mutating most of these amino acids have not been established. Besides, information about conserved amino acids could be combined with the coevolutive network data to give new insights into mutations to be experimentally tested (Fig. 1h).

The coevolutive networks indicate possible relationships between amino acid pairs in conserved positions. Residue coevolved networks are constructed based on the technique called statistical coupling analysis and community detection [64]. These approaches use multiple sequence alignments of a protein family to detect conserved amino acids and correlations among a set of residues considering all the sequences. In statistical coupling analysis, the conservation and coupling of amino acid residues are outcomes of evolutive restrictions. On the other hand, less conserved positions or with no correlation to other amino acids are classified as not important for the proteins in that family. Our analysis returned 11 network communities, although we exhibited only the two most populated: community 1 colored in magenta and community 2 colored in cyan (details will be discussed in the next sections). For example, we detected that the appearance of phenylalanine in position 442, highlighted in Fig. 1h, is correlated to other residues, such as P200, E433, F96, Y498, T378, W434, H219, Y94, and N431. This suggests that substitution of the residue F442 is complemented by modifications in P200, E433, F96, Y498, T378, W434, H219, Y94, and N431, based on other occurrences in proteins from GH1 family. This could be taken into consideration if Glutantβase’s users decide to mutate these residues.

Β-glucosidases present an (α/β)_8_ TIM barrel folding, with an active site located at the bottom of a channel [7, 13, 26]. Several residues present in this region, known as the substrate channel or catalytic pocket, have been reported as important for substrate entrance and glucose withdrawal [9, 10, 45]. Previously, a set of 22 residues was reported as part of the substrate channel in glucose-tolerant β-glucosidases [4]. However, only half of them were conserved in most sequences, which may indicate that various combinations of amino acids in this region could take to glucose tolerance characteristics. Glutantβase shows substrate channel residues with orange letters (Fig. 1e). Non-conserved residues in the substrate channel are candidates for initial studies to determine their role in the saccharification process.

Another feature worth mentioning is the secondary structure prediction (Fig. 1c). Recent studies have highlighted the importance of loops in the substrate’s entrance channel. Fang et al. [28] suggested that the geometry of loop C of β-glucosidases could be related to glucose tolerance characteristics. Costa et al. [45] reported an allosteric channel between B and C loops that, together with protein’s motions, promotes changes in the water’s dynamics in the region, which supports the glucose withdrawal. Hence, secondary structure data, combined with other visualizations, could be useful for decision making.

### 3D-models

**Fig. 2:**
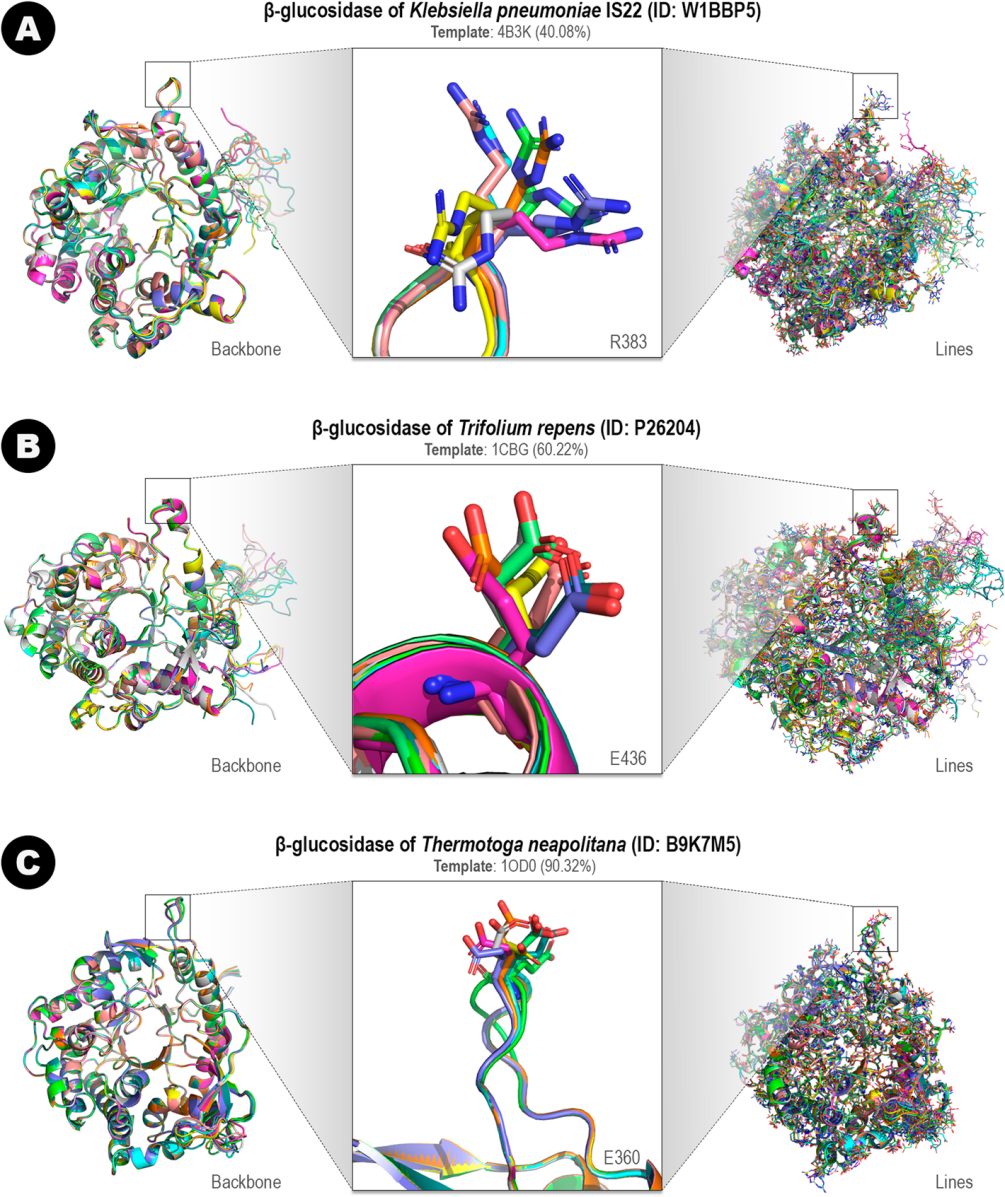
Variability of the ten best models constructed by comparative modeling for three β-glucosidases: (**a**) *Klebsiella pneumoniae* (40% of identity with the template of PDB ID: 4B3K); (**b**) *Trifolium repens* (60% of identity with the template of PDB ID: 1CBG); and (**c**) *Thermotoga neapolitana* (90% of identity with the template of PDB ID: 1OD0). For each protein (**a**, **b**, and **c**), ten models were superimposed using PyMOL software [78]. On the left, we showed the protein backbone as a cartoon of ten best models superimposed. On the right, we showed all residues as lines of the ten best models superimposed. In the center, we arbitrarily highlighted one amino acid: (**a**) R383, (**b**) E436, and (**c**) E360

Each entry of Glutantβase presents a 3D-model available to download (each entry is identified using the UniProt ID of the sequence). The models were constructed by comparative modeling using the MODELLER software (see methods section). MODELLER’s algorithm uses a known 3D-structure with the highest identity sequence (called template) to the target sequence and constructs models of the target based on spatial restrictions imposed by atoms of the template’s backbone. Comparative modeling is a computational alternative to represent protein structures not experimentally determined. Hence, we constructed 100 different models for each entry. In Fig. 2, we depict the variability of models constructed for three β-glucosidase sequences modeled using templates with 40, 60, and 90% of identity (comparative modeling requires at least 25% of sequence identity).

Comparative modeling uses the template’s backbone structure as a reference to construct the models. Therefore, models present a similar backbone structure when superimposed (Fig. 2a-c; left). The orientation of the side chains is defined using stereochemical restraints (bond length and angle) obtained from the CHARMM-22 molecular mechanics force field [79] and statistical preferences collected from a set of known structures for dihedral angles and non-bonded interatomic distances [55, 80]. Loops and other regions not covered by the sequence alignment are defined using an ab initio prediction strategy. Thus, each modeling attempt produces a variability range of different results (Fig. 2a-c; right). To define one representative model for each entry is necessary, an assessment step of all produced models. We selected only the best model based on the DOPE energy score to show in the 3D-panel. Also, we constructed a Ramachandran plot to verify residues with non-permissive positions. All these data are available on the protein’s entry page.

### Is it possible to extrapolated known beneficial mutations to other β-glucosidases?

We suggested mutation sites based on six mutations reported in the literature as responsible for improving β-glucosidase activity or stability (Fig. 1d). To evaluate if new mutations could lead to the same effect, we performed a test using molecular docking. Our experiment consisted of docking glucose and cellobiose molecules into the wild and the mutant proteins. As the β-glucosidase inhibition occurs in high glucose concentration, we expected that glucose-tolerant β-glucosidases showed a lower affinity for glucose (low capacity to keep the product in the active site pocket) and a higher affinity for cellobiose (high capacity to attract the substrate to the active site pocket).

We based our hypothesis on experimental data reported in the literature. Initially, we calculated the docking for the beneficial mutation (H228T) of the non-tolerant β-glucosidases from a marine metagenome (Bgl1B; Uniprot ID: D0VEC8), obtained from the study of Yang et al. [9]. This mutation has been described in the literature as favorable for increasing the β-glucosidase activity even at high concentrations of glucose. Hence, we expected that the mutant had a higher affinity for cellobiose (substrate) and a lower affinity for glucose (product). For the wild modeled protein, we obtained an affinity of − 5.94 kcal/mol for cellobiose and − 5.80 kcal/mol for glucose. For the mutant modeled protein, we obtained an affinity of − 6.43 kcal/mol for cellobiose and − 5.76 kcal/mol. Hence, we got the expected affinity values variation for cellobiose (a negative affinity variation of − 0.49 kcal/mol) and glucose (a positive affinity variation of + 0.04 kcal/mol). The reduction of glucose binding affinity to the active site in the mutated enzyme, observed here, agrees with the docking study that corroborates the experimental data presented in [9]. Our results also agree with other computational studies that verified the relation of this amino acid position for the interaction with the substrate and ligand [44–46]. Thus, we hypothesized that these results could be extrapolated to other β-glucosidases. Therefore, we detected analogous residues and repeated the same experiment to all modeled wild and mutant β-glucosidases from Glutantβase. In addition, we decided to verify if mutations in other sites reported as beneficial (A404V, E96K, H184F, L441F, and V174C) could present a similar impact in the interaction with substrate or product.

Since molecular docking scores are binding affinity approximations, we created a protocol to maximize sampling and ligand conformations. For each wild and mutant structures, we performed docking in triplicate for glucose and cellobiose. For each docking run, we collected the ten highest affinity poses returned by Autodock Vina. To define the variance in the affinity, we analyzed score for only the first pose (1), poses from the first to the third (1–3), and poses from the first to the tenth (1–10). Since results had similar values, we will describe only results for poses 1–3 (we used the average of affinity scores).

For cellobiose, we expected that a mutation would improve the affinity score from docking, i.e.*,* negative variation values when comparing wild and mutant scores. Therefore, cellobiose docking should achieve higher (more negative) scores in a mutant structure than in the wild one. However, only H228T derived mutants presented an improvement in affinity for cellobiose (with significant statistical values; Table 2).

**Table 2:** Docking results and hypothesis test for each protein wild and mutant docked to cellobiose (affinity score average for poses 1–3). For affinity scores, lower values represent more affinity. Affinity score variation (ΔAS)

Substrate (cellobiose)						
Mutation	Affinity score (wild; 1–3)	Affinity score (mutant; 1–3)	Variation (ΔAS)	ΔAS expected	***p***-value	status
A404V	−4.6347	−4.6057	0.0290	ΔAS < 0	1	X
E96K	−4.8509	−4.8341	0.0168	ΔAS < 0	1	X
H184F	−4.7473	−4.7009	0.0463	ΔAS < 0	1	X
H228T	−4.4806	−4.6509	−0.1703	ΔAS < 0	< 2.2e-16	✓
L441F	−4.8407	−4.8239	0.0169	ΔAS < 0	0.9031	X
V174C	−5.1818	−5.1669	0.0149	ΔAS < 0	1	X

For glucose, we expected that a mutation would decrease the affinity score from docking. In this case, the variation between wild and mutant scores should be positive, since glucose docking in the mutant structures would have a less (more positive) affinity. Two derived mutants of H184F and H228T showed a reduced affinity for glucose (with significant statistical values; Table 3).

**Table 3:** Docking results and hypothesis test for each protein wild and mutant docked to glucose (affinity score average for poses 1–3). For affinity scores, lower values represent more affinity. Affinity score variation (ΔAS)

Product (glucose)						
Mutation	Affinity score (wild; 1–3)	Affinity score (mutant; 1–3)	Variation (ΔAS)	ΔAS expected	p-value	status
A404V	−5.2930	− 5.2988	−0.0059	ΔAS > 0	1	X
E96K	−5.3079	−5.3083	−0.0004	ΔAS > 0	0.7174	X
H184F	−5.2912	−5.2567	0.0345	ΔAS > 0	< 2.2e-16	✓
H228T	−5.3664	−5.3149	0.0515	ΔAS > 0	< 2.2e-16	✓
L441F	−5.2366	−5.2561	−0.0195	ΔAS > 0	1	X
V174C	−5.3045	−5.3077	−0.0032	ΔAS > 0	0.9979	X

Our results show that only the mutation of a histidine to a threonine, at position 228 of the marine metagenome β-glucosidase, can lead to a ligand affinity similar to a glucose-tolerant β-glucosidase. These results concur with previous studies that reported exchanging histidine 228 for small amino acids (such as threonine) capable of acting as a hydrogen bond acceptor could improve the catalytic activity even in high glucose concentrations [9]. Also, the role of the residue in position 228 has been established in a molecular dynamics study [45]. When glucose is trapped in a hydrophobic region of the substrate channel, the residue D238 (analogous position to H228) takes part in a set of interactions that culminates in the expelling of glucose from the site (slingshot mechanism). Our results further characterize position 228 importance in the interaction process with ligand and substrate.

### Case study: β-glucosidase of Streptomyces sp. Root1295

To illustrate the webtool, we present an analysis of the *Streptomyces sp. Root1295* β-glucosidase (from now on labeled SrBGL; UniProt ID: A0A0Q7I6P6)*.* E184 and E379 are the acid/base catalytic and the nucleophile amino acids (Fig. 1c, i). Residue E184 was appointed this function in SrBGL since its position corresponds to the glutamate E745 (global position 745) found in the multiple sequence alignment. The same occurs to E379, which corresponds to E1567 in the global position. The secondary structure analysis also reveals that they are in the terminal region of β-strand 4 and 7, which matches the expected positions of the active site residues. Also, a visual analysis of the 3D-structure shows that the predicted amino acids are at the bottom of the substrate channel, which corroborates with our expectation of a correct prediction.

**Fig. 3:**
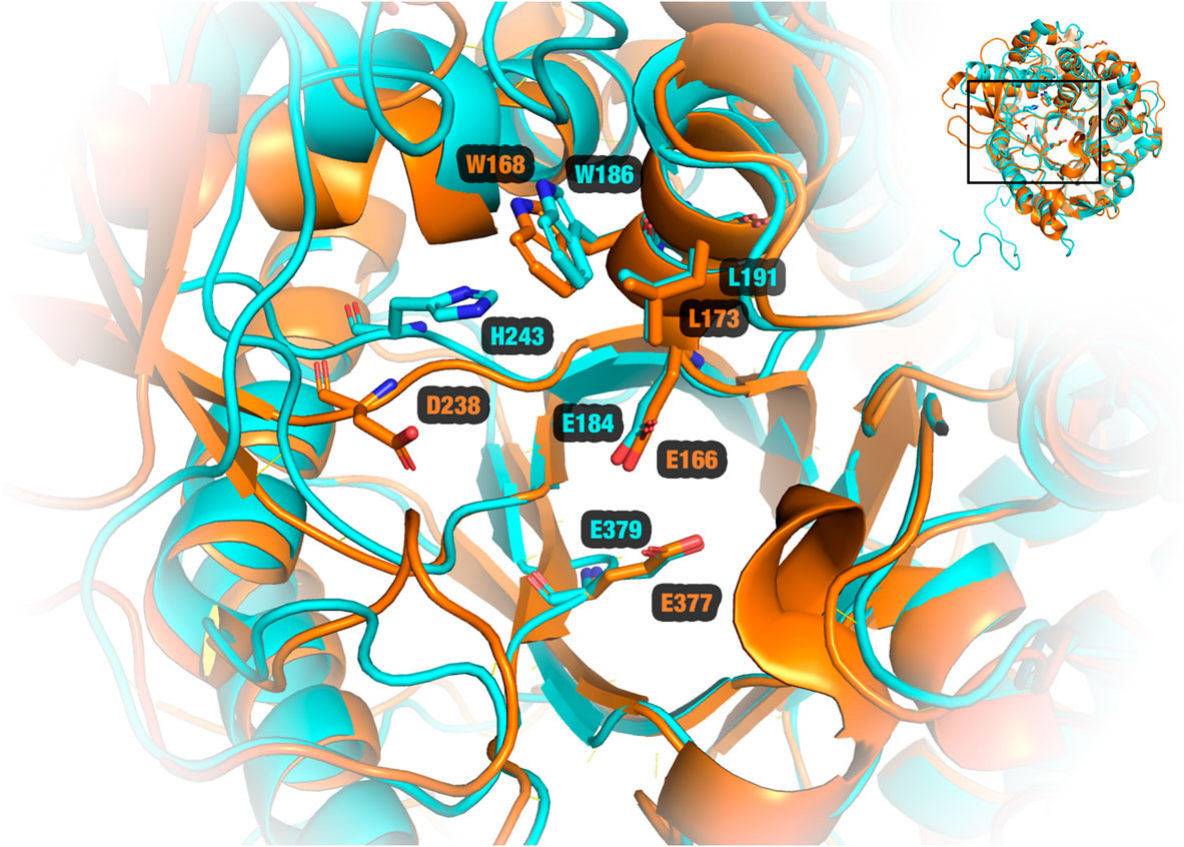
Structural alignment between the SrBGL model (cyan) and the crystal structure of the glucose-tolerant GH1 β-glucosidase from the fungus *Humicola insolens* (PDB ID: 4MDP; orange)*.* Analogous positions: E379- > E377 (nucleophile), E184- > E166 (acid/base catalytic), W168- > W186 (hydrophobic region), L191- > L173 (hydrophobic region), and H243- > D238 (slingshot mechanism). Image generated using PyMOL [78]

We detected 23 residues in the substrate channel: Q38, H139*, W140*, N183*, E184*, W186, C187, F190, H198, N241, L242, I261, N313*, Y314*, Y315, S316, S318, W352, E379*, W426*, E433*, W434*, and F442*. SrBGL presents 11 highly conserved amino acids in the substrate channel of glucose-tolerant β-glucosidases (shown previously by a *) [4]. SrBGL introduces W186 and L191, amino acids in analogous positions to W168 and L173 of the glucose-tolerant β-glucosidase of *Humicola insolens* (PDB ID: 4MDP; Fig. 3). Both amino acids are important for glucose tolerance mechanisms because they restrict access to the active site [10]. They act in collaboration with D238 to release glucose in the so-called slingshot mechanism [45]. Since in SrBGL a histidine is found in this position, this might suggest that SrBGL cannot perform the slingshot mechanism.

Glutantβase predicted three mutations for SrBGL: (i) E113K, analogous position to E96K [47]; (ii) H198F, analogous position to H184F [27]; and (iii) H243T, analogous position to H228T [9]. We observed a reduction of affinity between protein and product (glucose) for H243T and H198F mutants in all docking poses analyzed (Table 4). This suggests that these mutations could reduce the glucose inhibition of SrBGL, which could amplify its potential for hydrolyzing cellobiose for industrial purposes.

**Table 4:** Docking results for *Streptomyces sp. Root1295* β-glucosidase. Affinity score variation (ΔAS) negative values show the improved affinity between protein and ligand (the more negative scores, the stronger the affinity)

Mutation	Docking	Poses (average)	Affinity Score (wild)	Affinity Score (mutant)	ΔAS	ΔAS expected	Status
H243T	Cellobiose	1	−6.23	− 6.13	−0.1	ΔAS < 0	x
		1–3	−6.07	−6.08	−0.01	ΔAS < 0	✓
		1–10	−5.35	−5.53	−0.18	ΔAS < 0	✓
	Glucose	1	−6.23	−5.83	0.4	ΔAS > 0	✓
		1–3	−6.07	−5.61	0.46	ΔAS > 0	✓
		1–10	−5.44	−5.37	0.07	ΔAS > 0	✓
H198F	Cellobiose	1	−6.23	−6.33	−0.1	ΔAS < 0	✓
		1–3	−6.07	−6.19	−0.12	ΔAS < 0	✓
		1–10	−5.35	−5.32	0.03	ΔAS < 0	x
	Glucose	1	−6.23	−5.90	0.33	ΔAS > 0	✓
		1–3	−6.07	−5.67	0.4	ΔAS > 0	✓
		1–10	−5.44	−5.43	0.01	ΔAS > 0	✓
E113K	Cellobiose	1	−6.23	−6.23	0	ΔAS < 0	x
		1–3	−6.07	−6.02	0.05	ΔAS < 0	x
		1–10	−5.35	−5.00	0.35	ΔAS < 0	x
	Glucose	1	−6.23	−5.87	0.36	ΔAS > 0	✓
		1–3	−6.07	−5.67	0.4	ΔAS > 0	✓
		1–10	–	–	–	ΔAS > 0	–

The E113K mutant presented few differences for cellobiose docking. Also, for unknown reasons, the glucose docking did not return poses enough for 1–10 poses analysis, which prevents us from having more accurate conclusions. The E113K mutation occurs on the surface of the protein, distant to the substrate channel. This mutation is based on the E96K mutant of *Bacillus polymyxa* β-glucosidase (PDB ID: 1BGA) [47]. E96K has been previously reported as responsible for improving the protein structure thermostability [81]. Thus, we expected that this mutation would not impact the interactions between ligand and protein. We also should mention the possibility of the molecular docking method not being able to detect the impact of this mutation in the protein structure. SrBGL case study is available at http://bioinfo.dcc.ufmg.br/glutantbase/protein/id/A0A0Q7I6P6.

### Thermostabilizing mutations are positioned in a coevolutive network

We included in Glutantβase, the corresponding amino acids of the GH1 family found in coevolutive networks communities. This step aims at extending the analysis of conserved amino acids to residue-residue connections. The study of residue-residue coevolutive networks by statistic coupling is useful for analyzing conserved protein families [82–85] and identifying important residues in protein folding and stability [83, 85–88].

In Glutantβase, we suggest the use of coevolved residue networks to highlight possibly essential amino acids for the protein structure and function. Mutations in residues of the coevolved network or their neighbors could affect the protein function, causing changes in thermostability. In addition, a list of correlated mutation pairs could be used to identify double mutations found in other sequences of the GH1 family.

Thus, we used a recent version of this technique known as DRCN [64] for the available GH1 family sequences. With DRCN, it is possible to deconvolute conserved networks in the same family in different subsets known as communities. This analyzes can reveal subnetworks affecting different parts and functions of the protein or located in different GH1 subclasses.

### Case study: β-glucosidase A of *Bacillus polymyxa*

To illustrate coevolutive analysis, we will use as a model, the structure of *Bacillus polymyxa* β-glucosidase A (PDB ID: 1BGA; from now on labeled BgA). Studies have reported a collection of mutations that enhance BgA’s thermoresistance, such as E96K and M416I [89].

**Fig. 4:**
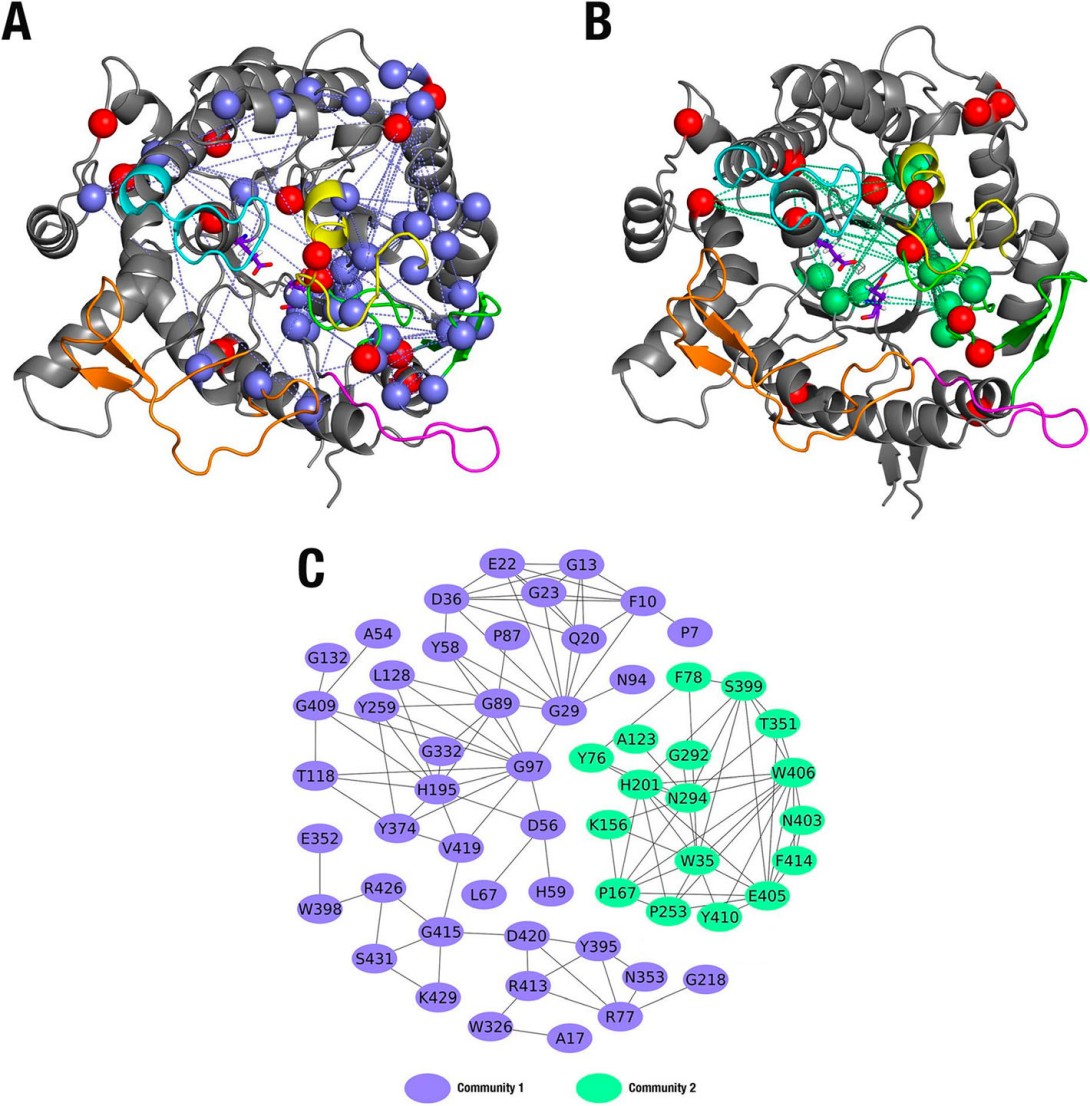
Groups of residues involved based on coevolution analysis. **a**–**c***Bacillus polymyxa* β-glucosidase (PDB ID: 1BGA) is represented as a cartoon. The colors yellow, cyan, orange, magenta, and green represent loops **a**–**e**, respectively. Catalytic residues (E166 and E352) are presented in purple. The red spheres represent thermostabilizing mutations described for GH1 β-glucosidases in the literature [85, 89] and from a thermophile GH1 structure (PDB: 5IDI). **a** Blue spheres depict community one residues. **b** Green spheres depict community two residues. **c** Graph of coevolving residues and their communities

Highly correlated positions were clustered in 11 coevolved sets (not shown). However, only two communities showed a significant number of descriptive residues and average conservation. Hence, only these two were included in Glutantbase. The communities were named, in decrescent order of the number of residues, as community 1 (41 descriptive residues for GH1 family) and community 2 (19 descriptive residues; Fig. 4).

Community 1 describes a long-range network of residues evolutionarily correlated. It includes the internal β-barrel (between them, the catalytic E352) and a set of mobile regions at the loops and helix around the protein surface (Fig. 4a, c). Community 2 is centered on the active site and surroundings (Fig. 4b-c). Together, both communities (Fig. 4) suggest the existence of a multi-correlated evolutive network integrating all globular protein cores and active site loops. Interestingly, the positions described in the literature to influence thermostability are retrieved or located nearby this same coevolutionary network (Fig. 4) [85, 89].

For the E96K mutant, the residue found in position 96 is closely surrounded or is distance-compatible with electrostatic influence (approximately 12 Å) by a set of residues from community 1 (Fig. 4). Between them, two glycine amino acids were found as a set of highly coevolved partners. The first is G29, which is evolutionarily correlated with ten residues from the same community. G29 is located in loop A and presents a distance compatible with contacts with the side chain residue 96 and its close neighbor, N94. The second is G97, the immediate neighbor to the amino acid in position 96 in helix 7, which is evolutionarily correlated with 11 other residues, including G29 (Fig. 4a, c). The evolutionary correlations involving these two specific residues spread around loops A and E, the catalytic β-barrel, and even loops and helices on the opposite side of the protein (Fig. 4a). From the 21 residues evolutionarily influenced by the G29-G97 pair, 18 are in highly mobile regions (loops, interfaces between loops, helices, or ribbons).

Additionally, the two residues G29-G97 are very close to the two respective aspartate residues in a position compatible with transitory salt bonds with the residue in position 96, D28, and D99, respectively. The residue R30, a neighbor to G29 from community 1 in loop A and near residue 96, was previously suggested to have a thermostabilizing effect when substituted for alanine in *Spodoptera frugiperda* β-glucosidase [85]. The substitution of the positive residue at position 30 disrupts the salt bridge with D36 (community 1). β-Glucosidase from the thermophile *Thermotoga neapolitana* (PDB ID: 5IDI), in addition to the two substitutions E96K and M416I, also showed disruption of the conserved internal salt bridge in loop A, with an alanine in position 30 and a histidine opposed to the conserved aspartate in position 36 from community 1.

For the M416I mutant, the residue in position 416 has numerous neighbors from communities 1 and 2 (Fig. 4). From community 1, the closest neighbors are: (i) R413 and G415 in loop E; (ii) W398 in the active site β-barrel, in close contact with the catalytic E352 and evolutionarily correlated with it; and (iii) K429 and S431 in the N-terminal extremity of helix 22, located at the protein surface and packed against position 416. From community 2, the closest neighbors to position 416 are: (i) F414 in loop E; and (ii) S399 in the C-terminal extremity of ribbon 12 of the catalytic β-barrel. Between the residues surrounding position 416, W398 (community 1) and F414 (community 2) have been reported in the literature as necessary for substrate interaction and stabilization of the transition state [4, 47]. In addition, a set of residues from communities 1 and 2 are preserved around the two respective β-hairpins motifs in the two opposite extremities from loop E. Between these motifs, the W406 residue from community 2, placed in the β-hairpin motif bordering the active site, is involved in substrate recognition and transition state stabilization [4, 47]. Also, it is evolutionarily correlated with F414 (near to position 416). In the superficial β-hairpin motif opposite from loop E, community 1 residues V419, D420, and R426 participate in a local evolutionary subnetwork with closer contacts to position 416 (W398, R413, and G415). This suggests a collaborative behavior of all residues under the dynamic and topological influence of the position 416.

Furthermore, the evolutionary correlations above-described affect the entire protein surface, with significant participation of mobile or functional positions in the active site loops or catalytic β-barrel. At least two other thermostabilizing substitutions documented in the literature in the same neighborhood, N437K (helix 22) and N411S (loop E), support its importance [89]. Additionally, N411S is close to position 416 and is involved in the shortening of the side chain, which enhances the mobility of loop E.

Our results suggest the existence of a multi-coevolutionary network for all protein structures in the GH1 family. A set of potentially thermostabilizing positions appears to be strategically allocated along this network and neighborhood to modulate its topology or dynamics. It is important to highlight that this network was previously reported in a statistic coupling study for this family by Tamaki et al. [85]. However, because of the limited availability of GH1 sequences deposited in the free databases at the time (the authors used 768 sequences, while our study analyzed 4084 sequences), the previous study provided a considerably limited view of the network. Compared to the 61-residue network (considering the two communities in BgA) recovered here, Tamaki et al. recovered a network of 23 covariant positions, most of which correspond to our community 2. The BgA’s case study is available at http://bioinfo.dcc.ufmg.br/glutantbase/protein/id/P22073.

## Conclusions

In this study, we presented Glutantβase: a database of β-glucosidase structures and several predicted features. Β-glucosidases are vital enzymes for saccharification process that has been target of many studies since they represent a bottleneck for second-generation biofuel production. More glucose resistant β-glucosidases are essential to saccharification in industrial applications. The web-based tool and the database introduced here provide a powerful source of features for supporting the rational design of β-glucosidase enzymes. Glutantβase provides information about catalytic amino acids, conserved amino acids, residues found in a coevolution network, protein secondary structure, and residues in the channel that guides to the active site. We also suggested mutations for the Glutantβase’s structures based on six mutations described in the literature as able to improve catalytic activity or thermostability (A404V, E96K, H184F, H228T, L441F, and V174C). The molecular docking score was used to verify the impact of the suggested mutations in the affinity of protein and ligands (substrate and product). Our results suggest that only mutations based on the H228T mutant presented reduced affinity for glucose (product) and increased affinity for cellobiose (substrate), which shows an improvement in the resistance to product inhibition. We intend to automatize the insertion of newly discovered β-glucosidase sequences in Glutantβase. Therefore, we hope that Glutantβase is useful for the design of more efficient β-glucosidases, which may help to improve second-generation biofuel production. Glutantβase is available at http://bioinfo.dcc.ufmg.br/glutantbase.

## Supplementary information

**Additional file 1: Table S1.** Templates used for modeling each structure of Glutantβase.

**Additional file 2: Table S2.** The DOPE score for each model selected.

## Data Availability

The datasets generated and/or analysed during the current study are available in the Glutantβase’s downloads page: http://bioinfo.dcc.ufmg.br/glutantbase/home/download.

## Abbreviations

BgA: *Bacillus polymyxa* β-glucosidase A
DRCN: Decomposition of residue coevolution networks
GH1: Glycoside hydrolase (Family) 1
PDB: Protein data bank
SrBGL: *Streptomyces sp. Root1295* β-glucosidase
