# Supplementary material for: Glutantβase: a database for improving the rational design of glucose-tolerant β-glucosidases
Source: BMC Mol Cell Biol. 2020 Jul 1;21:50. doi: 10.1186/s12860-020-00293-y (PMC7329481; doi:10.1186/s12860-020-00293-y)
Supplement: Supplementary file 1 — Additional file 1: Table S1. Templates used for modeling each structure of Glutantβase. [file 12860_2020_293_MOESM1_ESM.pdf]

Table S1. Templates used for modeling each structure of Glutantβase.

Uniprot ID corresponds to the same ID used in Glutantβase. PDB ID from the template is represented by a four-character code followed by the chain (only one chain was modeled). Sequence identity of local alignment using blastp tool between the target and template sequences. Sequence length, alignments start and end positions correspond to the template.

Uniprot ID	PDB ID (template)	Sequence identity	BLAST score	Sequence length	Alignment start position	Alignment end position
Q9H227	2E9L:A	100.00	2529	469	1	469
Q9SR37	3PTK:A	44.04	1093	505	15	503
Q9FYS3	1V02:E	58.35	1763	565	1	561
Q75I93	2RGL:A	99.17	2581	481	1	481
P37702	3PTK:A	49.17	1136	505	33	503
P49235	1V03:A	70.12	2142	565	1	561
Q8L7J2	3GNO:A	99.59	2626	488	3	488
Q1XIR9	1V02:E	58.27	1770	565	1	559
Q1XH05	1V02:E	58.07	1776	565	1	561
Q9C525	3PTK:A	44.81	1057	505	29	505
Q7XKV4	3PTK:A	98.58	2614	505	14	505
Q1XH04	1V02:E	58.99	1764	565	1	552
O64883	1CBG:A	48.64	1191	490	16	489
Q9C8Y9	3PTK:A	45.83	1074	505	29	503
D5MTF8	1V02:E	58.80	1779	565	1	563
O64879	3PTK:A	56.08	1461	505	19	503
Q9LV33	2RGL:A	63.98	1670	481	13	480
Q75I94	2RGL:A	76.28	2026	481	13	480
A3BMZ5	2RGL:A	64.88	1719	481	13	479
Q8GRX1	3PTK:A	53.67	1350	505	30	505
Q25BW5	2E3Z:A	100.00	2466	465	4	465
Q3ECS3	3PTK:A	53.67	1329	505	30	505
B9K7M5	1OD0:A	90.32	2190	468	25	468
P94248	1GNX:A	54.13	1183	479	16	467
Q41761	1V03:A	72.71	2228	565	1	561
P26205	1CBG:A	98.29	2172	490	1	409
Q9LZJ1	3GNO:A	38.99	962	488	12	486
Q9STP4	3GNO:A	43.83	1128	488	4	486
Q7XSK2	4QLK:A	48.41	1176	481	14	479
Q84WV2	1CBG:A	47.00	1103	490	14	488
P10482	3AHX:A	52.84	1260	453	4	444
Q9FMD8	2RGL:A	40.64	546	481	158	437
Q5N863	3GNO:A	49.06	1255	488	12	487
Q93ZI4	3GNO:A	43.12	1142	488	4	486
O64882	3PTK:A	55.16	1467	505	29	503
Q0DIT2	3GNO:A	42.47	1035	488	12	487
Q60DY1	3GNO:A	43.31	1089	488	12	487
Q84YK7	3PTK:A	59.54	1531	505	30	505
Q0J0N4	3PTK:A	59.03	1528	505	30	505
O48779	3PTK:A	46.99	1185	505	26	503
Q53NF0	3PTK:A	53.07	1310	505	22	505
P26208	4PTV:A	55.28	1356	452	2	445
Q59437	3TA9:A	36.36	728	458	8	450
Q8GVD0	3PTK:A	47.25	1258	505	4	505
P12614	1OD0:A	44.80	1047	468	25	462
P26204	1CBG:A	60.22	1443	490	10	468
B7F8N7	3GNO:A	45.29	1136	488	2	487

Q9ZUI3	2RGL:A	43.65	1019	481	15	480
Q7F9K4	3PTK:A	79.02	2137	505	20	505
Q9LU02	3PTK:A	56.58	1482	505	19	503
Q9M1D0	3PTK:A	56.49	1436	505	20	503
Q9LIF9	3PTK:A	45.64	1098	505	29	503
Q25BW4	2E3Z:A	64.32	1630	465	8	465
B9FHH2	3PTK:A	46.57	1070	505	31	503
Q4V3B3	3PTK:A	49.69	1258	505	27	503
Q8GXT2	3GNO:A	46.53	1251	488	12	486
Q9M1C9	3PTK:A	50.10	1277	505	29	505
Q9FZE0	3GNO:A	75.26	2061	488	12	488
Q8RXN9	3GNO:A	41.43	1031	488	1	487
Q7XKV5	3PTK:A	82.14	2153	505	20	505
Q9SLA0	3PTK:A	53.61	1358	505	20	503
Q60DX8	3GNO:A	45.40	1156	488	12	487
Q5Z9Z0	3PTK:A	65.62	1717	505	24	503
Q339X2	3GNO:A	81.61	2227	488	5	488
Q9FIU7	3GNO:A	60.83	1669	488	8	486
Q9FIW4	3GNO:A	52.10	1310	488	14	487
Q5QMT0	4QLK:A	67.02	1761	481	13	479
O65458	3PTK:A	46.54	1071	505	31	503
B8AVF0	3PTK:A	98.98	2623	505	14	505
Q6L597	3PTK:A	44.15	960	505	60	503
Q5JK35	3GNO:A	44.05	1115	488	11	487
Q0DA21	3GNO:A	59.03	1643	488	11	486
Q9M1D1	3PTK:A	50.84	1219	505	31	503
B7F7K7	3GNO:A	45.29	1145	488	5	487
Q2QSR8	2RGL:A	57.73	1477	481	15	480
O80689	3GNO:A	47.47	1142	488	17	487
P0C946	1OD0:A	90.59	2100	468	25	449
Q3ECW8	3GNO:A	42.44	1038	488	13	487
Q8RZL1	3PTK:A	46.86	1131	505	30	503
Q682B4	3GNO:A	47.35	859	488	1	321
Q67XN2	3GNO:A	41.00	1007	488	12	486
B3H5Q1	3GNO:A	44.11	1143	488	13	487
Q7XKV2	3PTK:A	87.81	2274	505	23	505
Q7XPY5	4QLK:A	42.16	455	481	276	479
Q7XSK1	2RGL:A	49.29	696	481	1	276
Q7XSK0	4QLL:A	46.53	1164	481	15	481
Q9FLU8	3PTK:A	51.55	1287	505	28	505
Q9SVS1	3GNO:A	45.44	1101	488	17	486
P22073	1BGG:A	100.00	2424	448	1	448
P38645	1GNX:A	52.19	1032	479	4	472
Q9FH03	3PTK:A	57.20	1484	505	19	503
Q9LKR7	3PTK:A	44.72	1079	505	29	503
Q7EXZ4	3PTK:A	57.62	1498	505	29	505
A3C053	3PTK:A	56.52	1496	505	23	505
Q9FLU9	3PTK:A	52.39	1291	505	28	503
Q0J0G2	3GNO:A	46.58	1158	488	5	486
Q9LV34	2RGL:A	60.54	1614	481	3	481
O80690	3GNO:A	47.26	1162	488	17	487
Q03506	1QOX:A	100.00	2402	449	1	449
Q08638	1OD0:A	100.00	2361	468	24	468
P22505	2JIE:A	100.00	2410	454	8	454
Q5RF65	2E9L:A	98.29	2493	469	1	469
P97265	2E9L:A	84.33	2160	469	1	466
Q51723	3WDP:P	99.36	2485	473	3	473

O08324	1VFF:A	63.88	1445	423	3	420
A0A075HLG3	1VFF:A	40.19	711	423	3	403
A0A0A7RBQ4	3WDP:P	76.59	1986	473	1	473
A0A0S1XBC4	1VFF:A	64.20	1468	423	3	421
A0A0K1E0N6	1VFF:A	26.57	271	423	48	383
R1FT37	1VFF:A	32.19	457	423	7	382
H6Q9A0	1VFF:A	24.50	257	423	45	383
C6A200	1VFF:A	76.74	1811	423	5	421
E7FHY4	3WDP:P	99.36	2485	473	3	473
Q9HIL6	3WDP:P	46.14	1125	473	4	469
M5C1D8	2E3Z:A	61.75	1590	465	8	465
Q8X214	3AHY:A	62.79	1589	473	9	470
M5C6H3	2E3Z:A	51.86	993	465	9	353
M5BYZ4	3AHY:A	39.24	437	473	259	471
M5C0E3	2E3Z:A	58.85	794	465	9	257
M1NQJ6	3AHY:A	74.36	1928	473	9	470
A0A0G2R2A3	3AHY:A	85.94	2080	473	14	461
A0A127SA86	3AHY:A	89.66	2259	473	8	471
B8P2C6	2E3Z:A	70.33	1776	465	8	462
A2QID8	2E3Z:A	31.26	486	465	8	440
A2QGL6	3AHY:A	65.28	1676	473	1	470
A0A084G332	3AHY:A	47.76	1102	473	9	466
Q12601	2E3Z:A	31.33	525	465	8	451
L7RVW5	3AHY:A	63.22	1659	473	1	473
Q4WBS7	2E3Z:A	33.12	551	465	8	451
Q9C122	2E3Z:A	34.13	715	465	7	465
Q870B5	3CMJ:A	71.88	150	465	25	56
Q870B6	2E3Z:A	35.70	820	465	5	465
H8XVY6	3AHY:A	60.47	1529	473	9	470
A0A0D3RLY6	3AHY:A	61.95	767	473	203	428
A0A076JRL8	3AHY:A	73.09	1876	473	9	470
A0A0L1I0Z5	3AHY:A	64.90	1660	473	9	470
B8M2Y0	3AHY:A	62.24	1570	473	9	471
Q9UUQ3	2E3Z:A	36.89	824	465	3	465
K4K173	3AHY:A	62.03	1576	473	9	471
L7T8J0	3AHY:A	72.67	1853	473	9	470
A0A0F8CX61	3AHY:A	62.31	1487	473	22	472
Q2UL94	3AHY:A	62.87	1501	473	1	428
B7Z536	2E9L:A	100.00	2431	469	20	469
D0VYR5	3AHZ:A	71.22	1872	487	4	479
A0A060NTE4	3AHZ:A	76.16	1282	487	129	430
H9CGR5	3AHZ:A	74.05	1937	487	6	479
A0A0E3URF5	4HZ6:A	34.30	633	444	5	440
A0A125T0U2	3AHZ:A	63.06	1598	487	9	478
A0A060NUP4	3AHZ:A	62.05	999	487	129	430
A0A060P1M7	3AHZ:A	53.97	886	487	129	430
A0A060NZS1	3AHZ:A	79.14	1308	487	129	430
Q86D78	3AHZ:A	49.04	1191	487	12	476
D5KQK3	3AHZ:A	56.17	1457	487	11	480
Q8T0W7	3AHZ:A	100.00	2562	487	2	479
Q9GSE6	3AHZ:A	57.26	1379	487	10	473
A0A060NR41	3AHZ:A	50.00	844	487	129	430
A0A060NUN8	3AHZ:A	76.49	1316	487	129	430
A0A059WHP2	3AHZ:A	64.47	1691	487	10	479
O61594	3AHZ:A	48.72	1182	487	12	473
D0VYR6	3AHZ:A	71.22	1872	487	4	479
D0VYS1	3AHZ:A	73.17	1906	487	4	479

D0VYR8	3AHZ:A	71.43	1879	487	4	479
D0VYS0	3AHZ:A	73.53	1918	487	4	479
D0VYR9	3AHZ:A	70.80	1848	487	4	479
A0A060NZS6	3AHZ:A	61.84	1008	487	129	430
A0A060P1N1	3AHZ:A	52.98	889	487	129	430
H9ZGE0	1CBG:A	62.60	1633	490	12	490
B8A1R1	3GNO:A	48.12	1240	488	12	487
Q53WW9	1V03:A	70.12	2142	565	1	561
K7W2F0	3GNO:A	49.58	1269	488	12	487
A0A0R4J4H8	3GNO:A	51.25	1323	488	12	488
E6NU37	3GNO:A	50.62	1302	488	9	487
B5M9E4	3PTK:A	55.22	1429	505	9	505
K3XH76	3GNO:A	48.74	1255	488	12	487
I1HU83	3GNO:A	48.95	1279	488	12	486
A0A077S2F2	3GNO:A	48.67	1265	488	8	486
A8TVR1	3GNO:A	52.41	1343	488	12	487
V4UC97	3GNO:A	51.26	1302	488	12	487
I3SYX2	3GNO:A	51.99	1332	488	12	487
A0A0A7HJS7	3PTK:A	37.43	306	505	317	502
J3L6Q8	3GNO:A	49.06	1236	488	12	487
U5KJR1	1CBG:A	69.83	1812	490	7	490
A0A0J8CLR4	3GNO:A	52.00	1345	488	14	487
A0A0J8CRP3	3GNO:A	52.00	1344	488	14	487
T1NU52	1OD0:A	43.37	1002	468	21	461
A0A0D9V904	3GNO:A	47.85	1261	488	10	487
A0A0D9YIM6	3GNO:A	49.06	1253	488	12	487
D8LGS1	3GNO:A	46.49	1052	488	69	487
B9SAQ1	3PTK:A	48.21	1123	505	30	503
D8LQW6	1VFF:A	33.21	288	423	200	409
M4F2P8	3GNO:A	50.00	1292	488	12	487
B9REH3	3PTK:A	58.25	1365	505	28	450
B9RI70	3PTK:A	63.75	1697	505	5	505
B9REG8	3PTK:A	57.35	1054	505	30	369
B9SAQ5	3PTK:A	38.55	283	505	328	503
B9RI71	3PTK:A	65.44	1709	505	15	503
B9REG9	3PTK:A	64.15	1700	505	30	505
B9SAQ2	3GNO:A	46.33	1148	488	13	487
B9REH5	3PTK:A	59.28	1159	505	28	387
B9S3T2	3GNO:A	55.81	257	488	401	486
B9SY45	3GNO:A	58.49	1565	488	12	487
B9RXP7	3GNO:A	46.22	1211	488	1	488
B9SAQ6	3PTK:A	48.99	1145	505	16	503
B9REF8	3GNO:A	72.02	2001	488	3	488
B9T4F7	2RGL:A	63.21	1673	481	13	481
B9RAJ3	3PTK:A	50.00	1113	505	26	450
B9S3R9	3PTK:A	53.68	1351	505	34	503
B9SAQ4	3GNO:A	48.91	896	488	13	333
B9RAJ2	3PTK:A	44.44	801	505	148	486
B9SAQ3	3PTK:A	48.43	1120	505	30	503
B9RWJ7	3GNO:A	44.52	914	488	68	485
B9S3R8	3PTK:A	58.26	1507	505	20	503
B9RM06	3GNO:A	50.10	1281	488	12	487
I1NUA2	3GNO:A	49.06	1255	488	12	487
A0A059D1K5	3GNO:A	54.66	1405	488	6	487
A0A089GG65	3GNO:A	50.72	1313	488	6	487
A0A061EYK8	3GNO:A	51.35	1306	488	8	487
A0A061EVC3	3GNO:A	51.66	1341	488	7	487

A0A0D2KQR5	2JIE:A	39.62	208	454	69	174
A0A0D2KRE6	3AHY:A	41.83	873	473	9	459
A0A0D2MLI4	1BGA:A	55.77	374	447	2	105
A0A0D2LRL7	1CBG:A	52.03	467	490	33	180
A0A0D2LSH8	1GNX:A	44.19	283	479	350	476
A0A0D2MNY3	3AHY:A	42.92	979	473	9	466
A0A0B2QUI9	3PTK:A	56.67	291	505	233	322
A0A0B2RHR0	3GNO:A	45.02	1130	488	11	487
A0A0B2QZT4	1CBG:A	65.03	1665	490	3	490
A0A0B2R063	1CBG:A	72.77	1875	490	10	490
A0A0B2P194	3GNO:A	48.08	1185	488	21	487
A0A0B2QKD3	3GNO:A	38.03	119	488	186	256
A0A0B2RAL2	3PTK:A	37.06	576	505	167	505
A0A0B2QKY1	3GNO:A	48.84	1011	488	104	488
A0A0B2QDY0	3PTK:A	43.14	230	505	216	317
A0A0B2SPS7	3GNO:A	41.00	943	488	14	487
A0A0B2RL45	1CBG:A	69.77	1634	490	61	490
A0A0B2RFK5	1CBG:A	60.24	1332	490	10	434
A0A0B2R068	1CBG:A	68.12	1773	490	10	489
A0A0B2RR43	1CBG:A	68.97	1807	490	14	490
A0A0B2RYB1	3GNO:A	45.97	1117	488	21	486
A0A0B2PT59	3W53:A	44.09	213	506	44	136
A0A0B2PM93	3GNO:A	45.93	841	488	13	352
A0A0B2QNY1	1CBG:A	64.07	1419	490	14	434
A0A0B2QPR9	1CBG:A	68.34	1779	490	14	490
A0A0B2R4L8	3PTK:A	58.19	1480	505	29	503
A0A0B2R7W9	3GNO:A	47.70	1213	488	11	486
A0A0B2P7S5	3GNO:A	70.97	261	488	12	73
A0A0B2S898	3GNO:A	42.11	237	488	235	329
A0A0B2QG59	3GNO:A	73.65	2037	488	6	487
A0A0B2SQQ1	3PTK:A	56.12	1343	505	73	505
A0A0B2S9R3	3GNO:A	53.33	76	488	458	486
A0A0B2REW1	3GNO:A	45.23	1131	488	11	487
A0A0B2R9G2	3GNO:A	58.07	1590	488	12	487
A0A0B2R789	3GNO:A	45.11	1175	488	11	486
A0A0B2RNP1	1CBG:A	69.81	1819	490	14	490
A0A0B2QCU1	3PTK:A	60.42	1493	505	29	503
A0A0B2R1N8	3WDP:P	35.54	221	473	299	460
A0A0B2RQU5	3GNO:A	29.90	289	488	124	327
A0A0B2R9R7	1CBG:A	63.81	306	490	102	206
A0A0B2R6G1	3PTK:A	53.89	1359	505	31	505
A0A0B2RW08	3GNO:A	57.98	423	488	139	257
A0A0B2S984	3PTK:A	60.63	1486	505	29	503
A0A0B2P111	3GNO:A	47.08	1190	488	11	486
A0A0B2PAJ2	1CBG:A	54.19	505	490	10	188
A0A0B2R4M4	3PTK:A	51.86	1315	505	1	483
U5DDI2	3GNO:A	53.70	1407	488	6	486
A0A0B2S1N9	3GNO:A	60.44	330	488	397	487
A0A0B2RIG5	3GNO:A	45.22	1166	488	17	486
A0A0B2P7N9	3GNO:A	78.26	408	488	397	488
A0A0B2QUJ4	1CBG:A	69.65	1790	490	10	490
A0A0B2P7I6	3GNO:A	67.68	770	488	79	276
A0A0B2PQR3	1CBG:A	75.00	279	490	61	128
A0A0B2RUW4	3GNO:A	38.98	784	488	12	396
A0A0B2RCK9	1CBG:A	59.76	1492	490	3	489
A0A0B2RH44	1CBG:A	68.76	1806	490	14	490
A0A0B2RML7	3PTK:A	66.82	1614	505	72	505

A0A0B2PA60	1CBG:A	67.85	1745	490	12	489
A0A0B2QN99	1CBG:A	60.61	844	490	7	303
A0A0B2RXI2	1CBG:A	54.23	361	490	140	281
A0A0B2QHN2	3GNO:A	46.30	135	488	435	488
A0A0B2QVE6	2RGL:A	63.90	1580	481	38	479
A0A0B2SCY0	2RGL:A	63.93	1424	481	82	479
A0A0B2QZ86	1CBG:A	69.02	1788	490	10	490
A0A0B2S5K0	3GNO:A	43.63	1062	488	14	487
A0A0B2SW01	3GNO:A	40.29	887	488	12	486
A0A0B2SD69	3GNO:A	46.41	1169	488	2	487
A0A0B2PFA1	3PTK:A	50.36	1048	505	88	497
F6H7V7	3GNO:A	52.61	1356	488	10	487
Q93X78	3GNO:A	66.52	869	488	128	348
B6ZKM3	3PTK:A	49.90	1214	505	29	500
R0GZG2	3GNO:A	51.89	1323	488	14	487
V4LAH2	3GNO:A	51.57	1305	488	14	487
D8S991	3GNO:A	56.93	1466	488	12	486
A0A0E0N689	3GNO:A	48.85	1246	488	12	487
H9ZGD9	1CBG:A	63.52	1621	490	5	490
H9ZGE1	1CBG:A	62.60	1631	490	12	490
H9ZGD8	1CBG:A	63.52	1618	490	5	490
H9ZGE2	1CBG:A	63.52	1619	490	5	490
Q43014	1CBG:A	63.09	1641	490	12	490
A0A0E0JS71	3GNO:A	48.64	1250	488	12	487
C5XFD2	3GNO:A	48.33	1252	488	12	487
Q9SPK3	1CBG:A	61.22	1569	490	14	490
W0C065	1CBG:A	62.27	1622	490	12	490
W0BUD8	1CBG:A	62.89	1644	490	12	490
W0BZB0	1CBG:A	63.02	1637	490	12	490
A0A0D3BZC1	3GNO:A	50.42	1296	488	12	487
W0BWR5	1CBG:A	62.60	1626	490	12	490
A0A0K9R8Z7	3GNO:A	50.31	1325	488	3	487
A0A0A0LHE2	3GNO:A	51.36	1316	488	12	487
A0A0D2RJD0	3GNO:A	52.18	1343	488	8	487
A0A0D2SVQ2	3GNO:A	51.78	1333	488	12	487
V7BWU2	3GNO:A	51.46	1321	488	12	487
M1BC60	3GNO:A	50.73	1308	488	12	487
Q14QP8	3PTK:A	62.29	1591	505	30	501
A0A151QR95	1GNX:A	60.00	176	479	419	468
W8SAR2	3PTK:A	57.79	631	505	242	440
A0A151QXB7	3GNO:A	68.97	1909	488	12	488
A0A151RKM9	1CBG:A	45.30	1018	490	14	489
A0A151UAQ5	3GNO:A	73.74	2015	488	12	487
A0A151QVT5	3PTK:A	60.97	1306	505	114	505
A0A151REC3	3GNO:A	46.57	1199	488	11	486
Q700B1	1CBG:A	61.16	1575	490	10	490
B9HYG4	3GNO:A	51.99	1332	488	12	487
C0PT85	3GNO:A	55.04	1397	488	12	486
Q08IT7	1CBG:A	64.62	1653	490	3	490
K4D5D2	3GNO:A	50.73	1316	488	12	487
A0A0D6R2S3	3GNO:A	53.64	1383	488	7	486
A0A078CC24	3GNO:A	50.21	1291	488	12	487
A0A078HIJ6	3GNO:A	50.00	1286	488	12	487
Q42618	3PTK:A	45.68	1064	505	29	496
A0A078HP64	3GNO:A	52.10	1320	488	14	487
A0A0E0FX18	3GNO:A	49.06	1255	488	12	487
A0A087GTX2	3GNO:A	50.94	1314	488	12	487

A0A067GNY9	3GNO:A	51.26	1303	488	12	487
Q7Y073	3GNO:A	49.90	1285	488	12	487
U6C7K7	3GNO:A	52.20	1354	488	12	487
G3LY19	1CBG:A	64.40	1670	490	5	490
Q6V8P8	3PTK:A	78.12	135	505	472	503
A0A0D3EXD4	3GNO:A	49.06	1255	488	12	487
D7MI42	3GNO:A	52.31	1321	488	14	487
O24524	3PTK:A	51.50	1205	505	73	503
I0YK64	3GNO:A	48.71	1195	488	21	486
Q5UB04	1CBG:A	62.26	1596	490	14	490
I6ZYM4	1UYQ:A	35.95	673	447	5	438
Q9RA61	3ZJK:A	99.77	2220	431	1	431
A0A0I9PUS2	4PTV:A	46.31	1054	452	7	444
V9XM14	3TA9:A	34.58	507	458	65	449
A0A0U0C9H4	1UYQ:A	33.05	594	447	5	441
A0A0Y9CRS5	5FOO:A	47.63	1150	480	1	460
A0A155V2V5	3TA9:A	48.02	426	458	16	184
A0A155V0Z7	5DT5:A	47.37	249	471	356	464
Q2WGB4	1BGG:A	75.39	1949	448	1	447
Q9EXF8	1UYQ:A	33.40	596	447	5	441
W0FLD1	1OD0:A	45.54	977	468	27	466
Q8GEB3	3ZJK:A	99.54	2215	431	1	431
A8WAC9	1NP2:A	98.61	2211	436	1	431
Q8GEB4	3ZJK:A	98.61	2195	431	1	431
A0A0B4IY53	3TA9:A	45.45	1029	458	8	450
J9ZZW8	1UYQ:A	34.12	660	447	4	441
Q60026	5DT5:A	55.94	1394	471	25	460
A0A0L6CF69	1GNX:A	49.57	1041	479	18	472
L0EK01	1OD0:A	48.87	1084	468	26	465
W1B1K5	4B3K:A	45.04	1067	479	3	463
Q8GHE5	1NP2:A	98.84	2209	436	1	430
W1BBP5	4B3K:A	40.08	963	479	1	470
W1B355	3TA9:A	47.32	1070	458	13	450
A0A0K6I4N5	1OD0:A	47.21	1000	468	28	453
A0A0F7C1J0	1E4I:A	38.74	922	447	4	439
A0A0X2PCT3	4PTV:A	45.10	1046	452	6	444
A0A0H3V5F1	1OD0:A	47.44	1095	468	25	465
A0A143XQ59	1OD0:A	45.01	1045	468	28	465
Q9LAV5	1GNX:A	58.89	1373	479	12	474
A0A0U5NVN5	1OD0:A	43.53	949	468	28	464
A0A0A8VIP7	4B3K:A	48.37	1179	479	3	459
Q0R5R7	3TA9:A	57.24	1378	458	13	453
G9IS39	3GNO:A	47.00	1110	488	21	486
A0A157TCL9	5FOO:A	45.22	1058	480	4	460
A0A140JXI4	5DT5:A	43.12	981	471	21	463
A0A157TWG0	5FOO:A	45.48	928	480	4	409
A0A157TYJ0	3TA9:A	45.41	1053	458	13	450
A0A157TG10	3TA9:A	45.41	1046	458	13	450
A0A155TTI4	3TA9:A	38.04	786	458	16	452
L0EKA4	1OD0:A	50.00	1129	468	27	465
A0A078MRL3	3W53:A	65.96	1714	506	35	504
A0A0H3V883	1BGG:A	61.66	1542	448	1	445
M9ZC55	1VFF:A	37.62	694	423	5	408
Q0GMU3	4HZ6:A	98.87	2341	444	2	442
C5JB25	3TA9:A	44.54	986	458	8	455
A0A0F7KKB7	1OD0:A	51.80	1128	468	27	465
W6CG73	3AHX:A	48.87	1153	453	4	440

G9BYB8	1BGG:A	45.80	1059	448	1	440
A0A075IXW8	3WH5:A	46.55	971	457	2	441
K4I4U1	3WH5:A	56.14	1235	457	6	444
W0LHR5	1OD0:A	45.76	1017	468	28	465
D5KX75	2O9P:A	43.32	995	454	18	451
G3EGK5	3CMJ:A	50.34	1130	465	26	461
V5R1L0	3TA9:A	52.33	1222	458	9	452
D0VEC8	5DT5:A	43.99	982	471	23	463
C5JB26	3TA9:A	44.32	945	458	13	451
A0A075IXX4	1OD0:A	43.62	978	468	26	466
A0A0H3V8A5	1OD0:A	46.43	1052	468	27	465
D7RTU9	3WH5:A	44.60	1003	457	9	443
A0A075IXX0	4PTV:A	53.15	1246	452	1	444
G9IS31	3TA9:A	50.88	1187	458	8	455
Q7X3Y0	1GNX:A	51.81	1108	479	16	477
A0A0M0F8C3	1GNX:A	62.55	1495	479	7	478
K7PES2	3TA9:A	45.19	1099	458	8	450
Q9RA58	3ZJK:A	97.64	2137	431	1	423
J7IJ95	2O9P:A	99.55	2401	454	8	454
V9VGK4	3AHX:A	40.30	868	453	6	445
A0A0L8B0R6	3TA9:A	49.77	1158	458	13	450
Q8GEB0	3ZJK:A	98.61	2195	431	1	431
D3Y2V4	5DT5:A	54.83	1392	471	24	466
Q8GEB1	1NP2:A	97.70	2210	436	1	435
A0A0L1MTV9	5DT5:A	89.96	2239	471	24	471
A0A0L1MUX2	3AHX:A	40.48	956	453	4	440
A0A077XW08	1OD0:A	46.33	1047	468	21	462
A0A0D6GCL2	4B3K:A	45.10	1060	479	3	459
A0A0L1JVB2	1OD0:A	43.69	944	468	28	453
A0A076VZW1	2O9P:A	99.33	2396	454	8	454
S5U834	3TA9:A	53.26	1207	458	13	450
A0A0H5SI25	1OD0:A	47.58	1088	468	28	465
A0A0X8X3L2	1OD0:A	42.42	976	468	16	462
J7KFN3	3AHX:A	53.49	1244	453	4	444
F1JZ12	1OD0:A	49.66	1102	468	28	465
A0A0I9P479	3TA9:A	46.31	1054	458	13	450
A0A0U5BL20	3W53:A	69.05	1689	506	45	506
A0A0C7KXK1	1VFF:A	31.94	532	423	5	406
A0A0I9P996	3TA9:A	46.31	1049	458	13	450
A0A0C7KRG8	4PTV:A	46.31	1052	452	7	444
A0A0U5BSL5	1GNX:A	59.96	1365	479	14	477
A0A0Y4EJZ0	4PTV:A	41.20	890	452	7	445
Q53EH2	3AHX:A	100.00	2388	453	1	445
A0A0T8JGS2	4B3K:A	48.16	1160	479	1	459
A0A0Z5BZ97	5FOO:A	47.63	1149	480	1	460
A0A0T8XSW7	4B3K:A	49.82	732	479	183	459
A0A0U0LRY2	4B3K:A	47.95	1155	479	1	459
A0A0U0ASS4	1UYQ:A	33.05	590	447	5	441
A0A0U0FKQ8	3TA9:A	30.97	270	458	199	452
A0A0Y4F8K9	4B3K:A	49.57	1200	479	3	459
A0A0U0B443	4B3K:A	48.16	1160	479	1	459
A0A0T8XSY3	5FOO:A	46.29	421	480	1	171
A0A121JEF9	1UYQ:A	32.08	582	447	5	441
A0A0X9CC82	4B3K:A	47.73	1154	479	1	459
A0A110E8P6	4B3K:A	47.51	1177	479	2	459
A0A0S1URP8	1GNX:A	68.97	1631	479	18	479
A0A088ME52	1VFF:A	34.59	574	423	3	408

Q45FX7	2O9P:A	40.61	613	454	127	417
A0A0S1UWT1	3WH5:A	55.58	1209	457	6	434
A0A0S4PQL1	1OD0:A	57.63	1376	468	27	464
A0A0S1UK89	1GNX:A	76.20	1886	479	1	477
B3VE09	4B3K:A	51.52	1267	479	3	459
Q9X9R4	1GNX:A	72.73	888	479	14	244
A0A0H4NXH8	5DT5:A	55.38	1348	471	26	461
Q9L794	1NP2:A	100.00	2257	436	1	436
D0VLH9	5DT5:A	93.75	2308	471	24	471
A0A0K2E2E4	3TA9:A	32.43	555	458	13	451
Q8GKQ8	3TA9:A	56.26	1256	458	13	451
A0A0K3BGD4	3WH5:A	46.61	992	457	6	444
A0A0K3B3W1	1GNX:A	53.76	1201	479	8	472
A0A0K3BB21	1VFF:A	30.85	449	423	9	406
A0A0T9K4S5	1GNX:A	65.80	1494	479	16	473
A0A0T9K572	1GNX:A	58.25	1338	479	1	477
A0A0T9KSD8	3TA9:A	28.91	378	458	9	457
L0ELF5	1GNX:A	61.34	1398	479	14	473
A0A0T9MYM8	5AYI:A	54.09	1189	457	6	444
A0A0T9MR01	1GNX:A	49.70	1151	479	8	470
A0A0T9JWZ0	1NP2:A	53.36	1096	436	6	429
J9XU85	1GNX:A	53.70	1175	479	15	467
A0A0Y0M4F4	3W53:A	97.67	2425	506	35	506
G0M2J6	4PTV:A	31.62	514	452	10	452
B9V8P5	3W53:A	100.00	2506	506	35	506
A0A0Y0KCV2	3W53:A	97.88	2430	506	35	506
A0A154NB93	1OD0:A	46.52	1022	468	27	466
A0A143Z9N1	1OD0:A	47.15	1057	468	27	461
A0A0S4PIT6	1OD0:A	57.63	1371	468	27	464
A0A060ZTS4	1GNX:A	71.40	1725	479	16	477
Q59976	1GNX:A	99.37	2489	479	1	479
A0A0K6GS41	3TA9:A	52.55	1210	458	13	456
A0A060ZE79	3WH5:A	55.43	1232	457	6	446
I7CL63	1GNX:A	91.37	2245	479	5	479
E5B1F9	1QOX:A	36.93	721	449	5	443
A0A0M1JNQ3	3TA9:A	52.56	1199	458	13	455
C9YC48	3WH5:A	49.78	1055	457	6	446
A0A0U5NTI6	1VFF:A	29.64	496	423	4	402
Q8GEB2	1NP2:A	98.37	2196	436	1	430
A0A0U5JEL6	3TA9:A	49.33	1156	458	13	457
A0A0U5J538	3CMJ:A	45.27	1015	465	25	464
A0A0U5IDY0	3TA9:A	51.76	1234	458	1	454
G1C1T5	3CMJ:A	50.34	1087	465	26	458
A1C3J9	4B3K:A	48.15	1155	479	3	459
Q6QGY5	1UYQ:A	33.77	637	447	4	440
Q9ZNN7	1QOX:A	60.00	1472	449	4	443
E0X9H4	5DT5:A	83.26	2069	471	24	465
A4U5G3	3TA9:A	44.49	964	458	16	454
W0FQM2	1OD0:A	45.03	969	468	28	465
I7CPF4	1GNX:A	59.48	1347	479	18	476
A4U0J3	3WH5:A	46.55	958	457	4	445
A0A0A6ZH67	3TA9:A	47.25	1120	458	8	450
A0A140JXI1	5DT5:A	44.47	999	471	30	463
V5W6I4	3AHX:A	33.60	487	453	54	420
E3UMV6	1QOX:A	41.24	862	449	5	443
A0A023ZUM4	3TA9:A	32.21	282	458	197	450
V9XQW2	3AHX:A	32.35	476	453	55	440

A0A0A1E4J6	3TA9:A	35.12	235	458	235	430
A0A059U805	3TA9:A	33.44	324	458	165	449
A0A023ZT20	3TA9:A	32.35	351	458	128	450
I7DLX2	3AHX:A	53.71	1253	453	4	444
A0A0A1E9M1	3TA9:A	32.81	324	458	154	450
V9XR34	3AHX:A	34.68	579	453	5	421
V9XQ07	1OD0:A	30.99	356	468	118	442
V9XST0	3TA9:A	31.20	358	458	103	433
A0A023ZTM1	3TA9:A	33.33	364	458	139	452
A0A140JXI7	4PTV:A	44.31	995	452	6	427
A0A140JXI6	1NP2:A	47.37	980	436	2	428
A0A0S4QTN7	3W53:A	52.46	1173	506	46	503
F4N5B9	4B3K:A	42.64	1025	479	3	462
F4MXF8	4B3K:A	46.41	1113	479	3	459
F4MXN9	3TA9:A	33.97	617	458	8	450
I5CAT0	4PTV:A	46.59	1043	452	1	438
A0A0E3GPF8	3AHX:A	40.69	958	453	4	440
A0A0S4MXN0	1OD0:A	57.63	1376	468	27	464
A0R2K0	1GNX:A	56.62	1345	479	10	477
A0A150RWY2	1OD0:A	45.95	1044	468	19	465
A0A150NUT2	4B3K:A	47.51	1155	479	1	459
A0A150TYH0	1OD0:A	46.39	1051	468	19	465
A0A150S4Q9	3TA9:A	51.86	1239	458	8	456
A0A0Q0VJU7	1OD0:A	46.42	1005	468	26	453
A0A0D0N6M1	1QOX:A	34.68	607	449	5	449
A3KK08	3TA9:A	50.88	1174	458	13	450
A3KIN3	1GNX:A	90.19	2221	479	1	479
A0AC59	3WH5:A	55.45	1220	457	6	444
A0A0D0GGG1	1BGG:A	61.88	1603	448	1	445
A0A0D0G1V9	1BGG:A	61.43	1594	448	1	445
A0A0S4KE28	4PTV:A	49.43	1152	452	5	444
A0A0S4NTP3	3WH5:A	51.11	1135	457	1	446
A0A0S4P4S7	4PTV:A	47.19	1062	452	7	445
A0A0B5SBW4	3AHX:A	41.81	929	453	4	441
A0A0H0ZZM6	3W53:A	83.05	2118	506	35	506
A0A0L8LRR7	1NP2:A	49.22	923	436	6	430
A0A0L8LCW7	1GNX:A	66.17	1616	479	12	477
A0A0L8LXE6	1QOX:A	46.71	1063	449	5	443
A0A0M3QKY5	1GNX:A	79.20	1952	479	1	476
A0A0U1BIX4	1E4I:A	28.00	364	447	9	419
A0A0U1BBI9	1E4I:A	28.00	364	447	9	419
A0A139R3I5	4B3K:A	65.50	1715	479	2	459
F1LL52	1GNX:A	48.72	1112	479	16	475
B4V9A8	1GNX:A	61.83	1437	479	17	476
B4VCY4	3WH5:A	53.41	1164	457	6	444
A0A0P7YNG9	1OD0:A	44.03	938	468	28	453
I3QIG4	1UYQ:A	32.17	617	447	4	441
A0A0P7ZLQ1	3CMJ:A	45.75	998	465	24	456
A0A0G3S6W4	3TA9:A	47.11	1062	458	13	450
A0A0M8K9N5	1VFF:A	35.43	628	423	1	412
A0A0B7G574	4PTV:A	46.31	1050	452	7	444
Q091M8	1VFF:A	35.94	637	423	5	405
Q08YK7	1OD0:A	47.43	1027	468	27	466
A0A076JJU4	1GNX:A	53.51	1185	479	16	467
C2E3G3	1OD0:A	30.64	536	468	24	467
A0A0U0DGH1	1UYQ:A	33.05	594	447	5	441
A0A0I9FSI8	5FOO:A	47.63	1148	480	1	460

A0A0E8TAC1	1UYQ:A	33.05	595	447	5	441
A0A0U0BFN0	1UYQ:A	33.26	599	447	5	441
A0A0E8P693	4B3K:A	47.95	1156	479	1	459
A0A0T8F0P2	1UYQ:A	33.05	597	447	5	441
A0A0T8M6A5	3TA9:A	40.04	924	458	13	451
A0A0T8WZJ2	5FOO:A	47.84	1152	480	1	460
A0A0U0PAY4	4B3K:A	47.73	1151	479	1	459
A0A0U0MQ66	1UYQ:A	33.05	598	447	5	441
A0A0E8ZC59	1UYQ:A	33.05	592	447	5	441
A0A0U0FWW9	1UYQ:A	33.05	594	447	5	441
A0A0T8UQJ1	1UYQ:A	32.83	593	447	5	441
A0A098Z269	1UYQ:A	33.05	595	447	5	441
A0A0U0F4G5	4B3K:A	48.16	1159	479	1	459
A0A0B7KU25	4B3K:A	48.16	1158	479	1	459
A0A110RL77	1UYQ:A	33.05	599	447	5	441
A0A0T8II41	4B3K:A	47.95	1155	479	1	459
A0A0T8JTT4	1UYQ:A	33.05	595	447	5	441
A0A0K9HVW3	3TA9:A	41.10	888	458	8	451
A0A0T8N2M5	3TA9:A	41.67	119	458	385	451
A0A0B7LKH4	4B3K:A	48.16	1159	479	1	459
A0A0I9LTB1	5FOO:A	47.84	1155	480	1	460
A0A0B7L5X3	4B3K:A	48.16	1161	479	1	459
A0A0I9GY50	1UYQ:A	33.05	595	447	5	441
A0A0B7LW39	4B3K:A	47.95	1156	479	1	459
A0A0U0FVD0	1UYQ:A	33.05	596	447	5	441
A0A0T9IAX2	4B3K:A	47.95	1156	479	1	459
A0A064C5A1	1UYQ:A	33.05	594	447	5	441
A0A098Z798	4B3K:A	47.95	1158	479	1	459
A0A064BWP9	4B3K:A	47.95	1155	479	1	459
A0A0U0BIG0	1UYQ:A	33.05	593	447	5	441
A0A0T7JLE7	4B3K:A	48.16	1164	479	1	459
A0A0T7JYV2	1UYQ:A	33.05	595	447	5	441
A0A0T8XAA3	1UYQ:A	33.05	592	447	5	441
A0A0T7YD17	4B3K:A	47.95	1155	479	1	459
A0A0D6J947	1UYQ:A	33.05	593	447	5	441
A0A0E7TKP3	1UYQ:A	33.05	598	447	5	441
A0A0T7YEA9	1UYQ:A	32.27	576	447	5	441
G4XU74	1OD0:A	99.77	2353	468	25	468
A8R876	1UYQ:A	31.76	553	447	4	441
K8A4I0	1UYQ:A	34.09	637	447	5	441
A0A139ME62	4PTV:A	40.81	870	452	7	446
T1VQ26	3ZJK:A	49.23	1030	431	3	431
K6PJ73	4B3K:A	56.58	1436	479	1	456
A0A0T9UY74	1UYQ:A	29.58	555	447	5	441
K4QAX1	4B3K:A	97.83	2437	479	1	461
K6QWU0	4B3K:A	45.43	1106	479	3	459
K6RDR9	4B3K:A	56.89	1439	479	1	457
A0A142LEP2	4PTV:A	44.35	1050	452	7	446
A0A0H5MA25	1UYQ:A	29.79	570	447	5	441
K6R4L1	1UYQ:A	33.69	648	447	4	436
A0A139MHM4	4B3K:A	50.00	137	479	163	225
A0A0W8EKX1	1OD0:A	42.11	985	468	16	465
A0A0M3BGC8	1E4I:A	45.00	1035	447	4	439
K7ZX81	4B3K:A	40.51	962	479	1	470
A0A139MFY5	5FOO:A	46.65	1122	480	1	460
A0A139P5P8	4B3K:A	46.10	1120	479	1	459
A0A0A0C667	3W53:A	52.37	1232	506	41	499

A0A0P7DRH9	3TA9:A	43.47	1000	458	9	452
F2JX03	3CMJ:A	48.25	1066	465	30	458
K8A1Z8	4B3K:A	46.85	1137	479	2	459
A0A139PC59	1UYQ:A	33.82	602	447	4	441
A0A0A0C5P9	3W53:A	54.23	1285	506	46	500
A0A0X3U6F4	1OD0:A	45.45	984	468	21	453
A0A0D0LT36	1OD0:A	43.67	995	468	20	457
A0A0A0C9G0	1GNX:A	61.78	1419	479	10	477
A0A139MFT5	4B3K:A	55.36	1435	479	1	456
A0A0M8JET1	1OD0:A	49.32	1096	468	28	465
A0A022MI49	1NP2:A	51.02	925	436	6	423
A0A0D0X8K6	1GNX:A	60.57	1323	479	15	472
A0A0D0WQW4	4PTV:A	51.47	1169	452	7	445
A0A149PEU0	3TA9:A	47.95	1092	458	12	444
F0GXD3	3AHX:A	34.34	536	453	6	441
A0A0D0UVT1	1GNX:A	58.71	1301	479	16	475
A0A0D0WQU9	1GNX:A	66.38	1523	479	17	476
A0A022M6R2	1GNX:A	68.25	1645	479	16	477
A0A149PG68	3TA9:A	50.11	1163	458	8	452
A0A022MK77	3WH5:A	55.68	1228	457	6	444
A0A0N7I4H8	3W53:A	52.98	1242	506	44	501
A0A0Q5WSI4	1OD0:A	46.14	1044	468	27	462
M5DXZ5	3TA9:A	62.53	1548	458	8	457
B6AXQ7	1OD0:A	44.29	974	468	26	462
H8FXJ8	3TA9:A	45.50	1003	458	13	455
D5ULE7	1GNX:A	60.42	1447	479	7	478
K6RUM4	4B3K:A	57.02	1428	479	1	456
S4XL86	4PTV:A	51.86	1240	452	2	450
A0A098BMM1	1VFF:A	29.27	369	423	7	399
A3UQH3	2O9R:A	46.06	1086	452	16	447
F6FR56	1GNX:A	62.24	1482	479	2	477
A0A154QHN7	1OD0:A	49.77	1124	468	27	465
F6FS57	3W53:A	56.93	1380	506	42	502
S4XJZ7	1OD0:A	46.39	1051	468	19	465
A6LNI1	1OD0:A	53.90	1191	468	28	463
R4PXL6	1VFF:A	33.33	600	423	2	405
A0A087AN97	1GNX:A	52.60	1165	479	15	472
S4XIQ3	1OD0:A	45.80	1029	468	23	465
K6RPI9	1UYQ:A	33.76	657	447	4	441
I2QQE8	3WH5:A	44.14	909	457	2	441
A0A0J8AUG9	3TA9:A	50.88	1164	458	13	450
A0A0F2KN96	1OD0:A	47.86	1078	468	28	466
A0A085HF97	3TA9:A	45.19	1035	458	13	450
G2PWX8	3AHX:A	52.84	1249	453	4	444
A0A0F2KL03	3TA9:A	42.54	927	458	10	451
A0A0J8AQ77	1GNX:A	68.03	1634	479	16	477
A0A085I917	4B3K:A	40.00	933	479	1	462
A0A0A3F356	2O9P:A	46.06	1048	454	18	449
A0A0F2KQJ2	3W53:A	45.67	972	506	40	503
A0A0J8A072	1NP2:A	49.66	893	436	12	430
A0A0V1R2M8	1OD0:A	41.08	928	468	24	461
U1HA43	3WH5:A	46.98	927	457	3	441
A0A0J8AQ73	1GNX:A	69.31	1718	479	1	477
X8HBB2	1UYQ:A	33.54	595	447	4	441
A0A0L6K173	1OD0:A	44.80	1038	468	25	462
A0A0F2KSV2	3TA9:A	42.12	944	458	13	456
A0A0J8CZL6	3TA9:A	46.98	1164	458	13	455

A0A085HKV0	4B3K:A	45.22	1056	479	3	459
A0A0J8A2L0	3WH5:A	56.79	1242	457	6	446
A0A085H3F7	4B3K:A	45.77	1076	479	3	459
A0A117KEY2	1OD0:A	43.05	993	468	20	461
F1TE14	1OD0:A	42.09	936	468	28	465
A0A0E0YIZ3	2O9P:A	42.86	999	454	9	454
A0A0T9QSJ4	1UYQ:A	29.38	569	447	5	441
D7AS23	5DT5:A	55.91	1405	471	24	461
A0A0E0YLJ1	3TA9:A	44.70	982	458	17	450
C5ENY1	1OD0:A	33.61	601	468	27	465
A0A011T636	3W53:A	55.86	1315	506	36	501
B3Q9I7	3TA9:A	43.54	958	458	15	454
A0A0T6LRD4	1GNX:A	67.60	1570	479	16	477
A0A154UZW3	1GNX:A	59.59	1393	479	3	479
A0A0T6LXE1	3TA9:A	47.43	1133	458	13	455
C5EP49	1UYQ:A	33.19	571	447	7	441
A0A0D0JB19	2O9P:A	45.37	1046	454	18	449
E4WGS6	3TA9:A	45.05	1010	458	13	450
A0A0Q7V023	1QOX:A	43.98	922	449	3	447
D4IYQ1	1VFF:A	31.80	533	423	5	402
R8KT44	4PTV:A	40.91	940	452	7	445
A0A0Q5W511	1GNX:A	55.36	1196	479	16	472
A0A0T6LP89	1GNX:A	75.65	1802	479	18	477
A0A0R2XD05	1OD0:A	43.43	971	468	26	465
A0A0G4AZ53	1VFF:A	33.10	580	423	5	396
R7DU43	1OD0:A	45.43	951	468	28	464
A0A0A5I272	3TA9:A	46.68	1072	458	15	450
W4N6E1	1GNX:A	52.95	1147	479	17	469
A0A075P142	5DT5:A	45.02	995	471	23	460
G2RNI7	3AHX:A	40.69	988	453	4	440
C5CHI5	1OD0:A	46.73	1010	468	24	461
W1H6G4	4B3K:A	41.19	842	479	31	415
A0A146GCV4	1OD0:A	44.49	974	468	23	464
A0A0P6TWT0	3WH5:A	56.25	1260	457	6	449
A0A0P6U1D2	1GNX:A	68.10	1609	479	14	476
A0A146GB87	1OD0:A	43.05	990	468	21	465
A0A0N1DIY9	1QOX:A	47.96	1054	449	5	444
M4KVI0	4B3K:A	49.78	1225	479	3	459
Q6CYW8	1UYQ:A	30.79	554	447	1	441
A0A146G4C1	1OD0:A	45.31	1032	468	27	465
M4KUV0	1QOX:A	41.24	860	449	5	443
W1GLR7	3TA9:A	47.32	1070	458	13	450
A0A0E3UIK8	1OD0:A	42.04	923	468	21	466
A0A0Q0UUL0	3W53:A	45.65	988	506	45	500
G7D1R8	3CMJ:A	44.49	919	465	26	463
A0A0P6UAH6	1GNX:A	68.54	1666	479	5	477
R9G8Q2	1QOX:A	41.58	871	449	5	442
A0A101AMM3	4HZ6:A	40.78	870	444	3	442
A0A0Q6VIU9	3TA9:A	47.62	1102	458	13	452
A0A0Q9UMU3	3AHX:A	41.59	925	453	4	441
A0A146G1V6	1QOX:A	46.07	1014	449	5	443
A0A146GBB3	1OD0:A	41.74	951	468	28	467
A0A0P6U989	1GNX:A	56.99	1181	479	18	472
A0A0F3I349	4B3K:A	46.41	1094	479	3	459
G7DLD6	3WH5:A	45.23	910	457	6	441
M4KYJ7	1UYQ:A	32.17	618	447	4	441
A0A0P6UGD3	3CMJ:A	48.65	1076	465	21	458

A0A0P6TUV8	3ZJK:A	48.43	910	431	8	430
I2SQZ8	1QOX:A	34.68	605	449	5	449
A0A146G7S6	1OD0:A	43.57	956	468	25	465
A0A075P3W9	3WH5:A	46.49	987	457	9	447
A0A146GB03	1OD0:A	42.67	974	468	28	467
W1GZC2	4B3K:A	45.04	1067	479	3	463
A0A0F3HTY9	4B3K:A	48.16	1154	479	1	459
R9GHS9	4B3K:A	43.72	1001	479	1	459
W1G240	4B3K:A	47.49	1117	479	3	459
A0A0N0T4C3	3TA9:A	46.59	1070	458	12	456
A0A0Q7MCA6	1GNX:A	59.70	1400	479	18	478
A6AMQ2	2O9P:A	46.06	1046	454	18	449
A0A0H3FW13	3TA9:A	45.64	1057	458	13	450
A0A0Q6HVV2	3TA9:A	42.21	942	458	13	451
A0A0U4B2Z9	1OD0:A	42.38	963	468	25	464
E4S6B1	3AHX:A	52.84	1249	453	4	444
K7VST0	4B3K:A	58.56	1523	479	1	471
S3AKQ3	1GNX:A	52.99	1248	479	16	477
A0A0U4C9Z2	3WH5:A	54.98	1223	457	5	441
A0A0S9RAU0	1QOX:A	45.38	969	449	5	443
C9QH22	2O9P:A	45.14	1042	454	18	449
I5BYR6	1OD0:A	46.28	988	468	28	453
R5EQ88	1QOX:A	44.32	920	449	5	443
A0A085CIX4	1OD0:A	44.39	1038	468	21	462
L8XKA1	2O9R:A	45.37	1041	452	16	447
A0A0S2JHQ8	2O9P:A	44.24	1020	454	18	451
A0A0M8VB47	3W53:A	47.61	963	506	45	503
A0A0M8WEL4	1GNX:A	66.09	1595	479	16	479
H9ZUT0	1NP2:A	98.16	2215	436	1	435
A0A0H0XXR0	4PTV:A	46.56	1041	452	10	444
A0A0S9R7Q3	3W53:A	54.33	1273	506	44	500
A0A0Q7MQI4	3TA9:A	46.58	1028	458	11	455
A0A0A2TZZ2	1BGG:A	75.17	1940	448	1	447
G8TT01	3TA9:A	55.43	1323	458	13	452
A0A0Q6WRY4	1GNX:A	63.81	1450	479	16	477
A0A0L8PA08	1GNX:A	49.79	971	479	11	472
Q1GUD6	1OD0:A	48.75	1101	468	28	465
F6BL86	5DT5:A	55.25	1361	471	26	463
A0A0A2MG18	1OD0:A	47.30	1031	468	24	462
A0A0Q6YIM5	1GNX:A	66.38	1623	479	12	477
A0A0Q6YR25	3WH5:A	55.93	1245	457	6	449
C5WI66	4B3K:A	97.40	2435	479	1	461
A0A0L8PBL5	1GNX:A	69.43	1669	479	16	477
Q11NH0	1OD0:A	38.67	881	468	18	462
A0A0Q6X661	1NP2:A	50.90	963	436	8	430
G8TSK9	1VFF:A	39.22	699	423	9	409
A0A139NTA1	4B3K:A	47.94	1135	479	1	459
U2A5Y3	1GNX:A	61.98	1488	479	2	479
A0A0M2VK60	3TA9:A	43.85	996	458	12	454
A0A0N0GVB2	1NP2:A	50.45	978	436	7	426
D1AWL3	5FOO:A	48.48	1200	480	1	460
A0A0F4NXS1	3CMJ:A	46.42	1016	465	30	458
F0YZT2	3CMJ:A	41.89	937	465	25	459
A0A0F4NX57	3CMJ:A	46.19	1015	465	30	458
B5IQL4	1VFF:A	35.07	589	423	5	390
F0Z422	1OD0:A	43.65	984	468	28	465
A0A0F0KTN0	1VFF:A	34.23	593	423	3	404

A0A0V2F7J8	1GNX:A	43.78	886	479	18	479
H5YQE7	3WH5:A	44.77	909	457	6	441
A0A0T6ZU64	1QOX:A	47.37	1022	449	5	439
A0A0Q8GU78	1GNX:A	45.92	919	479	18	479
A0A0K2SK68	3TA9:A	56.22	1320	458	8	455
X0R5P1	1UYQ:A	60.90	1543	447	1	444
A0A0F0KH49	3W53:A	52.77	1230	506	44	501
A0A150Z5A9	3CMJ:A	43.27	919	465	16	461
A0A101V084	1GNX:A	66.09	1624	479	12	477
A0A084JPX9	3AHX:A	49.33	1165	453	1	443
A0A101V4H0	1NP2:A	50.34	917	436	8	430
A0A089HNV9	1QOX:A	62.59	1545	449	5	445
E2PVV7	3WH5:A	47.00	933	457	6	443
R7BJP0	1OD0:A	45.90	1031	468	28	465
A0A117KA82	1GNX:A	51.85	1080	479	18	472
A0A0X7JPM1	1GNX:A	50.21	990	479	10	472
A0A0Q6I670	3AHX:A	43.10	968	453	4	441
A0A0N0SLS3	1GNX:A	64.79	1513	479	14	476
A0A0H3GTM8	3TA9:A	47.32	1070	458	13	450
A0A0Q8CF54	1GNX:A	59.49	1407	479	13	477
A0A0N0KBI6	1GNX:A	48.39	1055	479	16	479
A0A0X7JGE3	1GNX:A	69.85	1674	479	16	477
A0A084JRD9	1OD0:A	44.30	951	468	28	462
A0A0X3SV59	1GNX:A	69.91	1668	479	17	477
U3TK74	4B3K:A	98.05	2448	479	1	461
A0A0M8SSG9	3WH5:A	55.91	1237	457	6	444
A0A0A2TFJ7	1BGG:A	57.62	1437	448	1	445
B8HA17	3W53:A	56.12	1356	506	40	502
W1I0E0	3TA9:A	47.10	1061	458	13	450
A0A0M9VKZ6	1GNX:A	62.82	1455	479	13	477
R5DYS0	1OD0:A	41.50	895	468	28	462
A0A154L8Z0	1OD0:A	46.41	1059	468	25	466
A0A0A2TVI0	5DT5:A	65.68	1630	471	26	464
K6WUZ5	3TA9:A	46.14	1027	458	16	455
A0A0Q9QFX1	3TA9:A	49.20	1090	458	13	450
W1HPW0	4B3K:A	45.04	1067	479	3	463
W8EZI0	1OD0:A	42.04	963	468	16	461
W1I155	4B3K:A	40.30	966	479	1	470
B8HD08	3W53:A	47.33	970	506	46	506
A0A0A0J8T6	1GNX:A	52.64	1154	479	18	470
A0A154KSN9	1OD0:A	45.96	1048	468	25	466
W6WWY6	4PTV:A	46.59	1044	452	1	438
A0A0M2S5L5	1GNX:A	53.26	1083	479	18	472
S4MGR2	1NP2:A	51.56	961	436	8	434
E6U3G5	5DT5:A	58.77	1403	471	29	466
E1LMW8	4B3K:A	47.95	1160	479	1	459
S7XIH6	1GNX:A	62.02	1409	479	13	477
A0A117QJQ4	1GNX:A	67.82	1623	479	16	477
A0A066UD62	3WH5:A	53.85	1191	457	6	446
S4N9R4	1GNX:A	68.47	1662	479	16	477
A0A0N1AFJ4	3WH5:A	51.24	1098	457	6	444
A0A0M2RXG8	1NP2:A	55.10	1088	436	1	429
A0A101QKT1	3WH5:A	56.33	1243	457	6	446
T0TBU3	4B3K:A	65.12	322	479	2	87
G7G5P6	5DT5:A	42.32	956	471	15	462
X0N079	1NP2:A	52.60	1010	436	8	428
A0A101QJH3	3TA9:A	50.67	1170	458	13	450

S4MYQ7	1GNX:A	54.60	1149	479	18	470
E6K1F8	1GNX:A	50.96	1166	479	16	475
A0A0C1ZBY6	3TA9:A	48.44	1182	458	13	455
A0A0M9XVN1	1GNX:A	49.46	983	479	10	472
D6EU92	1GNX:A	89.56	2206	479	1	479
A0A0A6S8P4	2O9P:A	42.53	994	454	10	451
A0A0N0S9E3	1GNX:A	69.64	1667	479	16	477
A0A066TWD6	1GNX:A	54.60	1185	479	9	472
D6EXV5	1GNX:A	65.95	1619	479	12	477
E1LJV9	3CMJ:A	45.16	184	465	377	462
A0A0N0J8Y0	3TA9:A	48.43	1149	458	13	451
A0A0N1AEE0	3TA9:A	48.62	1089	458	17	450
A0A0C1Z6J0	2O9P:A	45.14	1038	454	18	449
G7G6E9	3TA9:A	42.92	988	458	12	452
A0A076M6B6	1NP2:A	52.02	935	436	6	426
A0A066TTF6	1NP2:A	52.09	1000	436	7	426
H9UL14	1GNX:A	51.85	1115	479	16	470
X5K473	1OD0:A	43.27	1017	468	21	462
A0A117V914	1OD0:A	43.50	1023	468	21	462
A0A124HPG9	1GNX:A	69.10	1715	479	1	477
I9DRN0	3TA9:A	45.25	985	458	8	442
A0A124HPM6	1NP2:A	49.66	894	436	12	430
G9A0Q1	1QOX:A	47.37	1021	449	5	439
A0A0Q5H8U8	3W53:A	47.64	975	506	46	503
A0A066U9N2	1GNX:A	52.37	1093	479	16	477
T0TF90	1OD0:A	39.62	815	468	21	465
D6ETZ7	1GNX:A	57.64	1165	479	18	470
T0ULY1	4B3K:A	65.90	1304	479	114	459
A0A0M2RXU4	3TA9:A	50.68	1179	458	11	452
A0A076LVL8	3WH5:A	55.20	1215	457	6	446
A0A066U508	3W53:A	53.70	1228	506	45	502
A0A0H3HA15	3TA9:A	46.65	1055	458	13	450
A0A066TXP2	1GNX:A	50.88	1038	479	17	467
A0A0D4DUA9	1NP2:A	50.34	954	436	12	426
A0A0M2S7U2	1GNX:A	61.93	1342	479	16	472
A0A0M2RR28	1GNX:A	57.46	1240	479	17	472
L7FBS5	1NP2:A	50.22	937	436	8	430
L0A0G1	4PTV:A	51.00	1222	452	1	449
L7EYW0	1GNX:A	53.29	1168	479	16	478
A0A0F2SQ31	3CMJ:A	47.07	1021	465	26	459
A0A0Q5TFA5	3CMJ:A	42.24	940	465	26	458
A0A101SG54	1GNX:A	66.38	1624	479	12	477
A0A0S7BW82	3TA9:A	52.80	1226	458	8	450
A0A100Y7K5	1GNX:A	68.99	1653	479	5	477
A0A100Y2V7	3WH5:A	54.09	1209	457	6	444
Q2WAV2	4PTV:A	46.85	1036	452	7	449
Q8DRA9	4B3K:A	47.95	1156	479	1	459
A0A101SET1	1NP2:A	50.67	954	436	8	430
L7FHS6	1GNX:A	65.01	1588	479	16	477
A1S5F3	3TA9:A	45.39	1010	458	10	454
L7FDA7	1GNX:A	53.42	1179	479	16	477
K6QPI5	1UYQ:A	33.76	663	447	4	441
A6GCT8	3WH5:A	52.85	1189	457	1	441
A0A0J1EVE4	3W53:A	58.12	1395	506	40	502
L7F5W9	3TA9:A	47.88	1094	458	12	450
A0A124HVR2	1GNX:A	63.60	1441	479	16	477
A0A117IUM8	1GNX:A	73.32	1717	479	18	477

Q8DNA9	4B3K:A	53.26	1315	479	1	459
K6QJK5	4B3K:A	56.80	1439	479	1	456
A0A023NXL5	3TA9:A	48.64	1113	458	13	451
A0A0F4QMM0	3TA9:A	47.62	1083	458	12	450
A0A0S8EKQ4	1QOX:A	46.89	1011	449	5	443
A0A100Y3R7	3TA9:A	49.34	1118	458	13	450
A0A101XZP8	1UYQ:A	61.88	1549	447	1	445
L7F4B6	1GNX:A	52.88	1175	479	16	477
A0A100YA05	3WH5:A	54.55	1202	457	6	444
G8P4F6	3AHX:A	41.53	873	453	6	445
R7D3D1	1OD0:A	44.30	1019	468	21	465
A0A0P0NA10	1UYQ:A	34.39	610	447	4	441
J9HP47	2JIE:A	51.93	1310	454	14	454
A0A0M2HAV7	1VFF:A	33.73	588	423	4	406
A0A0Q9M540	1GNX:A	55.36	1157	479	15	470
A0A0P0N9C0	4B3K:A	48.71	1204	479	1	459
A0A0M5IM31	3WH5:A	56.66	1236	457	6	445
A0A0Q9EMW1	1OD0:A	51.33	1170	468	23	465
E6RMI2	4PTV:A	45.37	1022	452	6	448
A0A0M4DCS3	3ZJK:A	48.76	928	431	8	427
D9XHQ6	1GNX:A	53.88	1135	479	18	470
A0A0M2HHG6	3W53:A	53.94	1253	506	45	501
G8P915	4B3K:A	55.19	1435	479	1	461
G8P4C7	4B3K:A	58.35	1517	479	1	471
U1J6A7	3TA9:A	43.17	998	458	8	450
C2G3Q4	1OD0:A	45.07	1039	468	21	462
D9XB25	1GNX:A	68.74	1670	479	12	477
D9X810	1NP2:A	52.13	940	436	8	430
A0A0M4D7X9	1GNX:A	57.05	1296	479	10	477
A0A0M4DNG6	1GNX:A	67.95	1597	479	12	476
A0A0P9GJ12	3TA9:A	44.22	1021	458	12	452
A0A0F2C9B3	1VFF:A	34.96	605	423	3	404
C9ZEJ6	1GNX:A	91.23	2228	479	1	479
A0A0A7I8R1	1GNX:A	52.39	1164	479	16	467
A0A0F2C518	1GNX:A	54.35	1225	479	16	468
A0A0Q5PPH5	1OD0:A	42.25	919	468	15	462
A0A0F2CCH8	1GNX:A	61.34	1414	479	14	473
C9ZAQ5	1NP2:A	50.00	943	436	8	431
C9Z448	1GNX:A	67.02	1646	479	12	477
A0A0F2C8E2	3W53:A	54.16	1256	506	45	501
R5TWR6	3AHX:A	49.21	1145	453	5	440
R5TRK9	1OD0:A	47.43	1022	468	28	465
R5SYI5	1OD0:A	45.07	1031	468	32	465
A0A0Q9PEY1	3W53:A	47.53	956	506	46	502
C9ZB09	1GNX:A	52.88	1175	479	16	477
F2NA43	3CMJ:A	27.80	417	465	26	452
A0A0Q5MHL7	3TA9:A	42.43	949	458	13	451
A0A0W1L3T4	3TA9:A	41.91	950	458	12	450
A0A0M9XK83	1GNX:A	50.00	984	479	11	472
A0A0W1L3I4	2O9P:A	43.78	996	454	18	451
F2N7E4	3AHX:A	37.26	792	453	10	447
A0A0Q6SYF4	4PTV:A	48.03	1119	452	1	446
A0A0Q6TNJ5	3WH5:A	51.83	1172	457	6	440
A0A0T6YD22	1QOX:A	46.83	1028	449	5	444
A0A0Q6TLJ1	3WH5:A	45.68	1010	457	4	441
F0MA10	3W53:A	48.16	959	506	46	498
A0A0C2UQI7	2O9P:A	53.06	1345	454	14	454

A0A0Q6T1D3	3TA9:A	48.42	1121	458	13	451
A0A0U2PBX4	1QOX:A	61.02	1573	449	1	449
A0A0D0K764	1GNX:A	62.39	1415	479	14	470
A0A0Q6U8J4	1QOX:A	45.02	963	449	5	442
A0A0R2QH41	3TA9:A	45.83	1047	458	13	456
F0M5E8	3W53:A	57.11	1376	506	40	502
A0A0Q6NPV4	4PTV:A	46.51	1030	452	1	430
A0A0Q6TZA9	1E4I:A	42.63	949	447	5	439
T5KMS4	3W53:A	55.20	1258	506	44	501
A4YNI1	3TA9:A	44.04	973	458	16	450
M9R842	1OD0:A	43.36	945	468	28	453
A0A127N547	3TA9:A	44.13	1020	458	13	452
A0A0P9GL28	2O9P:A	51.82	1307	454	14	453
A0A087DJE3	1VFF:A	33.98	574	423	4	405
A0A0Q6TBK9	1QOX:A	44.14	937	449	5	442
A0A0D0JER1	3W53:A	55.63	1291	506	44	501
K5USK6	2O9P:A	45.60	1047	454	18	449
C4ILL3	1QOX:A	38.92	769	449	5	443
A0A0Q6T930	1OD0:A	46.07	1023	468	26	465
A4YUN3	1OD0:A	42.86	938	468	26	467
E3E9Z4	2O9P:A	94.41	2308	454	8	454
E3EAC3	1BGG:A	98.66	2396	448	1	448
A0A0Q6P061	3TA9:A	44.64	1010	458	8	455
A0A0P9CFM9	3TA9:A	52.48	1245	458	13	455
F8F615	1BGG:A	59.73	1488	448	1	446
K7AIP5	3TA9:A	44.92	997	458	16	455
A0A081C5A0	1OD0:A	45.05	1029	468	27	465
A0A0Q4PLV2	1OD0:A	46.82	1041	468	26	464
A0A0S9KM05	1GNX:A	60.98	1436	479	13	477
Q9CJ31	3AHX:A	41.88	879	453	6	441
D6U0N6	4PTV:A	51.00	1207	452	1	446
U2H7J0	1OD0:A	45.43	1005	468	21	462
D6U006	3TA9:A	48.87	1176	458	12	451
T2S6V3	1NP2:A	50.35	959	436	6	430
A0A0Q9Q6L3	3W53:A	47.72	997	506	46	502
I3VS74	5DT5:A	55.38	1348	471	26	461
A0A0Q9Q766	3W53:A	55.93	1305	506	39	496
A8AZ19	5FOO:A	47.84	1154	480	1	460
A0A0A8X2J6	1QOX:A	63.03	1561	449	1	449
A0A0M8XWG1	3W53:A	50.00	1104	506	45	503
K8ADK6	3CMJ:A	32.08	253	465	242	464
A0A0Q6VEW7	1GNX:A	50.11	1022	479	18	472
A0A0Q3X082	2O9R:A	54.65	1340	452	11	451
K8ALA8	4B3K:A	47.73	656	479	197	459
V6F7V4	3WH5:A	46.55	958	457	4	445
Q9CFL0	3AHX:A	34.10	598	453	4	440
V6F0S1	3TA9:A	44.72	969	458	16	454
F8F6C7	2O9R:A	51.98	1107	452	74	452
A0A0A8X5Z5	5DT5:A	71.75	1759	471	25	462
A0A0D4C3S4	3CMJ:A	43.31	900	465	26	459
A0A0Q6VX85	3W53:A	56.93	1326	506	45	496
A0A0G2ZHK7	3TA9:A	50.00	1207	458	13	456
T2RZY0	1GNX:A	51.08	1104	479	15	477
D6TBS0	3TA9:A	49.43	1147	458	13	450
A0A132N7E2	1OD0:A	47.71	1108	468	22	461
A0A0N0AWW8	1GNX:A	58.49	1277	479	7	477
A0A0M8YEC2	1GNX:A	54.39	1213	479	18	472

I3VXG7	5DT5:A	52.25	1292	471	24	463
Q9CFI7	4B3K:A	55.19	1427	479	1	461
H5T5C4	4B3K:A	45.45	1106	479	3	459
D4YI50	1BGA:A	33.45	383	447	54	332
A0A0M4E5M2	1GNX:A	68.83	1617	479	16	475
A0A101NCB0	1GNX:A	66.45	1621	479	11	477
V4P7X7	3TA9:A	50.00	1148	458	13	450
V4PF31	3TA9:A	47.89	1089	458	13	455
A0A101N010	1GNX:A	78.50	1958	479	1	477
A0A0M4DS89	1GNX:A	69.29	1691	479	2	479
A0A0M4DVV9	3WH5:A	45.97	913	457	1	444
A0A085UBR0	4B3K:A	48.37	1179	479	3	459
A0A117PTH4	1NP2:A	51.69	948	436	8	430
A0A0Q8WWW6	4HZ6:A	47.30	1033	444	2	440
A0A0X8SJP0	3TA9:A	44.88	1038	458	12	450
D3R7U0	1GNX:A	53.81	1176	479	16	467
G4RAB5	1OD0:A	49.65	1069	468	28	453
A0A117V8E6	1OD0:A	44.17	1050	468	21	462
A0A0L0KU26	1GNX:A	67.24	1656	479	12	477
W5VYM2	1NP2:A	51.58	974	436	1	426
G5JTB7	3GNO:A	31.35	534	488	17	488
A0A0M3QLZ5	3W53:A	51.52	1171	506	46	503
A0A0Q7SSI2	1OD0:A	46.62	1051	468	26	465
A0A117PRT8	1GNX:A	64.10	1450	479	16	477
A0A0M3QL38	3WH5:A	57.14	1261	457	6	449
A0A0C1KVY7	1OD0:A	39.78	890	468	21	462
H5T6U3	1QOX:A	33.77	660	449	5	445
A0A0P7AJZ9	4PTV:A	42.18	929	452	7	445
A0A0J1GLR9	3TA9:A	56.92	1340	458	13	452
A0A0S1YQV9	3TA9:A	44.14	994	458	15	458
A0A0L0KWS1	1GNX:A	53.09	1179	479	16	477
A0A127VGJ3	1UYQ:A	40.82	875	447	5	440
A0A0Q7SUU0	3TA9:A	49.77	1154	458	13	451
A0A0Q7S151	4PTV:A	46.96	1100	452	1	446
A0A0L0L3Z8	1NP2:A	50.45	956	436	7	432
W5W1A4	1GNX:A	54.49	1200	479	17	472
A0A0Q7RVS2	1E4I:A	42.27	934	447	5	438
A0A0B8T902	1OD0:A	44.74	1047	468	21	463
A0A101N9T5	3WH5:A	55.70	1255	457	6	449
W5WHJ9	1GNX:A	57.17	1299	479	15	478
A4BJP2	4HZ6:A	48.17	1011	444	1	437
A0A0Q8XG34	3W53:A	53.91	1260	506	43	503
W5WEG0	1VFF:A	29.20	386	423	2	419
A0A0Q7T1L9	3TA9:A	48.16	1150	458	13	446
A0A031HLF2	1E4I:A	44.97	960	447	3	441
A0A0T6UQT1	3WH5:A	45.52	922	457	6	437
W2F271	3TA9:A	49.33	1129	458	8	455
M7N622	3W53:A	77.47	1938	506	41	506
A0A0R2QPV0	3TA9:A	45.83	1047	458	13	456
K5CYK6	1OD0:A	45.02	1041	468	25	462
W2F0A8	1GNX:A	54.25	1285	479	18	476
A8S1H9	4PTV:A	47.76	1130	452	7	449
C7QZW4	3W53:A	51.78	1218	506	41	506
A0A086E1I4	3W53:A	56.49	1354	506	35	505
A0A084YXZ2	4PTV:A	45.32	1049	452	6	444
A0A086DXW8	3W53:A	68.27	1670	506	45	501
A0A0M8QR72	3WH5:A	56.56	1211	457	11	449

M7MR34	3W53:A	56.72	1298	506	40	505
C1C5U9	1UYQ:A	33.05	596	447	5	441
A6CVW9	1OD0:A	45.76	1015	468	28	465
W2EFT1	1GNX:A	53.74	1136	479	18	466
A0A0F5LUQ4	4PTV:A	48.61	1066	452	1	430
R0FFY1	1OD0:A	44.29	994	468	26	453
A0A0M8QG56	1GNX:A	47.17	895	479	18	472
U4QC20	1OD0:A	45.02	1037	468	25	462
G8QYI0	1BGG:A	44.67	1021	448	1	440
C7QZ99	1GNX:A	59.96	1386	479	18	477
A0A0M8QK34	1GNX:A	57.99	1175	479	18	467
V7EGD6	1OD0:A	44.44	967	468	28	464
W2EJ94	1NP2:A	56.94	1109	436	8	427
W2F073	3WH5:A	53.32	1162	457	9	444
W2ESQ6	1GNX:A	53.52	1183	479	17	475
W2EKL3	1GNX:A	53.25	1118	479	17	477
Q0FHD6	1OD0:A	47.02	1060	468	21	453
Q9L2L6	1NP2:A	51.79	931	436	6	426
F3Y9S9	4B3K:A	45.45	1102	479	3	459
A0A061MV28	1QOX:A	48.87	1081	449	5	444
S4A001	1GNX:A	69.77	1680	479	13	477
Q9K440	1GNX:A	57.64	1162	479	18	470
F5WYI5	4B3K:A	65.50	1715	479	2	459
A0A0Q8HZT7	4HZ6:A	45.48	991	444	4	440
A0A062URN0	3CMJ:A	46.40	1004	465	26	453
A0A124GX52	1GNX:A	65.11	1476	479	13	477
A0A117P4A6	1NP2:A	51.90	976	436	6	430
E7FS70	1BGG:A	30.04	487	448	6	442
A0A0Y0P6F3	3W53:A	65.94	1634	506	45	503
S7U581	4B3K:A	49.13	1224	479	3	459
A0A0Q9A2C2	1OD0:A	43.89	1012	468	24	461
E7FMU7	3CMJ:A	30.60	560	465	24	464
A0A117P3R8	1GNX:A	64.89	1613	479	9	477
Q9F3I3	1GNX:A	65.95	1619	479	12	477
Q9F3B7	1GNX:A	89.35	2203	479	1	479
A0A0Q5B5F0	3W53:A	69.65	1708	506	45	502
F3YCS4	1QOX:A	33.55	656	449	5	445
A0A117NTJ8	3WH5:A	56.19	1240	457	6	440
S3Z732	3ZJK:A	51.34	987	431	8	430
A0A0D0Q9A4	3TA9:A	53.08	1232	458	11	457
D4HVI8	1QOX:A	36.93	720	449	5	443
A0A0Q5M1X8	3W53:A	71.76	1797	506	37	500
Q8CJM3	3WH5:A	55.20	1215	457	6	446
A0A0L8PFU2	1NP2:A	49.43	885	436	12	430
A0A068TBI5	1OD0:A	46.01	1066	468	28	462
A0A0C1Z713	2O9P:A	46.06	1048	454	18	449
A0A0L8P752	3TA9:A	50.88	1164	458	13	450
A0A101Q455	3WH5:A	55.33	1238	457	6	445
H2K5D1	1NP2:A	49.32	892	436	12	430
A0A101QHN0	1GNX:A	64.10	1456	479	10	472
H2JLX8	1GNX:A	67.82	1627	479	16	477
K2QF48	4B3K:A	55.96	1478	479	1	469
H2JP17	3WH5:A	56.11	1239	457	6	446
H2JI37	1OD0:A	41.87	943	468	28	465
A0A0L8NWB2	1GNX:A	69.31	1718	479	1	477
K2QBJ7	4B3K:A	46.30	1120	479	3	459
K2QB67	4PTV:A	37.96	765	452	7	450

B2JNV8	4PTV:A	46.01	1040	452	1	438
A0A0B5DTX4	1OD0:A	44.44	949	468	28	453
H2JLX3	1GNX:A	69.10	1715	479	1	477
A0A0F5FJM3	1OD0:A	48.86	1082	468	28	464
A5UZB6	3TA9:A	52.65	1264	458	8	456
A0A0K0TRD4	3TA9:A	48.00	1047	458	13	436
A0A101QFE1	1NP2:A	49.55	912	436	8	426
A0A117QH84	1GNX:A	66.60	1639	479	12	477
H2JXT2	3TA9:A	50.67	1171	458	13	450
A0A0L8NBA6	3WH5:A	56.79	1242	457	6	446
K2PXX1	1TR1:A	37.74	878	447	5	441
D4KCX2	1VFF:A	32.41	556	423	4	401
L0EHU9	1BGG:A	55.70	1420	448	1	445
A0A0J5AB40	3TA9:A	46.53	1054	458	13	450
A0A062VPY9	3W53:A	56.12	1268	506	40	500
L0EBS9	1QOX:A	60.13	1511	449	1	449
A0A0X3WTY9	3WH5:A	55.58	1208	457	6	434
A0A0S3FIH6	3TA9:A	47.10	1066	458	13	450
A0A0W8ADG0	3TA9:A	47.32	1067	458	13	450
A0A0Q6Y678	1GNX:A	61.30	1454	479	6	479
A0A0Q6XYF1	3W53:A	58.48	1323	506	60	501
A0A0X3WHC3	1GNX:A	68.97	1634	479	18	479
D4DZX8	1UYQ:A	35.64	651	447	4	441
D9SVH0	3AHX:A	100.00	2388	453	1	445
A0A0X3WVZ5	1GNX:A	76.41	1894	479	1	477
V0ARF9	3TA9:A	47.32	1071	458	13	450
A0A0J4RM61	3TA9:A	46.31	1054	458	13	450
A0A060VPE0	3TA9:A	47.10	1064	458	13	450
A0A0Q6Y5F7	3W53:A	64.57	1645	506	35	506
Q12KZ0	3TA9:A	43.65	996	458	10	451
A0A0R3JS14	5DT5:A	57.50	1459	471	24	463
D4E282	4B3K:A	40.73	955	479	1	461
W9BGS1	3TA9:A	47.32	1070	458	13	450
A0A101KYQ6	3W53:A	87.50	2213	506	35	506
B0K276	4B3K:A	48.70	1187	479	3	462
A0A062VVQ8	3W53:A	43.04	913	506	35	504
A0A0V9IX77	3TA9:A	47.10	1059	458	13	450
N9VN24	1OD0:A	45.33	1051	468	28	466
Q13PT6	5DT5:A	45.43	1034	471	29	465
D4LQI8	1OD0:A	45.41	1038	468	22	465
A0A0V9RYC4	4PTV:A	46.31	1052	452	7	444
A0A101LBH8	1QOX:A	44.27	965	449	9	442
D4KAE7	1OD0:A	32.85	545	468	27	467
B1VQ99	1GNX:A	52.06	1076	479	18	472
A0A0P6W4L4	5DT5:A	79.82	1977	471	24	464
A0A100J4D8	1VFF:A	32.94	555	423	9	404
A0A100J4D6	1GNX:A	53.09	1179	479	16	477
F9P3I4	4QLJ:A	32.81	332	481	75	323
B1VL16	1GNX:A	68.61	1607	479	16	475
A0A100J537	1VFF:A	35.17	594	423	1	404
A0A0L0KNJ9	1GNX:A	67.45	1646	479	10	477
N0CZM8	1GNX:A	67.60	1596	479	16	476
A0A0Q5IFM2	1NP2:A	47.94	938	436	8	431
A0A150VR81	3WH5:A	54.26	1218	457	4	448
B1VSM7	3WH5:A	56.79	1260	457	6	450
B0RIF2	1GNX:A	59.96	1383	479	16	477
A0A0Q0PYE0	4PTV:A	46.59	1077	452	6	444

Q7NYK2	1QOX:A	41.05	879	449	1	449
B1VZ46	1GNX:A	56.99	1174	479	18	472
A0A100JCY1	1NP2:A	47.54	876	436	7	430
A0A117EB11	1VFF:A	35.63	622	423	2	404
A0A0L0JVU3	1NP2:A	47.54	876	436	7	430
A0A100J6F7	1GNX:A	56.14	1229	479	8	473
N0CP03	1GNX:A	69.17	1677	479	4	479
J3C6P0	1OD0:A	44.47	1018	468	24	462
R4LMQ4	1GNX:A	54.74	1218	479	1	473
B1VWD4	3ZJK:A	48.43	903	431	8	430
N0D629	3WH5:A	57.21	1275	457	6	448
B1VW56	1GNX:A	69.52	1676	479	2	477
A0A0L0K9F0	1GNX:A	55.72	1223	479	8	473
N0D4U4	3WH5:A	46.07	909	457	1	444
R4LKY4	1GNX:A	52.08	1075	479	18	469
A0A0F5PXV3	1OD0:A	47.27	1039	468	26	464
A0A150VRJ4	1GNX:A	74.68	1773	479	14	476
A0A0E0Z721	4PTV:A	43.70	1034	452	7	446
N0D4R4	1GNX:A	55.89	1198	479	7	472
R4LWC4	1GNX:A	57.39	1301	479	16	472
M5QUM2	3TA9:A	54.42	1233	458	13	450
A0A0U2ZC99	1NP2:A	49.43	1016	436	5	433
A0A0H5D2M0	3TA9:A	46.02	1058	458	13	454
G0L4C6	1OD0:A	44.42	976	468	28	462
A0A0Q9D8P1	1QOX:A	47.62	1042	449	5	441
A9WAY6	3CMJ:A	56.48	1257	465	22	463
Q6AKE8	1OD0:A	43.17	961	468	22	466
H0K1S0	3W53:A	53.81	1233	506	45	502
A0A0H5CJN4	1GNX:A	59.52	1361	479	16	472
J1TBL4	1QOX:A	47.06	1040	449	5	444
F8CBN7	3TA9:A	50.79	1231	458	13	452
A0A074JXI5	1OD0:A	44.47	976	468	21	453
A0A127A2M4	1NP2:A	49.03	974	436	6	430
A0A0F4RW23	3TA9:A	43.15	995	458	12	452
A0A085ZID8	1OD0:A	46.01	1050	468	27	461
A0A101BU84	5AYI:A	43.42	844	457	2	438
F7X9K8	1QOX:A	47.51	1041	449	5	444
A0A0Q6WMT4	4PTV:A	45.80	1040	452	6	445
R4TBK3	1NP2:A	52.57	1057	436	6	428
A0A0F4RQE6	1OD0:A	44.29	994	468	26	453
A0JZ86	1QOX:A	43.39	932	449	8	441
A0A0J6XU50	1GNX:A	64.97	1524	479	9	477
A0A0Q8ZE07	3WH5:A	56.15	1243	457	6	449
A0A086ZKQ3	1OD0:A	49.55	252	468	365	466
A0A0F4SDI3	5DT5:A	42.41	963	471	15	462
A0A0Q8LU77	1GNX:A	62.18	1446	479	13	477
C6WCL5	1GNX:A	54.23	1213	479	14	472
J2WDV1	1OD0:A	44.59	979	468	28	466
Q89L91	3TA9:A	42.92	921	458	17	454
A0A0Q6XZ00	3AHX:A	46.83	1097	453	1	442
A0A0J7AKL5	3ZJK:A	51.57	1026	431	8	426
A0A154IS38	1QOX:A	47.29	1041	449	5	444
R4THI3	3W53:A	48.80	1074	506	46	499
A0A0M2ZFB9	3CMJ:A	40.35	858	465	24	463
Q89H18	3WH5:A	45.12	908	457	6	441
R4SWC3	3WH5:A	52.04	1161	457	6	446
A0A0J6XMR6	3WH5:A	54.32	1180	457	6	444

A0A0J6XGP7	1GNX:A	71.12	1696	479	14	476
A0A0Q9B0L2	1GNX:A	67.87	1645	479	12	479
C6WCW9	3W53:A	55.60	1330	506	41	502
U1LD59	3CMJ:A	46.19	1019	465	30	458
A0A0Q6X151	1QOX:A	53.08	1265	449	5	445
R4T6P4	1GNX:A	48.48	1057	479	18	478
A0A0Q9BCM6	3WH5:A	47.36	927	457	5	443
Q89UP6	3WH5:A	45.95	927	457	3	441
A0K0K0	3TA9:A	41.79	939	458	13	451
A0A154R397	1OD0:A	49.77	1115	468	27	465
A0A0Q6W596	3W53:A	42.83	972	506	45	504
R4SR38	3TA9:A	44.04	989	458	8	450
A0A0U5FP09	3TA9:A	47.30	1045	458	13	455
V5X0Q1	2O9P:A	90.83	2229	454	8	454
D5AU75	1OD0:A	45.48	1003	468	26	453
A0A151CU35	3W53:A	53.89	1238	506	44	501
A0A0B5ARU7	3TA9:A	50.23	1178	458	13	450
R4YWU4	1OD0:A	35.95	651	468	28	464
I0HYI5	4PTV:A	52.64	1310	452	2	450
A0A085G5B6	1QOX:A	40.34	804	449	5	447
A0A0Q8N5B6	1OD0:A	50.00	1115	468	27	465
E0MSZ2	1OD0:A	42.66	920	468	26	453
A0A0S8B4Z3	3TA9:A	48.90	1199	458	8	456
A0A059F8D5	1GNX:A	47.59	965	479	18	472
A0A0U5MJM8	1NP2:A	45.54	906	436	8	432
A0A022L2R1	3W53:A	55.01	1252	506	44	499
W6W1F3	1OD0:A	46.22	1066	468	25	457
A0A0L1LFK6	2O9P:A	44.74	1075	454	10	449
A0A0Q7LPS6	1QOX:A	46.61	1023	449	5	444
A0A085GPH6	4B3K:A	42.42	1026	479	1	459
A0A0P7CMK7	3W53:A	52.78	1182	506	46	505
V5X1T6	1BGG:A	97.77	2382	448	1	448
A0A085H4A7	3TA9:A	44.10	1014	458	13	450
A0A0F5YLR6	1OD0:A	45.12	968	468	27	464
A0A101NXW0	1GNX:A	67.82	1630	479	16	477
A0A117SXL6	4PTV:A	49.77	1155	452	7	446
Q97M15	3AHX:A	44.02	976	453	4	445
A0A0B2A5B4	3ZJK:A	48.86	955	431	6	430
A0A117Q144	3WH5:A	55.66	1215	457	6	446
A0A085GZG7	4B3K:A	46.96	1146	479	2	459
A0A014MMT7	3WH5:A	55.66	1233	457	6	446
A0A124HFX4	1NP2:A	49.66	913	436	6	430
A0A0B2A6F6	3W53:A	52.38	1224	506	39	505
I4VUG3	1OD0:A	49.77	1111	468	27	465
A0A037ZIH9	1OD0:A	44.13	951	468	28	453
A0A014LC96	1GNX:A	73.23	1768	479	16	479
A0A139M7S9	5FOO:A	48.48	1175	480	1	460
A0A117MJG3	1NP2:A	51.01	935	436	8	430
A0A0Q7VAY4	3WH5:A	45.20	906	457	1	444
A0A0Q7U570	1GNX:A	66.81	1572	479	14	475
M9TW44	1GNX:A	70.43	1724	479	11	477
A0A060I1G7	1QOX:A	48.28	1061	449	5	439
Q2ITY8	3TA9:A	43.47	978	458	16	458
M9U0R4	3WH5:A	45.20	910	457	1	444
A5CT94	1NP2:A	41.08	639	436	11	426
A0A139QSZ8	5FOO:A	48.70	1176	480	1	460
B7GU31	1GNX:A	52.25	1085	479	16	432

A0A139PV94	4B3K:A	63.29	277	479	381	459
A0A139PFS0	4B3K:A	49.02	1176	479	1	459
I7EAL6	1OD0:A	43.88	961	468	23	453
A0A100ZF69	5AYI:A	43.20	843	457	2	438
A0A0Q7UIX8	1GNX:A	55.37	1202	479	1	472
A0A084GQB3	3AHX:A	40.69	976	453	4	440
A0A0Q7U8Q1	1GNX:A	71.03	1725	479	15	477
A0A0S6VXA0	3WH5:A	56.69	1334	457	5	444
M9U478	1GNX:A	56.03	1194	479	7	467
A0A139RP68	4B3K:A	48.81	1168	479	1	459
A0A089J646	1BGG:A	60.18	1545	448	1	446
A0A0X3V0H3	1GNX:A	66.17	1622	479	12	477
A0A0Q7V4M3	1GNX:A	53.56	1112	479	17	470
A0A081BMQ2	1OD0:A	43.58	996	468	27	465
A0A139Q539	5FOO:A	48.92	1190	480	1	460
A0A139QXT6	5FOO:A	48.70	1177	480	1	460
A0A139PLL6	5FOO:A	48.27	1158	480	1	460
F8F2L0	3TA9:A	50.22	1125	458	8	450
M9TXM8	1GNX:A	54.00	1121	479	17	470
A0A031IDU7	5DT5:A	84.08	2105	471	24	469
A0A0N1NTA5	3WH5:A	56.82	1243	457	6	444
A0A139NW12	4B3K:A	48.81	1176	479	1	459
A0A081BLU3	1OD0:A	45.54	1049	468	28	465
A0A139P1Y0	4B3K:A	48.59	1167	479	1	459
A0A0X3VQR3	1GNX:A	63.62	1485	479	2	477
A0A0Q7TR74	3WH5:A	56.92	1260	457	6	449
A0A0N1H2Z5	3WH5:A	46.48	931	457	5	444
A5CM23	1GNX:A	51.81	1108	479	16	477
A5CPC2	1GNX:A	60.30	1384	479	16	477
A0A139PHH0	4B3K:A	50.83	790	479	159	459
A0A139PVD8	5FOO:A	46.28	726	480	1	307
Q8RCQ8	5DT5:A	55.91	1409	471	24	461
D2Q763	1GNX:A	52.95	1161	479	17	469
D4F1U7	3TA9:A	44.18	1053	458	13	456
Q5WL98	4B3K:A	46.85	1131	479	3	459
G2NE89	1GNX:A	69.65	1720	479	7	477
A1R8U7	1GNX:A	46.14	943	479	5	472
A0A139R2H8	1VFF:A	34.23	441	423	71	400
A0A0E9N138	1OD0:A	41.03	877	468	24	461
A0A076JFX4	1GNX:A	50.84	1202	479	16	476
A0A069DJR7	1BGG:A	72.93	1859	448	1	447
G2NJG8	1GNX:A	54.00	1134	479	17	470
G2NFR6	1GNX:A	56.25	1200	479	7	467
H0T5R6	3TA9:A	45.41	969	458	17	450
H0T1Z3	1OD0:A	42.86	931	468	26	467
G2NB62	3WH5:A	53.41	1175	457	6	444
G2NPU1	3ZJK:A	48.65	899	431	8	430
Q5WBD7	1QOX:A	33.83	562	449	1	443
Q5WKT0	4B3K:A	49.78	1220	479	2	459
A0A139QTD8	1TR1:A	33.12	610	447	5	441
A1R103	3W53:A	66.45	1679	506	45	506
A0A0T6YP47	1QOX:A	47.85	1047	449	5	441
A0A0M8KEI8	3CMJ:A	46.26	1025	465	24	460
A0A076MR11	3CMJ:A	44.49	940	465	21	458
B0KCW7	4B3K:A	48.70	1187	479	3	462
I2RVU7	1QOX:A	33.41	553	449	20	449
B0KDF9	5DT5:A	55.94	1393	471	25	460

A0A149W1J7	1VFF:A	30.65	516	423	4	408
K8BWT5	1UYQ:A	34.44	631	447	5	441
J1NZZ1	1UYQ:A	33.05	595	447	5	441
K8CBQ1	4B3K:A	47.72	1140	479	2	459
A0A0D6SQK1	3AHX:A	41.38	922	453	4	441
A0A0N1AA72	1OD0:A	45.12	1031	468	25	461
J0V2T6	4B3K:A	52.33	1194	479	31	459
A0A087CIL9	1VFF:A	34.38	573	423	4	405
S9RLG1	1OD0:A	42.34	967	468	21	463
A0A0D6T6K2	1QOX:A	37.77	807	449	5	443
K8C261	4B3K:A	40.93	965	479	1	470
B0KCV1	5DT5:A	55.43	1403	471	24	463
K8BYF6	4B3K:A	48.65	470	479	2	184
A0A074U5W9	1OD0:A	44.62	997	468	21	465
J5HAL3	1UYQ:A	34.82	611	447	4	441
F4KVL5	4GXP:A	53.46	1253	467	11	465
K7QWH8	1NP2:A	97.31	1893	436	1	372
N1V5H2	1NP2:A	46.34	918	436	7	423
Q3Y0M8	3TA9:A	31.70	545	458	8	451
Q3Y0A1	3CMJ:A	30.97	285	465	215	463
N1UX26	3W53:A	56.20	1364	506	39	502
A0A0F3H2P5	4B3K:A	46.62	1101	479	3	459
A0A0K2FDL8	1BGG:A	97.77	2386	448	1	448
J7LI95	3W53:A	66.02	1675	506	45	506
K7R8A6	3ZJK:A	75.89	1630	431	8	426
J7LYT0	1GNX:A	45.93	941	479	5	472
Q3XXN2	3TA9:A	41.42	920	458	13	452
A0A0S2FAN7	1OD0:A	51.56	1189	468	23	465
Q3Y0A9	4B3K:A	47.94	1171	479	3	459
I3U0Z7	4B3K:A	46.32	1141	479	2	459
J7LN09	3TA9:A	41.33	795	458	60	450
A0A147IX49	1OD0:A	45.27	1016	468	17	466
R6NN85	1OD0:A	46.12	1000	468	28	465
A0A0R3LCP3	3TA9:A	44.44	938	458	13	455
A0A0R3LYE3	3CMJ:A	44.49	920	465	26	463
A0A0K2FD16	2O9P:A	90.38	2217	454	8	454
J3ANZ0	3WH5:A	44.74	930	457	6	444
A0A0A0BZ90	3W53:A	53.28	1256	506	43	496
A0A0T5PLD9	3WH5:A	47.36	987	457	8	439
R8Q5X0	3TA9:A	40.91	931	458	13	451
I0V7N4	3W53:A	54.03	1226	506	45	502
D2AUF2	3CMJ:A	48.97	1076	465	30	461
B1IAA0	1UYQ:A	33.05	591	447	5	441
D2BAN2	1GNX:A	57.08	1319	479	1	472
A0A0Q8E7R1	3WH5:A	56.47	1261	457	6	449
A0A0C2W2Y2	1BGG:A	58.37	1486	448	1	440
A3D1N8	3CMJ:A	42.48	979	465	15	458
A0A0Q5VUS1	1OD0:A	43.48	972	468	28	463
D2BAN4	1GNX:A	55.27	1243	479	18	472
A0A0Q8F6H6	1QOX:A	46.61	1038	449	5	443
A0A0A0C0T3	1GNX:A	59.45	1382	479	10	477
A0A0Q8EP60	1GNX:A	68.10	1607	479	14	476
A0A0Q8EKW6	3CMJ:A	48.65	1077	465	21	458
W6K116	3CMJ:A	45.11	1023	465	23	458
A0A0Q8E1S0	3WH5:A	45.39	906	457	6	446
A0A087CQV8	1VFF:A	34.38	574	423	4	405
A0A0Q8F3Y1	1GNX:A	69.79	1669	479	11	477

A0A0Q5VVI3	1QOX:A	47.85	1034	449	5	443
A0A061QAE7	2O9P:A	45.37	1036	454	18	449
D2BDF5	1GNX:A	55.53	1289	479	16	476
A0A139MLS4	1UYQ:A	32.40	475	447	70	441
A0A0T1WPD0	1OD0:A	46.03	1048	468	25	461
K0PLM8	1QOX:A	47.96	1078	449	5	444
A0A0Q8E4G9	1GNX:A	56.99	1183	479	18	472
D2B261	1GNX:A	54.10	1086	479	18	467
R7H2Z3	4GXP:A	43.59	980	467	12	467
A0A0B5ELK7	1NP2:A	50.11	947	436	1	426
A9F279	3TA9:A	51.85	1245	458	8	456
A0A084GQG8	3AHX:A	40.48	972	453	4	440
A0A0B5EHK8	1GNX:A	71.22	1743	479	12	477
W7VBV3	1GNX:A	55.92	1211	479	17	472
I6ARU5	3CMJ:A	42.15	872	465	19	463
C0X123	1UYQ:A	31.33	601	447	3	441
I6APS1	1OD0:A	42.57	933	468	28	464
C0X5J2	1OD0:A	38.19	749	468	25	468
A0A0F4PXY4	3CMJ:A	46.26	1019	465	24	460
A0A0Q5VGQ5	1GNX:A	53.63	1161	479	5	472
J5U9U2	3TA9:A	46.65	1052	458	13	450
G1Y4C7	4PTV:A	46.21	1070	452	4	445
A9F5S4	1OD0:A	45.86	1039	468	28	465
I6AYS5	1UYQ:A	42.76	930	447	3	444
A0A0B4Y698	1OD0:A	45.49	1061	468	16	466
I6APM1	1E4I:A	41.07	907	447	3	441
C0X4F7	3TA9:A	41.20	893	458	13	451
Q92LX2	1QOX:A	47.51	1041	449	5	444
W7VLW8	3CMJ:A	48.65	1079	465	23	458
W7VRE1	1GNX:A	52.47	1082	479	18	472
A0A0B5F5Z8	3WH5:A	54.05	1202	457	6	448
W7VM47	1GNX:A	56.90	1265	479	16	475
W7VGI0	1GNX:A	60.78	1319	479	15	477
A0A0D1LRQ3	3WH5:A	47.20	935	457	3	441
A9GNP8	1OD0:A	45.45	1013	468	23	465
A0A0P7WQB8	1OD0:A	42.79	958	468	21	462
A0A0J7HLK2	1OD0:A	46.12	1062	468	28	465
A0A087EGL8	1GNX:A	48.85	1123	479	18	476
A7NGQ8	3TA9:A	52.82	1246	458	8	450
W7W731	3CMJ:A	52.48	1201	465	25	463
A0A0B2FD09	1BGG:A	59.51	1520	448	1	446
J5UTN3	1UYQ:A	34.85	664	447	5	441
F9Y846	2O9P:A	45.31	1027	454	14	450
A0A0M6XTZ2	3CMJ:A	46.50	950	465	26	450
L8F638	1GNX:A	56.62	1345	479	10	477
A0A0J1G016	4HZ6:A	42.15	925	444	4	438
U2T8H7	3TA9:A	30.83	507	458	12	450
A0A101R1A6	1GNX:A	89.98	2210	479	1	479
R6PXX2	1OD0:A	45.71	1056	468	27	467
U2T8E4	1UYQ:A	28.95	511	447	5	441
A0A0B3SAE8	1OD0:A	46.67	1009	468	21	453
A0A117QML6	1GNX:A	67.31	1650	479	11	477
A0A0R3C374	3WH5:A	46.43	926	457	3	441
F4GLK2	3TA9:A	48.29	1093	458	13	450
A0A087AXE0	1GNX:A	53.63	1207	479	17	467
A7ZAD8	1UYQ:A	33.97	658	447	4	441
F4GH96	1VFF:A	35.08	589	423	1	416

L0K7D4	3TA9:A	65.85	1641	458	8	455
A0A0B4X8M0	1QOX:A	47.74	1062	449	5	444
A0A0V8I648	3ZJK:A	48.67	1018	431	3	430
H0QPX1	1QOX:A	45.45	945	449	8	441
W0ZB74	3W53:A	55.32	1334	506	34	505
A0A0S2G7M6	1OD0:A	51.01	1166	468	26	465
A0A087AYW0	1GNX:A	51.32	1193	479	18	476
W0Z7Z9	1GNX:A	60.96	1444	479	4	477
A0A0D8CBC8	1OD0:A	45.96	1061	468	25	466
A0A117QPQ7	3WH5:A	56.08	1226	457	6	448
A0A0J1IZ59	3TA9:A	44.44	958	458	10	446
A0A0Q4PVH6	3TA9:A	46.78	1071	458	13	456
A0A0F0H1R8	1GNX:A	54.00	1189	479	16	477
A0A0F0GB25	3W53:A	54.00	1273	506	41	502
A0A0Q5BW34	1GNX:A	52.77	1125	479	16	478
A0A0L7BT19	1GNX:A	52.99	1248	479	16	477
A0A0Q5C7T9	3W53:A	53.45	1225	506	39	499
A0A0C2V0V3	4PTV:A	42.15	920	452	4	445
A0A0F7JQC2	1OD0:A	41.26	943	468	25	464
A0A0F7JM33	3WH5:A	54.75	1214	457	5	441
A0A0F0GS07	3W53:A	52.39	1172	506	46	503
G4NYJ9	1UYQ:A	32.17	613	447	4	441
A0A0Q5C0E3	3W53:A	69.21	1696	506	45	502
A0A0A0NSJ2	1GNX:A	73.02	1756	479	16	479
G4NQU7	3AHX:A	41.54	865	453	6	441
A0A0C2YSN9	3TA9:A	46.10	1025	458	13	456
V5WKT4	1OD0:A	41.99	937	468	28	467
A0A0F4KAY0	1GNX:A	68.40	1597	479	16	477
D3RPI3	3TA9:A	50.91	1147	458	13	452
A0A0F0H025	3TA9:A	41.78	928	458	15	451
A0A0A0N9S0	3WH5:A	55.66	1236	457	6	446
G4NYI0	1QOX:A	38.54	798	449	1	443
A0A139RE39	4B3K:A	47.94	1158	479	1	459
G4NR65	4B3K:A	49.89	1202	479	3	456
A0A0B5IJ38	1GNX:A	74.57	1786	479	14	476
K8B7R2	4B3K:A	47.29	1133	479	2	459
F2JPA6	1OD0:A	46.33	1027	468	27	465
K0JWV1	3W53:A	57.02	1308	506	45	506
A0A0B5IE61	1GNX:A	67.73	1585	479	10	479
G4F057	3TA9:A	39.57	856	458	13	451
A7N5G4	3TA9:A	45.23	1039	458	8	450
K0JRE9	1NP2:A	50.35	915	436	6	428
K0KB51	1GNX:A	55.35	1192	479	7	479
Q1CYU5	1VFF:A	37.44	684	423	5	408
U1Y804	1NP2:A	47.56	948	436	8	424
A0A090SXD7	1QOX:A	39.27	806	449	5	442
L1L7Z9	1GNX:A	66.74	1631	479	16	477
A0A117L4W2	3TA9:A	56.05	1370	458	13	458
A0A0B5I7U1	1GNX:A	52.71	991	479	18	467
A0A090SNE8	4B3K:A	40.80	960	479	1	470
K8AZ31	4B3K:A	41.16	928	479	1	444
K8BI51	1BGA:A	39.38	375	447	5	199
E8UXX5	1OD0:A	42.79	916	468	28	466
K8AW43	4B3K:A	45.00	1052	479	3	459
K0K6D9	3W53:A	50.11	1098	506	45	502
L1L4D1	1NP2:A	51.12	963	436	8	433
K8B4Z9	3CMJ:A	32.08	254	465	242	464

A0A0B5HY92	1GNX:A	53.98	1077	479	18	467
A0A090SKA4	5FOO:A	44.98	1066	480	3	458
Q1CY46	3TA9:A	50.56	1228	458	13	456
I3XDD0	1QOX:A	47.51	1034	449	5	444
R8HJ90	3TA9:A	40.69	930	458	13	451
H8MU62	3WH5:A	53.36	1186	457	5	441
E8JUK5	4GXP:A	29.01	335	467	68	423
A0A099IN71	3WH5:A	45.12	908	457	6	441
A0A0N8W654	1OD0:A	39.73	848	468	28	462
A0A0U3FRQ6	1QOX:A	44.71	1003	449	1	442
A0A0Q9CVH6	1GNX:A	61.37	1424	479	18	477
A0A0M9JGD5	1GNX:A	55.58	1177	479	18	472
A0A0M7QM17	1GNX:A	73.61	1756	479	14	478
A0A0N7FAE4	3W53:A	86.86	2199	506	35	506
A0A0M7R2Z5	3WH5:A	53.88	1217	457	4	448
A0A151DH08	3WH5:A	55.68	1228	457	6	444
A0A151D8A3	1NP2:A	52.26	958	436	8	426
A0A151DI30	1GNX:A	90.19	2221	479	1	479
A0A0M7QHN9	3ZJK:A	50.56	1002	431	8	426
D9UP56	1GNX:A	70.17	1734	479	3	477
A0A0Q7QAP1	4PTV:A	43.89	986	452	4	444
A0A0M0T212	5DT5:A	89.73	2230	471	24	471
A0A099IJ12	3CMJ:A	44.72	924	465	26	463
A0A085PF90	3TA9:A	46.59	1075	458	12	450
A0A0Q9CXP9	3W53:A	57.14	1367	506	42	506
A0A151DN79	1GNX:A	57.86	1167	479	18	470
A0A0S9M311	1OD0:A	50.57	1157	468	28	465
A0A151D612	1GNX:A	66.17	1631	479	12	477
A0A0M7QGT7	1GNX:A	66.38	1557	479	15	476
W9GB96	3WH5:A	46.38	899	457	2	437
A0A0C1LYC1	3CMJ:A	46.42	1016	465	30	458
A0A139MES3	1OD0:A	36.31	264	468	308	465
A0A139MRB6	1QOX:A	40.46	904	449	5	449
A0A0E1CAA6	4B3K:A	45.04	1067	479	3	463
A0A139R6K5	1QOX:A	40.66	910	449	5	449
A0A0S8EL39	4PTV:A	48.56	1145	452	2	446
A0A139NB07	1GNX:A	34.60	620	479	17	474
C4L1S4	5DT5:A	83.26	2061	471	24	465
Q6N915	4PTV:A	43.47	954	452	9	451
A0A0N0B0F3	3WH5:A	55.15	1203	457	11	446
A0A0M8Z4E7	1GNX:A	64.89	1607	479	9	477
A0A0E1C991	4B3K:A	40.30	968	479	1	470
A0A0E1CHW4	3TA9:A	47.32	1068	458	13	450
D3F6P8	1GNX:A	54.96	1228	479	14	476
A0A142KH94	1GNX:A	59.13	1350	479	18	476
A4XIG7	3AHX:A	52.84	1260	453	4	444
V4MY52	3AHX:A	49.20	1122	453	6	440
A0A0F4VWL7	3AHX:A	41.90	971	453	4	441
A0A086BNK9	1VFF:A	35.17	629	423	3	410
A0A0F4VX87	4B3K:A	58.82	1523	479	1	459
A0A0M8Z1Z8	1GNX:A	57.86	1157	479	18	470
A0A142KE92	3W53:A	46.44	1066	506	41	499
A0A024KFD3	3TA9:A	48.71	1061	458	13	436
R5WJG7	3TA9:A	46.31	1051	458	13	450
C4L1S9	3AHX:A	40.91	972	453	4	440
A0A139QJB7	1QOX:A	34.90	325	449	214	449
B9K076	4PTV:A	43.05	960	452	7	444

C7XXG7	3CMJ:A	31.48	545	465	23	464
C4L0E9	4B3K:A	50.55	1216	479	3	455
B9JSR2	1QOX:A	46.83	1049	449	5	444
W9GDW6	3W53:A	55.51	1301	506	46	501
A0A0M8Z8E8	1NP2:A	50.78	939	436	1	427
A0A016QQV3	3WH5:A	54.59	1181	457	6	440
A0A150LW25	4B3K:A	49.78	1250	479	1	459
D5HDJ9	1OD0:A	38.76	806	468	28	465
A0A0E3ZBU6	1OD0:A	44.90	999	468	28	464
A0A0M3UGE2	3TA9:A	44.30	996	458	12	454
A0A031JM39	1VFF:A	32.66	548	423	4	408
I2CBS4	4B3K:A	49.24	1195	479	3	459
A0A0J0Y754	1OD0:A	39.73	835	468	28	463
A0A0M2WQN6	3TA9:A	48.87	1074	458	13	450
D1A1R2	1GNX:A	66.08	1478	479	18	470
C3M8X6	1E4I:A	46.49	1030	447	4	438
A0A0H4RRS8	3AHX:A	40.43	977	453	6	440
H3MW96	3TA9:A	45.86	1052	458	13	450
A0A0Q9TTG0	3W53:A	56.40	1313	506	46	503
A0A150LGF5	3AHX:A	40.72	891	453	2	445
A0A150LIB8	4B3K:A	49.46	1224	479	3	459
V6JZT7	1GNX:A	68.32	1664	479	16	478
H0BE14	1GNX:A	67.46	1592	479	14	476
I2C178	3AHX:A	40.55	869	453	6	445
A0A150LBY8	1OD0:A	36.48	682	468	29	467
A0A0Q6MA37	5DT5:A	46.42	1085	471	23	454
D1A786	1NP2:A	54.55	1088	436	7	430
H9U9K9	3TA9:A	56.04	1260	458	13	451
V6JX71	1NP2:A	49.44	907	436	6	430
H0BDP8	1GNX:A	68.89	1672	479	2	477
D5VFQ0	1QOX:A	43.27	914	449	3	447
A0A0P1H107	1OD0:A	43.09	974	468	28	453
A0A0F5FIF7	4PTV:A	49.31	1059	452	1	430
H0BCI1	3WH5:A	56.25	1259	457	6	449
D5HDE6	3TA9:A	38.51	837	458	13	451
A0A0Q6MCU6	3TA9:A	47.57	1086	458	8	454
H0BLZ7	3ZJK:A	48.65	922	431	8	430
D1AC41	1GNX:A	56.72	1298	479	4	477
H0BIK3	1GNX:A	56.77	1173	479	18	472
C7PAW2	1OD0:A	44.14	984	468	28	463
T0UEH1	4B3K:A	49.61	379	479	332	459
T0U295	4B3K:A	47.47	904	479	106	459
A0A0F7BQ11	3TA9:A	51.69	1252	458	13	452
A0A0Q7NQ96	1QOX:A	46.03	1034	449	5	443
D9WNM9	1GNX:A	71.89	1751	479	16	479
T0U0H1	4B3K:A	64.15	1477	479	50	459
M4ZDN8	3TA9:A	43.91	954	458	17	450
K8CT21	4B3K:A	40.93	965	479	1	470
W9ADI0	5DT5:A	66.06	1695	471	25	465
A0A0Q7UH73	3W53:A	55.98	1342	506	44	501
K8CMR9	3CMJ:A	32.84	280	465	218	464
M4Z8X1	1OD0:A	42.28	933	468	25	465
A6WF35	1GNX:A	61.25	1451	479	6	479
T0VQA6	3TA9:A	41.29	930	458	13	451
T0UE82	5FOO:A	44.42	1074	480	1	464
T0VJW6	1QOX:A	38.36	754	449	5	445
T0U8Z8	4B3K:A	44.88	760	479	3	331

T0U8V9	4B3K:A	55.81	131	479	1	41
T0U8J7	4B3K:A	50.00	94	479	64	97
T0VMS9	4B3K:A	38.28	891	479	1	461
A0A0U3EGQ1	3AHX:A	46.40	1090	453	5	447
A0A0U9H595	5DT5:A	65.84	1694	471	25	465
K8D7Z9	4B3K:A	47.51	1135	479	2	459
D3Q414	1GNX:A	51.31	1161	479	18	474
G8NVF9	3CMJ:A	43.05	991	465	16	463
M7R6A1	4PTV:A	45.68	1040	452	2	444
A0A0Q6S7V2	1OD0:A	46.28	1071	468	25	463
B5HNI5	1GNX:A	65.68	1614	479	7	477
A0A117L8X6	1OD0:A	98.65	2336	468	25	468
A0A101NSI8	3WH5:A	56.79	1255	457	6	446
R9PFT7	4PTV:A	44.99	940	452	39	446
A0A0A7FUT5	3AHX:A	43.53	982	453	4	441
D3Q2J6	1NP2:A	50.46	1009	436	6	427
A0A099JMJ2	3W53:A	57.54	1365	506	39	505
B5I7D1	3WH5:A	56.38	1266	457	6	449
B5HXI9	1NP2:A	51.24	969	436	8	430
L0LQR9	1QOX:A	48.31	1082	449	5	444
A0A0E0X0A5	3TA9:A	46.67	1052	458	13	450
T0BPF3	2O9P:A	54.11	1316	454	14	450
A0A101NDF9	1QOX:A	46.39	1082	449	4	443
M3EIG2	1QOX:A	60.30	1361	449	52	449
B5I181	1GNX:A	63.68	1464	479	16	478
A0A101NKE4	1GNX:A	68.90	1660	479	16	477
A0A101NFG4	1NP2:A	48.13	908	436	1	430
G8P195	3TA9:A	42.68	1028	458	8	456
G8NWT0	3TA9:A	45.01	958	458	13	456
A0A0A7PHH8	1OD0:A	49.10	1086	468	28	465
U1M199	3AHX:A	40.87	966	453	6	440
R0E6N0	1GNX:A	44.68	909	479	18	479
A0A0Q6TWU7	1GNX:A	42.83	893	479	18	476
U1M1A3	5DT5:A	84.16	2070	471	24	465
C6R4W1	1QOX:A	35.23	597	449	5	449
A0A0Q7X946	3TA9:A	48.77	1096	458	10	450
A0A0Q7Y9N4	4HZ6:A	50.34	1129	444	5	437
U4EAX8	3TA9:A	47.50	1079	458	12	450
A0A0M8K9L7	1OD0:A	52.85	334	468	344	466
A6W3B1	3CMJ:A	46.92	1055	465	24	460
A0A0P6YGK0	3TA9:A	49.78	1161	458	10	452
I0IGW5	1OD0:A	45.23	1025	468	25	465
W9GTD4	1GNX:A	48.23	1078	479	1	477
C7Q8K7	1NP2:A	53.42	1040	436	7	428
A0A090T3G1	1E4I:A	43.90	490	447	12	215
A0A090T285	1QOX:A	35.29	90	449	256	317
A0A0Q8UIK4	3TA9:A	45.45	1003	458	13	450
S9QDH2	1OD0:A	48.42	1056	468	27	466
U2URX1	1UYQ:A	34.83	630	447	5	441
C7Q825	1GNX:A	63.76	1501	479	17	470
A0A090SW15	3AHX:A	42.67	924	453	4	441
A0A090T7F7	1NP2:A	57.89	383	436	7	120
A0A090TL25	4B3K:A	43.73	755	479	2	328
R8V3Y2	3TA9:A	40.69	933	458	13	451
A0A101U6D1	1GNX:A	64.76	1483	479	12	477
A0A0A6UCN6	1GNX:A	57.86	1313	479	16	472
C7PXI2	1GNX:A	53.61	1143	479	15	466

A7HN90	3TA9:A	55.02	1208	458	13	450
D0KKZ7	4PTV:A	42.79	999	452	6	443
A0A101S8C8	1GNX:A	64.83	1613	479	9	479
A0A0M3AQ52	1OD0:A	42.60	950	468	21	466
S9QAM8	4PTV:A	51.40	1179	452	28	450
A0A101TH24	3CMJ:A	43.60	903	465	17	458
A0A090SYM9	4B3K:A	54.55	234	479	381	457
V6FCW6	1QOX:A	34.47	607	449	5	449
A0A090RZQ3	4B3K:A	31.37	156	479	1	100
A0A0A6XEE8	3W53:A	44.98	923	506	46	502
A0A090TQ63	1OD0:A	48.00	231	468	371	465
A0A0A5G8H5	1QOX:A	59.33	1439	449	5	449
A0A0A6UC18	1GNX:A	52.63	1075	479	18	466
A0A090RRD9	4B3K:A	45.24	1072	479	2	461
A0A124HZ19	1NP2:A	51.90	982	436	6	430
A0A0P6XSE8	3TA9:A	53.93	1253	458	13	455
A0A101S675	1GNX:A	65.11	1476	479	13	477
A0A086ZGS5	1VFF:A	33.10	567	423	3	412
A0A0F7P351	1OD0:A	43.72	1022	468	21	462
A0A117R0S1	3WH5:A	55.96	1234	457	6	440
A0A0N0KK57	4PTV:A	45.23	978	452	10	448
J4XBG7	1UYQ:A	34.32	617	447	4	441
A0A090SZU6	3AHX:A	38.13	749	453	6	440
A0A090SBY8	1QOX:A	39.48	812	449	5	442
A0A090SN13	4B3K:A	43.13	801	479	101	470
A0A0A6UGB3	3W53:A	49.56	1065	506	48	496
A0A101U0K9	1GNX:A	66.88	1625	479	12	477
A0A0C9NHL2	1OD0:A	45.74	1008	468	26	466
A0A0Q8USD4	1GNX:A	50.22	1023	479	16	469
D0KIH4	1QOX:A	39.52	777	449	5	442
B9MNR1	3AHX:A	52.40	1239	453	4	444
A0A0H4KYB8	1OD0:A	49.31	1073	468	26	453
H3MMQ0	3TA9:A	45.76	1049	458	13	450
A0A0H5NKN5	1VFF:A	28.85	393	423	9	408
I0BJW8	1BGG:A	59.96	1495	448	1	446
A0A0Q7KM67	3WH5:A	56.92	1260	457	6	449
A0A0Q7KJD1	1GNX:A	55.37	1202	479	1	472
A0A099KSH6	2O9P:A	42.40	981	454	18	451
A0A0Q7L9U0	3WH5:A	45.20	906	457	1	444
G8SC15	1GNX:A	52.81	1058	479	10	469
D9XZ76	1GNX:A	67.30	1649	479	9	477
D3AL50	1OD0:A	44.87	1029	468	30	465
D9R0D8	1OD0:A	42.76	930	468	27	464
L8P7A3	1GNX:A	67.82	1623	479	17	477
G8S7I1	1GNX:A	53.96	1114	479	22	474
I0BCR1	2O9P:A	52.77	1117	454	76	454
A0A0Q7L731	1GNX:A	53.56	1112	479	17	470
D9Y101	3WH5:A	56.33	1217	457	6	446
A0A0Q7L146	1GNX:A	66.81	1572	479	14	475
I7L7J4	4B3K:A	57.45	308	479	1	94
I7KI36	4B3K:A	63.14	1267	479	95	461
A0A151BU23	1GNX:A	56.24	1202	479	18	470
L8PCP8	1NP2:A	50.11	921	436	8	430
G8SB50	1GNX:A	46.45	892	479	16	473
A0A087DF01	1GNX:A	51.17	1175	479	16	476
A0A0X4JTP1	1NP2:A	47.56	947	436	8	424
D9Y1S6	1NP2:A	51.02	910	436	6	426

A0A099L2C3	3TA9:A	44.42	1020	458	12	450
G8S7Q9	1GNX:A	57.47	1340	479	1	472
D3AAZ8	1OD0:A	47.65	1023	468	28	465
I7KHU7	1QOX:A	32.93	432	449	145	449
I7LS21	1QOX:A	42.89	937	449	5	442
A0A0K8QB92	3CMJ:A	47.22	1111	465	23	459
D3ADZ0	1QOX:A	45.61	1030	449	5	449
A0A0Q7KEA9	1GNX:A	71.03	1725	479	15	477
A0A0Q8NH52	1OD0:A	43.89	1021	468	24	461
I7LPV0	1BGA:A	63.33	463	447	5	124
A0A151C116	1GNX:A	59.23	1339	479	7	470
D9XPF0	1GNX:A	69.49	1647	479	7	475
R8TNK1	4PTV:A	40.91	940	452	7	445
A0A0Q9E6X9	1OD0:A	45.15	1031	468	24	462
A0A0D8IVH6	1QOX:A	46.89	1015	449	5	443
A0A0F0EY28	3W53:A	54.09	1285	506	32	505
A0A0W7WMQ7	1OD0:A	45.85	1029	468	21	453
A0A0K1JFK2	3WH5:A	48.04	1050	457	5	444
W3Y128	1UYQ:A	32.42	596	447	3	441
A0A0M2QQ93	3TA9:A	47.32	1070	458	13	450
A0A150N7F7	1BGA:A	63.04	180	447	5	50
Q608B9	3TA9:A	50.00	1160	458	8	451
A0A0K1JFX1	1NP2:A	45.74	916	436	6	427
A0A0D8HP97	3CMJ:A	30.39	410	465	28	463
A0A0N0SPK0	3WH5:A	55.88	1215	457	6	446
A0A0D8HXD7	1NP2:A	49.66	1026	436	7	427
J2JX81	1NP2:A	51.66	983	436	7	432
A0A0U4W872	3TA9:A	44.52	991	458	12	449
A0A0M8U5W7	1GNX:A	72.17	1717	479	18	476
Z5DUF2	3TA9:A	46.31	1054	458	13	450
L1KDW9	4PTV:A	44.65	989	452	1	430
A0A150NE90	1UYQ:A	34.30	699	447	6	441
I0G3J4	3WH5:A	44.55	910	457	6	441
A0A0K1JLL6	1GNX:A	55.46	1180	479	13	467
A0A0M3H2D5	3TA9:A	45.41	1053	458	13	450
A0A095ALE8	1OD0:A	49.77	1099	468	28	465
A0A010QHQ5	2JIE:A	44.59	1025	454	10	451
A0A0Q5LAF9	3W53:A	54.56	1249	506	44	501
A0A089Y711	3AHX:A	37.83	849	453	10	440
A0A0M2H901	1VFF:A	32.72	549	423	1	402
A0A0M2HKV3	3W53:A	53.93	1255	506	44	501
A0A0M2HCX3	1VFF:A	34.22	575	423	3	408
A0A0M2H0T8	3W53:A	46.93	988	506	35	503
D2BKE1	4B3K:A	55.19	1435	479	1	461
D1BC10	3W53:A	53.26	1234	506	43	505
A0A0Q5L579	1GNX:A	62.66	1417	479	16	470
B0C9B0	1OD0:A	45.19	1025	468	28	465
I7EUV9	3AHX:A	42.57	966	453	6	445
C9D1B8	1OD0:A	48.44	601	468	26	250
A0A136H420	1OD0:A	46.41	1071	468	25	466
D6B2A8	1GNX:A	68.97	1633	479	18	479
D1BCW8	1GNX:A	61.03	1413	479	14	477
A0A0L8NR76	1GNX:A	50.43	984	479	10	472
A0A126PGE0	1OD0:A	42.51	932	468	28	463
A0A059VW33	1NP2:A	52.60	1010	436	8	428
A0A095AS65	1VFF:A	32.62	593	423	9	404
M1MT62	3AHX:A	44.09	995	453	4	442

A0A143CBS3	1GNX:A	57.33	1192	479	17	467
A0A143C0M6	1GNX:A	68.31	1665	479	12	477
D2BKB9	3AHX:A	34.10	597	453	4	440
A0A0M2HC25	1VFF:A	36.94	191	423	295	404
A0A0M2HAH2	1VFF:A	36.84	297	423	9	156
A0A143BYV7	1NP2:A	50.56	915	436	8	430
A0A010PLT1	2O9P:A	42.44	968	454	9	454
A0A0L8N1U2	1GNX:A	69.43	1666	479	16	477
A0A0M4MCU7	1QOX:A	44.35	971	449	5	441
A0A0T7NNF6	1UYQ:A	30.00	560	447	5	441
A0A0P1JCR9	1OD0:A	44.57	1004	468	21	461
A0A0Q9RA48	3TA9:A	44.20	946	458	8	452
A0A0M4CRD3	1OD0:A	48.31	1064	468	26	465
A0A094IRZ0	3CMJ:A	47.39	995	465	30	456
M5A7J9	2JIE:A	45.11	1072	454	12	454
A0A0T9S6E5	1UYQ:A	29.38	557	447	5	441
A0A100HNZ0	3WH5:A	54.52	1214	457	5	441
A0A0Q9QQN6	1GNX:A	60.43	1431	479	13	477
F7UBG1	1QOX:A	46.03	1032	449	5	443
A0A117DMM0	1NP2:A	49.89	935	436	1	423
A0A0Q9R0X3	3W53:A	66.38	1646	506	40	503
F3ZIC4	1GNX:A	70.17	1729	479	3	477
A0A0P0D6B0	3TA9:A	41.50	954	458	13	451
A0A0B5DL02	1GNX:A	87.68	2183	479	1	479
A0A095B600	1OD0:A	49.10	1089	468	28	465
A0A0D0K9U3	1OD0:A	42.26	960	468	32	463
H5XJU1	3W53:A	54.33	1250	506	43	502
W8I1S6	1QOX:A	47.05	1013	449	5	441
A0A0F4FKU4	1QOX:A	46.95	1047	449	5	444
A0A101M6I5	3TA9:A	45.45	1026	458	12	449
E8U905	3WH5:A	54.67	1177	457	6	440
A0A0B5DKC1	3WH5:A	56.33	1249	457	6	446
A0A087AN75	1VFF:A	34.14	570	423	4	405
H3ZEG5	3WH5:A	48.25	994	457	8	433
A0A0B5DC15	1GNX:A	66.45	1612	479	16	477
A0A024J0W6	1QOX:A	46.03	1026	449	5	443
C8WXU5	3TA9:A	57.47	1389	458	13	453
I8TQ36	3AHX:A	42.55	943	453	4	440
A0A0A2WJJ7	1OD0:A	52.68	1201	468	21	465
A0A0G9L7U4	3AHX:A	42.98	966	453	4	441
A0A087AM90	1GNX:A	54.15	1157	479	17	470
A0A0B5DAL1	1NP2:A	50.00	908	436	6	430
F3ZH12	3WH5:A	54.52	1237	457	6	446
C1CXP6	4PTV:A	48.89	1202	452	1	452
A0A0Q9Q5T4	1GNX:A	51.87	1029	479	18	470
D4ZIJ5	2JIE:A	42.12	963	454	9	451
H1QQV6	1NP2:A	50.67	929	436	6	426
H1QA68	3WH5:A	55.43	1212	457	6	446
A0A081EBT3	1GNX:A	70.70	1730	479	7	477
M1ZM06	3TA9:A	56.63	1201	458	13	404
A0A085EQI1	3WH5:A	49.55	1083	457	5	449
A0A081EFN3	1GNX:A	57.59	1308	479	6	475
H1QRE5	1GNX:A	65.74	1618	479	9	477
M1Z429	4PTV:A	55.98	1323	452	5	446
A0A081EFM8	1GNX:A	70.47	1680	479	14	477
A0A0Q9PTK7	3W53:A	55.05	1317	506	45	498
A0A081EGY5	1GNX:A	78.59	1939	479	1	479

H0FZK7	1QOX:A	47.51	1040	449	5	444
H1Q7A1	1GNX:A	90.19	2224	479	1	479
A0A081EFM7	1QOX:A	48.55	1103	449	5	443
A0A081ECE4	3W53:A	48.69	994	506	46	502
L0NI99	1QOX:A	46.03	1018	449	5	443
A0A085FY80	3WH5:A	51.81	1139	457	6	443
A0A084SEI6	3TA9:A	50.00	1199	458	13	456
A0A0Q6ZTG4	1QOX:A	46.71	1032	449	5	439
W6TLA4	1QOX:A	39.77	853	449	5	443
S6EVS8	4B3K:A	55.97	789	479	215	456
V4IND8	1GNX:A	69.61	1642	479	18	479
B8CL86	4PTV:A	45.18	1020	452	10	445
A0LR48	1GNX:A	59.22	1412	479	16	476
S6EUE8	3AHX:A	34.10	599	453	4	440
S6EVI6	1UYQ:A	31.19	582	447	4	441
V4KKK5	1GNX:A	76.62	1900	479	1	477
A0A094JYA8	3TA9:A	52.68	1218	458	13	457
V6T323	5DT5:A	69.86	1718	471	25	461
A0A0K9X890	1GNX:A	69.40	1622	479	17	479
A0A062Y195	3TA9:A	49.09	1115	458	13	450
S6F9A3	5FOO:A	56.37	646	480	2	205
I1D3B4	3W53:A	54.47	1250	506	45	502
S6FR19	3AHX:A	42.09	882	453	6	441
V4IGK2	3WH5:A	55.58	1210	457	6	434
A0A091C594	1QOX:A	33.22	329	449	176	449
A0A031GUM1	4PTV:A	48.87	1076	452	7	444
A0A074LBK9	1BGG:A	97.54	2386	448	1	448
A0A0M1QAN2	2O9P:A	90.60	2225	454	8	454
L8K5M4	1OD0:A	44.17	1036	468	21	462
A0A0A6DXI2	3TA9:A	48.87	1075	458	13	450
A0A0S2P088	1GNX:A	69.10	1715	479	1	477
A0KK20	1QOX:A	41.42	883	449	5	445
A0A150YZ37	1BGG:A	97.77	2388	448	1	448
A0A0F0FYZ1	1BGG:A	99.78	2420	448	1	448
A0A136JUN2	1VFF:A	35.99	634	423	28	408
A0A0S2P002	1GNX:A	67.82	1628	479	16	477
G7VTE8	2O9P:A	89.04	2212	454	8	454
A0A0S2PBQ0	3TA9:A	50.89	1177	458	13	450
A0A0S2P9U1	3WH5:A	55.88	1236	457	6	446
A0A0T0MLA0	1GNX:A	59.70	1408	479	18	478
A0A074L9I1	2O9P:A	90.38	2226	454	8	454
A0A0M1Q006	1BGG:A	97.54	2374	448	1	448
A0A150YZH1	2O9P:A	91.05	2231	454	8	454
Q07HP5	3TA9:A	43.08	936	458	15	455
B1FT15	3TA9:A	46.00	1062	458	10	454
G7W3A7	1BGG:A	95.54	2347	448	1	448
A0A0F6F044	2O9P:A	98.88	2386	454	8	454
A0A0S2NZV3	1NP2:A	50.34	920	436	8	430
A0A0N0UI50	1BGG:A	75.39	1942	448	1	447
A0A0H2NBH8	2O9P:A	45.37	1056	454	18	449
A0NVK9	1QOX:A	48.18	1046	449	5	442
M8AF35	1OD0:A	44.35	1037	468	25	466
W6RG33	1QOX:A	47.06	1050	449	5	444
K0D5L8	2JIE:A	42.73	989	454	10	454
A4FKW1	1GNX:A	51.08	1104	479	15	477
A4FH46	1VFF:A	28.16	378	423	9	402
A0A0R3DLK6	3WH5:A	43.69	904	457	2	441

C2LSG6	1UYQ:A	32.42	596	447	3	441
A0A0M8VJD7	3WH5:A	53.55	1173	457	11	446
A0A0P7XDC1	1OD0:A	44.19	846	468	70	453
A0A0H4WPC8	3TA9:A	50.33	1229	458	13	456
C3JLK0	3ZJK:A	49.11	1021	431	7	430
H7GEV0	1NP2:A	97.70	2211	436	1	435
Q9A6F8	1GNX:A	43.32	896	479	18	479
A0A0M6Y187	1QOX:A	48.51	1039	449	5	439
A4F947	1NP2:A	50.35	958	436	6	430
A0A0M8VPF2	1GNX:A	53.33	1103	479	18	474
A0A0N0T1Q2	1NP2:A	49.66	899	436	8	430
A0A0M8W1N2	1GNX:A	81.03	1989	479	14	477
U7G846	1QOX:A	47.73	1034	449	5	442
K0D3E2	2O9P:A	41.27	961	454	13	451
A0A0M9ZF02	1GNX:A	66.74	1636	479	7	477
J1ACA0	1OD0:A	43.67	1021	468	24	461
A0A0W1D8C6	1OD0:A	49.10	1095	468	28	465
A0A109QK50	1NP2:A	97.91	2188	436	1	430
A0A0A0JPW7	1GNX:A	56.13	1194	479	18	470
A0A0F0LL99	3W53:A	54.18	1283	506	36	502
E8VZV1	3WH5:A	45.20	910	457	1	444
A0A0S8IDN0	4PTV:A	56.05	1361	452	2	446
A0A0L8LF50	1GNX:A	67.65	1589	479	5	476
A0A0F0KW36	1VFF:A	33.25	561	423	5	412
A0A0Q6G1R6	1GNX:A	60.17	1411	479	14	477
A0A0F0LCC5	1OD0:A	47.26	1058	468	14	465
A0A0N9I2T3	3W53:A	52.49	1204	506	43	502
A0A0L8JAM5	1GNX:A	66.60	1602	479	2	477
Q167E6	1OD0:A	43.78	975	468	21	453
A0A0H4C1P7	3WH5:A	57.01	1254	457	6	446
A0A0J7ZD37	1GNX:A	62.73	1466	479	2	479
A0A0N7F2D9	3WH5:A	47.74	996	457	6	444
F3BMW9	2O9P:A	41.88	963	454	13	449
A0A0Q7XSS8	1QOX:A	47.83	1056	449	5	439
A0A0J7Z7J6	1GNX:A	66.45	1616	479	12	477
A0A0L8JI51	1GNX:A	73.28	1746	479	14	476
A0A0P0CRR6	1OD0:A	44.09	979	468	26	461
A0A0J8BU96	1NP2:A	50.67	924	436	8	430
A0A0F0LXG2	1NP2:A	48.17	894	436	7	431
A0A0V8IVW0	3W53:A	47.00	974	506	46	503
A0A081MJ60	1QOX:A	45.54	1035	449	5	439
A0A0H4C4H9	1GNX:A	68.09	1661	479	14	479
E0PFZ9	1QOX:A	32.07	552	449	3	443
C7BJX2	1E4I:A	34.14	644	447	4	441
A0A0F0L6Y0	3W53:A	55.11	1301	506	45	502
A0A0F0LIL6	1VFF:A	36.06	644	423	4	405
A0A142Z2D1	4PTV:A	46.24	1050	452	1	438
A0A0N9ID20	1GNX:A	53.75	1212	479	6	472
F3BJ64	3TA9:A	42.70	989	458	12	452
A0A0M0WIV8	3AHX:A	43.10	968	453	4	441
A0A0L8JEN6	1GNX:A	51.17	1012	479	8	470
E0PDF8	1OD0:A	38.98	809	468	21	465
A0A067LG20	1GNX:A	60.30	1385	479	16	477
A0A081MJA7	1OD0:A	42.63	990	468	25	466
A0A0H4CJ37	1GNX:A	56.83	1144	479	18	467
E8W810	1GNX:A	70.21	1717	479	11	477
A0A0H4CKJ0	1NP2:A	50.11	890	436	12	426

E8W143	1GNX:A	54.00	1120	479	17	470
E8W9K4	1GNX:A	56.25	1203	479	7	467
A0A0F0LQJ8	1GNX:A	59.62	1324	479	17	478
A0A0Q7VW69	3W53:A	52.77	1228	506	44	501
A0A0L8L9U3	3WH5:A	54.55	1208	457	6	444
A0A0F0K8M2	1NP2:A	49.66	919	436	10	431
A0A0Q6G2C2	3W53:A	53.35	1244	506	40	505
G5ZW36	1OD0:A	45.06	998	468	21	453
A0A0Q7W0B1	4PTV:A	47.01	1068	452	2	445
A3YHU8	3TA9:A	46.49	1047	458	12	450
T0IT97	1OD0:A	44.59	972	468	28	466
K7S4S9	3W53:A	46.87	1072	506	41	499
I9KXA9	4PTV:A	32.08	585	452	7	444
A0A0D6B1Z9	3TA9:A	46.02	972	458	13	443
A0A0U5JDH6	3TA9:A	46.67	1075	458	13	458
H0TI04	1OD0:A	43.97	958	468	25	466
A0A0T5ZXI6	1VFF:A	38.98	727	423	4	408
A0A0Q7WMB8	3WH5:A	51.37	1127	457	6	440
A0A0D8KD33	1OD0:A	44.12	1036	468	25	462
E3CIV2	1BGG:A	32.70	615	448	6	441
H0TP06	3TA9:A	45.29	1024	458	17	450
A0A0U0XYC0	1E4I:A	28.24	370	447	9	419
A0A101RD42	1NP2:A	50.34	947	436	8	426
A0A100JTN4	1VFF:A	35.90	612	423	4	408
A0A060V7Z8	3TA9:A	47.10	1059	458	13	450
A0A0U1AVQ1	1UYQ:A	31.48	552	447	8	441
A0A0Q9RIY6	4PTV:A	43.89	986	452	4	444
A0A0U1BB08	1E4I:A	28.00	364	447	9	419
K9TDJ7	3TA9:A	51.12	1169	458	13	456
C7IQT1	4PTV:A	58.35	1405	452	5	450
A0A0U1A3Y3	1E4I:A	28.00	364	447	9	419
X8ITU6	1UYQ:A	32.20	585	447	3	441
A0A0U1B050	4B3K:A	49.57	1199	479	3	459
A0A0J6DJV4	1GNX:A	59.79	1410	479	7	478
A0A100JPU0	1NP2:A	50.45	954	436	8	431
A0A101RI91	3WH5:A	55.93	1239	457	6	449
A0A0U1ASY1	1UYQ:A	32.08	582	447	5	441
A0A0G3V5H1	1VFF:A	28.37	401	423	5	406
A0A0U0WYT0	1E4I:A	28.24	370	447	9	419
D1BX60	3W53:A	54.66	1258	506	44	502
A0A0D7X402	2O9P:A	90.16	2222	454	8	454
A0A0D7X839	1BGG:A	95.09	2336	448	1	448
A0A124C423	1VFF:A	35.63	622	423	2	404
A0A0J6GGH7	3CMJ:A	46.55	1000	465	25	463
A0A0U1AB91	1OD0:A	38.19	749	468	25	468
A0A0U0Z2D9	1VFF:A	37.91	243	423	260	411
A0A100JPB7	1GNX:A	53.09	1179	479	16	477
A0A0U1AD04	3TA9:A	41.63	898	458	13	451
A0A086GLW0	1GNX:A	67.45	1661	479	12	477
A0A086GRE0	1NP2:A	49.55	937	436	8	433
I0JQY5	1BGG:A	56.28	1442	448	1	445
A0A100JXZ0	1GNX:A	90.81	2220	479	1	479
D3T6M2	5DT5:A	55.91	1403	471	24	461
A0A0U1AT11	1QOX:A	34.02	613	449	5	443
J0VWU7	1QOX:A	47.51	1049	449	5	444
A0A100JTZ6	1GNX:A	67.02	1644	479	12	477
A0A0U1B430	3AHX:A	33.12	556	453	5	445

D2EH44	3TA9:A	32.47	543	458	12	450
A0A101R2P9	1GNX:A	68.39	1650	479	16	479
A0A023X547	1GNX:A	56.92	1236	479	16	470
A0A0C1BUB6	1NP2:A	47.72	934	436	8	431
A0A0G3V6A1	1VFF:A	32.16	479	423	4	384
D1BWB0	1GNX:A	58.25	1352	479	15	477
A0A0U1BE00	1E4I:A	33.51	256	447	9	181
S9RDB0	1OD0:A	44.59	1007	468	21	462
D3FS09	1BGG:A	62.86	1600	448	1	446
A0A0N0MMB8	1GNX:A	70.97	1693	479	16	477
A0A076LLP4	3TA9:A	43.26	1027	458	13	452
A0A0X3W9T3	1NP2:A	53.24	1002	436	8	430
A0A0T2IK19	1GNX:A	59.65	1293	479	18	468
A0A0Q7TBR9	1QOX:A	42.83	921	449	3	447
L8ENY0	1GNX:A	68.83	1675	479	9	477
F9HL45	1UYQ:A	33.48	608	447	5	441
A0A0N1NI34	1VFF:A	33.17	543	423	5	408
N0DY37	1GNX:A	51.87	1094	479	17	470
A0A0Q6SHI3	3TA9:A	48.68	1091	458	10	457
A0A0X3W714	1GNX:A	71.49	1724	479	16	477
A0A0Q6SJH3	4HZ6:A	50.93	1135	444	5	434
A0A0C7NKR7	1OD0:A	67.42	1632	468	24	467
A0A0V0PQ32	2O9P:A	52.61	1328	454	14	454
L8EVL9	3W53:A	47.52	947	506	50	491
E8N3Y4	3TA9:A	54.36	1222	458	13	455
A0A0N1GF50	1NP2:A	50.22	923	436	7	434
D1APK8	4B3K:A	41.47	1037	479	1	462
D1APC8	4B3K:A	45.95	1133	479	3	459
A0A0G8EM50	3AHX:A	42.24	934	453	4	441
A0A0Q5CQA0	1OD0:A	43.79	1020	468	25	461
A0A0F5R775	1QOX:A	59.87	1499	449	4	449
U6SRQ3	1BGG:A	62.64	1596	448	1	446
A0A0D5VJL0	4PTV:A	47.83	1064	452	6	437
A0A0G8CCQ6	3AHX:A	42.03	933	453	4	441
D1ANN6	4B3K:A	44.66	1077	479	3	459
A0A0G8EP62	3AHX:A	33.89	614	453	1	440
A0A068NKR9	1QOX:A	44.70	970	449	11	443
A0A0Q7Z0P8	3WH5:A	47.90	998	457	6	444
D1AQC4	3AHX:A	40.76	878	453	6	445
T0HTG3	3WH5:A	44.07	920	457	6	444
C4VNB6	3CMJ:A	29.68	567	465	28	462
C4VL63	1QOX:A	33.74	601	449	4	449
I3AIN0	3TA9:A	44.23	1028	458	12	450
C0FVV0	1OD0:A	45.21	1042	468	28	465
B5GET9	1GNX:A	69.96	1720	479	3	477
J2LTU4	3TA9:A	47.10	1065	458	13	450
A0A085I3Q9	3CMJ:A	37.53	761	465	25	465
J5MQG5	1QOX:A	46.83	1049	449	5	444
A0A0Q9ND22	3W53:A	48.07	961	506	46	503
A0A0S9DWR5	1GNX:A	60.97	1395	479	13	477
A0A0D1C9J4	3W53:A	58.60	1383	506	40	502
B5GF53	3WH5:A	54.98	1242	457	6	446
A0A0D1A767	1OD0:A	42.73	924	468	32	462
A0A085IFG4	3TA9:A	46.21	1051	458	13	450
K8GQK7	3WH5:A	44.03	896	457	1	444
A0A0K8P3N6	3TA9:A	48.75	1144	458	13	452
A0A0D1A4S8	3TA9:A	41.58	932	458	13	451

K6YVQ1	3TA9:A	45.12	1033	458	15	455
E8N6X0	3W53:A	52.51	1251	506	39	500
E0NHM3	1UYQ:A	30.83	567	447	5	441
A5KVQ8	2O9R:A	45.19	1094	452	8	447
W4BBB3	1BGG:A	75.39	1936	448	1	447
A0A0S9DNH2	3ZJK:A	47.37	933	431	7	423
K2GR21	1OD0:A	46.17	996	468	24	453
B8DVE3	1GNX:A	53.70	1175	479	15	467
B1KHD2	3TA9:A	43.75	995	458	10	450
A0A0S2FRN1	1OD0:A	51.68	1170	468	26	465
A0A023XM13	3WH5:A	45.23	910	457	6	441
Q3J2E9	4PTV:A	45.24	992	452	1	430
C4RCW7	1GNX:A	57.17	1270	479	16	475
A0A0T5VRL9	1OD0:A	40.35	865	468	24	462
D7VE52	4B3K:A	58.17	1432	479	1	459
A0A0U3N3Y9	3WH5:A	49.43	1054	457	6	444
A0A023XS67	3WH5:A	44.87	911	457	6	440
C4RJD4	1GNX:A	65.73	1517	479	17	476
A0A0U3L7K8	3TA9:A	48.42	1086	458	13	451
F7NRC1	3TA9:A	43.86	1028	458	12	451
D7VDX2	3TA9:A	32.64	610	458	13	451
A0A085TXE9	1OD0:A	46.01	1035	468	21	453
B5ZQU8	1QOX:A	47.96	1054	449	5	444
A0A099XYQ1	4GXP:A	49.36	1211	467	10	466
A0A104NM65	3TA9:A	45.54	1039	458	12	436
A0A0U1NND8	1OD0:A	44.42	963	468	28	453
A0A0Q5N8P0	1OD0:A	41.52	892	468	28	463
A0A074JPD3	1OD0:A	45.49	1063	468	16	466
V6KM89	3WH5:A	57.34	1251	457	6	440
A0A087AD30	1GNX:A	53.49	1249	479	16	479
V9HHQ1	3AHX:A	43.68	983	453	4	445
A0A0H2ZM73	1BGA:A	31.50	579	447	5	441
W0IYZ5	1E4I:A	41.68	873	447	1	441
F2G9T0	2JIE:A	43.21	982	454	10	454
A0A073CLU2	1OD0:A	25.57	278	468	72	444
V6KP04	1GNX:A	76.92	1876	479	9	476
J0KUK3	1QOX:A	47.62	1048	449	5	443
W0J296	1OD0:A	43.05	940	468	21	466
W0J0Z0	1OD0:A	43.02	936	468	28	464
C6CZG8	1OD0:A	42.83	987	468	27	464
U6FJR1	2E9L:A	35.71	55	469	288	314
K2N268	1OD0:A	44.59	998	468	21	462
A0A0F5VQF2	3W53:A	46.52	930	506	45	503
S5V2I9	1NP2:A	50.67	926	436	8	430
A0A139C0C4	3CMJ:A	46.06	980	465	30	452
A0A0F5W8Q8	3WH5:A	54.75	1215	457	6	446
K4KIJ3	4PTV:A	47.72	1086	452	7	444
A0A0F5VT61	1GNX:A	66.38	1624	479	12	477
S5UVC9	1GNX:A	68.68	1656	479	16	477
V6KM14	1GNX:A	55.94	1160	479	7	467
S5VXZ2	3WH5:A	54.75	1214	457	6	446
W0J651	1E4I:A	40.85	903	447	3	441
Q28R01	1OD0:A	42.56	938	468	26	459
A0A0D6VK56	1GNX:A	56.58	1186	479	18	472
A0A109CDY6	1OD0:A	44.57	1035	468	25	462
F5XGJ5	1VFF:A	32.93	561	423	9	406
A0A101IMH3	4PTV:A	52.24	1226	452	2	445

R4G036	3TA9:A	52.55	1214	458	13	456
A0A0D6VJ04	3WH5:A	56.79	1271	457	6	446
A0A0Q8B8V1	1OD0:A	41.78	961	468	25	466
A0A0D6V8N6	1GNX:A	69.15	1669	479	11	477
Q47PF5	1GNX:A	55.02	1145	479	18	470
A0A0D6VAY7	3WH5:A	45.51	906	457	6	448
A3DFD0	1VFF:A	38.02	751	423	1	416
F5XLS1	1VFF:A	35.65	606	423	3	406
G8AWD9	3TA9:A	43.34	984	458	12	450
G8AWC9	3TA9:A	44.67	1003	458	8	456
W7SV29	4HZ6:A	45.35	988	444	2	437
Q47RE2	1GNX:A	58.89	1373	479	12	474
A0A0Q6UY27	1QOX:A	47.71	1050	449	5	438
W7TCZ5	1GNX:A	54.04	1211	479	6	472
A0A0S7YLD5	1QOX:A	46.89	1011	449	5	443
A0A0N7FUC0	3W53:A	44.69	917	506	46	503
S9SHG7	3TA9:A	46.17	1007	458	13	455
A0A0F4JRE9	1GNX:A	71.73	1722	479	5	476
W7TGA6	3W53:A	54.31	1243	506	39	502
G8ARQ1	4PTV:A	45.54	1049	452	7	452
G8AWE1	3TA9:A	42.25	989	458	10	454
A0A0D6UP56	1GNX:A	66.53	1598	479	9	476
A0A0N8GNU0	3TA9:A	52.93	1278	458	9	452
I0HIP5	1GNX:A	59.00	1350	479	14	472
A0A124H6G3	3W53:A	47.10	926	506	45	491
I0HF78	3W53:A	50.22	1098	506	49	491
A0A0P6XFQ8	3WH5:A	59.17	1408	457	6	440
A0A117RCC8	1GNX:A	68.40	1713	479	1	479
W1E6M2	3TA9:A	47.32	1071	458	13	450
A0A101T0Q8	1NP2:A	50.11	881	436	12	430
A0A126Z4Y4	1GNX:A	52.65	1104	479	18	468
W1E5P8	5FOO:A	44.83	1064	480	4	464
A0A117PCI5	3WH5:A	55.28	1203	457	6	440
A0A0H3NSP1	4B3K:A	46.41	1113	479	3	459
A0A101TCI3	3WH5:A	56.11	1237	457	6	446
A0A0N0SCB5	1GNX:A	64.71	1502	479	16	474
W1E580	4B3K:A	42.97	834	479	96	470
A0A0G1YZ56	1VFF:A	38.89	221	423	7	107
A0A101SZN6	1GNX:A	69.57	1665	479	12	478
E1UTL1	4B3K:A	48.81	1180	479	3	459
I8R1J8	5DT5:A	54.98	1396	471	24	463
A0A0M4GBU3	1E4I:A	59.64	1488	447	5	444
A0A0H3NVV8	4B3K:A	42.64	1026	479	3	462
E1UKU7	3AHX:A	39.49	850	453	6	445
E1UTQ7	1UYQ:A	34.35	659	447	4	441
A0A0P6XT49	4PTV:A	51.00	1203	452	2	446
A0A0H3NTL2	4B3K:A	48.91	1177	479	3	459
A0A0H5B9M3	3CMJ:A	43.45	977	465	27	458
A0A124GVZ2	1GNX:A	67.52	1634	479	12	477
A0A0T8PG43	1UYQ:A	33.05	592	447	5	441
A0A0T8BU07	4B3K:A	48.81	1181	479	1	459
A0A0U0GY98	1UYQ:A	30.00	400	447	85	441
A0A0T8QFQ2	1UYQ:A	33.05	592	447	5	441
A0A0Z5Q194	1UYQ:A	33.05	590	447	5	441
A0A100CLM4	5FOO:A	47.63	1149	480	1	460
A0A0T8VXI9	4PTV:A	37.41	194	452	323	445
A0A0T8WD98	4B3K:A	52.60	551	479	268	459

F9EGJ8	1UYQ:A	33.12	605	447	1	446
I1AVY5	1OD0:A	45.77	1022	468	21	453
A0A0T8BNH2	1UYQ:A	33.05	596	447	5	441
A0A0T9GUQ6	5FOO:A	45.12	381	480	1	160
A0A0Y0C405	1UYQ:A	33.05	594	447	5	441
A0A0I5H012	1UYQ:A	33.05	591	447	5	441
A0A0T8N3C8	1BGG:A	34.29	421	448	1	300
A0A139MY13	4B3K:A	48.16	1152	479	1	459
A0A0T8BKP6	4B3K:A	65.36	1683	479	3	461
A0A0U0KAV4	4B3K:A	48.37	1165	479	1	459
A0A0T8CHH2	1UYQ:A	32.83	589	447	5	441
A0A0U0CTK4	4B3K:A	47.94	1166	479	1	459
A0A0T8BX03	1UYQ:A	33.05	592	447	5	441
A0A086Z2F7	1VFF:A	29.22	484	423	1	405
W6LTU9	3TA9:A	51.46	1175	458	9	452
A0A0U0IRN4	4B3K:A	47.62	209	479	258	341
D4LC32	1OD0:A	43.46	973	468	27	465
A0A0T8B005	1UYQ:A	33.05	599	447	5	441
A0A098LEE1	1OD0:A	44.12	999	468	26	463
A0A0T8QCX9	4B3K:A	48.22	934	479	95	459
A0A0F2CN10	5FOO:A	48.05	1165	480	1	460
A0A111XY92	1UYQ:A	33.05	596	447	5	441
A0A0Z6YG58	1UYQ:A	33.05	593	447	5	441
A0A0T8AC09	3TA9:A	31.34	266	458	199	452
A0A0T8SM74	1UYQ:A	32.14	581	447	5	441
A0A0X9B453	4B3K:A	47.95	1155	479	1	459
A0A0Y1UNU7	3AHX:A	34.88	554	453	2	440
G0AX44	3CMJ:A	42.07	974	465	15	460
A0A0U0KVE4	1BGG:A	34.24	407	448	1	284
A0A0U0IRU8	5FOO:A	50.00	215	480	1	83
A0A0T7ZHF0	1UYQ:A	33.05	596	447	5	441
A0A100H6V6	3CMJ:A	39.29	92	465	413	462
A0A0T8B4P4	1UYQ:A	33.05	591	447	5	441
A0A0T8EH97	1UYQ:A	33.05	600	447	5	441
A0A139N629	1UYQ:A	35.64	149	447	351	441
A0A0T8QCZ5	5FOO:A	45.61	260	480	1	110
A0A0T7Z081	4B3K:A	47.95	1149	479	1	459
A0A0T8PUS0	1QOX:A	41.24	856	449	5	443
A0A0T8ALD0	1UYQ:A	39.13	165	447	356	441
A0A0Y3JN85	4B3K:A	48.38	1164	479	1	459
A0A111FXJ5	1UYQ:A	33.05	593	447	5	441
A0A0T9AI77	3GNO:A	47.73	237	488	64	150
A0A0T8TLC8	1UYQ:A	33.05	592	447	5	441
G4HKQ4	1QOX:A	89.51	2190	449	1	448
A0A0T8SYL5	4B3K:A	47.95	1154	479	1	459
A0A0T8EII7	5FOO:A	48.49	1179	480	1	460
A0A0T8U444	5FOO:A	47.19	1153	480	1	460
A0A0T9AER4	4B3K:A	50.00	737	479	184	459
A0A0U0KIB7	4B3K:A	65.58	1692	479	3	461
A0A0U0HLV8	4B3K:A	51.82	694	479	213	459
A0A0P6YDC3	2O9P:A	46.53	1098	454	18	449
A0A0P6Z665	2O9P:A	46.06	1088	454	18	449
A0A0U0KVE8	1UYQ:A	33.05	591	447	5	441
A0A0T8KCR7	1UYQ:A	32.48	586	447	5	441
A0A0T7K5J3	5FOO:A	48.28	1166	480	1	460
A0A0U0IRR0	4B3K:A	59.79	318	479	363	459
A0A0T9AH70	5FOO:A	44.76	484	480	1	206

E7RC93	1QOX:A	62.14	1578	449	1	449
A0A0L1LVD4	3W53:A	46.47	961	506	46	504
A0A0X9UAT9	5FOO:A	48.06	1163	480	1	460
A0A0T8THB7	5FOO:A	48.48	1182	480	1	460
A0A0L0H003	3TA9:A	45.00	1008	458	12	450
J4XRR1	1UYQ:A	32.47	555	447	5	441
A1JNB7	1UYQ:A	29.66	502	447	54	441
D3P5V9	1OD0:A	43.14	958	468	21	463
A0A0W1BNW5	2O9P:A	43.32	994	454	18	451
A0A0W1BP02	2O9P:A	43.02	971	454	18	449
A0A0N6ZV69	1GNX:A	57.77	1173	479	18	470
A0A150MKW7	1UYQ:A	34.98	706	447	6	441
A0A0N6ZTF1	1GNX:A	90.61	2234	479	1	479
A0A0B2BNX5	1NP2:A	52.06	1023	436	6	429
A0A126Y788	1GNX:A	68.97	1636	479	18	479
A0A126Y5X3	1GNX:A	76.41	1894	479	1	477
A0A085G494	4B3K:A	39.37	927	479	1	465
A0A0N6ZUJ3	3WH5:A	55.88	1225	457	6	446
A0A085GLV6	4B3K:A	45.32	1074	479	3	459
D3P3S6	3TA9:A	42.32	928	458	10	451
A0A126YDG3	3WH5:A	55.58	1208	457	6	434
A0A0Q9SHL8	1GNX:A	58.76	1276	479	18	468
A0A0E2PCP3	1OD0:A	45.48	1003	468	26	453
E2NPA7	1UYQ:A	32.41	621	447	5	441
D3P757	3TA9:A	44.39	967	458	11	455
A0A0Q9S1Z4	1GNX:A	51.38	1125	479	16	477
D3P779	1OD0:A	45.82	1030	468	28	466
A0A0Q9SCY2	3W53:A	48.93	1032	506	46	503
A0A085GCD2	4B3K:A	48.16	1176	479	2	459
A0A0N6ZNZ5	1NP2:A	51.00	923	436	6	430
T1ZGJ3	4B3K:A	48.07	1189	479	1	459
U1JXP4	2O9P:A	42.79	971	454	18	449
A0A0U3LW38	1GNX:A	56.14	1172	479	18	472
A4X939	3CMJ:A	50.79	1148	465	25	463
A6DUB8	1OD0:A	45.29	1011	468	27	465
K8BAR2	4B3K:A	40.51	962	479	1	470
K8C0I1	1UYQ:A	33.20	623	447	5	441
A0A0A1RS35	4B3K:A	47.59	1107	479	3	456
A0A0M6YJY7	1OD0:A	42.46	929	468	26	453
A0A087K4Q9	3WH5:A	45.39	905	457	6	446
A0A0R2SVI2	3TA9:A	45.83	1047	458	13	456
A0A098M8A7	1QOX:A	59.60	1495	449	1	448
L0IIV7	5DT5:A	53.86	1317	471	26	463
A4X576	1GNX:A	59.74	1340	479	16	472
A0A0P0MBL4	3AHX:A	46.82	1089	453	2	441
A0A0U3LL56	3WH5:A	45.18	897	457	6	446
A0A0U3LCB8	1GNX:A	68.72	1663	479	11	477
A4X1R1	1GNX:A	55.84	1267	479	14	470
K8BQP1	4B3K:A	47.07	1122	479	2	459
I3YH25	3TA9:A	49.24	1173	458	9	455
I0R7E1	3TA9:A	29.26	428	458	18	452
A0A087K759	3WH5:A	57.46	1279	457	6	449
I3Y5S0	3CMJ:A	46.21	1022	465	21	464
A0A0V8GHV7	3AHX:A	40.69	958	453	4	440
A0A087KGB8	3W53:A	51.73	1174	506	46	503
C2FAB9	1UYQ:A	33.76	657	447	4	441
C2F9E3	4B3K:A	57.02	1440	479	1	456

A6DGU2	4GXP:A	50.21	1159	467	11	464
F6IDL9	1VFF:A	30.02	513	423	5	408
A0A0A0J974	1GNX:A	52.83	1141	479	18	473
A6DLV2	4GXP:A	50.54	1181	467	13	465
A0A0F5Q355	3TA9:A	48.36	1031	458	13	436
A0A087K8X1	1GNX:A	68.72	1658	479	11	477
A0A0V8GEY5	5DT5:A	89.96	2236	471	24	471
A0A0U3KF18	3WH5:A	56.57	1265	457	6	449
R7RUN9	3AHX:A	62.28	1536	453	1	447
A0A087KCY5	1GNX:A	66.74	1604	479	9	479
U1JYQ2	2O9P:A	43.45	993	454	18	451
K2Q1B7	1OD0:A	43.79	1016	468	25	463
A0A0U3LHT9	1GNX:A	66.53	1602	479	9	479
A0A0D7N7J6	3WH5:A	43.85	913	457	6	441
G5IC88	1OD0:A	46.98	1047	468	28	465
A0A0N0AST4	1GNX:A	75.70	1778	479	14	476
A0A117L2T0	1OD0:A	90.32	2191	468	25	468
S2YRW1	1GNX:A	68.79	1653	479	10	477
A0A074LMB2	2O9R:A	55.20	1361	452	12	452
A0A0U2XFH3	1QOX:A	44.32	960	449	4	441
S2YRJ9	1GNX:A	87.47	2177	479	1	479
A0A0T1SL12	3WH5:A	54.09	1188	457	6	444
W0V5Z2	3TA9:A	47.36	1053	458	10	443
A0A098UBW7	3TA9:A	48.90	1103	458	10	457
H6NMQ2	1BGG:A	59.96	1496	448	1	446
A0A0T7D0Q1	2O9P:A	45.41	1088	454	10	449
A0A0L8PRD7	1GNX:A	68.18	1614	479	16	476
J0N0T0	1OD0:A	39.96	870	468	27	465
A0A0B3Y5K1	2JIE:A	43.08	1004	454	9	454
G5I9K8	1OD0:A	43.47	1020	468	32	464
A0A0B3XR14	3CMJ:A	43.21	976	465	21	460
A0A0T1SCL4	1GNX:A	73.28	1749	479	14	476
A0A0L8PTA5	1GNX:A	69.49	1663	479	11	479
A0A0T1S683	1GNX:A	53.55	1132	479	17	467
A0A0D7PHQ3	3WH5:A	46.17	920	457	6	441
S2YBY3	1NP2:A	50.00	918	436	8	430
A0A0N0AQ64	3WH5:A	54.48	1219	457	4	448
A0A0M8Y623	1GNX:A	69.26	1611	479	16	476
G5IMS5	3AHX:A	47.89	1146	453	1	447
A0A101EQH9	1OD0:A	98.88	2345	468	24	468
A0A0L8Q9D9	3ZJK:A	47.53	901	431	8	430
G5IHC4	1QOX:A	45.93	978	449	5	442
A0A098UII0	3CMJ:A	50.82	1115	465	31	455
E6S7C2	3W53:A	55.80	1287	506	46	499
H6N9L4	2O9P:A	53.71	1341	454	10	454
S2YZB7	3WH5:A	55.66	1240	457	6	446
A0A0T1SJX2	1GNX:A	68.25	1578	479	15	476
A0A154VLE0	1OD0:A	46.41	1072	468	25	466
A0A0A2UWI2	5DT5:A	64.09	1570	471	25	463
B0T4Z7	1GNX:A	45.18	908	479	18	479
A0A0F2IVI1	2O9P:A	45.85	1067	454	16	449
R5ZQZ5	1OD0:A	43.66	974	468	28	464
A0A0B4DM32	3W53:A	47.21	944	506	46	503
A0A136I0T0	2JIE:A	43.97	994	454	9	454
G7ZEM2	4PTV:A	43.05	947	452	4	447
E9UUK0	1QOX:A	47.39	1014	449	5	443
L8J8Z6	4PTV:A	43.66	1015	452	7	444

G7ZID5	3TA9:A	45.29	995	458	11	455
E9UXK4	3W53:A	50.72	1215	506	35	506
A0A0K2RIW5	3CMJ:A	47.22	1111	465	23	459
G7ZIC3	1OD0:A	46.10	1071	468	22	466
A0A0F4J890	1GNX:A	72.39	1718	479	18	476
G7ZH49	4PTV:A	43.34	983	452	7	449
A8L4K2	3W53:A	52.36	1175	506	46	506
A0A136I1I5	3TA9:A	44.47	990	458	12	454
A0A0F4JPP7	4HZ6:A	48.07	1032	444	5	438
A0A0F4IV83	3WH5:A	55.88	1214	457	6	446
H8GDM6	3W53:A	53.81	1236	506	45	502
A0A0S9QPS4	3W53:A	46.72	976	506	35	503
A0A127CMC6	1QOX:A	46.83	1001	449	5	441
A0A117RXI0	1NP2:A	50.67	925	436	7	430
I2X5G8	1QOX:A	34.68	607	449	5	449
A0A0F0HCP0	3WH5:A	53.86	1151	457	6	444
D9VHM8	1NP2:A	51.74	997	436	8	426
K0A8J9	5DT5:A	100.00	2429	471	24	471
D9UYK1	1GNX:A	56.56	1199	479	16	477
A0A0Q6PPJ2	1GNX:A	62.66	1486	479	13	477
H0S8U3	3TA9:A	43.27	971	458	14	458
A0A0Q6PNC8	3W53:A	71.76	1806	506	41	506
A0A0Q6PS41	3TA9:A	44.54	966	458	13	452
A0A0F0HB62	1GNX:A	65.15	1497	479	16	476
H0SMM9	1OD0:A	42.19	927	468	26	467
A0A101L4W0	1QOX:A	43.61	935	449	8	441
A0A124ID14	1GNX:A	68.72	1674	479	10	478
A0A0Q7QBW8	1QOX:A	47.05	1013	449	5	441
A8GA02	4B3K:A	48.15	1193	479	3	459
G7EF52	3AHX:A	42.06	957	453	1	440
A0A0L8LPE2	3W53:A	49.44	987	506	45	491
N6W1U8	3TA9:A	44.22	1016	458	12	452
I2X071	1QOX:A	34.47	604	449	5	449
A0A085FII2	1OD0:A	45.12	1038	468	28	465
S3B776	3W53:A	55.74	1304	506	36	501
A0A0W1KW42	3TA9:A	45.68	989	458	17	452
A0A0N1AXA3	1QOX:A	46.94	1032	449	5	443
W1RQB9	1OD0:A	44.30	983	468	16	466
D3PLV5	3WH5:A	60.68	1357	457	1	440
A8GJM5	3TA9:A	45.10	1043	458	12	450
A0A095XJG5	1OD0:A	44.25	1042	468	16	463
S2LVQ2	4B3K:A	56.58	1426	479	1	456
G7EHU0	4PTV:A	43.36	1013	452	2	446
A0A0G3X6P8	1OD0:A	48.87	1065	468	28	465
A0A0P0NMM1	1BGA:A	40.95	874	447	5	439
D3PQW3	1OD0:A	48.30	1042	468	28	463
C6PVA7	4B3K:A	46.41	1144	479	3	459
A3JMJ0	1OD0:A	42.86	937	468	27	463
A9AYT4	3TA9:A	53.51	1271	458	13	450
A9AYR3	3TA9:A	49.56	1189	458	8	456
A0A0B5L2L8	1OD0:A	90.54	2195	468	25	468
J8RCF0	3TA9:A	40.69	930	458	13	451
D7UV53	1QOX:A	41.01	883	449	5	449
A0A072CG79	1QOX:A	48.19	1071	449	5	444
D7UXB4	4B3K:A	49.35	1220	479	2	459
F5YNT1	3TA9:A	51.84	1241	458	8	452
A0A0V8SNK2	1GNX:A	61.60	1480	479	7	477

F5YNT7	1OD0:A	40.90	952	468	28	465
D7UV05	1UYQ:A	31.36	602	447	4	441
A0A0A0K5H9	1GNX:A	54.07	1174	479	17	470
A4AF25	3W53:A	59.53	1408	506	39	499
A0A085HGX9	4B3K:A	39.75	943	479	1	470
G0GFK4	3TA9:A	47.88	1109	458	8	455
A0A0R2QZI4	3TA9:A	49.44	1193	458	15	453
A4AFR4	1GNX:A	51.61	1117	479	3	467
D7UW26	1BGA:A	32.29	613	447	1	441
F5YNN0	1UYQ:A	32.98	611	447	5	441
A0A0R2QM17	3TA9:A	48.85	1149	458	17	452
D7UWY0	4B3K:A	44.25	1084	479	1	459
A0A0V8TAB5	5AYI:A	44.73	888	457	5	443
A0A0D7QRU5	1GNX:A	60.59	1406	479	13	477
B9L147	3TA9:A	50.55	1234	458	8	452
A0A085H8M1	4B3K:A	47.84	1160	479	2	461
A0A0Q0XT79	1OD0:A	42.76	997	468	24	461
K0EER5	2JIE:A	43.08	997	454	9	454
A4AK24	1GNX:A	60.30	1383	479	13	477
F5YLG7	3AHX:A	33.47	619	453	3	445
D7UWE1	1UYQ:A	30.42	573	447	3	441
D7UW38	1QOX:A	38.44	754	449	5	449
A0A0B8XPV5	1OD0:A	41.85	959	468	21	461
T0TSU1	4B3K:A	46.41	1099	479	3	459
K2IR40	1OD0:A	42.89	964	468	26	464
X2H6V1	1QOX:A	34.40	704	449	5	443
A3V9E7	3W53:A	44.13	990	506	45	500
A6UD86	3AHX:A	45.93	1019	453	5	442
Z5XXT5	3TA9:A	43.47	980	458	12	452
A6T9X1	3TA9:A	47.32	1070	458	13	450
Z5XSU4	3TA9:A	42.73	977	458	12	451
E8KV08	2RGL:A	45.35	220	481	70	154
A0A072CAW3	1QOX:A	46.15	1025	449	5	444
B8EBW4	3CMJ:A	43.05	973	465	28	458
A0A0Q8A5B5	4PTV:A	44.34	995	452	4	444
A0A0T7DJF3	2O9R:A	44.74	1071	452	8	447
M3D2W6	1GNX:A	66.81	1644	479	12	477
A0A0P0P0I4	1OD0:A	38.80	875	468	26	466
U4R6Q3	1OD0:A	42.54	953	468	28	465
A0A0R3L252	3TA9:A	43.78	921	458	13	455
A0A0M8V6K2	1GNX:A	68.31	1669	479	14	479
A0A0W1F394	1OD0:A	49.77	1094	468	28	465
D2Q1I7	1NP2:A	50.11	998	436	8	430
I2F6Y5	3TA9:A	53.38	1223	458	10	452
A0A0E4FYJ7	3TA9:A	42.47	914	458	17	454
D2PL17	1GNX:A	58.55	1366	479	8	472
A0A0S9Q9E5	1NP2:A	51.82	992	436	11	431
M3FUY6	1NP2:A	50.22	957	436	7	432
A0A0L8F3N5	3AHX:A	44.28	998	453	4	441
A0A0M8VAL7	3WH5:A	55.61	1208	457	11	446
M3EK39	1GNX:A	89.14	2192	479	1	479
D2PL35	1GNX:A	51.28	1148	479	4	472
D2PR30	4HZ6:A	46.31	1005	444	4	443
A0A0M8VHV9	1NP2:A	49.22	903	436	6	430
A0A0E3VUW2	3WH5:A	46.40	934	457	3	441
A0A0Q5NHD7	3TA9:A	48.06	1074	458	12	444
A0A0R3LCR8	3TA9:A	43.27	917	458	13	455

F3NAM3	1NP2:A	50.90	940	436	12	432
F3NIG0	1GNX:A	67.03	1639	479	15	477
A0A0M2HKR7	1VFF:A	32.80	565	423	1	405
A0A0Q5I3J8	1GNX:A	59.28	1404	479	13	477
A0A0X3XP64	1GNX:A	68.38	1643	479	12	477
E4MZV6	3WH5:A	55.66	1228	457	6	446
A0A0C1R2B8	3TA9:A	41.23	932	458	13	455
F1ZUP6	5DT5:A	55.20	1392	471	24	463
A0A0X3XPM3	1GNX:A	87.89	2189	479	1	479
A0A0D0G9C2	4PTV:A	53.71	1230	452	7	444
Q1MBM6	1QOX:A	47.29	1047	449	5	444
A0A0Q5DY39	3TA9:A	47.85	1080	458	13	450
A0A0Q5HGK7	1GNX:A	59.05	1339	479	7	470
F3NEX3	3WH5:A	56.36	1231	457	6	444
A0A0D0P4K6	4HZ6:A	47.97	1028	444	4	438
A0A0M2HVL8	3W53:A	54.29	1270	506	45	501
A0A0E3V7Q7	1OD0:A	43.53	943	468	28	464
A0A034T2M4	3TA9:A	43.26	1027	458	13	452
D8UQ06	1UYQ:A	33.55	607	447	5	444
A0A0X7A059	1OD0:A	44.12	1032	468	25	466
Q5KXG4	1UYQ:A	34.75	712	447	6	441
E4N3R8	1GNX:A	66.59	1562	479	18	477
E6VGW0	3TA9:A	42.40	931	458	15	454
F3NIF5	1GNX:A	70.45	1723	479	13	477
A0A0X3XQQ6	3WH5:A	57.01	1266	457	6	446
Q5KUY7	1QOX:A	41.08	881	449	5	443
D2QBR1	1OD0:A	44.35	961	468	28	467
A0A0S6UWR9	1OD0:A	42.12	909	468	28	465
A0A0M2HNU0	3CMJ:A	42.60	881	465	26	465
E4N5Z2	3CMJ:A	47.18	1018	465	26	459
F4LRK3	3TA9:A	41.30	904	458	15	452
A0A0X3XGT4	1NP2:A	50.78	937	436	6	430
E4NBD1	1GNX:A	66.18	1560	479	4	476
A0A0M3DAJ0	3W53:A	54.49	1261	506	36	501
M5B8Z3	1GNX:A	59.26	1393	479	3	479
A0A0L0KIU7	3ZJK:A	48.43	907	431	8	430
A0A0L0K065	1GNX:A	69.57	1668	479	11	477
A0A0L0JIW0	3WH5:A	56.25	1255	457	6	449
E0NZZ0	3TA9:A	40.95	179	458	355	450
A0A0L0JGV9	1GNX:A	68.10	1607	479	14	476
A1SNN0	1NP2:A	52.90	1055	436	6	430
A0A0L0JHM1	1GNX:A	56.77	1174	479	18	472
A0A0H3C0Y6	4B3K:A	44.88	1056	479	3	459
A0A0T6ZA30	1QOX:A	47.85	1046	449	5	441
A0A0L0JEL4	3CMJ:A	48.43	1077	465	21	458
A1SQJ7	3W53:A	48.14	1010	506	46	500
A0A0H3BZ57	4B3K:A	98.33	2538	479	1	479
A0A0N0A4A4	3TA9:A	42.24	906	458	15	451
A0A0M8W781	1GNX:A	54.74	1223	479	15	477
W7NGV8	4B3K:A	49.35	1179	479	2	461
A0A150JWH8	4B3K:A	50.00	1248	479	3	459
A0A0N0A0C0	3W53:A	52.46	1286	506	41	506
A0A0M8WJJ4	1GNX:A	52.76	1104	479	16	467
A0A0L0AP41	3TA9:A	45.51	1052	458	13	450
A0A089XBM8	1GNX:A	65.83	1634	479	1	479
A0A0F7NE55	3WH5:A	58.37	1274	457	4	444
A0A0N8TWM4	3TA9:A	45.43	1030	458	17	454

A0A0F7N5B2	1GNX:A	71.24	1724	479	13	477
A0A0P4R6P8	3WH5:A	48.46	975	457	5	443
A0A089X8V3	1NP2:A	49.33	904	436	8	430
A0A0Q9N6R4	1QOX:A	44.42	955	449	5	441
A0A0Q7GH87	1GNX:A	59.49	1407	479	13	477
E3IUS0	1GNX:A	51.53	1128	479	15	472
A0A101JF42	1GNX:A	52.72	1089	479	10	466
A0A101JFL3	1GNX:A	58.53	1367	479	1	472
D4K1K7	4GXP:A	33.60	562	467	13	464
A0A0Q9S832	1NP2:A	49.78	988	436	7	435
A0A101JT74	1NP2:A	53.20	1057	436	7	426
A0A0P4RAL1	3WH5:A	54.42	1159	457	6	443
A0A089Z8E9	1GNX:A	89.79	2212	479	1	479
Q2S749	3TA9:A	50.23	1153	458	13	452
A0A089XFH1	3WH5:A	53.93	1211	457	6	449
J7L8F2	3TA9:A	46.46	1080	458	13	452
A0A0S2S780	1BGG:A	94.87	2325	448	1	448
A0A0Q9N857	1NP2:A	44.44	927	436	8	426
A0A089XFH5	1GNX:A	53.15	1122	479	7	472
A0A098QUS4	1QOX:A	43.24	1002	449	4	443
J2H761	1QOX:A	41.88	901	449	3	449
J7LBN1	1GNX:A	56.49	1251	479	18	477
C6W5Y0	3CMJ:A	43.84	979	465	26	458
A3TGZ0	1GNX:A	55.68	1217	479	17	470
A0AFB1	3TA9:A	38.38	810	458	16	451
A0A0P6XTN0	3TA9:A	50.33	1207	458	8	457
H5XAW9	3W53:A	55.97	1334	506	41	503
A0AF81	3AHX:A	40.25	874	453	2	445
A0A024H7A5	3W53:A	68.80	1724	506	36	503
G2PEP9	3WH5:A	56.11	1230	457	6	446
S6GNS4	3TA9:A	45.80	1114	458	12	450
H8E6T3	3W53:A	55.74	1304	506	36	501
A0A091CE87	1QOX:A	36.02	510	449	103	449
A0A0A1DS80	3CMJ:A	47.40	1028	465	25	462
A0A024GZT8	3W53:A	46.37	957	506	46	505
A0AF66	1OD0:A	31.89	624	468	21	465
A0AG26	4B3K:A	50.22	1228	479	3	459
W7IPT8	1QOX:A	47.02	1013	449	5	445
X5NUA2	4B3K:A	49.57	1199	479	3	459
W7IVY8	3W53:A	53.78	1214	506	45	504
A0A024GYL3	3W53:A	44.47	908	506	46	499
W8YRW3	1QOX:A	60.00	1495	449	5	449
A0A0D0G0X7	1UYQ:A	41.23	872	447	5	438
A0A144MT99	3W53:A	44.16	907	506	51	504
A0A0M2T717	3TA9:A	44.77	1044	458	12	451
A0A0Q6WIJ1	3WH5:A	50.68	1145	457	4	440
A0A0U5H4F0	1NP2:A	50.79	918	436	8	426
B5XXA4	3TA9:A	33.06	591	458	8	450
A0A0Q6WUH5	1QOX:A	43.89	941	449	5	442
Q7MG41	4PTV:A	46.79	1046	452	10	444
A0A099D543	3W53:A	57.52	1322	506	45	502
A0A098TDE4	1OD0:A	48.74	1094	468	27	461
A0A0U5HFY8	3WH5:A	56.79	1251	457	6	446
A0A0U5HHD9	1GNX:A	56.39	1133	479	18	467
A0A0M5LL83	3TA9:A	46.21	1061	458	10	452
A0A0Q6WU06	3WH5:A	45.45	1007	457	4	441
A0A0Q6X5W0	1E4I:A	42.31	947	447	5	440

M9RMG1	1OD0:A	42.19	950	468	26	453
A0A058ZJC5	1OD0:A	44.24	984	468	21	453
A0A0Q6W5S0	4PTV:A	49.54	1130	452	11	446
A0A0U5M6X4	1GNX:A	68.31	1660	479	14	479
S4YMW4	4PTV:A	44.44	1030	452	6	444
A0A098RPC9	1OD0:A	44.80	1038	468	25	462
A0A0Q6VNH8	1GNX:A	58.11	1306	479	18	472
A0A0Q6VEF7	1GNX:A	56.83	1242	479	18	470
W0A629	4B3K:A	47.83	1181	479	3	459
A0A0Q6X8T7	1QOX:A	44.80	958	449	5	442
A0A0Q6WXJ2	1OD0:A	46.74	1043	468	26	465
A0A0Q6X6S9	4PTV:A	48.64	1119	452	7	445
B5XWQ4	4PTV:A	46.31	1051	452	7	444
V9ZZ66	1QOX:A	42.49	916	449	5	445
E4LYQ9	1TR1:A	34.96	706	447	4	441
A0A0Q8GK83	3WH5:A	52.05	1172	457	4	440
A0A0S9CMV1	1QOX:A	44.62	943	449	8	441
A0A071IES9	1QOX:A	49.10	1091	449	5	444
A0A060DVX9	3TA9:A	44.44	997	458	8	456
A0A060DMA5	3TA9:A	43.89	999	458	13	450
E4LT50	1OD0:A	29.36	494	468	28	465
E4LZ66	1OD0:A	32.16	598	468	21	467
E4LYB4	1UYQ:A	29.87	536	447	5	441
A0A0Q8GG41	3TA9:A	48.62	1093	458	13	447
A0A0L0LVF3	1GNX:A	48.72	1111	479	16	475
A0A0N7I902	4PTV:A	43.89	996	452	7	444
A0A0Q8GMG1	3WH5:A	46.76	1022	457	4	446
A0A0Q8H6P4	1E4I:A	42.70	936	447	5	440
K4R9P9	1GNX:A	66.60	1630	479	12	477
C4Z6T5	1OD0:A	43.68	979	468	28	464
E4LW77	3TA9:A	31.96	546	458	13	450
A0A0Q8GD26	1OD0:A	45.72	1021	468	26	465
A0A0Q7K4S6	1GNX:A	62.26	1445	479	18	479
A0A0Q8GHX1	4PTV:A	47.72	1097	452	1	446
E4LNR8	3TA9:A	34.47	620	458	15	456
E4LW40	1TR1:A	30.90	535	447	5	441
E4LNK4	1E4I:A	57.14	282	447	6	96
R6ZYM6	1OD0:A	43.02	965	468	27	465
K4QVT5	3WH5:A	54.05	1211	457	6	448
A0A060DQK7	3TA9:A	42.92	989	458	13	457
A0A0P0F0Y5	3TA9:A	45.76	1045	458	13	458
E4LRI3	4GXP:A	34.61	570	467	17	464
E4LXN4	1OD0:A	32.29	588	468	30	467
K4R1I7	1NP2:A	48.88	925	436	8	430
A5IL97	1OD0:A	98.88	2345	468	24	468
A0A0B0HK50	2JIE:A	43.33	262	454	233	350
A0A060DJG7	3TA9:A	45.54	1051	458	13	458
A0A0P0EHD0	3TA9:A	44.10	990	458	8	455
A0A0P0ERM0	3TA9:A	42.31	969	458	13	454
A0A0B0HH03	3TA9:A	61.44	516	458	13	165
W2VAQ7	1GNX:A	52.95	1158	479	17	469
A0YUE1	1OD0:A	45.53	977	468	27	464
M2YAZ1	1NP2:A	53.71	1085	436	8	428
A0A0B2AU34	1NP2:A	46.93	878	436	8	427
A0A147KFJ2	1GNX:A	58.17	1357	479	7	474
M2ZB83	3TA9:A	46.52	1026	458	13	456
M2ZG92	3TA9:A	43.82	991	458	8	450

M2XRQ3	3W53:A	47.84	1051	506	46	503
J0NGX7	1UYQ:A	34.26	615	447	5	441
M2X794	3W53:A	55.43	1253	506	45	502
M2YLB3	3WH5:A	52.04	1183	457	6	446
D2C6W2	1OD0:A	98.88	2345	468	24	468
U2CBP5	3CMJ:A	43.66	971	465	21	464
A0A147KMZ8	1GNX:A	54.09	1144	479	18	470
A0A0F7CHR9	1BGG:A	61.30	1569	448	1	446
M2YJT2	1GNX:A	48.60	1066	479	18	478
S2P6U4	4B3K:A	56.58	1432	479	1	456
A0A0C2TJZ0	1UYQ:A	35.65	676	447	5	441
A6X2M0	3WH5:A	49.44	1076	457	2	447
K9WQ83	3CMJ:A	43.83	951	465	21	461
H3R9U2	1UYQ:A	34.54	701	447	1	441
A0A086ZNH9	1GNX:A	55.39	1298	479	19	478
A0A0K0I289	4B3K:A	40.25	931	479	1	467
A0A0F6ALA9	1OD0:A	99.33	2354	468	24	468
A0A0F4KH84	1QOX:A	47.32	1090	449	5	443
A0A0K0I0D8	4B3K:A	47.72	1183	479	2	459
A0A0F4KFS6	3WH5:A	55.23	1192	457	6	444
A0A0D3LHF6	1OD0:A	44.22	989	468	26	461
H3RE30	1UYQ:A	32.72	591	447	5	445
H3RIM2	4B3K:A	42.89	817	479	3	380
A0A0C2PJ36	4B3K:A	48.16	1192	479	2	459
A0A0K0I4N2	1QOX:A	38.41	789	449	1	444
A0A0C2TLE6	3TA9:A	32.85	562	458	13	451
A0A0K0I0Z3	1UYQ:A	34.57	664	447	5	441
S3C2X1	1GNX:A	51.62	1013	479	17	472
K9WM42	3CMJ:A	42.14	916	465	17	461
A0A0F4KGD7	1GNX:A	66.52	1571	479	16	477
S3AU36	1GNX:A	71.34	1686	479	15	477
A0A0C2TJW8	4B3K:A	40.22	933	479	1	461
A0A0C2PIF5	1QOX:A	38.10	778	449	5	444
A0A0A2HBW8	1OD0:A	43.95	1042	468	21	462
A0A0W8EVK2	1OD0:A	44.39	1048	468	21	462
S2NFY0	1UYQ:A	33.76	663	447	4	441
F2RLS6	3ZJK:A	51.68	1024	431	7	426
E8LUA8	3TA9:A	45.18	1022	458	16	450
A0A0P9FDI8	5DT5:A	43.92	984	471	23	465
A0A0Q5EKW2	3W53:A	66.67	1670	506	36	504
A0A0M2GZY7	1GNX:A	67.88	1653	479	12	477
A0A0F4NPY6	2O9P:A	45.60	1044	454	18	449
E4Q7Z7	3AHX:A	52.52	1238	453	4	444
A0A0M2GKD2	1GNX:A	57.21	1188	479	17	467
A0A0A7KKG8	3WH5:A	55.46	1240	457	6	448
A0A0B0S9N8	3ZJK:A	81.56	1738	431	4	426
A0A0N0VN29	5DT5:A	73.29	1790	471	24	461
Q7CV27	1QOX:A	46.95	1047	449	5	444
A0A0Q0NVG9	3TA9:A	44.14	1009	458	9	452
F2R8K2	1GNX:A	54.03	1158	479	3	472
F2RKF5	1GNX:A	68.82	1597	479	16	479
S3FEZ0	3AHX:A	41.52	981	453	6	440
A0A0M2GZ61	1NP2:A	51.68	933	436	8	430
T2KKC3	3AHX:A	40.90	839	453	5	444
F2RGF6	1GNX:A	73.28	1751	479	14	476
A0A0C2LD29	3CMJ:A	43.11	932	465	25	461
F2R8J7	3WH5:A	55.45	1204	457	4	437

E5YL48	3TA9:A	43.45	995	458	13	450
D4JG79	1TR1:A	31.01	582	447	5	441
A0A0B0SBM0	1NP2:A	98.37	2196	436	1	430
M1QQC9	3TA9:A	40.26	926	458	13	451
S3FCI8	5DT5:A	83.30	2066	471	24	466
D4JEZ1	4B3K:A	37.37	923	479	1	461
U1K5V5	3TA9:A	44.25	1030	458	8	452
Q53W75	3ZJK:A	99.54	2215	431	1	431
A0A0K8JF42	3AHX:A	43.20	951	453	4	440
A0A0U3SAF1	1NP2:A	51.37	922	436	8	423
W1HHQ1	3TA9:A	47.32	1067	458	13	450
F7K4L0	4PTV:A	43.27	969	452	2	450
A0A0U3RIE4	1GNX:A	90.81	2245	479	1	479
U1J1N0	2O9P:A	41.88	967	454	13	449
U6FI09	2E9L:A	35.71	55	469	288	314
A0A0Q9N1K2	4PTV:A	40.55	918	452	7	452
G7ET08	3TA9:A	44.72	1016	458	12	454
A0A0S9DCH0	3W53:A	57.91	1347	506	40	502
A0A0N0MVS6	1GNX:A	68.60	1657	479	16	479
A0A0B8QE43	3TA9:A	46.12	1034	458	16	450
A0A0B8QLW1	4B3K:A	49.38	223	479	379	459
A0A0B8QPM7	4B3K:A	44.09	779	479	1	345
A0A0Q5AXY6	3W53:A	68.34	1664	506	49	506
A0A0Q9MDS1	1GNX:A	47.92	945	479	5	472
R0A635	4PTV:A	47.76	1130	452	7	449
A0A0N1NAQ0	1NP2:A	49.89	940	436	8	426
A0A0B8QRR9	4B3K:A	54.63	333	479	352	459
A0A0B8QNU0	3W53:A	53.45	372	506	107	221
A0A0N1NEX6	3WH5:A	55.93	1228	457	6	449
U4KIY0	3TA9:A	47.50	1079	458	12	450
A0A0B8QJZ1	5FOO:A	42.42	784	480	3	357
W1HGY6	4B3K:A	40.30	968	479	1	470
A0A0U3HG46	1GNX:A	66.24	1621	479	9	477
E1VSX4	3W53:A	93.64	2381	506	35	506
A0A0U3R8G3	3WH5:A	56.95	1226	457	6	443
A0A0B8QJC8	5DT5:A	33.33	317	471	245	470
W1HE52	5FOO:A	42.28	597	480	170	464
F7KF50	1OD0:A	44.97	1034	468	28	465
A0A0N1KDT4	1GNX:A	51.62	1016	479	17	472
A0A0U3EHY6	1OD0:A	46.98	989	468	28	453
A0A0Q6RH46	3W53:A	56.30	1343	506	44	500
A0A075UTP7	3W53:A	54.35	1242	506	45	502
A0A0F2GBE9	3TA9:A	47.27	1026	458	13	450
I4ERL5	3W53:A	52.60	1212	506	43	503
B8HM35	3WH5:A	46.04	924	457	6	444
A0A0P0U7Y3	1OD0:A	43.99	1002	468	28	463
A0A150HHU3	3W53:A	56.37	1318	506	36	501
A0A0Q6RLN9	1GNX:A	60.96	1431	479	6	477
Q21ZF1	3WH5:A	51.82	1155	457	6	443
B5YCI2	1VFF:A	44.68	917	423	4	416
A0A075UHQ3	3W53:A	48.36	1068	506	46	499
A0A0N0Z262	1GNX:A	71.27	1681	479	16	477
A0A0U3N6M7	1GNX:A	46.65	987	479	18	476
A0A0B0D6F9	1TR1:A	56.66	1423	447	3	444
A0A075UMV5	3W53:A	49.35	1047	506	43	502
B7GM33	3AHX:A	53.36	1238	453	1	440
A0A110AVA9	1GNX:A	45.30	762	479	18	421

V3K6Z5	3TA9:A	46.88	1053	458	13	450
C9Y5F8	1UYQ:A	35.27	616	447	5	446
H0S301	1OD0:A	42.86	934	468	26	467
B5YAN1	3TA9:A	58.41	1406	458	8	457
C9XUV9	4PTV:A	33.12	626	452	2	444
C9Y3J9	4B3K:A	47.51	1143	479	2	459
Q21ZG0	3TA9:A	48.75	1135	458	13	452
A0A075UKX9	3TA9:A	44.32	985	458	13	450
H0S7F0	4HZ6:A	44.47	971	444	9	440
C9XZL5	4B3K:A	40.72	964	479	1	470
K8AS35	1TR1:A	41.98	233	447	5	107
A0A0F2TGG7	3CMJ:A	47.07	1022	465	26	459
A0A0R2P8F3	3TA9:A	49.21	1185	458	15	453
K8AJR8	4B3K:A	47.29	1136	479	2	459
K8ASY6	1UYQ:A	30.41	371	447	117	441
A0A0M4U7M4	3TA9:A	46.65	1056	458	13	450
V9WHC2	1OD0:A	44.39	962	468	28	453
K6VWJ1	3W53:A	51.69	1246	506	39	502
A3VMT2	1OD0:A	45.16	1005	468	21	453
A0A0G3R1D6	3TA9:A	46.31	1060	458	13	450
K8B0Z4	4B3K:A	40.17	955	479	1	470
A0A0J2GV46	3TA9:A	47.32	1067	458	13	450
A0A0Q7AXH6	4PTV:A	47.72	1097	452	1	446
G2MUZ4	5DT5:A	55.13	1395	471	24	469
A0A0Q7BD23	3WH5:A	52.05	1172	457	4	440
S0AM59	3TA9:A	44.44	1032	458	12	450
B9JAE6	1QOX:A	49.10	1091	449	5	444
B4AJV7	1UYQ:A	35.87	679	447	5	441
A0A085WWB9	3TA9:A	47.98	1094	458	8	450
A0A0Q7BR74	1E4I:A	42.70	936	447	5	440
A0A0M2CYF8	3W53:A	67.38	1690	506	36	501
A0A0X6HAV7	1OD0:A	44.77	1043	468	21	462
A0A0N0THA8	1NP2:A	51.23	921	436	8	430
A0A085WL72	3WH5:A	53.48	1196	457	2	444
A0A0Q9UMW3	3AHX:A	43.10	968	453	4	441
A0A0M8X1X4	1GNX:A	68.95	1662	479	12	477
A0A085VZK2	3TA9:A	52.88	1265	458	11	455
C0ZYY4	3ZJK:A	49.23	1031	431	3	431
A0A0Q8E7L7	4HZ6:A	45.48	991	444	4	440
A0A0N7JUH3	4PTV:A	45.79	1044	452	1	438
B4AK23	3TA9:A	33.06	569	458	13	451
A0A0Q7AXU6	3TA9:A	48.62	1093	458	13	447
A0A0T6ALV9	1VFF:A	42.04	780	423	2	408
K2PNL2	1OD0:A	55.48	1240	468	28	456
B4AMR5	1QOX:A	38.10	780	449	5	444
A0A0Q7BD61	3WH5:A	46.76	1022	457	4	446
A0A0N0THX2	1GNX:A	57.21	1186	479	17	467
A0A0Q7ARW6	1OD0:A	45.72	1021	468	26	465
B4ALA4	4B3K:A	48.37	1195	479	2	459
G2MRY3	5DT5:A	55.20	1386	471	24	463
A0A0M2MU01	1GNX:A	69.81	1645	479	12	477
J2R701	1QOX:A	47.96	1067	449	5	444
K6ZHI5	3TA9:A	44.53	922	458	49	452
K2M7D3	1OD0:A	45.48	1019	468	15	453
A0A0A7EN85	5DT5:A	44.84	1004	471	22	465
A0A0M2MHD7	3WH5:A	55.66	1217	457	6	446
A0A0M2MU32	1NP2:A	49.77	907	436	12	430

A0A0R2RI35	3TA9:A	48.98	1184	458	15	453
A0A0M2T3Q2	1QOX:A	60.94	1489	449	2	449
A0A0H3C9V1	1GNX:A	43.32	897	479	18	479
A0A0T7DE67	2O9P:A	45.19	1095	454	10	449
A0A148KN47	3CMJ:A	46.44	1029	465	30	460
A0A0Q6QMQ8	1QOX:A	47.71	1029	449	5	438
A0A139QFH1	4B3K:A	49.30	1119	479	1	422
A0A0M3GQ75	1QOX:A	47.74	1065	449	5	444
A0A087VSR2	1GNX:A	51.26	1202	479	16	476
A0A0D6TF18	3ZJK:A	44.65	904	431	2	427
A0A139QFA8	1UYQ:A	33.55	590	447	4	441
D9TR57	5DT5:A	53.64	1313	471	26	463
A0A0Q6QR31	3WH5:A	49.77	1077	457	6	433
E4SGY9	3AHX:A	51.97	1223	453	4	444
Q47XU7	2JIE:A	42.13	989	454	18	449
B7LVM8	4B3K:A	49.24	1181	479	3	459
A0A0N8TZN0	3CMJ:A	44.19	980	465	22	458
Q1Z7Q5	3TA9:A	43.53	1027	458	13	451
A0A0D7CQQ7	3W53:A	46.95	968	506	45	506
D4MZ70	3TA9:A	38.95	859	458	8	452
A0A0S9PEN9	3ZJK:A	47.69	931	431	7	423
A0A075JVS6	1OD0:A	49.66	1104	468	28	465
A0A081CSM4	1OD0:A	43.08	986	468	22	461
D4MVD3	4B3K:A	52.37	1336	479	1	463
A0A0L8BHM2	1QOX:A	47.17	1017	449	5	441
R9CE34	3AHX:A	42.98	968	453	4	441
U5W6M1	1GNX:A	54.27	1210	479	12	467
A0A0R2PMK7	3ZJK:A	46.09	943	431	7	430
H2CFD7	3TA9:A	44.34	999	458	8	447
U5VW16	3TA9:A	48.98	1106	458	13	451
U5WB57	1GNX:A	57.05	1318	479	14	472
A0A0J8VU23	3TA9:A	44.86	1047	458	13	450
F9HIS5	3AHX:A	28.66	342	453	61	362
A0A0N0SC67	1NP2:A	51.00	1023	436	7	426
A0A0M9XZM4	3WH5:A	55.00	1192	457	6	444
U1RC45	1GNX:A	50.54	1119	479	17	476
A0A087LSW7	4PTV:A	48.47	1058	452	7	430
A0A0Q5TZT4	1OD0:A	40.72	868	468	28	462
C4LLI8	3CMJ:A	29.75	386	465	30	463
A0A0K2YKI0	3W53:A	46.90	994	506	45	503
W8UJL1	3TA9:A	47.32	1068	458	13	450
W8UVM0	4B3K:A	45.04	1067	479	3	463
W8UFH6	4B3K:A	40.30	968	479	1	470
A0A0D6IWA2	1GNX:A	56.62	1346	479	10	477
R9JK27	1OD0:A	45.88	1043	468	28	465
E6TUY6	1QOX:A	61.01	1533	449	1	449
A0A0E1XFT7	1OD0:A	39.19	819	468	21	465
A0A0X8LK10	3TA9:A	45.23	1045	458	12	450
A0A0M8SDN5	1GNX:A	66.95	1550	479	16	477
D5DZI8	1QOX:A	33.70	424	449	107	449
A0A0F4L1B4	1GNX:A	55.39	1228	479	13	470
D5E2L2	3AHX:A	43.10	968	453	4	441
F1Z0S2	3TA9:A	31.55	598	458	13	450
W4QKR4	1BGG:A	63.98	1620	448	1	446
A0A0X3S6R1	3WH5:A	46.04	909	457	5	444
F1YZM7	1UYQ:A	33.06	668	447	4	440
W8XWK7	3TA9:A	47.10	1059	458	13	450

A0A0Q9JVE0	3W53:A	56.93	1317	506	45	499
M4NMY0	3TA9:A	48.98	1096	458	13	452
B2TFW7	3TA9:A	47.83	1071	458	12	443
A0A0Q9JM05	1GNX:A	60.34	1430	479	13	477
A0A0Q9JNU3	1GNX:A	50.33	1112	479	13	467
A0A0Q7SFW2	1OD0:A	50.23	1094	468	27	465
A0A0X3SKD1	1GNX:A	70.35	1732	479	7	477
A0A0D5CLN7	1GNX:A	59.74	1366	479	16	477
A0A0X3SP08	1GNX:A	65.33	1606	479	7	477
A0A0Q9IZQ1	3WH5:A	48.32	1007	457	6	444
M4YYI1	4B3K:A	45.22	1051	479	3	459
A0A0Q7T801	1OD0:A	51.47	1135	468	27	465
F1YZM4	1BGG:A	31.20	538	448	1	442
M4YXZ5	4B3K:A	98.05	2447	479	1	461
A0A0X3SEI7	3WH5:A	55.20	1223	457	6	446
F1YXP4	1UYQ:A	31.67	561	447	4	441
I2W777	1OD0:A	32.54	604	468	25	466
U6EMH2	3AHX:A	34.10	598	453	4	440
A0A069CLU2	3TA9:A	44.88	1041	458	12	450
A0A0W8IIB6	3W53:A	54.19	1249	506	40	500
F6DJP6	1NP2:A	97.47	2209	436	1	435
F9ULH8	4B3K:A	58.61	1442	479	1	459
U6EQP7	5DT5:A	30.47	299	471	237	463
A0A0Q7A3M4	1OD0:A	51.25	1153	468	28	465
M8CQP7	4B3K:A	48.92	1193	479	3	462
A0A0C2KLS1	1OD0:A	44.16	959	468	28	453
A0A0M9YWR2	1GNX:A	65.95	1620	479	12	477
M8CYT6	5DT5:A	55.43	1402	471	24	463
C4L9V0	4B3K:A	48.91	1174	479	3	459
C4LD42	3TA9:A	44.25	1042	458	12	452
M8CQU4	1E4I:A	34.33	726	447	4	441
A0A077MDS7	1QOX:A	45.07	992	449	5	443
H6PBK3	1QOX:A	33.68	557	449	5	443
F1YKW9	1GNX:A	46.15	895	479	10	470
A0A0S3UBN2	1OD0:A	42.89	919	468	27	464
A0A126Q4N7	3TA9:A	44.22	982	458	12	452
U6EME9	4B3K:A	54.76	1416	479	1	461
A0A0M8TIP7	1NP2:A	49.67	930	436	6	430
A0A0M8TXZ8	3WH5:A	55.20	1201	457	11	449
J0BEL1	1QOX:A	46.83	1042	449	5	444
U6ET99	3AHX:A	56.76	582	453	6	189
A0A0H1AV25	1GNX:A	69.40	1652	479	18	479
A0A0T7FU31	1OD0:A	45.79	1056	468	28	462
K8QBV8	4B3K:A	56.50	1426	479	1	466
G2LE13	1OD0:A	45.78	1088	468	16	465
A0A0Q9SPK9	1GNX:A	58.76	1273	479	18	468
A0A081XU26	1GNX:A	67.30	1654	479	9	477
A0A0G3AH06	1NP2:A	50.00	917	436	7	430
A0A0W7X383	1GNX:A	69.49	1660	479	14	476
A0A0T7FNB3	1OD0:A	45.33	1055	468	28	462
A0A081XXW6	1GNX:A	91.58	2239	479	5	479
A0A0Q9SPL9	3W53:A	49.14	1031	506	46	503
A0A150UJJ5	3WH5:A	45.45	920	457	6	441
A0A081XPY9	1NP2:A	51.03	914	436	8	424
A0A087C7J4	1GNX:A	50.32	1153	479	17	476
F9XYD9	1VFF:A	34.61	578	423	5	408
A0A0G3AB23	3WH5:A	57.24	1242	457	6	446

A0A0H1AY18	3WH5:A	55.81	1217	457	6	434
A0A0G3AGN5	1GNX:A	68.09	1661	479	12	477
A0A0W0YT16	1VFF:A	31.58	499	423	10	406
B6G7Y7	3CMJ:A	35.04	691	465	23	463
A0A0A0DJG4	1UYQ:A	33.76	609	447	4	441
A0A0T7GMG4	1OD0:A	45.25	1060	468	25	462
A0A0Q8RPE3	1OD0:A	49.77	1096	468	28	465
A0A0W7XBG1	3ZJK:A	51.35	983	431	6	426
A0A0H1AXC9	1GNX:A	76.20	1906	479	1	477
K8QQB4	4HZ6:A	34.45	669	444	5	443
A0A022LMS0	5AYI:A	46.64	952	457	6	447
A0A081XV05	1GNX:A	69.25	1649	479	5	475
A0A0E0UG31	1QOX:A	47.51	1041	449	5	444
A0A0T9QJD0	1UYQ:A	30.00	560	447	5	441
U3AQY2	1OD0:A	45.05	1015	468	21	463
F5Y9D3	1OD0:A	44.49	1023	468	28	465
B3Q0Q7	1QOX:A	47.74	1062	449	5	444
A0A126YS63	5DT5:A	46.80	1059	471	29	465
A0A0N8GTC3	3TA9:A	49.34	1180	458	8	456
G9RQQ0	1QOX:A	46.89	1015	449	5	443
B0N8V4	1QOX:A	34.19	564	449	5	442
K9DZC8	3WH5:A	50.56	1125	457	6	440
A0A147EWX1	3W53:A	52.74	1249	506	39	500
K9E351	3TA9:A	48.75	1074	458	13	450
A0A0P6YCA1	3TA9:A	53.03	1274	458	13	450
A0A0V8HU92	3W53:A	84.11	2153	506	35	506
B0N3K6	3AHX:A	32.90	576	453	4	440
R5V1N6	1OD0:A	44.05	1001	468	27	465
S0FYL9	3AHX:A	52.00	1285	453	1	447
A0A0N0GQQ4	3TA9:A	47.76	1110	458	8	452
D0D2N8	1OD0:A	45.98	1034	468	21	464
F9U737	3CMJ:A	46.40	1036	465	25	464
A0A0X3R7W8	1GNX:A	49.79	971	479	11	472
A0A0N0S6N1	1GNX:A	69.43	1673	479	16	477
A0A017HBK6	3W53:A	46.85	1026	506	45	500
J2KWY4	1QOX:A	49.10	1091	449	5	444
A0A0M4H5T3	1GNX:A	60.30	1397	479	14	479
A0A017H8P6	3W53:A	46.52	1007	506	33	491
F9U6X5	3TA9:A	49.66	1176	458	14	452
Q1R4L5	1QOX:A	34.47	610	449	5	449
A0A0T7ETW5	2O9P:A	44.97	1080	454	10	449
W4Q343	1BGG:A	63.53	1606	448	1	446
E3F031	2O9P:A	45.31	1027	454	14	450
Q1R7Y4	1UYQ:A	33.05	623	447	5	441
A0A0X3RDA4	1GNX:A	69.43	1669	479	16	477
I2IRY4	5DT5:A	45.21	1035	471	29	465
B6QX09	3AHX:A	46.91	1080	453	3	436
A0A0Q7US81	1GNX:A	48.82	998	479	12	467
A0A0Q7UHV2	1GNX:A	61.83	1404	479	13	477
A0A0M8QWT2	1GNX:A	49.37	979	479	2	472
S0FIW2	4PTV:A	54.92	1332	452	11	445
M3ED66	1NP2:A	49.78	911	436	8	432
Q1AUD7	1GNX:A	58.26	1317	479	16	474
A0A099DE23	5DT5:A	84.16	2074	471	24	465
A0A099DEC7	3AHX:A	40.91	977	453	4	440
A0A109WPC7	4PTV:A	47.61	1059	452	6	438
A0A0N0TLW9	1GNX:A	64.87	1511	479	17	479

A0A0B8QBC2	3AHX:A	53.33	542	453	4	183
A0A0N0ALA8	3CMJ:A	49.66	1159	465	25	463
M3DHJ5	1GNX:A	91.23	2257	479	1	479
A0A0N0AJ32	1GNX:A	60.26	1343	479	16	472
A0A0N0TL46	1NP2:A	54.48	1095	436	1	434
A0A0J1DC04	3WH5:A	52.52	1161	457	6	440
A0A0Q6G3J3	1OD0:A	46.95	1058	468	25	463
M3D3N7	1GNX:A	58.64	1204	479	18	467
A0A0B8PXL0	5DT5:A	42.96	267	471	337	470
B7IEC2	1OD0:A	55.71	1244	468	28	456
M3BYH3	1GNX:A	65.82	1618	479	9	477
A0A151FUL3	1OD0:A	46.79	1039	468	30	463
W6M8D9	3TA9:A	43.13	1006	458	13	452
M3D101	3WH5:A	54.44	1188	457	6	443
A0A151FDK9	1NP2:A	48.20	911	436	8	432
A0A0M8XHD0	3WH5:A	53.95	1194	457	4	444
A0A0B8PJ41	3TA9:A	46.12	1030	458	16	450
W6M302	4PTV:A	51.35	1174	452	3	446
R6VC17	1OD0:A	45.75	1053	468	21	467
A0A0B8PN97	4B3K:A	46.85	1132	479	1	459
A0A0A1WBR5	1OD0:A	45.05	1012	468	17	466
S3H9L5	1QOX:A	47.29	1051	449	5	444
A0A150KLU3	1BGG:A	32.70	636	448	1	442
A0A077M7L6	1GNX:A	56.10	1239	479	17	476
H8H0B5	3TA9:A	49.21	1148	458	13	452
A0A0M8WZR1	1GNX:A	55.60	1138	479	18	467
F4FFK1	1GNX:A	56.84	1289	479	14	475
I7J5U3	3AHX:A	60.94	1520	453	1	447
A0A0J5QET7	1OD0:A	42.89	978	468	21	462
A0A0F5AH02	1GNX:A	70.68	1727	479	16	479
A0A0F5ABA1	3WH5:A	54.95	1216	457	6	446
A0A0N0TDW5	1NP2:A	50.34	922	436	8	430
G7F3X3	2O9P:A	41.88	965	454	13	449
A0A0Q8RI14	3TA9:A	46.56	1063	458	8	451
M3BZA6	1GNX:A	67.25	1560	479	17	474
A0A0F4IEP9	1GNX:A	65.67	1531	479	9	476
B7VTD4	2O9P:A	46.06	1090	454	18	449
A0A0J0UXC6	1OD0:A	40.77	903	468	15	462
F4FEA7	1GNX:A	66.31	1560	479	16	477
F4FD86	4PTV:A	50.78	1195	452	1	449
A0A0N0A859	1GNX:A	68.17	1674	479	14	477
A0A0G0JIG9	1VFF:A	37.68	728	423	3	411
G7F6V7	3TA9:A	44.25	1030	458	8	452
A0A0Q8RPN9	3WH5:A	52.05	1127	457	6	440
A0A0M8WSX9	3WH5:A	56.92	1249	457	6	445
A0A0A1CVU2	3TA9:A	46.37	1032	458	13	454
F4FEZ0	1GNX:A	62.09	1370	479	14	472
G0V3V5	5DT5:A	57.53	1437	471	24	461
A0A0J0UV64	3CMJ:A	39.61	811	465	23	461
A0A0F4I4Z2	3WH5:A	54.55	1169	457	6	444
D4LKX5	1UYQ:A	33.69	625	447	4	438
X7EHA7	1OD0:A	45.67	1021	468	28	453
F4F3X7	1NP2:A	56.32	1084	436	8	430
C1ATZ8	4PTV:A	28.89	405	452	2	447
Q08S20	3TA9:A	51.95	1196	458	34	458
A0A0N0H8Q2	1GNX:A	56.30	1151	479	18	470
A0A0N0HQN8	1NP2:A	50.67	929	436	6	426

A0A0Q5JYC8	3WH5:A	53.99	1171	457	6	443
A0A0Q6RZ82	3WH5:A	49.31	1072	457	2	433
E3FSZ5	1VFF:A	37.20	693	423	5	408
Q63F48	3AHX:A	34.16	618	453	1	441
E3FN28	4PTV:A	52.71	1288	452	2	452
E0RA06	2O9P:A	91.72	2242	454	8	454
A0A0N0Y7R6	1GNX:A	90.36	2223	479	1	477
A0A0N0HTJ2	3WH5:A	55.20	1205	457	6	446
A0A0N1K8N4	1GNX:A	65.74	1618	479	9	477
Q1J2J3	3WH5:A	56.29	1207	457	6	440
A0A083XQA5	1GNX:A	48.59	1109	479	16	470
E0RE68	1BGG:A	97.32	2380	448	1	448
A0A0Q6SCD7	1QOX:A	48.74	1067	449	5	439
E0RL55	4B3K:A	46.52	1124	479	3	459
A0A0K8PDQ2	1NP2:A	51.24	953	436	8	430
A0A0Q8DHT9	1OD0:A	44.70	947	468	27	464
A0A0K8PEI1	1GNX:A	58.13	1204	479	17	470
E0RJV2	1OD0:A	34.58	618	468	23	465
A0A0K8PQB4	1GNX:A	68.74	1658	479	12	477
A0A068HD71	3TA9:A	46.65	1055	458	13	450
E0RCR1	1BGG:A	32.14	602	448	1	442
E0RB30	4B3K:A	39.70	956	479	1	463
G7M6D5	3AHX:A	45.63	1006	453	4	446
W4V2R1	3TA9:A	55.28	1363	458	8	451
A9BFQ9	1OD0:A	67.19	1605	468	26	466
Q21EM1	4GXP:A	48.92	1181	467	10	465
W1EPA2	4B3K:A	45.04	1067	479	3	463
Q8ES64	4B3K:A	41.08	989	479	1	461
Q8EMS8	4B3K:A	49.13	1219	479	3	459
A0A086W5P1	3WH5:A	52.04	1137	457	6	443
A0A154MSG3	3TA9:A	44.49	999	458	8	450
W1EMD8	3AHX:A	35.63	129	453	3	86
I4B8U7	3CMJ:A	44.98	970	465	30	459
I0UW37	1UYQ:A	33.26	595	447	4	444
E0Q968	1GNX:A	52.95	1158	479	17	469
A0A0S7AN66	1OD0:A	48.30	1042	468	28	463
A0A154MUI7	1GNX:A	48.27	1052	479	18	477
W1EPJ8	4B3K:A	42.97	834	479	96	470
A0A154MP58	3WH5:A	52.49	1184	457	6	446
A0A0S7ALL5	3WH5:A	60.91	1361	457	1	440
A0A154MPI7	3W53:A	54.47	1246	506	45	502
W0LJ28	3TA9:A	42.61	1002	458	13	456
Q21KX3	2JIE:A	42.82	978	454	18	451
A4C562	3TA9:A	45.75	1033	458	16	450
A0A086WCG9	3WH5:A	49.10	1063	457	5	449
F9LWP8	3AHX:A	27.78	326	453	51	403
W1EU18	4PTV:A	47.32	1072	452	7	444
A0A0Q9P6A8	1OD0:A	50.45	1135	468	27	465
U6FEB3	2E9L:A	35.71	55	469	288	314
X7F492	1OD0:A	46.08	1014	468	21	453
C7D674	1OD0:A	42.86	975	468	21	453
W9G2W6	3WH5:A	58.51	1266	457	6	433
W1KV84	1OD0:A	44.57	1036	468	25	466
M2PZ93	3TA9:A	44.04	1000	458	8	450
B5JPY7	4GXP:A	49.89	1209	467	10	465
F4BL34	1BGG:A	33.76	630	448	1	442
W9FVS7	1GNX:A	57.08	1175	479	22	472

F0I9D9	1BGA:A	65.62	116	447	4	35
K8CTC7	4B3K:A	41.14	967	479	1	470
W9FS44	1GNX:A	67.75	1608	479	16	476
M2Q4V4	3W53:A	48.36	1071	506	46	499
K8C913	3CMJ:A	32.64	255	465	242	464
M2NRX8	3W53:A	54.78	1244	506	45	502
A0A0M0KJQ7	1QOX:A	62.36	1577	449	1	449
A0A0T9Q4C1	3TA9:A	42.42	998	458	13	451
A0A0P8W829	1VFF:A	34.14	607	423	5	402
A0A0T9TWD4	1UYQ:A	29.58	566	447	5	441
W9FXL9	3ZJK:A	47.43	896	431	8	431
K8CVG7	4B3K:A	47.72	1137	479	2	459
A0A0Q4TNS4	1OD0:A	45.05	1009	468	17	466
W9FP75	1GNX:A	69.36	1673	479	11	477
M2NN97	1NP2:A	54.16	1096	436	8	428
C7RFE4	5FOO:A	48.47	1152	480	1	457
A0A0D6SDX6	1OD0:A	45.02	1037	468	25	462
B5JPZ5	4GXP:A	52.36	1232	467	10	466
W1KXX8	1QOX:A	46.83	1036	449	5	443
F4BLB9	1OD0:A	38.53	729	468	25	466
D6X9X8	1GNX:A	57.05	1296	479	10	477
A0A0M2J903	1GNX:A	67.67	1641	479	12	477
A0A0Q4LI65	1OD0:A	40.54	859	468	28	463
A0A0N0T5H1	3TA9:A	50.88	1167	458	13	450
B5HHQ0	3WH5:A	56.66	1236	457	6	445
A0A0M2J855	3WH5:A	55.78	1219	457	6	445
B5HAV3	1GNX:A	79.20	1952	479	1	476
A0A0W1KWY9	3TA9:A	41.46	950	458	12	450
A0A0C1Y8K3	1GNX:A	56.72	1196	479	6	472
D6Y646	1GNX:A	52.18	1051	479	22	474
A0A0C2BEE0	1GNX:A	76.51	1870	479	14	477
D6Y6J1	1GNX:A	57.84	1296	479	4	472
A0A0W8IMR8	3W53:A	56.99	1382	506	40	506
M3VGR2	3W53:A	44.59	904	506	46	503
F7RR92	2JIE:A	42.17	920	454	37	449
F4H7E6	1GNX:A	60.17	1404	479	7	479
Q99Z97	4B3K:A	45.10	1060	479	3	459
D6YBP6	1GNX:A	55.72	1318	479	15	477
D6YBP8	3TA9:A	48.88	1110	458	13	456
A0A0W1KX11	3AHX:A	43.53	1002	453	1	441
A0A117M3L0	1OD0:A	66.82	1604	468	26	467
B5H6C9	3ZJK:A	48.76	928	431	8	427
A0A0N1L3X6	1OD0:A	43.69	955	468	28	466
D6Y5B2	1GNX:A	54.84	1151	479	4	466
A0A0A6VUV8	3W53:A	67.83	1685	506	45	501
B5FAU8	3AHX:A	42.33	930	453	4	440
A0A109J7P9	1QOX:A	47.96	1071	449	5	444
A0A0M6YSW6	1QOX:A	48.18	1036	449	5	442
H5WVY6	4PTV:A	49.20	1141	452	7	445
A0A0C2BIK2	3WH5:A	56.75	1233	457	6	441
A0A0G9HBF3	1OD0:A	49.55	1117	468	27	465
F4XR96	4HZ6:A	42.67	924	444	4	440
A0A0M8WHP2	3WH5:A	56.75	1226	457	11	446
A0A150M9Y2	1QOX:A	36.84	689	449	3	449
A0A0M8VZE5	1NP2:A	50.34	919	436	8	430
A0A0L7BTV1	1GNX:A	53.59	1175	479	16	467
A0A0M8WYZ7	1GNX:A	68.25	1645	479	16	477

A0A0N0A8X6	1GNX:A	69.52	1726	479	1	477
A0A0B9AI87	3TA9:A	42.79	993	458	13	450
M0Q9T2	3TA9:A	44.18	1053	458	13	456
A0A0M2JM03	1NP2:A	49.67	913	436	8	430
V6UK54	1GNX:A	67.75	1608	479	16	476
E0SEG6	1UYQ:A	32.44	585	447	5	441
A0A0Q4FAZ5	1GNX:A	61.80	1407	479	13	477
A0A0G3BNS0	3TA9:A	48.08	1076	458	16	457
A0A0M2N3D1	3TA9:A	44.13	1017	458	13	452
V6UJ24	3WH5:A	58.51	1264	457	6	433
A0A0T6YV65	1QOX:A	46.83	1028	449	5	444
A0A0C2ALF5	1GNX:A	70.59	1754	479	6	479
E0SEG5	1BGG:A	32.47	599	448	1	442
A0A0J5QXF6	5DT5:A	64.04	1618	471	24	467
A0A0D7DCY0	3CMJ:A	49.78	1089	465	21	458
B7JD71	1QOX:A	38.86	808	449	5	443
E0SEH6	4B3K:A	40.61	947	479	1	455
F9TCL2	2O9P:A	45.37	1054	454	18	449
A0A0F0LTP0	3W53:A	54.27	1261	506	45	500
A0A0D7D5G2	1GNX:A	66.67	1604	479	2	476
A0A0C2JEX1	1GNX:A	57.14	1284	479	23	477
A0A0C2AUK0	3WH5:A	55.88	1218	457	6	446
A0A0J5TLL7	1QOX:A	62.79	1529	449	5	442
V6UKL6	3ZJK:A	47.43	897	431	8	431
A0A0D7D1B2	1GNX:A	69.79	1681	479	11	477
A0A0F0M3K8	1GNX:A	62.26	1398	479	16	473
A0A0C1WWF2	1GNX:A	52.54	1081	479	17	467
A0A0D7DDZ0	3WH5:A	58.51	1267	457	6	433
A0A0C2JA10	3TA9:A	47.47	1117	458	13	452
A0A0G3BK76	1QOX:A	57.27	1386	449	1	441
A0A066Z0U4	4HZ6:A	46.86	1014	444	4	440
A0A136PT97	3CMJ:A	50.22	1167	465	16	463
A0A0N0YNN0	1GNX:A	70.97	1713	479	18	479
A0A0A5GAG4	1BGG:A	56.28	1402	448	1	445
D7C5W5	3WH5:A	55.88	1248	457	6	446
A0A0Q8AIT6	3W53:A	54.47	1292	506	44	501
A0A136PQ79	1GNX:A	58.62	1276	479	16	475
A0A101SYK5	1GNX:A	68.94	1673	479	10	478
A0A136PLI8	1GNX:A	62.45	1379	479	16	472
I7LD22	4PTV:A	29.88	519	452	1	448
A0A0A5GRV1	3TA9:A	55.30	1308	458	11	452
D7C0L6	1GNX:A	53.66	1142	479	11	470
A0A136PR14	3WH5:A	52.48	1070	457	6	444
A0A0A0BS35	1GNX:A	65.59	1499	479	17	476
A0A0A0BTI4	3W53:A	52.68	1228	506	42	503
A0A0N0YGY9	1GNX:A	53.56	1119	479	17	470
A0A0M8UI27	3WH5:A	54.55	1172	457	6	444
A0A117R8V6	1NP2:A	49.55	912	436	8	430
D7C1E5	1GNX:A	71.24	1721	479	16	479
A0A0M9Z7X1	1GNX:A	65.46	1527	479	9	476
A0A0F2CIZ5	4B3K:A	47.51	1147	479	1	459
A0A0U3KJP0	3ZJK:A	49.01	1023	431	3	431
A0A0N0HMH4	1NP2:A	47.56	914	436	8	434
E1LQA9	5FOO:A	48.28	1170	480	1	460
A0A136PSV5	1GNX:A	65.38	1529	479	15	478
L0M412	4PTV:A	45.51	1047	452	7	444
A0A139N452	5FOO:A	46.32	1108	480	1	460

B7QXQ6	1OD0:A	43.72	964	468	26	453
A0A0N0YI18	1GNX:A	55.51	1183	479	12	472
D6AQT5	1GNX:A	57.08	1175	479	22	472
D6ABL3	1GNX:A	67.75	1608	479	16	476
A0A0Q4GK27	3W53:A	41.19	881	506	47	492
D6ABM2	3ZJK:A	47.43	897	431	8	431
A9VS04	3AHX:A	41.38	918	453	4	441
U3UZV3	5DT5:A	40.68	91	471	418	470
Q38WG1	4B3K:A	43.51	1003	479	1	459
D5BF76	1OD0:A	43.86	979	468	28	463
J9A1G5	1OD0:A	45.96	1005	468	32	462
U0FGF9	3ZJK:A	49.23	1022	431	3	431
A0A0K2ART6	1NP2:A	51.11	919	436	6	430
K6TJ93	3AHX:A	43.75	1015	453	4	441
A0A0K4AQD0	4B3K:A	49.02	1184	479	3	459
D6AEP8	1GNX:A	69.36	1673	479	11	477
A0A0P4UKT9	1OD0:A	43.61	930	468	27	464
A4WRN3	4PTV:A	42.92	950	452	1	430
A5FAA5	1OD0:A	43.44	1007	468	24	461
A0A0K2ASA3	1GNX:A	65.74	1621	479	9	477
A0A087M1I5	1OD0:A	47.73	1061	468	26	464
A0A0K4TBV5	1OD0:A	50.00	149	468	138	189
D6AI79	3WH5:A	58.51	1264	457	6	433
E0RQX8	1OD0:A	48.17	1098	468	28	464
A3JYS5	1OD0:A	45.52	1013	468	21	453
A0A150KUW4	3TA9:A	35.88	629	458	13	450
S5S0W9	1QOX:A	48.05	1055	449	5	439
A0A150L0E6	4PTV:A	39.35	867	452	7	445
A0A0Q5LN16	3W53:A	55.63	1293	506	44	501
N6V0Q4	1QOX:A	46.83	1052	449	5	444
A0A099LJ91	3TA9:A	45.60	1025	458	12	454
I9NFG2	1QOX:A	47.74	1054	449	5	444
A0A0D0FQS3	4B3K:A	51.20	1254	479	3	459
A0A0D1MRA2	1OD0:A	46.56	1025	468	21	466
K0YU62	3W53:A	49.35	1098	506	46	492
A0A0Q5LYA3	1GNX:A	61.74	1399	479	14	470
A0A0D0F5F3	4B3K:A	50.98	1254	479	3	459
A0A0T2MCU7	1OD0:A	46.14	1026	468	26	464
B9KSP3	4PTV:A	45.94	1000	452	1	430
X4ZXV2	1QOX:A	64.17	1566	449	5	445
B8E1X9	1VFF:A	43.53	882	423	4	416
B1XZK8	3TA9:A	46.41	1049	458	15	452
A0A072S7E3	3WH5:A	56.49	1213	457	6	443
A0A0Q5QEF8	3W53:A	54.27	1259	506	45	500
G0PVZ2	1GNX:A	51.63	1068	479	18	472
A0A0M9YP81	1GNX:A	68.18	1614	479	16	476
G0Q2R3	3WH5:A	56.57	1266	457	6	450
G0PQW9	1VFF:A	34.88	592	423	1	408
G0Q381	1GNX:A	68.61	1610	479	16	475
A0A0L7CC49	1GNX:A	49.25	1110	479	16	475
A0A076K4F3	1OD0:A	43.93	972	468	28	453
G0PUU1	3ZJK:A	48.88	913	431	8	430
G0Q8E5	1GNX:A	70.21	1675	479	11	477
A0A072SFZ8	1GNX:A	66.24	1621	479	9	477
A0A072S5G8	1GNX:A	90.81	2245	479	1	479
G0Q482	1GNX:A	56.99	1175	479	18	472
A0A0M8T2X6	1GNX:A	69.28	1663	479	11	479

A0A072SIA6	1NP2:A	51.36	928	436	8	423
W0IHU8	1QOX:A	47.06	1042	449	5	444
A0A0N0SHM4	3ZJK:A	47.53	898	431	8	430
B8E0Q1	3TA9:A	57.68	1410	458	8	456
A0A0Q8IYW4	1OD0:A	51.45	1162	468	26	465
G0FZ95	1GNX:A	53.22	1153	479	9	472
A0A142HV74	1OD0:A	44.80	974	468	26	462
D7VPW1	1OD0:A	45.10	1033	468	28	462
A0A0M3T701	1GNX:A	51.95	1064	479	44	477
A0A0Q4RZP5	1OD0:A	42.76	992	468	24	461
E8X6A1	1OD0:A	45.55	1017	468	13	464
R4YH72	3TA9:A	47.10	1062	458	13	450
A0A0D7Q7W0	3WH5:A	45.41	920	457	3	441
I1E031	3TA9:A	44.29	990	458	12	449
A0A0Q2Y808	3AHX:A	42.41	987	453	1	445
G0G5T9	4PTV:A	44.94	994	452	2	444
F4AIE0	3TA9:A	45.91	1024	458	16	455
A0A0M0ILU9	2O9P:A	45.14	1047	454	18	449
G0FRP6	1GNX:A	53.43	1122	479	16	478
G0FQQ5	3W53:A	53.70	1235	506	45	502
A0A0Q8W087	1GNX:A	60.17	1400	479	5	477
H1Y3B9	1OD0:A	45.17	980	468	24	462
A0A077EM06	1OD0:A	43.95	1028	468	21	462
D7B2H4	1NP2:A	53.79	1019	436	8	427
A0A0M7BCL0	2JIE:A	42.92	936	454	14	451
A9L506	3CMJ:A	42.04	957	465	15	458
F4C2M4	1OD0:A	45.33	980	468	24	457
A0A101PJZ1	3WH5:A	55.93	1251	457	6	449
F8L746	3CMJ:A	30.51	452	465	24	458
B8D213	1VFF:A	36.18	737	423	1	416
D7AZG0	1GNX:A	57.36	1272	479	18	477
A0A135IMR7	3AHX:A	42.57	963	453	6	445
A0A0Q8VXQ9	3W53:A	57.95	1340	506	45	499
A0A0Q8VXC8	1GNX:A	49.56	1010	479	15	467
J2ZSL2	1UYQ:A	35.27	616	447	5	441
Q13A13	3TA9:A	43.64	965	458	16	454
A0A0B8NYV8	3TA9:A	45.77	1033	458	16	450
U2ZB65	1OD0:A	50.56	1123	468	27	465
A0A117Q2M6	1GNX:A	66.38	1627	479	12	477
A0A0B8NWC0	4B3K:A	46.64	1132	479	1	459
A0A087AH51	1GNX:A	54.13	1177	479	16	467
A0A117Q6Z6	1NP2:A	51.12	973	436	8	430
A0A101P7I9	1GNX:A	63.38	1435	479	16	477
B8CYA8	4PTV:A	100.00	2413	452	2	452
D7AXP9	1GNX:A	52.88	1084	479	17	467
F0SBK6	1OD0:A	45.48	1012	468	28	462
A0A0Q4H9L6	3W53:A	55.82	1312	506	45	505
A0A0D8KR32	1OD0:A	44.12	1022	468	25	462
W9EAC3	5DT5:A	52.25	1292	471	24	463
A0A0H2KNB6	1GNX:A	62.13	1485	479	7	478
Q82CT5	1GNX:A	67.51	1665	479	4	479
A0A0Q4H829	4HZ6:A	43.74	984	444	2	440
S5C7J4	2O9R:A	42.98	977	452	8	452
A0A0X3WSQ3	3WH5:A	55.88	1221	457	6	446
W9VIM8	3TA9:A	48.18	1100	458	13	452
J2VR00	3TA9:A	43.30	917	458	9	455
Q82M59	3WH5:A	56.11	1239	457	6	446

E0RXJ5	1VFF:A	31.14	522	423	4	395
Q82BV6	1NP2:A	49.55	910	436	8	426
A0A0Q7EUW1	3TA9:A	47.42	1108	458	13	452
A0A0T1UM92	1GNX:A	67.88	1637	479	12	476
I4VWU8	1OD0:A	44.70	954	468	27	464
A0A0T1ULF9	1NP2:A	50.90	939	436	7	430
A0A0X3XDC3	1GNX:A	71.91	1747	479	16	479
A0A0Q4HCT3	1GNX:A	60.30	1407	479	13	479
W9EEC0	5DT5:A	55.38	1348	471	26	461
A0A0T1U9U5	3WH5:A	56.82	1249	457	6	444
L5N1X7	1QOX:A	57.40	1381	449	5	443
Q1GM35	1OD0:A	44.76	984	468	26	453
I0GV58	1UYQ:A	31.91	599	447	3	441
A0A0R2SN20	3TA9:A	49.21	1190	458	15	453
A0A0H3CV54	4PTV:A	44.94	994	452	2	444
A0A087DDN9	1GNX:A	51.69	1214	479	16	477
A0A0H3DGN3	1GNX:A	53.22	1153	479	9	472
W1DXI4	3TA9:A	54.26	774	458	13	270
A0A0Q7BKB2	1GNX:A	50.22	1023	479	16	469
A5EJ08	1OD0:A	42.63	941	468	26	467
A0A074J7B8	1NP2:A	49.08	982	436	2	426
W1DNR7	3AHX:A	35.63	129	453	3	86
W1DLQ7	5FOO:A	37.16	353	480	198	412
A0A0M2Z050	1NP2:A	49.89	939	436	6	430
A0A147KBW9	5DT5:A	68.18	1702	471	25	463
A0A0Q9T5A1	1GNX:A	58.44	1338	479	5	477
W1DNG5	4B3K:A	51.31	550	479	3	193
A0A0H3D0W0	3W53:A	48.27	1036	506	44	503
A0A0Q7BHY9	3TA9:A	45.45	1003	458	13	450
A0A0M2Z648	3WH5:A	56.79	1235	457	6	446
W1DQF6	4B3K:A	42.97	836	479	96	470
A0A0M2YZ00	1GNX:A	70.97	1713	479	17	479
A0A0M2YVG2	1GNX:A	69.46	1679	479	16	479
A0A0H3DI81	1GNX:A	53.43	1122	479	16	478
A5EQE2	3TA9:A	43.45	959	458	17	450
A0A086N873	1GNX:A	90.61	2223	479	1	479
A0A086N2K8	1GNX:A	66.17	1620	479	12	477
A0A0J1FMP4	3TA9:A	47.60	1060	458	12	443
A0A0Q8DC24	1OD0:A	50.67	1169	468	23	465
A0A0Q9T503	2O9P:A	46.07	994	454	13	452
A0A086N6Y5	1GNX:A	58.08	1174	479	18	470
A0A0M0TGQ2	1OD0:A	43.67	1020	468	24	461
W1DVS7	3CMJ:A	48.06	297	465	335	462
A0A0H3D8I7	3W53:A	53.70	1235	506	45	502
Q746L1	3ZJK:A	99.77	2220	431	1	431
A0A0R3CQ33	1OD0:A	41.29	900	468	24	465
D6KER2	3WH5:A	55.68	1230	457	6	444
A0A0Q5FWL5	1GNX:A	62.61	1419	479	14	470
A0A0A7K2D1	1OD0:A	41.50	931	468	26	462
L9P9G7	3TA9:A	47.54	1059	458	10	452
H6SRJ1	4PTV:A	44.65	957	452	7	444
A0A0W8I5T1	3W53:A	52.90	1182	506	39	496
D6K1J5	1GNX:A	71.03	1734	479	14	477
L9PHR2	4PTV:A	46.36	1016	452	7	446
L9PE61	3TA9:A	47.15	1096	458	13	451
D8FNI9	1UYQ:A	30.37	586	447	4	441
D1CGH4	3TA9:A	55.16	1351	458	1	455

E4Q361	3AHX:A	52.63	1236	453	4	444
A0A0Q7RUG0	3W53:A	54.24	1259	506	36	505
A0A127D520	3TA9:A	41.13	927	458	13	451
D6K754	3WH5:A	54.55	1216	457	6	444
A0A0Q5FPQ8	3W53:A	54.99	1263	506	44	501
A0A087E771	1GNX:A	53.12	1159	479	17	471
L8DCH1	2O9P:A	44.70	1000	454	10	450
R1IFP3	3W53:A	52.17	1204	506	45	502
G5RMN3	4B3K:A	50.37	356	479	73	207
A3CN02	3AHX:A	41.69	849	453	5	441
F5Z6I3	3TA9:A	45.05	1007	458	15	458
A0A120F862	1GNX:A	61.62	1349	479	17	472
A0A0M9YNV5	1GNX:A	69.55	1662	479	18	479
K9CYL1	1OD0:A	44.59	979	468	28	466
A0A0Q8VGX6	3W53:A	53.91	1260	506	43	503
G5RMN4	4B3K:A	49.79	633	479	221	459
G2DX76	4PTV:A	47.73	1083	452	7	446
A0A109II49	1GNX:A	56.59	1261	479	18	475
A0A0Q8VS90	4HZ6:A	47.30	1033	444	2	440
C5BYX0	3W53:A	54.49	1262	506	45	498
A0A084U7W6	1OD0:A	47.15	1048	468	28	462
R1FKF0	4HZ6:A	46.50	972	444	4	443
A0A0G0GD78	1VFF:A	37.80	718	423	3	411
R1GBW0	3TA9:A	45.07	993	458	8	450
F5ZGD5	3TA9:A	43.99	989	458	12	450
A0A120F783	1NP2:A	55.66	1079	436	8	428
G5RMN2	4B3K:A	40.00	167	479	3	80
A0A011AHF0	3WH5:A	52.85	1163	457	6	444
R1GAE9	3CMJ:A	47.35	1058	465	23	459
A0A0Q8WCX9	1GNX:A	58.11	1314	479	18	472
A0A0M8SMA1	3WH5:A	55.58	1232	457	6	434
D9T3K2	4HZ6:A	52.70	1205	444	4	442
A0A010ZQC5	3CMJ:A	49.44	1117	465	25	459
A0A120F8N8	4PTV:A	49.89	1163	452	1	445
A0A011AM20	1GNX:A	62.34	1426	479	16	476
D9SZB9	1GNX:A	57.88	1287	479	16	475
D9T8D4	1NP2:A	55.23	1097	436	1	428
A0A0E4A6R5	3ZJK:A	49.23	1029	431	3	431
R1I2E4	1GNX:A	52.27	1112	479	16	476
D9TCC7	1GNX:A	56.58	1226	479	17	472
A0A0M8SEZ4	1NP2:A	50.78	1007	436	6	431
A0A134DEC1	3W53:A	54.79	1277	506	36	502
D9SZT8	1GNX:A	59.48	1299	479	15	477
A0A0F7PCX2	1OD0:A	46.59	1067	468	27	464
F7ZA00	1OD0:A	43.82	967	468	26	453
A0A0F4W6W8	4B3K:A	58.82	1523	479	1	459
A0A011SVF9	3WH5:A	49.44	1071	457	2	447
A0A0F4RJA0	3TA9:A	44.94	974	458	13	436
A0A0G1NE73	1VFF:A	40.80	752	423	1	404
A0A0F4WR38	3AHX:A	41.90	971	453	4	441
U7QKM3	1OD0:A	43.79	943	468	27	464
F8I8K7	3TA9:A	55.43	1323	458	13	452
A0A0Q7HY00	1GNX:A	56.99	1183	479	18	472
A0A0D5BTJ1	1OD0:A	44.62	1045	468	21	462
A0A0Q7HH44	3WH5:A	56.47	1261	457	6	449
A0A0Q7I6P6	1GNX:A	68.10	1607	479	14	476
F8IE65	4PTV:A	56.11	1357	452	7	447

X6L011	1OD0:A	43.82	986	468	21	463
A0A0Q8YFH8	3W53:A	56.18	1319	506	46	503
A0A0D8HFJ3	1GNX:A	51.39	1204	479	13	477
A0A0Q7HBJ5	1GNX:A	69.79	1669	479	11	477
A0A0Q7J164	3WH5:A	45.39	906	457	6	446
A0A0T9T467	3TA9:A	43.20	1011	458	13	452
Q1J655	4B3K:A	45.10	1061	479	3	459
A0A0D2WNN4	3AHX:A	40.91	947	453	3	439
Q1J5L4	4B3K:A	97.70	2526	479	1	479
A0A0D2VQR6	1GNX:A	49.24	1074	479	18	477
A0A0Q7HUD3	3CMJ:A	48.65	1077	465	21	458
A0A0W1ND10	1GNX:A	66.03	1635	479	9	477
R9U0C2	1QOX:A	37.63	776	449	1	443
A0A0Q7TCD8	1BGG:A	75.39	1942	448	1	447
A0A0C5FZ74	3WH5:A	53.60	1208	457	6	448
A0A0W1NKP4	1GNX:A	90.81	2235	479	1	479
A0A069JV67	3WH5:A	54.20	1199	457	6	445
A0A0Q8F9X3	1GNX:A	44.56	898	479	18	477
A0A147DHV1	5DT5:A	89.96	2239	471	24	471
A0A0C5FQ82	1GNX:A	68.53	1661	479	15	477
A0A069JRR8	1GNX:A	70.40	1725	479	8	477
A0A147DK95	3AHX:A	40.69	958	453	4	440
F6CUI3	4HZ6:A	47.27	1061	444	3	439
A0A024YMJ6	1GNX:A	64.43	1502	479	16	476
V6FP83	1QOX:A	34.68	604	449	5	449
R5N4U6	1OD0:A	45.66	1059	468	28	465
R9TRH3	1QOX:A	43.82	171	449	360	442
A0A0C3EDV1	3TA9:A	46.82	1085	458	12	450
A0A0W1NQW9	1GNX:A	56.77	1158	479	17	467
A0A031FTF5	3W53:A	52.65	1238	506	36	502
F5JK61	1OD0:A	44.34	1027	468	25	462
D0X5D9	2O9P:A	45.14	1040	454	18	449
A0A0J7XQ01	1OD0:A	44.37	970	468	28	466
A0A0W1NHN6	1NP2:A	49.89	913	436	6	432
H0HC94	1QOX:A	46.03	1034	449	5	443
A0A069JXB6	1GNX:A	54.22	1170	479	1	472
R9U0D6	1UYQ:A	33.97	654	447	4	441
A0A0C5FSX0	1GNX:A	56.99	1164	479	18	470
A0A0C5FZ69	1GNX:A	55.36	1146	479	18	471
A0A069K8M0	1GNX:A	54.00	1123	479	17	470
K2LMK5	1OD0:A	45.96	1057	468	25	466
A0A0Q7SPH8	2O9P:A	69.80	1819	454	8	454
A0A0L7CUF9	1GNX:A	53.81	1178	479	16	467
U4ZZN7	2O9R:A	44.97	1087	452	8	447
A0A0C5FQ81	1GNX:A	69.65	1734	479	1	479
A0A0W1NGA5	3WH5:A	55.20	1203	457	6	446
A0A097R6G4	4PTV:A	44.32	1015	452	7	444
A0A0B9A8W4	3W53:A	43.94	891	506	51	504
A0A069JY86	3TA9:A	47.12	1097	458	13	448
B5WE49	3TA9:A	48.50	1084	458	12	443
A0A0Q7DXY7	3TA9:A	44.06	1000	458	11	455
A0A0B5QJW2	3AHX:A	44.68	1017	453	4	446
C2K0R7	1OD0:A	33.06	606	468	28	465
R1CUK8	5DT5:A	60.32	1490	471	25	463
Q5XAU7	5FOO:A	97.92	2536	480	1	480
A0A0H5D0V7	1OD0:A	43.49	962	468	26	453
A0A0C2RQE4	3TA9:A	41.99	947	458	13	451

L7LMB8	3W53:A	44.03	922	506	46	503
Q5XBM4	4B3K:A	45.10	1060	479	3	459
A0A0R3MMQ8	1OD0:A	41.10	903	468	18	466
A0A0L8M4U2	1GNX:A	64.71	1497	479	16	474
A0A0Q8VA65	3W53:A	69.74	1730	506	41	503
A0A0R3M9W1	3TA9:A	43.71	924	458	13	455
A0A0Q7E0M4	1OD0:A	46.21	1015	468	32	464
A0A087IJD4	2O9P:A	45.16	1045	454	16	449
A0A142HL66	1OD0:A	42.02	912	468	28	461
A0A0F3FR95	3AHX:A	43.59	995	453	4	445
C2JTN8	4B3K:A	46.73	1055	479	3	427
B5WNC7	4PTV:A	49.67	1145	452	2	446
D5WLR1	3TA9:A	49.89	1148	458	8	452
U2YMR8	3W53:A	53.44	1278	506	34	500
D5WGC6	4PTV:A	47.32	1093	452	6	452
C6Y1A3	1OD0:A	40.63	885	468	28	462
A0A100VQ06	1BGG:A	75.39	1939	448	1	447
K5VIP2	4PTV:A	45.68	1038	452	2	444
F4NJN0	3TA9:A	34.05	633	458	12	450
F4NPV6	1QOX:A	34.68	605	449	5	449
Q2K441	1QOX:A	47.83	1047	449	5	439
A0A021X5U5	1OD0:A	44.19	986	468	27	461
G6FBI5	3AHX:A	34.10	598	453	4	440
A0A0C2L810	3CMJ:A	42.70	950	465	21	461
G6F9T1	3AHX:A	41.88	878	453	6	441
F0LAM7	1QOX:A	46.26	1039	449	5	443
T0PWK0	1OD0:A	44.39	1038	468	21	462
Q6MSD6	5FOO:A	41.89	1021	480	1	456
G6FAV8	4B3K:A	55.19	1428	479	1	461
G6FFS4	1UYQ:A	31.19	581	447	4	441
I2GLH4	1OD0:A	41.80	913	468	27	461
A0A085AL28	3TA9:A	44.66	996	458	13	450
A0A0C1JFQ6	1OD0:A	45.29	974	468	21	453
A0A109YN89	1VFF:A	31.31	538	423	6	408
A0A087CV22	1GNX:A	53.22	1232	479	16	477
A0A0G0SN72	1VFF:A	39.31	691	423	4	406
A0A0Q9U494	3W53:A	54.91	1304	506	39	503
A0A098EWY7	1BGG:A	58.30	1505	448	1	445
D6A0U2	1GNX:A	67.30	1639	479	9	477
A0A0M0KQU0	5DT5:A	75.34	1909	471	24	461
A0A0N1NBL9	1GNX:A	72.84	1747	479	14	476
A0A0D8FUW7	3TA9:A	47.85	1161	458	11	456
D6A5X6	3WH5:A	55.81	1203	457	6	443
A0A068SUC0	1OD0:A	46.01	1063	468	28	462
A0A0N1H0N8	3ZJK:A	52.35	1007	431	7	426
D6A2J4	1GNX:A	87.89	2189	479	1	479
A0A0Q8N452	1QOX:A	46.83	1053	449	5	444
A0A0N1G7G6	1QOX:A	47.32	1062	449	5	443
A0A0N1GIB1	1GNX:A	66.67	1552	479	8	477
V6L2T1	1GNX:A	69.65	1643	479	23	479
A5KN03	1E4I:A	29.71	575	447	5	441
D6A3T6	1NP2:A	51.22	912	436	8	432
A0A0D0MJ01	1OD0:A	47.30	1025	468	28	463
J0P229	3AHX:A	34.73	607	453	5	440
A0A0U3QTC1	1NP2:A	50.56	931	436	8	430
A0A0U3P0Q0	1GNX:A	66.38	1621	479	7	477
A0A087CJA9	1VFF:A	33.91	563	423	5	395

J1HX71	5FOO:A	39.00	897	480	1	456
A0A0T9T6C9	1UYQ:A	29.38	551	447	5	441
Q5FJD3	3AHX:A	40.55	872	453	6	440
A0A0U3PI65	3WH5:A	55.88	1246	457	6	446
T5I764	3ZJK:A	49.45	1036	431	3	431
A0A0U3QV55	1GNX:A	80.00	1990	479	1	478
J1HVE4	4PTV:A	34.26	602	452	2	444
A0A0D0MCX8	3WH5:A	60.68	1355	457	1	440
A3PJW4	3TA9:A	46.35	994	458	13	436
A0A119CYV6	3AHX:A	46.44	1015	453	8	441
A0A0P6XZW3	3TA9:A	53.32	1260	458	8	455
A9D1X4	1OD0:A	46.49	1071	468	25	461
A0A150LYB2	4B3K:A	49.13	1223	479	3	459
A0A010NF87	3W53:A	54.01	1247	506	44	500
A0A0C5BBK0	3W53:A	65.94	1619	506	45	502
S5ASB4	2JIE:A	42.95	1013	454	13	449
A0A150LXU1	1QOX:A	38.15	783	449	1	443
A0A0B3BP14	5DT5:A	54.63	1389	471	24	464
A0A059GAD1	3CMJ:A	45.45	1006	465	26	462
A0A0L8QR02	1GNX:A	70.43	1697	479	17	476
I7IVF0	3CMJ:A	30.45	566	465	24	464
F5U4N6	1UYQ:A	35.04	635	447	5	441
A0A0Q7GQM0	1OD0:A	45.06	1028	468	27	457
S5AK99	2JIE:A	43.21	982	454	10	454
A0A095TZ25	1OD0:A	40.54	899	468	18	457
A0A0M1NP20	3TA9:A	41.56	928	458	13	451
D7CRB8	3TA9:A	52.05	1233	458	13	450
R7XT60	4PTV:A	44.42	994	452	7	451
U3VF21	5DT5:A	40.68	91	471	418	470
A0A0Q9NU55	3W53:A	57.20	1346	506	36	502
A0A081PGZ2	1OD0:A	40.68	918	468	28	462
A0A0Q9NW18	1OD0:A	42.52	934	468	25	462
H6CD97	4B3K:A	40.60	972	479	3	462
Q48SY1	4B3K:A	45.10	1060	479	3	459
A0A0Q9DSX3	1GNX:A	61.52	1395	479	14	470
Q1JKS5	4B3K:A	98.75	2554	479	1	479
A0A0D0EF31	1OD0:A	43.21	1011	468	24	461
H6CRB9	4B3K:A	45.22	1102	479	3	459
U2KX49	1OD0:A	42.19	984	468	27	465
A0A0Q9DF12	3W53:A	54.87	1267	506	44	501
A0A0Q2UU82	3W53:A	45.65	984	506	45	500
W0HUE2	1OD0:A	45.29	1019	468	26	467
A0A0X8RL12	3W53:A	54.53	1246	506	44	501
A0A0Q8Q0Z9	1GNX:A	65.34	1580	479	7	477
H6CP27	1BGG:A	96.65	2356	448	1	448
H6CNP0	1UYQ:A	32.63	625	447	2	441
Q48S46	4B3K:A	98.75	2548	479	1	479
E1IGK6	3CMJ:A	54.71	1289	465	22	463
Q1JLA4	4B3K:A	45.10	1060	479	3	459
A0A0Q8PYN3	1GNX:A	67.46	1559	479	18	477
A0A0Q8Q1K8	3WH5:A	56.56	1261	457	6	446
A0A0Q8PTI6	1GNX:A	66.45	1581	479	17	476
A0A0Q8PNU2	1QOX:A	47.77	1087	449	5	443
A0A0J5JMB8	1BGG:A	60.85	1577	448	1	446
A0A150REQ2	1OD0:A	46.55	1047	468	26	465
A0A0F2D7W6	4B3K:A	48.16	1165	479	1	459
A0A0S3AS57	3TA9:A	45.60	1026	458	12	454

A3WL20	3CMJ:A	46.30	986	465	30	452
C6AWP7	1QOX:A	47.06	1041	449	5	444
I0L9J4	4HZ6:A	49.89	1086	444	2	438
C6D9L1	1QOX:A	39.01	775	449	5	443
C6DGK6	4B3K:A	48.15	1154	479	3	459
H1X6D4	1TR1:A	33.48	607	447	5	441
A0A0F2DGL5	4B3K:A	47.72	1158	479	1	459
A0A150SQJ4	1OD0:A	46.99	1059	468	26	465
A0A0F2E2Y8	4B3K:A	48.81	1175	479	1	459
A0A139Q647	4B3K:A	47.06	1045	479	37	459
A0A150TFS4	3TA9:A	51.86	1237	458	8	456
A0A150PZ21	1OD0:A	44.57	978	468	33	465
I0L805	1GNX:A	66.31	1529	479	15	476
A0A150NKB0	4B3K:A	47.94	1163	479	1	459
A0A150PSM8	4PTV:A	51.86	1240	452	2	450
M2V479	1NP2:A	51.34	1015	436	6	427
A0A150SAJ0	3TA9:A	51.75	1225	458	8	456
I0LCR6	1GNX:A	57.48	1286	479	16	475
A0A150SS85	1OD0:A	45.25	988	468	33	465
A0A150PV36	1OD0:A	46.10	1040	468	26	465
A0A0F2CZY0	4B3K:A	49.02	1178	479	1	459
I0L5G8	3WH5:A	54.18	1191	457	4	444
A0A0F2E7V7	5FOO:A	48.48	1168	480	1	460
A0A150QY19	3TA9:A	51.42	1218	458	8	456
I0L7W2	3CMJ:A	49.89	1168	465	25	463
A0A0F2E069	5FOO:A	47.84	1163	480	1	460
A0A139R9R9	4B3K:A	47.94	1165	479	1	459
A0A081QCW9	4B3K:A	47.73	1153	479	1	459
A0A0F2D4E2	4B3K:A	48.37	1161	479	1	459
A0A150Q700	1OD0:A	46.77	1052	468	26	465
A0A150QHI3	1OD0:A	44.57	984	468	33	465
A0A0F3HNR9	4B3K:A	48.16	1160	479	1	459
U3UHB2	5DT5:A	40.68	91	471	418	470
A0A0A8GHZ3	1QOX:A	47.96	1061	449	5	444
I0KZ53	1GNX:A	59.74	1324	479	16	472
A0A081KEN4	1OD0:A	48.20	1068	468	29	465
A0A150RSE8	3TA9:A	51.42	1225	458	8	456
A6LTH5	3AHX:A	44.68	1022	453	4	446
A2RK29	3AHX:A	34.10	599	453	4	443
D1C7U8	1OD0:A	42.89	918	468	26	466
W4R1E9	1BGG:A	60.99	1597	448	1	445
A2RLY4	1UYQ:A	30.97	575	447	4	441
A0A0Q6A8N8	1OD0:A	45.87	1024	468	30	463
A0A093BBS0	1GNX:A	49.89	1052	479	17	471
A6M001	4B3K:A	45.85	1124	479	3	459
B8I5U2	1OD0:A	42.76	936	468	28	465
A0A0M1TAG1	3TA9:A	47.32	1067	458	13	450
E3H2Z7	1UYQ:A	32.90	586	447	5	444
C8S0J3	1OD0:A	44.65	1005	468	27	464
A0A0R2PV35	3TA9:A	45.83	1047	458	13	456
A2RJU6	1BGA:A	43.03	347	447	5	165
B2J7D8	1VFF:A	38.95	285	423	1	169
A2RJZ1	4B3K:A	51.21	618	479	256	461
C8SYI3	1UYQ:A	36.03	661	447	5	440
A0A0Q0GK92	3TA9:A	44.24	1020	458	12	452
A0A0P8RGQ5	4PTV:A	46.31	1050	452	7	444
C8TAN5	3TA9:A	47.10	1062	458	13	450

A0XZR8	2O9P:A	43.45	992	454	18	451
R9GWA4	1OD0:A	42.34	959	468	24	462
A0A093B2U2	3WH5:A	52.26	1168	457	6	446
W4QZ14	1BGG:A	62.50	1614	448	1	447
A0A0Q5ZL74	1NP2:A	47.07	905	436	8	432
A0A0A0BA14	1GNX:A	59.53	1404	479	17	478
A0A093B6E8	3WH5:A	46.85	1000	457	1	440
A0XZU6	4PTV:A	42.14	959	452	6	444
K1UFA6	1GNX:A	76.20	1891	479	1	477
A2RJU7	1OD0:A	40.16	218	468	339	465
A0A093AY50	3W53:A	55.00	1254	506	45	502
A6M245	4B3K:A	58.82	1519	479	1	459
A0A093B609	1NP2:A	53.03	1073	436	8	428
K1UXI9	1GNX:A	68.97	1638	479	18	479
A0A0Q4DSU9	3W53:A	53.80	1261	506	39	500
A2RHQ9	1QOX:A	41.40	878	449	1	442
A0A0F0HR66	3W53:A	47.03	1017	506	39	503
A0A0R2RRM8	1OD0:A	43.43	971	468	26	465
A0A089J7H3	1BGG:A	60.22	1532	448	1	444
L8TQ75	3W53:A	46.91	972	506	46	506
A0A0N0JP47	4PTV:A	49.10	1144	452	7	445
A0A0Q9KWX0	1GNX:A	54.80	1185	479	17	470
V4PU71	4PTV:A	49.66	1141	452	7	446
A0A0D7EGA0	3TA9:A	45.76	1052	458	13	450
D9TIB4	3AHX:A	53.20	1249	453	4	444
A0A038CWS1	3TA9:A	46.21	1053	458	13	450
E6XES3	1OD0:A	41.95	937	468	26	462
A0A0B5IUZ3	4PTV:A	46.24	1065	452	7	448
U1L837	3W53:A	55.75	1300	506	46	499
A0A0Q5I7L5	3TA9:A	47.49	1071	458	13	450
U2EIJ2	1OD0:A	46.85	1093	468	17	465
A0A0F7FXF9	1GNX:A	65.01	1550	479	18	476
W1RVK6	2O9R:A	46.09	1066	452	1	447
A0A0F7G0F6	1GNX:A	54.13	1129	479	18	467
A0A0N1L7J0	3WH5:A	52.93	1132	457	6	445
A0A089J6U8	1BGG:A	59.51	1520	448	1	446
A0A0N0J8C4	4PTV:A	49.09	1122	452	7	446
A0A0N1AU11	3TA9:A	49.31	1093	458	17	450
F4QIE2	3TA9:A	50.45	1147	458	13	450
A0A0N0TS10	1GNX:A	50.22	1000	479	17	474
D3HB41	4B3K:A	48.60	1169	479	1	459
F8K1W6	1GNX:A	71.40	1676	479	16	479
F8JXM9	1NP2:A	52.61	989	436	6	427
F2AFP4	1QOX:A	48.19	1071	449	5	444
F4QNW2	1VFF:A	30.28	523	423	4	408
A0A0F5L4U3	1OD0:A	47.73	1053	468	26	464
A0A0N0AL52	1GNX:A	54.17	1127	479	18	472
A0A0T1QDX5	1GNX:A	55.24	1174	479	17	472
E8M8E2	2O9P:A	45.37	1058	454	18	449
A0A0R2U6R1	3TA9:A	45.83	1047	458	13	456
F4QTA0	1VFF:A	33.10	565	423	4	408
A0A087E2J5	1GNX:A	50.53	1138	479	16	479
A8LUQ2	1GNX:A	59.74	1347	479	16	472
A0A0N0TPC5	1GNX:A	55.11	1232	479	1	477
A0A099CYQ1	3TA9:A	50.23	1133	458	13	450
A0A0R2QCF3	3TA9:A	46.48	953	458	75	453
A8LZF3	1GNX:A	56.30	1281	479	16	475

A0A0T1Q6H1	1GNX:A	73.71	1761	479	14	476
A0A0F5L2M2	1OD0:A	44.63	993	468	28	453
V4PUN6	3TA9:A	50.23	1143	458	13	450
A0A0T1QEB7	3WH5:A	53.90	1203	457	6	448
A0A0V8HGU8	5DT5:A	79.91	1978	471	24	466
J9Z024	3TA9:A	45.18	957	458	13	436
F4QNW3	1VFF:A	32.57	563	423	3	408
I0K7N3	1OD0:A	40.68	887	468	28	461
I2UCB9	1QOX:A	34.68	608	449	5	449
A0A0R2PFJ7	1OD0:A	47.39	1067	468	25	465
A0A0D5LPH5	1QOX:A	47.17	1025	449	5	441
A0A094MEV8	3W53:A	48.36	1064	506	46	499
K9XKL8	3AHX:A	41.91	881	453	10	440
T0K8N3	1OD0:A	45.15	1018	468	18	466
A0A0D5LVG4	1GNX:A	44.37	953	479	15	476
A0A094LWT0	1NP2:A	52.57	1056	436	6	428
A0A108U516	1OD0:A	51.68	1170	468	26	465
F5X2P1	5FOO:A	53.70	1435	480	1	471
A0A0B0I1D9	1BGG:A	66.89	1637	448	1	446
A0A066Y8A5	1GNX:A	62.79	1470	479	2	477
E9SVV9	3TA9:A	45.27	1019	458	13	450
A0A094MUC7	3W53:A	54.35	1243	506	45	502
F5X4E2	4B3K:A	65.50	1709	479	2	459
Q1JFR5	4B3K:A	98.75	2549	479	1	479
A0A066XYV3	1NP2:A	52.13	941	436	8	430
Q2GAY4	1VFF:A	31.84	497	423	5	408
A0A0N9ZMP8	1OD0:A	44.19	980	468	28	461
Q2GA89	1VFF:A	31.92	516	423	4	408
A0A0P7FFY7	3W53:A	65.39	1655	506	35	503
Q1JGD8	4B3K:A	45.10	1061	479	3	459
A0A094PKY4	3WH5:A	52.49	1178	457	6	446
H4FDK8	1OD0:A	45.80	1044	468	25	461
K9XAN6	4PTV:A	49.00	1163	452	7	450
A0A094MYW7	3TA9:A	44.27	992	458	8	450
A0A0M2Y0R3	1OD0:A	43.21	969	468	28	462
A0A0R3N3E9	3TA9:A	44.67	947	458	13	455
A0A0Q5UWJ7	1OD0:A	44.82	971	468	27	466
A0A066Y3W8	1GNX:A	68.09	1640	479	12	477
U7DA26	1OD0:A	44.79	976	468	27	465
A0A0B2JR07	3TA9:A	42.92	986	458	12	452
A0A0Q8IF10	1OD0:A	47.05	1045	468	26	464
A0A087C406	1GNX:A	54.35	1185	479	16	467
A0A147J2G4	1OD0:A	45.05	1009	468	17	466
A0A0T2KUG9	3CMJ:A	43.97	974	465	19	460
A0A0B2JMG4	5DT5:A	42.41	964	471	15	462
A0A063KM13	3TA9:A	43.15	995	458	12	452
S2VME4	1GNX:A	56.84	1352	479	16	477
A0A147JDD4	1OD0:A	44.62	1009	468	17	466
A0A063KQP8	5DT5:A	42.41	963	471	15	462
A0A0T7H874	1OD0:A	45.33	1050	468	28	462
A0A0T2L2H1	3W53:A	54.70	1285	506	45	500
A0A0Q7HSM6	1OD0:A	46.36	1035	468	26	464
A0A147HV64	1OD0:A	44.62	1005	468	17	466
A0A069EZ07	5DT5:A	82.81	2048	471	24	465
A0A0C1FML2	1OD0:A	40.86	840	468	28	462
A0A0J1BRZ8	1OD0:A	43.37	1002	468	24	464
M4MZB5	1QOX:A	47.51	1041	449	5	444

A0A084TBH7	1OD0:A	45.98	1019	468	28	465
A0A069EX53	3AHX:A	41.13	981	453	4	440
A0A132BZI3	1OD0:A	44.76	991	468	26	453
A0A0W1DQ54	1OD0:A	48.53	1108	468	28	465
L0DCM3	1E4I:A	42.95	923	447	3	441
A0A0C5VPU5	4HZ6:A	49.06	1074	444	8	431
K5TC37	4PTV:A	45.68	1038	452	2	444
A6EHL7	1OD0:A	39.91	863	468	28	463
A0A109CSG1	1QOX:A	47.29	1051	449	5	444
A0A0C5VV92	1OD0:A	43.49	1029	468	24	464
A0A101I5U2	1OD0:A	50.56	1179	468	24	462
A0A109CYL7	4PTV:A	43.05	963	452	7	444
A0A0M9B721	3WH5:A	45.12	908	457	6	441
A0A0F0L861	1VFF:A	34.30	609	423	3	404
A0A0U4GRN2	1GNX:A	56.69	1325	479	2	478
C2EMN9	3CMJ:A	30.59	575	465	24	464
R5JYX8	4GXP:A	44.42	950	467	13	464
A0A0A3XQP1	3WH5:A	45.02	915	457	4	441
A0A099LU17	2O9P:A	45.62	1068	454	16	449
A0A0F0L7Y0	1GNX:A	51.48	1137	479	13	478
A0A098Y9E7	3W53:A	52.38	1231	506	43	503
A0A147E1P0	3W53:A	54.49	1261	506	36	501
A0A0Q6V157	1GNX:A	42.83	893	479	18	476
C2EKY3	4B3K:A	58.35	1477	479	1	459
A0A0B2ANF2	1NP2:A	49.45	913	436	6	430
A0A011A404	3AHX:A	48.55	1158	453	1	444
A0A010Z8Q7	1OD0:A	42.63	938	468	28	464
A0A0F0L936	1VFF:A	34.38	588	423	4	408
A0A0F0LH38	3W53:A	53.83	1245	506	44	501
A0A0A6N2G4	1OD0:A	98.65	2336	468	25	468
A0A0B1ZDX7	1GNX:A	68.11	1641	479	18	477
A0A0F0KY85	3W53:A	55.53	1267	506	44	500
A0A0U4GCW9	1GNX:A	62.63	1414	479	14	473
Q084Z6	3TA9:A	44.87	1015	458	10	450
A0A0F0KI14	4HZ6:A	39.37	390	444	226	440
A0A0F0KV72	1VFF:A	35.75	619	423	3	404
A0A0A3XRN5	3WH5:A	45.45	921	457	6	441
A0A084EKA4	1OD0:A	44.59	981	468	28	466
A0A0J9D3P9	1OD0:A	45.50	997	468	28	466
A0A0A3ALS5	3AHX:A	41.25	925	453	4	440
A0A0F7VKY4	1GNX:A	55.56	1153	479	18	472
A0A136P200	3TA9:A	49.28	1105	458	13	425
A0A139NFW8	1UYQ:A	34.75	623	447	4	441
A0A088F313	1OD0:A	46.33	1049	468	21	462
A0A0D1M1H3	1VFF:A	32.64	498	423	1	400
A0A120FLV9	3WH5:A	45.41	901	457	3	441
A0A0Q9CW60	1GNX:A	59.70	1409	479	18	478
A0A0R0CU61	1OD0:A	51.70	1149	468	28	465
A0A085K4X8	1OD0:A	45.05	983	468	28	466
A0A0F7VM41	3TA9:A	50.77	1185	458	12	452
A0A0F7VZ69	1GNX:A	69.23	1724	479	1	479
A5ZMW4	1OD0:A	44.08	1012	468	21	465
A0A0Q5IZB9	1GNX:A	59.70	1400	479	18	478
A0A0F7W447	1GNX:A	56.99	1163	479	18	470
A0A0Q5J8U7	3TA9:A	46.36	1012	458	11	455
A0A0F7VZ66	1GNX:A	68.53	1656	479	15	477
R7I469	1OD0:A	45.56	1046	468	27	465

F8A202	1GNX:A	61.99	1451	479	18	477
A0A139NE14	5FOO:A	46.24	1110	480	1	460
A0A0D1M074	1VFF:A	32.87	506	423	1	400
A0A0F7VKY3	3WH5:A	53.03	1201	457	6	449
W1SK95	1BGG:A	58.30	1492	448	1	445
A0A0Q9CPY4	4HZ6:A	47.10	1010	444	5	442
A0A0W8JD77	2O9R:A	44.68	1062	452	16	447
A0A0Q8PY62	3CMJ:A	46.38	1021	465	25	461
F8A798	3CMJ:A	45.43	987	465	25	463
A6V4K6	1GNX:A	40.44	743	479	18	472
A0A0Q8PYD2	3TA9:A	41.35	885	458	13	450
G2GDH5	1GNX:A	86.58	2129	479	1	477
E8URA9	5DT5:A	55.94	1393	471	25	460
A0A0J6CHW8	5DT5:A	79.91	2015	471	24	471
G2G8K4	1GNX:A	67.32	1602	479	17	477
A0A076HY78	1OD0:A	43.18	945	468	28	465
E8UQS3	5DT5:A	55.43	1403	471	24	463
C8XJ34	1GNX:A	63.28	1482	479	17	477
Q9KBK3	1QOX:A	62.36	1574	449	1	449
W2U3C5	1NP2:A	98.37	2200	436	1	430
A0A075R1I1	1E4I:A	39.18	928	447	4	439
A0A0M9CQ95	3WH5:A	55.68	1205	457	6	444
N1JSG8	1VFF:A	39.26	707	423	3	396
A0A0J6FY31	1QOX:A	58.88	1455	449	5	449
A0A089LV95	1TR1:A	59.28	1505	447	5	445
A0A0N0CH46	1GNX:A	66.52	1570	479	16	477
K8E1P0	3AHX:A	33.33	582	453	6	440
A0A0N0CIB1	1QOX:A	47.10	1087	449	5	443
D7A385	3TA9:A	45.45	1059	458	13	451
N1JQ25	3TA9:A	53.38	1228	458	10	452
A0A0M8UQ50	3WH5:A	54.77	1185	457	6	444
A0A0D1EMG8	3CMJ:A	43.65	896	465	21	450
Q0SHX5	3TA9:A	29.69	415	458	13	453
G2GLZ6	1NP2:A	51.47	934	436	8	426
G2GLL5	3WH5:A	55.98	1231	457	6	447
A0A0N1IYI6	1GNX:A	71.98	1717	479	14	476
A0A0M9CPC8	3ZJK:A	53.24	1053	431	7	426
A0A0M9ZAW8	1GNX:A	65.25	1515	479	9	476
