# Supplementary material for: Glutantβase: a database for improving the rational design of glucose-tolerant β-glucosidases
Source: BMC Mol Cell Biol. 2020 Jul 1;21:50. doi: 10.1186/s12860-020-00293-y (PMC7329481; doi:10.1186/s12860-020-00293-y)
Supplement: Supplementary file 2 — Additional file 2: Table S2. The DOPE score for each model selected. [file 12860_2020_293_MOESM2_ESM.pdf]

Table S2. The DOPE score for each model selected.

For each structure, we constructed 100 models. The best model was selected based on the DOPE score (lower values are best). We also constructed Ramachandran plots for each model selected (available at http://bioinfo.dcc.ufmg.br/glutantbase/index.php/home/download).

UniProt ID	Model selected	DOPE score
A3BMZ5	8	2655.425
A3C053	18	6362.4331
B3H5Q1	58	3616.4424
B7F7K7	75	2871.0156
B7F8N7	10	2658.4309
B8AVF0	63	2495.0457
B9FHH2	73	3053.041
B9K7M5	66	2163.438
D5MTF8	58	3006.3699
O48779	4	3154.0103
O64879	21	2674.2595
O64882	9	2631.813
O64883	58	2920.5146
O65458	30	4434.3618
O80689	75	2803.762
O80690	59	2679.5713
P0C946	45	2098.9036
P10482	10	2333.947
P12614	12	2379.8975
P22073	63	2168.9048
P22505	95	2260.5066
P26204	3	2866.5449
P26205	57	2250.7615
P26208	95	2362.6294
P37702	94	3522.1802
P38645	3	4955.9355
P49235	11	2948.4392
P94248	36	3618.9153
P97265	45	2310.5354
Q03506	13	1985.4253
Q08638	1	2212.4216
Q0DA21	2	2312.4216
Q0DIT2	11	3002.1899
Q0J0G2	57	2968.166
Q0J0N4	2	2725.4453
Q1XH04	61	3024.189
Q1XH05	23	3041.3918
Q1XIR9	47	3063.7942
Q25BW4	56	2818.2173
Q25BW5	77	2178.8625
Q2QSR8	97	2801.1116
Q339X2	29	2419.8491
Q3ECS3	78	4316.9551
Q3ECW8	98	2605.2808

Q41761	57	2954.0562
Q4V3B3	52	3193.7681
Q53NF0	74	8842.6777
Q59437	38	2949.8555
Q5JK35	56	4359.9702
Q5N863	18	2421.5591
Q5QMT0	89	2506.3003
Q5RF65	17	2275.4353
Q5Z9Z0	84	2556.4578
Q60DX8	24	2837.9316
Q60DY1	66	2751.4182
Q67XN2	12	3071.3784
Q682B4	1	3016.6482
Q6L597	95	8774.6699
Q75I93	96	2415.7561
Q75I94	45	2677.8999
Q7EXZ4	7	2880.9048
Q7F9K4	53	2793.8032
Q7XKV2	11	2596.4497
Q7XKV4	86	2526.228
Q7XKV5	19	2733.7671
Q7XPY5	91	3178.905
Q7XSK0	56	2597.3027
Q7XSK1	32	1503.8289
Q7XSK2	62	2546.3025
Q84WV2	42	3062.0791
Q84YK7	18	2718.5356
Q8GRX1	86	4529.8462
Q8GVD0	94	2960.7493
Q8GXT2	12	3148.5762
Q8L7J2	73	2420.8108
Q8RXN9	53	3203.1064
Q8RZL1	93	4224.4175
Q93ZI4	58	3147.4402
Q9C525	85	2839.7109
Q9C8Y9	10	2969.106
Q9FH03	84	2685.616
Q9FIU7	93	2567.1611
Q9FIW4	71	2498.2009
Q9FLU8	46	2868.3157
Q9FLU9	29	2939.2844
Q9FMD8	37	1712.8651
Q9FYS3	43	3022.5012
Q9FZE0	6	2403.1724
Q9H227	60	2299.2747
Q9LIF9	27	2831.1072
Q9LKR7	14	2904.0576
Q9LU02	57	2690.696
Q9LV33	68	2576.0356
Q9LV34	76	2673.5105

Q9LZJ1	56	2992.2244
Q9M1C9	7	3179.0718
Q9M1D0	74	2718.4314
Q9M1D1	20	2919.3989
Q9SLA0	73	2900.884
Q9SR37	92	2751.002
Q9STP4	44	3280.9331
Q9SVS1	91	3320.6323
Q9ZUI3	13	3938.603
A0A010NF87	6	2611.0261
A0A010PLT1	6	2582.9121
A0A010QHQ5	89	2556.9211
A0A010Z8Q7	35	2780.3193
A0A010ZQC5	50	2263.6855
A0A011A404	36	2498.9272
A0A011AHF0	2	2051.0544
A0A011AM20	67	4182.3066
A0A011SVF9	21	2473.2344
A0A011T636	72	2731.0854
A0A014LC96	74	2211.0042
A0A014MMT7	74	2260.4409
A0A016QQV3	31	2653.113
A0A017H8P6	9	3074.7234
A0A017HBK6	25	4193.9985
A0A021X5U5	14	2897.9094
A0A022L2R1	60	2583.1042
A0A022LMS0	94	2749.8569
A0A022M6R2	74	2280.2107
A0A022MI49	48	2880.29
A0A022MK77	39	2234.3254
A0A023NXL5	55	2588.1912
A0A023X547	8	3195.2109
A0A023XM13	91	2584.4609
A0A023XS67	63	2490.8611
A0A023ZT20	11	1897.1439
A0A023ZTM1	23	1802.2599
A0A023ZUM4	29	1421.6946
A0A024GYL3	52	2916.2422
A0A024GZT8	70	2645.9072
A0A024H7A5	34	2294.0093
A0A024J0W6	4	2281.5801
A0A024KFD3	72	2619.863
A0A024YMJ6	40	4822.7769
A0A031FTF5	3	2811.9397
A0A031GUM1	71	2437.3315
A0A031HLF2	12	2799.2063
A0A031IDU7	18	2035.13
A0A031JM39	59	2502.4155
A0A034T2M4	27	2637.7178
A0A037ZIH9	2	2482.603

A0A038CWS1	32	2545.4304
A0A058ZJC5	48	2470.5835
A0A059D1K5	10	2379.252
A0A059F8D5	47	4251.7983
A0A059GAD1	8	2127.9172
A0A059U805	38	1775.5981
A0A059VW33	31	2695.374
A0A059WHP2	48	2566.718
A0A060DJG7	87	2784.4741
A0A060DMA5	90	2842.6331
A0A060DQK7	38	2655.0669
A0A060DVX9	92	2315.0491
A0A060I1G7	75	2515.1208
A0A060NR41	67	1568.8278
A0A060NTE4	14	1393.0901
A0A060NUN8	21	1384.3101
A0A060NUP4	63	1507.8369
A0A060NZS1	43	1401.0066
A0A060NZS6	50	1543.3534
A0A060P1M7	27	1626.3906
A0A060P1N1	54	1437.658
A0A060V7Z8	24	2588.7117
A0A060VPE0	16	2677.7793
A0A060ZE79	72	2285.4517
A0A060ZTS4	100	2289.2563
A0A061EVC3	32	2311.1956
A0A061EYK8	26	2396.1396
A0A061MV28	32	2416.3423
A0A061QAE7	92	2419.9912
A0A062URN0	58	2163.95
A0A062VPY9	95	2751.157
A0A062VVQ8	9	3108.1494
A0A062Y195	30	2927.7114
A0A063KM13	57	2523.718
A0A063KQP8	57	2810.1797
A0A064BWP9	12	2845.7471
A0A064C5A1	3	2973.0305
A0A066TTF6	79	2596.6279
A0A066TWD6	98	2444.0315
A0A066TXP2	61	2658.5227
A0A066U508	83	2821.3076
A0A066U9N2	52	2497.1064
A0A066UD62	64	2070.2244
A0A066XYV3	70	2945.2698
A0A066Y3W8	90	4096.8589
A0A066Y8A5	19	2568.9023
A0A066Z0U4	23	2319.7676
A0A067GNY9	93	2973.425
A0A067LG20	51	2657.3689
A0A068HD71	31	2630.1008

A0A068NKR9	99	2584.1348
A0A068SUC0	59	3116.8579
A0A068TBI5	1	2819.3235
A0A069CLU2	44	2577.6189
A0A069DJR7	12	2148.0356
A0A069EX53	20	2368.4375
A0A069EZ07	25	2012.386
A0A069JRR8	96	2360.0938
A0A069JV67	7	2166.3093
A0A069JXB6	25	2642.3591
A0A069JY86	62	2438.2542
A0A069K8M0	16	4440.8989
A0A071IES9	51	2396.2583
A0A072CAW3	18	2271.5068
A0A072CG79	62	2399.8308
A0A072S5G8	62	4143.8232
A0A072S7E3	19	2230.0886
A0A072SFZ8	75	3690.1985
A0A072SIA6	84	4026.3018
A0A073CLU2	47	4372.0518
A0A074J7B8	84	2514.6836
A0A074JPD3	37	2854.1812
A0A074JXI5	94	2774.0139
A0A074L9I1	12	2130.042
A0A074LBK9	89	2131.1042
A0A074LMB2	4	2297.4146
A0A074U5W9	27	2502.4741
A0A075HLG3	48	2676.1621
A0A075IXW8	94	2830.2742
A0A075IXX0	53	2437.8542
A0A075IXX4	71	2630.6331
A0A075JVS6	85	2281.9023
A0A075P142	2	2472.5225
A0A075P3W9	62	2462.3718
A0A075R1I1	100	3475.7383
A0A075UHQ3	94	3779.2095
A0A075UKX9	58	2987.9014
A0A075UMV5	47	4955.519
A0A075UTP7	1	2732.1338
A0A076HY78	8	2482.9756
A0A076JFX4	93	4500.6348
A0A076JJU4	86	3618.4639
A0A076JRL8	53	2471.929
A0A076K4F3	67	2616.4851
A0A076LLP4	16	2691.3621
A0A076LVL8	32	2235.5305
A0A076M6B6	91	2916.1836
A0A076MR11	82	2555.134
A0A076VZW1	33	2218.0813
A0A077EM06	37	2612.4976

A0A077M7L6	79	4272.0669
A0A077MDS7	58	2403.5251
A0A077S2F2	20	2667.7344
A0A077XW08	44	2575.5615
A0A078CC24	35	2486.4285
A0A078HIJ6	23	2534.0942
A0A078HP64	57	2431.1863
A0A078MRL3	69	2369.2798
A0A081BLU3	19	2718.9058
A0A081BMQ2	66	2510.5056
A0A081C5A0	69	2619.1736
A0A081CSM4	68	3170.3936
A0A081EBT3	26	4304.7412
A0A081ECE4	15	3558.3071
A0A081EFM7	88	2521.0713
A0A081EFM8	18	4655.0728
A0A081EFN3	59	2458.6238
A0A081EGY5	86	3974.4075
A0A081KEN4	4	2703.0029
A0A081MJ60	50	2353.166
A0A081MJA7	5	2692.9727
A0A081PGZ2	57	2523.8586
A0A081QCW9	30	2798.1892
A0A081XPY9	24	4067.4238
A0A081XU26	81	2347.0925
A0A081XV05	16	3982.041
A0A081XXW6	82	4118.0386
A0A083XQA5	39	4286.6445
A0A084EKA4	32	2694.0205
A0A084G332	15	3008.7678
A0A084GQB3	32	2436.2275
A0A084GQG8	99	2464.0422
A0A084JPX9	11	2444.7556
A0A084JRD9	90	2517.3696
A0A084SEI6	43	2576.479
A0A084TBH7	14	3001.4907
A0A084U7W6	65	2737.3152
A0A084YXZ2	24	2346.4341
A0A085AL28	96	2658.6584
A0A085CIX4	58	2639.2888
A0A085EQI1	42	2529.2051
A0A085FII2	13	2966.3574
A0A085FY80	9	2290.0427
A0A085G494	40	2799.6621
A0A085G5B6	90	2740.8408
A0A085GCD2	32	2743.2036
A0A085GLV6	36	2764.5413
A0A085GPH6	14	2794.6438
A0A085GZG7	15	2855.8694
A0A085H3F7	51	2922.6619

A0A085H4A7	100	2560.4817
A0A085H8M1	33	2599.2129
A0A085HF97	43	2509.8313
A0A085HGX9	51	2733.6892
A0A085HKV0	56	2569.082
A0A085I3Q9	79	2681.7278
A0A085I917	14	2813.7214
A0A085IFG4	75	2566.0522
A0A085K4X8	78	2659.4126
A0A085PF90	92	2584.2793
A0A085TXE9	4	2536.5454
A0A085UBR0	71	2650.894
A0A085VZK2	31	2558.4014
A0A085WL72	100	2701.1265
A0A085WWB9	73	2669.6887
A0A085ZID8	15	2462.645
A0A086BNK9	20	2200.6802
A0A086DXW8	97	2230.272
A0A086E1I4	68	2576.0288
A0A086GLW0	5	4215.334
A0A086GRE0	64	4470.2998
A0A086N2K8	98	4211.2642
A0A086N6Y5	43	3171.1108
A0A086N873	83	2257.8313
A0A086W5P1	94	2315.3174
A0A086WCG9	51	2574.5586
A0A086Z2F7	72	2549.5869
A0A086ZGS5	100	2174.7051
A0A086ZNH9	32	3538.7771
A0A087AD30	97	2484.2595
A0A087AH51	55	3627.1953
A0A087AM90	56	3583.9092
A0A087AN75	45	2327.9106
A0A087AN97	98	3567.5242
A0A087AXE0	95	3551.6199
A0A087AYW0	75	2471.793
A0A087C406	60	3617.469
A0A087C7J4	66	4551.7075
A0A087CIL9	78	2534.6584
A0A087CJA9	61	2698.2668
A0A087CQV8	55	2325.8269
A0A087CV22	93	2419.4109
A0A087DDN9	59	3636.5635
A0A087DF01	63	2263.7314
A0A087DJE3	64	2501.9968
A0A087E2J5	77	4065.6738
A0A087E771	90	4253.856
A0A087EGL8	4	2443.9153
A0A087GTX2	75	2980.3398
A0A087IJD4	81	2482.2817

A0A087K4Q9	21	2989.5144
A0A087K759	48	2116.9614
A0A087K8X1	96	2406.3652
A0A087KCY5	76	3615.0151
A0A087KGB8	23	4335.3516
A0A087LSW7	59	2651.762
A0A087M1I5	73	3076.9607
A0A087VSR2	49	4483.3613
A0A088F313	4	2600.1694
A0A088ME52	6	2395.5947
A0A089GG65	54	2537.6013
A0A089HNV9	46	2133.6936
A0A089J646	84	2189.7039
A0A089J6U8	29	2221.7603
A0A089J7H3	87	2200.3398
A0A089LV95	8	2162.3525
A0A089X8V3	23	2988.6021
A0A089XBM8	61	4337.6646
A0A089XFH1	96	2278.3489
A0A089XFH5	41	2646.7856
A0A089Y711	65	2668.3945
A0A089Z8E9	16	2241.6411
A0A090RRD9	76	2550.2917
A0A090SBY8	8	2904.9697
A0A090SKA4	61	2409.0115
A0A090SN13	95	1938.2991
A0A090SNE8	3	2713.6719
A0A090SW15	39	2505.7634
A0A090SXD7	50	2796.0435
A0A090SZU6	23	2472.1174
A0A090T3G1	30	1035.8912
A0A090T7F7	74	662.9805
A0A090TL25	54	3790.7966
A0A091C594	48	2010.077
A0A091CE87	45	2599.0525
A0A093AY50	35	2819.6777
A0A093B2U2	6	2122.1538
A0A093B609	39	2507.9209
A0A093B6E8	55	2826.4355
A0A093BBS0	76	2516.741
A0A094IRZ0	13	2365.7866
A0A094JYA8	98	2805.5835
A0A094LWT0	18	2926.1963
A0A094MEV8	45	3766.6389
A0A094MUC7	40	2807.9912
A0A094MYW7	85	2972.8281
A0A094PKY4	26	2129.2878
A0A095ALE8	45	2764.7461
A0A095AS65	77	2406.637
A0A095B600	29	2842.6365

A0A095TZ25	34	2467.7307
A0A095XJG5	97	2809.7957
A0A097R6G4	81	2441.4773
A0A098BMM1	55	2929.7168
A0A098EWY7	37	2195.3323
A0A098LEE1	78	2527.3772
A0A098M8A7	94	2236.0637
A0A098QUS4	79	2540.886
A0A098RPC9	33	2569.197
A0A098TDE4	43	2453.7532
A0A098UBW7	56	2536.8335
A0A098UII0	85	1953.4158
A0A098Y9E7	16	2869.4072
A0A098Z269	61	3013.2832
A0A098Z798	69	2796.9785
A0A099CYQ1	27	2760.8318
A0A099D543	97	3051.4231
A0A099DE23	41	1982.2843
A0A099DEC7	32	2361.4004
A0A099IJ12	52	2338.3972
A0A099IN71	43	2746.7583
A0A099JMJ2	98	2623.1824
A0A099KSH6	92	2757.491
A0A099L2C3	46	2979.4172
A0A099LJ91	9	2560.9885
A0A099LU17	58	2538.4778
A0A099XYQ1	10	2717.3599
A0A0A0BA14	11	4430.04
A0A0A0BS35	17	4759.0264
A0A0A0BTI4	51	2527.4885
A0A0A0BZ90	93	2569.4236
A0A0A0C0T3	17	3533.8535
A0A0A0C5P9	7	2501.072
A0A0A0C667	11	2591.8108
A0A0A0C9G0	27	3828.4966
A0A0A0DJG4	48	3020.3901
A0A0A0J8T6	71	2381.0381
A0A0A0J974	35	2354.6909
A0A0A0JPW7	75	2543.3135
A0A0A0K5H9	75	3252.3357
A0A0A0LHE2	9	2468.2009
A0A0A0N9S0	37	2275.7866
A0A0A0NSJ2	33	2262.0691
A0A0A1CVU2	45	2758.8713
A0A0A1DS80	14	2515.1157
A0A0A1E4J6	100	1475.8983
A0A0A1E9M1	90	1811.5743
A0A0A1RS35	61	2506.5012
A0A0A1WBR5	87	3079.2544
A0A0A2HBW8	45	2636.6748

A0A0A2MG18	96	2548.7986
A0A0A2TFJ7	73	2243.6294
A0A0A2TVI0	81	2055.553
A0A0A2TZZ2	76	2183.0356
A0A0A2UWI2	39	2052.0083
A0A0A2WJJ7	23	2407.8186
A0A0A3ALS5	11	2684.1819
A0A0A3F356	94	2627.9336
A0A0A3XQP1	99	2577.7842
A0A0A3XRN5	38	2526.6521
A0A0A5G8H5	84	2213.5427
A0A0A5GAG4	89	2224.4875
A0A0A5GRV1	84	2501.5435
A0A0A5I272	56	2666.9927
A0A0A6DXI2	61	2583.3582
A0A0A6N2G4	77	2124.9185
A0A0A6S8P4	47	2623.3933
A0A0A6UC18	25	4427.7808
A0A0A6UCN6	51	4240.0288
A0A0A6UGB3	53	2782.189
A0A0A6VUV8	15	2238.9072
A0A0A6XEE8	52	4417.0229
A0A0A6ZH67	38	2709.8459
A0A0A7EN85	4	2928.6367
A0A0A7FUT5	77	2429.0269
A0A0A7HJS7	30	1187.2146
A0A0A7I8R1	89	3604.6362
A0A0A7K2D1	42	2597.2178
A0A0A7KKG8	41	2409.5789
A0A0A7PHH8	47	2709.2102
A0A0A7RBQ4	45	2331.5806
A0A0A8GHZ3	25	2463.0879
A0A0A8VIP7	24	2653.9609
A0A0A8X2J6	24	2170.053
A0A0A8X5Z5	69	2051.5054
A0A0B0D6F9	12	2230.6853
A0A0B0HH03	38	866.1043
A0A0B0HK50	70	2128.2434
A0A0B0I1D9	29	2220.6606
A0A0B0S9N8	40	2144.4377
A0A0B0SBM0	94	2078.1487
A0A0B1ZDX7	54	3984.3662
A0A0B2A5B4	34	2458.7332
A0A0B2A6F6	35	2415.8096
A0A0B2ANF2	78	3159.98
A0A0B2AU34	32	2670.124
A0A0B2BNX5	78	2460.3367
A0A0B2FD09	28	2206.1047
A0A0B2JMG4	4	2851.0986
A0A0B2JR07	71	2540.261

A0A0B2P111	70	2709.5618
A0A0B2P194	56	3022.6411
A0A0B2P7I6	82	1659.1562
A0A0B2PA60	11	2683.8096
A0A0B2PAJ2	28	2534.7834
A0A0B2PFA1	41	7978.9517
A0A0B2PM93	53	4255.9849
A0A0B2QCU1	58	2782.6787
A0A0B2QG59	74	2350.4788
A0A0B2QKY1	77	2334.9182
A0A0B2QN99	86	4254.1284
A0A0B2QNY1	30	2661.0232
A0A0B2QPR9	95	2825.7795
A0A0B2QUJ4	62	2765.0298
A0A0B2QVE6	40	2271.8545
A0A0B2QZ86	57	2810.7029
A0A0B2QZT4	11	2699.9612
A0A0B2R063	51	2723.0608
A0A0B2R068	27	2685.9856
A0A0B2R1N8	3	2864.0293
A0A0B2R4L8	40	2671.7578
A0A0B2R4M4	96	2998.2061
A0A0B2R6G1	55	2589.6626
A0A0B2R789	54	2715.0103
A0A0B2R7W9	51	2832.0859
A0A0B2R9G2	40	2370.325
A0A0B2RAL2	68	5267.644
A0A0B2RCK9	99	3743.8169
A0A0B2REW1	62	2665.2241
A0A0B2RFK5	29	4828.1909
A0A0B2RH44	77	2763.3706
A0A0B2RHR0	75	2711.1453
A0A0B2RIG5	86	2477.3936
A0A0B2RL45	36	2260.656
A0A0B2RML7	79	2211.6704
A0A0B2RNP1	11	2695.708
A0A0B2RQU5	47	6127.8271
A0A0B2RR43	7	2734.7402
A0A0B2RUW4	30	6066.1807
A0A0B2RXI2	97	2060.8093
A0A0B2RYB1	56	3145.0823
A0A0B2S5K0	8	2892.0225
A0A0B2S984	35	2750.1509
A0A0B2SCY0	51	2031.9269
A0A0B2SD69	94	2690.0098
A0A0B2SPS7	48	8656.6816
A0A0B2SQQ1	54	2279.4912
A0A0B2SW01	86	3190.7141
A0A0B3BP14	30	2049.5862
A0A0B3SAE8	32	2661.2368

A0A0B3XR14	39	2339.5449
A0A0B3Y5K1	87	2694.7673
A0A0B4DM32	51	3737.4412
A0A0B4IY53	33	2743.7769
A0A0B4X8M0	14	2383.03
A0A0B4Y698	40	2831.6633
A0A0B5ARU7	8	2517.0391
A0A0B5DAL1	97	3097.583
A0A0B5DC15	12	4668.6821
A0A0B5DKC1	26	2384.8591
A0A0B5DL02	71	4233.2529
A0A0B5DTX4	78	2498.229
A0A0B5EHK8	92	2421.231
A0A0B5ELK7	5	2697.085
A0A0B5F5Z8	94	2253.3748
A0A0B5HY92	58	4708.21
A0A0B5I7U1	26	5395.8989
A0A0B5IE61	85	4030.0518
A0A0B5IJ38	97	2317.5461
A0A0B5IUZ3	17	2252.8076
A0A0B5L2L8	33	2209.8955
A0A0B5QJW2	70	2453.1558
A0A0B5SBW4	66	2516.3501
A0A0B7G574	7	2457.0625
A0A0B7KU25	69	2791.7107
A0A0B7L5X3	33	2797.6733
A0A0B7LKH4	78	2828.8623
A0A0B7LW39	4	2830.717
A0A0B8NWC0	6	2666.606
A0A0B8NYV8	51	2710.8884
A0A0B8PJ41	71	2722.3188
A0A0B8PN97	76	2631.2961
A0A0B8PXL0	10	609.8697
A0A0B8QBC2	21	852.1509
A0A0B8QE43	2	2737.0381
A0A0B8QJC8	39	3346.2566
A0A0B8QJZ1	46	1992.5613
A0A0B8QPM7	62	2876.4622
A0A0B8T902	87	2489.0879
A0A0B8XPV5	12	2555.4475
A0A0B9A8W4	82	2578.0354
A0A0B9AI87	60	2616.2058
A0A0C1BUB6	41	2612.6677
A0A0C1FML2	86	2405.021
A0A0C1JFQ6	12	2375.7346
A0A0C1KVY7	34	2347.8513
A0A0C1LYC1	19	2113.3191
A0A0C1R2B8	74	2846.8948
A0A0C1WWF2	43	4570.0913
A0A0C1Y8K3	80	2805.9863

A0A0C1Z6J0	16	2466.2646
A0A0C1Z713	15	2579.4077
A0A0C1ZBY6	13	2533.1038
A0A0C2ALF5	19	2361.4875
A0A0C2AUK0	55	2155.9756
A0A0C2BEE0	57	4063.8054
A0A0C2BIK2	52	2184.6333
A0A0C2JA10	94	2841.739
A0A0C2JEX1	38	4788.3525
A0A0C2KLS1	62	2262.4148
A0A0C2L810	43	2504.8357
A0A0C2LD29	34	2472.1135
A0A0C2PIF5	98	2690.238
A0A0C2PJ36	45	2813.4053
A0A0C2RQE4	56	2623.9111
A0A0C2TJW8	84	2725.5979
A0A0C2TJZ0	73	2568.2354
A0A0C2TLE6	65	2828.3635
A0A0C2UQI7	9	2262.0942
A0A0C2V0V3	35	2402.7717
A0A0C2W2Y2	75	2208.7439
A0A0C2YSN9	36	2658.4631
A0A0C3EDV1	39	2689.9414
A0A0C5BBK0	18	2219.835
A0A0C5FQ81	9	3936.5317
A0A0C5FQ82	14	3941.1177
A0A0C5FSX0	74	3139.2993
A0A0C5FZ69	78	2615.5945
A0A0C5FZ74	42	2250.8701
A0A0C5VPU5	55	2358.5964
A0A0C5VV92	30	2481.9746
A0A0C7KRG8	16	2413.2988
A0A0C7KXK1	32	2333.6094
A0A0C7NKR7	27	2380.0864
A0A0C9NHL2	80	2979.5576
A0A0D0EF31	27	2313.5427
A0A0D0F5F3	11	2661.8784
A0A0D0FQS3	91	2612.9817
A0A0D0G0X7	49	2409.4941
A0A0D0G1V9	95	2158.8555
A0A0D0G9C2	65	2659.9995
A0A0D0GGG1	76	2229.6262
A0A0D0JB19	13	2564.6248
A0A0D0JER1	39	2636.9727
A0A0D0K764	61	3924.7219
A0A0D0K9U3	12	3059.7046
A0A0D0LT36	29	2497.1001
A0A0D0MCX8	65	2407.3394
A0A0D0MJ01	86	2758.5466
A0A0D0N6M1	64	2733.2766

A0A0D0P4K6	47	2330.4041
A0A0D0Q9A4	82	2766.6294
A0A0D0UVT1	55	3583.1912
A0A0D0WQU9	59	4360.1914
A0A0D0WQW4	18	2556.5679
A0A0D0X8K6	49	4912.0635
A0A0D1A4S8	39	2935.0144
A0A0D1A767	54	2576.4897
A0A0D1C9J4	70	2450.0842
A0A0D1EMG8	61	2393.8354
A0A0D1LRQ3	69	2598.8237
A0A0D1M074	61	2562.7012
A0A0D1M1H3	37	2626.0344
A0A0D1MRA2	17	2700.4712
A0A0D2KRE6	58	4375.1538
A0A0D2LRL7	21	3729.7532
A0A0D2LSH8	84	1196.0232
A0A0D2MNY3	94	3143.1294
A0A0D2RJD0	30	2455.084
A0A0D2SVQ2	10	2329.2537
A0A0D2VQR6	9	4138.4692
A0A0D2WNN4	70	2486.0222
A0A0D3BZC1	79	2534.8003
A0A0D3EXD4	18	2421.5591
A0A0D3LHF6	49	2477.8206
A0A0D3RLY6	65	2802.728
A0A0D4C3S4	56	2550.7397
A0A0D4DUA9	83	2826.6218
A0A0D5BTJ1	42	2662.6709
A0A0D5CLN7	84	2640.6501
A0A0D5LPH5	37	2739.4585
A0A0D5LVG4	66	4051.4907
A0A0D5VJL0	81	2526.9934
A0A0D6B1Z9	63	2533.2493
A0A0D6GCL2	71	2734.4502
A0A0D6IWA2	83	4721.6685
A0A0D6J947	90	3014.8535
A0A0D6R2S3	15	2504.4517
A0A0D6SDX6	27	2578.8408
A0A0D6SQK1	10	2572.054
A0A0D6T6K2	32	3033.3557
A0A0D6TF18	15	2769.2507
A0A0D6UP56	22	3632.7393
A0A0D6V8N6	51	2386.5854
A0A0D6VAY7	9	3066.4617
A0A0D6VJ04	15	2174.9341
A0A0D6VK56	29	2507.3477
A0A0D7CQQ7	87	3610.1077
A0A0D7D1B2	59	2476.9119
A0A0D7D5G2	18	3654.3342

A0A0D7DCY0	44	2339.0542
A0A0D7DDZ0	62	2122.8369
A0A0D7EGA0	50	2564.7444
A0A0D7N7J6	4	2565.9941
A0A0D7PHQ3	23	2488.7629
A0A0D7Q7W0	64	2483.7712
A0A0D7QRU5	13	4167.9229
A0A0D7X402	84	2206.0173
A0A0D7X839	94	2136.9575
A0A0D8CBC8	66	2781.2322
A0A0D8FUW7	73	2606.6658
A0A0D8HFJ3	68	4101.7632
A0A0D8HP97	30	4527.8032
A0A0D8HXD7	32	2470.7896
A0A0D8IVH6	36	2623.7554
A0A0D8KD33	13	2408.5771
A0A0D8KR32	97	2556.323
A0A0D9V904	98	2469.1294
A0A0D9YIM6	24	2678.3171
A0A0E0FX18	18	2421.5591
A0A0E0JS71	53	2402.176
A0A0E0N689	43	2632.4324
A0A0E0UG31	30	2375.9395
A0A0E0X0A5	88	2548.6931
A0A0E0YIZ3	5	2661.3906
A0A0E0YLJ1	35	2746.6846
A0A0E0Z721	37	2387.1816
A0A0E1C991	35	2932.1414
A0A0E1CAA6	33	2591.6797
A0A0E1CHW4	19	2661.9041
A0A0E1XFT7	98	2990.7239
A0A0E2PCP3	45	2734.6726
A0A0E3GPF8	73	2331.1926
A0A0E3UIK8	14	2459.3103
A0A0E3URF5	25	3342.0911
A0A0E3V7Q7	28	2685.1101
A0A0E3VUW2	72	2393.8833
A0A0E3ZBU6	2	2446.783
A0A0E4A6R5	39	2562.8433
A0A0E4FYJ7	96	2488.1228
A0A0E7TKP3	64	3011.1643
A0A0E8P693	53	2809.334
A0A0E8TAC1	21	2913.0847
A0A0E8ZC59	77	2967.0886
A0A0E9N138	93	2842.0247
A0A0F0EY28	13	2535.7751
A0A0F0FYZ1	88	2133.4314
A0A0F0GB25	32	2760.0564
A0A0F0GS07	13	3904.3381
A0A0F0H025	1	2940.9434

A0A0F0H1R8	75	3705.1343
A0A0F0HB62	77	4847.1763
A0A0F0HCP0	50	2109.7917
A0A0F0HR66	22	4189.0234
A0A0F0K8M2	97	2954.426
A0A0F0KH49	8	2545.23
A0A0F0KI14	81	1252.6069
A0A0F0KTN0	93	2080.9153
A0A0F0KV72	16	3738.4675
A0A0F0KW36	53	3021.4697
A0A0F0KY85	67	2741.8867
A0A0F0L6Y0	27	2735.0239
A0A0F0L7Y0	4	2729.0969
A0A0F0L861	36	2785.6055
A0A0F0L936	18	2294.657
A0A0F0LCC5	58	2788.0483
A0A0F0LH38	66	2476.9209
A0A0F0LIL6	17	4009.4351
A0A0F0LL99	3	2867.0342
A0A0F0LQJ8	67	4329.2495
A0A0F0LTP0	94	2619.8643
A0A0F0LXG2	5	2815.4387
A0A0F0M3K8	46	3887.0139
A0A0F2C518	73	4940.7163
A0A0F2C8E2	42	2546.3555
A0A0F2C9B3	89	3162.4602
A0A0F2CCH8	42	4166.0962
A0A0F2CIZ5	60	2940.8711
A0A0F2CN10	73	2687.2073
A0A0F2CZY0	21	2775.8352
A0A0F2D4E2	30	2760.8245
A0A0F2D7W6	31	2721.5105
A0A0F2DGL5	17	2762.8701
A0A0F2E069	88	2631.4939
A0A0F2E2Y8	83	2776.3452
A0A0F2E7V7	42	2600.3123
A0A0F2GBE9	49	2802.0139
A0A0F2IVI1	49	2547.5793
A0A0F2KL03	93	2562.0793
A0A0F2KN96	3	2497.5742
A0A0F2KQJ2	96	4082.1606
A0A0F2KSV2	98	2621.886
A0A0F2SQ31	83	2473.4849
A0A0F2TGG7	8	2318.0618
A0A0F3FR95	66	2404.9624
A0A0F3H2P5	19	2970.2495
A0A0F3HNR9	60	2807.2546
A0A0F3HTY9	3	2784.9912
A0A0F3I349	76	2957.4951
A0A0F4FKU4	23	2462.4707

A0A0F4I4Z2	53	2174.5044
A0A0F4IEP9	66	4779.1445
A0A0F4IV83	9	2277.0046
A0A0F4J890	61	3962.0188
A0A0F4JPP7	24	2387.2837
A0A0F4JRE9	38	2335.6228
A0A0F4KAY0	86	4298.9028
A0A0F4KFS6	41	2139.7507
A0A0F4KGD7	19	4307.8022
A0A0F4KH84	6	2353.4131
A0A0F4L1B4	95	4018.4062
A0A0F4NPY6	56	2485.3716
A0A0F4NX57	7	2100.4414
A0A0F4NXS1	7	2104.7678
A0A0F4PXY4	20	2245.8162
A0A0F4QMM0	53	2645.4141
A0A0F4RJA0	50	2643.3198
A0A0F4RQE6	66	2399.2354
A0A0F4RW23	57	2523.718
A0A0F4SDI3	57	2810.1797
A0A0F4VWL7	28	2446.8457
A0A0F4VX87	82	2435.0806
A0A0F4W6W8	82	2435.0806
A0A0F4WR38	28	2446.8457
A0A0F5ABA1	38	2478.8972
A0A0F5AH02	23	4209.5137
A0A0F5FIF7	55	3340.6099
A0A0F5FJM3	10	2967.998
A0A0F5L2M2	39	2855.198
A0A0F5L4U3	27	3023.2134
A0A0F5LUQ4	22	3326.2832
A0A0F5PXV3	99	2993.7761
A0A0F5Q355	8	3429.5405
A0A0F5R775	88	2067.3831
A0A0F5VQF2	65	4797.8945
A0A0F5VT61	57	2256.2573
A0A0F5W8Q8	57	2286.0383
A0A0F5YLR6	23	3069.2642
A0A0F6ALA9	56	2169.1658
A0A0F6F044	25	2227.6572
A0A0F7BQ11	19	2594.8481
A0A0F7C1J0	22	3490.6694
A0A0F7CHR9	21	2171.5732
A0A0F7FXF9	44	2348.0049
A0A0F7G0F6	47	3278.1814
A0A0F7JM33	42	2399.8875
A0A0F7JQC2	56	3100.2244
A0A0F7KKB7	52	2668.7283
A0A0F7N5B2	9	4613.5903
A0A0F7NE55	52	2234.762

A0A0F7P351	71	2599.6838
A0A0F7PCX2	16	2380.6277
A0A0F7VKY3	41	2254.0981
A0A0F7VKY4	24	2701.7832
A0A0F7VM41	6	2692.8
A0A0F7VZ66	7	3913.0066
A0A0F7VZ69	3	3972.3293
A0A0F7W447	12	3157.1296
A0A0F8CX61	91	7920.6431
A0A0G0GD78	36	4134.3477
A0A0G0JIG9	29	4026.2673
A0A0G0SN72	97	3898.1379
A0A0G1NE73	51	4721.3613
A0A0G2R2A3	84	2244.3506
A0A0G2ZHK7	89	2665.4797
A0A0G3AB23	20	2264.2798
A0A0G3AGN5	66	4127.4658
A0A0G3AH06	5	2846.4236
A0A0G3BK76	74	2270.7886
A0A0G3BNS0	58	2879.615
A0A0G3R1D6	76	2635.9773
A0A0G3S6W4	53	2539.1914
A0A0G3V5H1	87	2191.3818
A0A0G3V6A1	15	3437.2593
A0A0G3X6P8	18	2700.7219
A0A0G4AZ53	98	2458.2344
A0A0G8CCQ6	49	2503.3359
A0A0G8EM50	52	2490.7434
A0A0G8EP62	2	2709.1682
A0A0G9HBF3	36	2359.7407
A0A0G9L7U4	1	2439.0559
A0A0H0XXR0	54	2524.3386
A0A0H0ZZM6	66	2251.4048
A0A0H1AV25	68	2532.9434
A0A0H1AXC9	91	3888.4956
A0A0H1AY18	99	2271.0588
A0A0H2KNB6	83	4041.3184
A0A0H2NBH8	35	2469.3264
A0A0H2ZM73	93	2877.821
A0A0H3BZ57	2	2449.0754
A0A0H3C0Y6	70	2778.645
A0A0H3C9V1	14	4422.6577
A0A0H3CV54	41	2924.4722
A0A0H3D0W0	74	4081.481
A0A0H3D8I7	90	2761.1492
A0A0H3DGN3	19	2670.8999
A0A0H3DI81	54	2484.6338
A0A0H3FW13	38	2506.3113
A0A0H3GTM8	29	2536.3438
A0A0H3HA15	31	2630.1008

A0A0H3NSP1	42	2671.887
A0A0H3NTL2	32	2958.8049
A0A0H3NVV8	11	2599.0142
A0A0H3V5F1	75	2628.8096
A0A0H3V883	31	2200.782
A0A0H3V8A5	33	2713.6636
A0A0H4C1P7	47	2185.5049
A0A0H4C4H9	15	4207.1567
A0A0H4CJ37	99	3382.9661
A0A0H4CKJ0	35	2753.4241
A0A0H4KYB8	7	2170.748
A0A0H4NXH8	78	2062.9062
A0A0H4RRS8	4	2367.1753
A0A0H4WPC8	6	2588.7935
A0A0H5B9M3	54	2401.8394
A0A0H5CJN4	77	4083.9663
A0A0H5D0V7	30	2471.7043
A0A0H5D2M0	5	2579.7227
A0A0H5MA25	70	2784.7119
A0A0H5NKN5	44	3746.0234
A0A0H5SI25	79	2723.0425
A0A0I5H012	74	2970.6975
A0A0I9FSI8	9	2608.4553
A0A0I9GY50	95	2993.2463
A0A0I9LTB1	89	2632.406
A0A0I9P479	69	2588.6299
A0A0I9P996	84	2588.4724
A0A0I9PUS2	82	2371.231
A0A0J0UV64	44	2835.8442
A0A0J0UXC6	33	2978.9685
A0A0J0Y754	48	2536.873
A0A0J1BRZ8	1	2374.165
A0A0J1DC04	38	2376.6284
A0A0J1EVE4	38	2547.3396
A0A0J1FMP4	18	2682.9431
A0A0J1G016	98	2638.7236
A0A0J1GLR9	58	2465.7505
A0A0J1IZ59	31	2713.949
A0A0J2GV46	57	2528.7561
A0A0J4RM61	15	2624.3865
A0A0J5AB40	38	2596.3564
A0A0J5JMB8	47	2201.4578
A0A0J5QET7	73	2664.1128
A0A0J5QXF6	36	2042.0623
A0A0J5TLL7	1	2133.0139
A0A0J6CHW8	50	2037.5107
A0A0J6DJV4	7	4813.0713
A0A0J6FY31	39	2103.1609
A0A0J6GGH7	50	4278.7544
A0A0J6XGP7	51	2328.4387

A0A0J6XMR6	44	2207.3303
A0A0J6XU50	33	4276.6802
A0A0J7AKL5	22	2451.7593
A0A0J7HLK2	27	2857.5662
A0A0J7XQ01	3	2694.5271
A0A0J7Z7J6	90	4232.8262
A0A0J7ZD37	17	2427.1223
A0A0J8A072	64	2910.4929
A0A0J8A2L0	44	2337.3362
A0A0J8AQ73	58	3890.9255
A0A0J8AQ77	30	4172.6123
A0A0J8AUG9	54	2544.262
A0A0J8BU96	24	3148.6838
A0A0J8CLR4	90	2668.4714
A0A0J8CRP3	20	2661.478
A0A0J8CZL6	78	2490.0938
A0A0J8VU23	74	2596.061
A0A0J9D3P9	91	2715.7563
A0A0K0I0D8	49	2945.8503
A0A0K0I0Z3	29	2571.7175
A0A0K0I289	76	2659.3665
A0A0K0I4N2	70	2696.6775
A0A0K0TRD4	76	2636.5923
A0A0K1E0N6	40	4782.561
A0A0K1JFK2	5	2630.1697
A0A0K1JFX1	54	2419.1482
A0A0K1JLL6	61	4352.9062
A0A0K2ART6	36	2914.2151
A0A0K2ASA3	86	4208.5332
A0A0K2E2E4	68	2869.4133
A0A0K2FD16	74	2200.249
A0A0K2FDL8	53	2116.9629
A0A0K2RIW5	77	2294.4812
A0A0K2SK68	40	2638.8105
A0A0K2YKI0	31	3215.7063
A0A0K3B3W1	25	3780.1511
A0A0K3BB21	49	4441.186
A0A0K3BGD4	65	2949.9141
A0A0K4AQD0	13	2571.2021
A0A0K6GS41	38	2725.3564
A0A0K6I4N5	18	2843.6218
A0A0K8JF42	48	2480.4524
A0A0K8P3N6	75	2412.1599
A0A0K8PDQ2	29	3341.3269
A0A0K8PEI1	48	4472.252
A0A0K8PQB4	2	4174.562
A0A0K8QB92	77	2294.4812
A0A0K9HVW3	52	3619.9812
A0A0K9R8Z7	43	2549.0183
A0A0K9X890	25	4358.3374

A0A0L0AP41	27	2666.343
A0A0L0H003	15	2611.666
A0A0L0JEL4	51	2280.0696
A0A0L0JGV9	76	3653.0146
A0A0L0JHM1	3	2674.8574
A0A0L0JIW0	97	2154.77
A0A0L0JVU3	83	2885.5493
A0A0L0K065	32	2440.6931
A0A0L0K9F0	31	4653.5137
A0A0L0KIU7	92	2839.1987
A0A0L0KNJ9	100	4529.4917
A0A0L0KU26	69	4169.48
A0A0L0KWS1	55	3090.4084
A0A0L0L3Z8	51	2907.6121
A0A0L0LVF3	1	2247.9304
A0A0L1I0Z5	29	2432.6782
A0A0L1JVB2	10	2695.0652
A0A0L1LFK6	60	2601.7959
A0A0L1LVD4	28	3695.1201
A0A0L1MTV9	24	2018.0481
A0A0L1MUX2	57	2360.0291
A0A0L6CF69	55	4562.7573
A0A0L6K173	83	2316.8936
A0A0L7BT19	69	2470.6228
A0A0L7BTV1	82	3841.45
A0A0L7CC49	92	2241.4729
A0A0L7CUF9	65	3814.7263
A0A0L8B0R6	55	2912.3379
A0A0L8BHM2	53	2597.4766
A0A0L8F3N5	22	2476.001
A0A0L8JAM5	35	4076.6523
A0A0L8JEN6	17	5667.5884
A0A0L8JI51	53	2332.2014
A0A0L8L9U3	14	2231.6243
A0A0L8LCW7	75	4467.9834
A0A0L8LF50	75	4001.179
A0A0L8LPE2	46	3807.6741
A0A0L8LRR7	3	4003.7617
A0A0L8LXE6	59	2352.6389
A0A0L8M4U2	66	7711.5503
A0A0L8N1U2	86	4348.5244
A0A0L8NBA6	44	2337.3362
A0A0L8NR76	48	4816.3643
A0A0L8NWB2	58	3890.9255
A0A0L8P752	50	2526.6719
A0A0L8PA08	44	4908.9399
A0A0L8PBL5	38	4422.9448
A0A0L8PFU2	95	2864.1062
A0A0L8PRD7	98	3602.5073
A0A0L8PTA5	26	2381.1223

A0A0L8Q9D9	11	2753.3242
A0A0L8QR02	92	4798.3936
A0A0M0F8C3	60	3856.2107
A0A0M0ILU9	99	2557.7151
A0A0M0KJQ7	20	2202.1338
A0A0M0KQU0	92	2079.9084
A0A0M0T212	98	2011.6304
A0A0M0TGQ2	76	2427.5403
A0A0M0WIV8	16	3157.0786
A0A0M1JNQ3	95	2497.4255
A0A0M1NP20	35	2636.2485
A0A0M1Q006	15	2159.7175
A0A0M1QAN2	71	2203.7705
A0A0M1TAG1	57	2528.7561
A0A0M2CYF8	48	2559.228
A0A0M2GKD2	89	4453.4878
A0A0M2GZ61	22	2785.7144
A0A0M2GZY7	88	4030.1331
A0A0M2H0T8	64	6128.8794
A0A0M2H901	12	3698.7146
A0A0M2HAH2	7	1269.0206
A0A0M2HAV7	87	2352.3811
A0A0M2HC25	7	2101.8154
A0A0M2HCX3	42	2382.2075
A0A0M2HHG6	74	2579.9553
A0A0M2HKR7	22	3729.9351
A0A0M2HKV3	60	2608.927
A0A0M2HNU0	69	2525.7771
A0A0M2HVL8	16	2421.96
A0A0M2J855	77	2258.8467
A0A0M2J903	80	4204.9453
A0A0M2JM03	14	4227.0225
A0A0M2MHD7	57	2219.4485
A0A0M2MU01	70	4038.6643
A0A0M2MU32	23	3157.5212
A0A0M2N3D1	75	2689.7114
A0A0M2QQ93	29	2536.3438
A0A0M2RR28	28	3999.5139
A0A0M2RXG8	42	2494.7415
A0A0M2RXU4	65	2600.3755
A0A0M2S5L5	82	5458.1792
A0A0M2S7U2	63	5100.3799
A0A0M2T3Q2	10	2145.2832
A0A0M2T717	98	2548.3418
A0A0M2VK60	94	2714.2957
A0A0M2WQN6	24	2607.1077
A0A0M2Y0R3	31	2542.2932
A0A0M2YVG2	97	4045.2644
A0A0M2YZ00	74	3966.051
A0A0M2Z050	74	4141.917

A0A0M2Z648	38	2280.2283
A0A0M2ZFB9	27	2402.2952
A0A0M3AQ52	20	2732.1914
A0A0M3BGC8	86	2551.8091
A0A0M3DAJ0	80	2766.1196
A0A0M3GQ75	6	2395.4253
A0A0M3H2D5	73	2529.946
A0A0M3QKY5	29	4196.5093
A0A0M3QL38	52	2108.1262
A0A0M3QLZ5	36	3986.2542
A0A0M3T701	4	3974.0381
A0A0M3UGE2	59	2870.2222
A0A0M4CRD3	1	2649.4333
A0A0M4D7X9	4	2330.4299
A0A0M4DCS3	99	2759.1631
A0A0M4DNG6	35	3942.1523
A0A0M4DS89	31	2403.9797
A0A0M4DVV9	81	2991.5518
A0A0M4E5M2	14	3938.187
A0A0M4GBU3	19	2243.4292
A0A0M4H5T3	47	2574.999
A0A0M4MCU7	51	2340.8635
A0A0M4U7M4	33	2609.6941
A0A0M5IM31	78	2206.1221
A0A0M5LL83	68	2710.4648
A0A0M6XTZ2	17	2222.762
A0A0M6Y187	98	2457.0537
A0A0M6YJY7	98	2555.0195
A0A0M6YSW6	26	2354.3113
A0A0M7BCL0	3	2591.5962
A0A0M7QGT7	27	4160.4258
A0A0M7QHN9	94	2525.9326
A0A0M7QM17	37	2356.0037
A0A0M7R2Z5	75	2417.9412
A0A0M8JET1	99	2394.9106
A0A0M8K9L7	100	561.5416
A0A0M8K9N5	28	2360.6558
A0A0M8KEI8	73	2258.4302
A0A0M8QG56	3	5673.1069
A0A0M8QK34	29	4650.5596
A0A0M8QR72	62	2108.2205
A0A0M8QWT2	1	4673.2515
A0A0M8SDN5	52	4064.8062
A0A0M8SEZ4	10	2951.6421
A0A0M8SMA1	78	2293.874
A0A0M8SSG9	26	2242.8142
A0A0M8T2X6	59	2353.791
A0A0M8TIP7	69	4073.2578
A0A0M8TXZ8	51	2104.3408
A0A0M8U5W7	21	3992.0247

A0A0M8UI27	44	2179.4329
A0A0M8UQ50	18	2163.6355
A0A0M8V6K2	68	4207.0005
A0A0M8VAL7	19	2195.4697
A0A0M8VB47	21	3550.2749
A0A0M8VHV9	61	3045.7876
A0A0M8VJD7	2	2184.7427
A0A0M8VPF2	79	3386.5369
A0A0M8VZE5	47	2964.2053
A0A0M8W1N2	81	2337.7773
A0A0M8W781	26	4346.2705
A0A0M8WEL4	11	4695.8311
A0A0M8WHP2	20	2236.1931
A0A0M8WJJ4	27	3071.9795
A0A0M8WSX9	52	2223.439
A0A0M8WYZ7	26	4052.1233
A0A0M8WZR1	79	3313.314
A0A0M8X1X4	58	4230.0522
A0A0M8XHD0	81	2125.8579
A0A0M8XWG1	94	2711.9626
A0A0M8Y623	15	4046.0891
A0A0M8YEC2	36	2549.9646
A0A0M8Z1Z8	48	3225.8242
A0A0M8Z4E7	77	4217.6582
A0A0M8Z8E8	8	2901.0933
A0A0M9B721	43	2746.7583
A0A0M9CPC8	16	2387.083
A0A0M9CQ95	54	2142.7703
A0A0M9JGD5	77	2739.1731
A0A0M9VKZ6	49	4258.1274
A0A0M9XK83	68	4863.8535
A0A0M9XVN1	44	4892.2505
A0A0M9XZM4	39	2198.7561
A0A0M9YNV5	9	3933.3311
A0A0M9YP81	98	3602.5073
A0A0M9YWR2	20	4646.5962
A0A0M9Z7X1	24	4778.9658
A0A0M9ZAW8	31	4823.4824
A0A0M9ZF02	42	3963.3633
A0A0N0A0C0	52	3400.5112
A0A0N0A4A4	87	2937.072
A0A0N0A859	32	4210.2598
A0A0N0A8X6	48	3866.6826
A0A0N0AJ32	76	4784.9307
A0A0N0AL52	59	3594.4055
A0A0N0ALA8	44	2422.1003
A0A0N0AQ64	11	2188.7952
A0A0N0AST4	35	4418.7573
A0A0N0AWW8	85	4081.6333
A0A0N0B0F3	33	2169.958

A0A0N0CH46	29	4306.2305
A0A0N0CIB1	31	2330.3062
A0A0N0GQQ4	74	2443.9465
A0A0N0GVB2	19	2955.2925
A0A0N0H8Q2	88	4376.2061
A0A0N0HMH4	62	3115.6702
A0A0N0HQN8	53	2958.5464
A0A0N0HTJ2	34	2268.9761
A0A0N0J8C4	92	2186.6221
A0A0N0J8Y0	63	2493.0491
A0A0N0JP47	16	2215.3115
A0A0N0KBI6	76	4171.9766
A0A0N0KK57	14	2588.7371
A0A0N0MMB8	83	2302.2847
A0A0N0MVS6	29	2298.8247
A0A0N0S6N1	57	4356.812
A0A0N0S9E3	73	4451.9399
A0A0N0SC67	100	2346.627
A0A0N0SCB5	56	7602.2705
A0A0N0SHM4	95	2768.5251
A0A0N0SLS3	16	6342.1328
A0A0N0SPK0	40	2240.4131
A0A0N0T1Q2	5	3649.2632
A0A0N0T4C3	74	2801.7937
A0A0N0T5H1	64	2521.1943
A0A0N0TDW5	14	4200.8335
A0A0N0THA8	36	3333.3308
A0A0N0THX2	83	4323.7871
A0A0N0TL46	51	2541.998
A0A0N0TLW9	63	4401.4497
A0A0N0TPC5	98	4917.3691
A0A0N0TS10	41	4702.4014
A0A0N0UI50	18	2150.3284
A0A0N0VN29	84	1957.7095
A0A0N0Y7R6	83	4179.7944
A0A0N0YGY9	67	4443.7134
A0A0N0YI18	65	2338.8142
A0A0N0YNN0	76	2377.2192
A0A0N0Z262	3	2272.8628
A0A0N1AA72	99	2392.6492
A0A0N1AEE0	95	2455.7695
A0A0N1AFJ4	89	2844.0913
A0A0N1AU11	75	2430.2878
A0A0N1AXA3	77	2386.7512
A0A0N1DIY9	53	2364.3931
A0A0N1G7G6	92	2292.3997
A0A0N1GF50	11	3032.1692
A0A0N1GIB1	2	3768.0342
A0A0N1H0N8	79	2435.5886
A0A0N1H2Z5	81	2884.7625

A0A0N1IYI6	36	2292.1206
A0A0N1K8N4	27	4180.9136
A0A0N1KDT4	94	5256.4121
A0A0N1L3X6	100	2671.3291
A0A0N1L7J0	10	2293.0889
A0A0N1NAQ0	49	2868.8577
A0A0N1NBL9	45	2365.2437
A0A0N1NEX6	13	2343.0703
A0A0N1NI34	92	3429.7761
A0A0N1NTA5	98	2255.0698
A0A0N6ZNZ5	85	2969.9851
A0A0N6ZTF1	41	4253.5015
A0A0N6ZUJ3	84	2220.3354
A0A0N6ZV69	100	4591.4302
A0A0N7F2D9	84	2927.7009
A0A0N7FAE4	26	2185.4336
A0A0N7FUC0	60	2839.4375
A0A0N7I4H8	73	2747.9958
A0A0N7I902	65	2365.3872
A0A0N7JUH3	99	2475.5449
A0A0N8GNU0	10	2713.7256
A0A0N8GTC3	61	2655.4199
A0A0N8TWM4	52	2718.1931
A0A0N8TZN0	11	2318.4143
A0A0N8W654	41	2578.4531
A0A0N9I2T3	89	6866.2969
A0A0N9ID20	86	4326.3257
A0A0N9ZMP8	53	2341.7095
A0A0P0CRR6	12	2339.1602
A0A0P0D6B0	49	2629.231
A0A0P0EHD0	73	2423.8284
A0A0P0ERM0	86	2403.3726
A0A0P0F0Y5	1	2717.6199
A0A0P0MBL4	24	2153.8865
A0A0P0N9C0	8	3004.0581
A0A0P0NA10	23	3096.7864
A0A0P0NMM1	80	2238.3889
A0A0P0P0I4	85	2853.7795
A0A0P0U7Y3	49	2387.854
A0A0P1H107	8	2062.7615
A0A0P1JCR9	21	2438.8301
A0A0P4R6P8	58	3180.498
A0A0P4RAL1	25	2259.925
A0A0P4UKT9	67	2474.8408
A0A0P6TUV8	11	2827.394
A0A0P6TWT0	73	2158.0076
A0A0P6U1D2	91	3632.9629
A0A0P6U989	100	2624.085
A0A0P6UAH6	48	2456.8811
A0A0P6UGD3	74	2326.2109

A0A0P6W4L4	53	2066.0051
A0A0P6XFQ8	97	2164.7576
A0A0P6XSE8	55	2626.2415
A0A0P6XT49	12	2337.2336
A0A0P6XTN0	60	2527.0935
A0A0P6XZW3	17	2648.9106
A0A0P6YCA1	76	2709.2817
A0A0P6YDC3	14	2566.4871
A0A0P6YGK0	79	2538.8215
A0A0P6Z665	33	2539.5835
A0A0P7AJZ9	2	2249.1389
A0A0P7CMK7	85	3522.2705
A0A0P7DRH9	81	2575.6646
A0A0P7FFY7	95	2406.8184
A0A0P7WQB8	24	2346.624
A0A0P7XDC1	94	2304.4292
A0A0P7YNG9	98	2372.2585
A0A0P7ZLQ1	40	2258.8215
A0A0P8RGQ5	7	2457.0625
A0A0P8W829	72	3320.2319
A0A0P9CFM9	83	2994.4656
A0A0P9FDI8	34	2898.5579
A0A0P9GJ12	56	2683.3745
A0A0P9GL28	85	2319.8789
A0A0Q0GK92	47	2726.0093
A0A0Q0NVG9	1	2628.7712
A0A0Q0PYE0	8	2590.3896
A0A0Q0UUL0	67	4995.6304
A0A0Q0VJU7	33	2709.0403
A0A0Q0XT79	8	2517.6926
A0A0Q2UU82	78	5231.0781
A0A0Q2Y808	75	2210.0063
A0A0Q3X082	14	2269.9575
A0A0Q4DSU9	52	2428.3259
A0A0Q4FAZ5	33	4137.8359
A0A0Q4GK27	26	3059.3208
A0A0Q4H829	46	2471.0005
A0A0Q4H9L6	28	2553.321
A0A0Q4HCT3	57	3516.2263
A0A0Q4LI65	15	2453.1113
A0A0Q4PLV2	96	3080.6125
A0A0Q4PVH6	7	2396.0818
A0A0Q4RZP5	44	2360.22
A0A0Q4TNS4	7	2860.3147
A0A0Q5AXY6	27	2216.2439
A0A0Q5B5F0	37	2226.3569
A0A0Q5BW34	87	3922.0217
A0A0Q5C0E3	19	2235.8257
A0A0Q5C7T9	10	3049.1665
A0A0Q5CQA0	62	2462.1289

A0A0Q5DY39	59	2404.3411
A0A0Q5EKW2	49	2370.0605
A0A0Q5FPQ8	53	2676.0315
A0A0Q5FWL5	37	4142.4224
A0A0Q5H8U8	44	3096.2454
A0A0Q5HGK7	91	3234.5879
A0A0Q5I3J8	85	4482.2222
A0A0Q5I7L5	44	2370.386
A0A0Q5IFM2	76	2567.4968
A0A0Q5IZB9	86	4774.1445
A0A0Q5J8U7	50	3335.3513
A0A0Q5JYC8	49	2680.0452
A0A0Q5L579	51	4246.0767
A0A0Q5LAF9	56	2643.9148
A0A0Q5LN16	94	2636.6611
A0A0Q5LYA3	97	4084.5596
A0A0Q5M1X8	56	2990.2952
A0A0Q5MHL7	86	3024.0364
A0A0Q5N8P0	17	2574.3972
A0A0Q5NHD7	93	2685.6289
A0A0Q5PPH5	77	2546.9104
A0A0Q5QEF8	35	2635.2798
A0A0Q5TFA5	76	2087.6768
A0A0Q5TZT4	12	2381.7778
A0A0Q5UWJ7	26	2656.3345
A0A0Q5VGQ5	72	4049.5156
A0A0Q5VUS1	22	3172.2146
A0A0Q5VVI3	45	2231.9038
A0A0Q5W511	7	4155.5068
A0A0Q5WSI4	43	2908.8809
A0A0Q5ZL74	45	2838.5662
A0A0Q6A8N8	48	2504.2737
A0A0Q6G1R6	17	3713.3857
A0A0Q6G2C2	75	2704.0034
A0A0Q6G3J3	73	2775.3801
A0A0Q6HVV2	92	2638.1206
A0A0Q6I670	16	3157.0786
A0A0Q6MA37	35	2352.2771
A0A0Q6MCU6	20	2716.5229
A0A0Q6NPV4	65	2664.2319
A0A0Q6P061	21	2413.7678
A0A0Q6PNC8	56	2655.4858
A0A0Q6PPJ2	100	4964.6406
A0A0Q6PS41	1	3025.3835
A0A0Q6QMQ8	33	2354.5481
A0A0Q6QR31	55	2661.0803
A0A0Q6RH46	83	2384.1394
A0A0Q6RLN9	58	2558.8203
A0A0Q6RZ82	6	2484.55
A0A0Q6S7V2	56	2658.0908

A0A0Q6SCD7	62	2437.3914
A0A0Q6SHI3	83	2836.7534
A0A0Q6SJH3	9	2149.3616
A0A0Q6SYF4	93	2427.1987
A0A0Q6T1D3	84	2485.2976
A0A0Q6T930	85	2703.6958
A0A0Q6TBK9	52	3446.2803
A0A0Q6TLJ1	17	2702.4331
A0A0Q6TNJ5	56	2182.6775
A0A0Q6TWU7	22	3716.0632
A0A0Q6TZA9	57	2711.3972
A0A0Q6U8J4	17	2444.0762
A0A0Q6UY27	20	2333.7427
A0A0Q6V157	22	3716.0632
A0A0Q6VEF7	93	3881.0496
A0A0Q6VEW7	96	5280.6421
A0A0Q6VIU9	33	2412.5498
A0A0Q6VNH8	28	3933.2529
A0A0Q6VX85	51	2738.1353
A0A0Q6W596	11	3788.9634
A0A0Q6W5S0	98	2500.5913
A0A0Q6WIJ1	22	2389.2375
A0A0Q6WMT4	9	2448.4312
A0A0Q6WRY4	64	2464.0657
A0A0Q6WU06	35	2675.033
A0A0Q6WUH5	7	2504.5
A0A0Q6WXJ2	71	2727.1128
A0A0Q6X151	42	2232.4683
A0A0Q6X5W0	70	2637.0176
A0A0Q6X661	7	4395.6372
A0A0Q6X6S9	8	2381.4697
A0A0Q6X8T7	63	2491.6963
A0A0Q6XYF1	79	2433.7295
A0A0Q6XZ00	5	2510.0427
A0A0Q6Y5F7	46	2417.3101
A0A0Q6Y678	32	4679.3364
A0A0Q6YIM5	19	2309.95
A0A0Q6YR25	4	2239.9976
A0A0Q6ZTG4	4	2414.3955
A0A0Q7A3M4	10	2563.8667
A0A0Q7ARW6	82	2352.7683
A0A0Q7AXH6	100	2489.8442
A0A0Q7AXU6	62	2632.7981
A0A0Q7BD23	55	2339.4197
A0A0Q7BD61	14	2923.2053
A0A0Q7BHY9	96	2673.3694
A0A0Q7BKB2	25	4829.8633
A0A0Q7BR74	97	2720.5144
A0A0Q7DXY7	74	2384.9946
A0A0Q7E0M4	90	3682.604

A0A0Q7EUW1	47	2410.6912
A0A0Q7GH87	82	4457.1025
A0A0Q7GQM0	34	2428.4304
A0A0Q7HBJ5	78	2523.6538
A0A0Q7HH44	50	2098.0393
A0A0Q7HSM6	32	3003.751
A0A0Q7HUD3	46	2326.1394
A0A0Q7HY00	87	2594.7703
A0A0Q7I6P6	76	3653.0146
A0A0Q7J164	15	3003.2002
A0A0Q7K4S6	22	3876.2234
A0A0Q7KEA9	71	2364.6575
A0A0Q7KJD1	47	2629.3247
A0A0Q7KM67	10	2176.1572
A0A0Q7L146	89	3618.2512
A0A0Q7L731	88	4463.5688
A0A0Q7L9U0	12	2831.552
A0A0Q7LPS6	17	2287.7239
A0A0Q7MCA6	3	4691.7905
A0A0Q7MQI4	83	3087.2905
A0A0Q7NQ96	38	2405.3933
A0A0Q7QAP1	28	2259.7971
A0A0Q7QBW8	61	2576.9192
A0A0Q7RUG0	36	2512.7815
A0A0Q7RVS2	47	2638.0413
A0A0Q7S151	4	2486.3738
A0A0Q7SFW2	98	2573.5859
A0A0Q7SPH8	4	2323.7139
A0A0Q7SSI2	81	2456.6409
A0A0Q7SUU0	41	2504.0183
A0A0Q7T1L9	44	2263.6748
A0A0Q7T801	27	2618.959
A0A0Q7TBR9	98	3329.0159
A0A0Q7TCD8	43	2178.2673
A0A0Q7TR74	10	2176.1572
A0A0Q7U570	89	3618.2512
A0A0Q7U8Q1	71	2364.6575
A0A0Q7UH73	35	2694.1631
A0A0Q7UHV2	8	4415.1025
A0A0Q7UIX8	47	2629.3247
A0A0Q7US81	94	4503.1475
A0A0Q7V023	25	3229.9285
A0A0Q7V4M3	88	4463.5688
A0A0Q7VAY4	12	2831.552
A0A0Q7VW69	85	2683.3875
A0A0Q7W0B1	59	3020.2368
A0A0Q7WMB8	8	2332.6616
A0A0Q7X946	12	2553.6174
A0A0Q7XSS8	58	2435.8362
A0A0Q7Y9N4	7	2134.1768

A0A0Q7Z0P8	64	3115.156
A0A0Q8A5B5	23	2285.0259
A0A0Q8AIT6	17	2560.6494
A0A0Q8B8V1	17	2615.23
A0A0Q8CF54	82	4457.1025
A0A0Q8DC24	24	2678.624
A0A0Q8DHT9	26	2765.74
A0A0Q8E1S0	15	3003.2002
A0A0Q8E4G9	87	2594.7703
A0A0Q8E7L7	74	2364.1287
A0A0Q8E7R1	50	2098.0393
A0A0Q8EKW6	46	2326.1394
A0A0Q8EP60	76	3653.0146
A0A0Q8F3Y1	78	2523.6538
A0A0Q8F6H6	43	2420.9875
A0A0Q8F9X3	79	3559.6418
A0A0Q8GD26	82	2352.7683
A0A0Q8GG41	62	2632.7981
A0A0Q8GHX1	100	2489.8442
A0A0Q8GK83	55	2339.4197
A0A0Q8GMG1	14	2923.2053
A0A0Q8GU78	81	4363.8882
A0A0Q8H6P4	97	2720.5144
A0A0Q8HZT7	74	2364.1287
A0A0Q8IF10	69	3051.6028
A0A0Q8IYW4	35	2620.7253
A0A0Q8LU77	89	3932.0173
A0A0Q8N452	20	2389.6912
A0A0Q8N5B6	80	2337.1052
A0A0Q8NH52	79	2375.7781
A0A0Q8PNU2	42	2389.822
A0A0Q8PTI6	15	2239.6379
A0A0Q8PY62	26	2543.594
A0A0Q8PYD2	33	2783.6064
A0A0Q8PYN3	41	4851.2461
A0A0Q8Q0Z9	30	2227.4446
A0A0Q8Q1K8	11	2201.667
A0A0Q8RI14	52	2303.4031
A0A0Q8RPE3	6	2424.5283
A0A0Q8RPN9	55	2346.9263
A0A0Q8UIK4	96	2673.3694
A0A0Q8USD4	25	4829.8633
A0A0Q8VA65	96	2377.8188
A0A0Q8VGX6	78	2533.137
A0A0Q8VS90	71	2399.894
A0A0Q8VXC8	11	4326.374
A0A0Q8VXQ9	7	2658.3018
A0A0Q8W087	61	4397.5962
A0A0Q8WCX9	60	3954.373
A0A0Q8WWW6	71	2399.894

A0A0Q8XG34	78	2533.137
A0A0Q8YFH8	40	2556.1543
A0A0Q8ZE07	59	2242.2893
A0A0Q9A2C2	26	2457.2412
A0A0Q9B0L2	4	3817.5034
A0A0Q9BCM6	83	3902.1362
A0A0Q9CPY4	94	2940.5671
A0A0Q9CVH6	25	3850.2668
A0A0Q9CW60	42	4845.8208
A0A0Q9CXP9	85	2642.0664
A0A0Q9D8P1	20	2362.0962
A0A0Q9DF12	34	2748.0515
A0A0Q9DSX3	15	4069.7314
A0A0Q9E6X9	67	2377.3909
A0A0Q9EMW1	51	2673.3936
A0A0Q9IZQ1	34	2317.0334
A0A0Q9JM05	16	2525.928
A0A0Q9JNU3	97	4310.8301
A0A0Q9JVE0	94	2635.5586
A0A0Q9KWX0	85	2468.8616
A0A0Q9M540	70	4264.4351
A0A0Q9MDS1	90	4219.5439
A0A0Q9N1K2	6	2786.3936
A0A0Q9N6R4	42	3245.1284
A0A0Q9N857	97	2678.4346
A0A0Q9ND22	71	3501.0483
A0A0Q9NU55	84	2632.0442
A0A0Q9NW18	65	2985.8132
A0A0Q9P6A8	97	2424.9885
A0A0Q9PEY1	32	3574.8596
A0A0Q9PTK7	87	2767.2
A0A0Q9Q5T4	2	5356.7783
A0A0Q9Q6L3	28	3560.9277
A0A0Q9Q766	73	2582.0999
A0A0Q9QFX1	4	2720.5569
A0A0Q9QQN6	70	3623.6458
A0A0Q9R0X3	26	2447.7378
A0A0Q9RA48	43	2849.8818
A0A0Q9RIY6	28	2259.7971
A0A0Q9S1Z4	69	3707.6887
A0A0Q9S832	80	2393.0759
A0A0Q9SCY2	10	3214.2913
A0A0Q9SHL8	57	3942.3325
A0A0Q9SPK9	67	3942.6316
A0A0Q9SPL9	39	3185.6707
A0A0Q9T503	32	2604.5088
A0A0Q9T5A1	98	4426.0439
A0A0Q9TTG0	91	2487.8035
A0A0Q9U494	90	2582.9241
A0A0Q9UMU3	14	3058.6272

A0A0Q9UMW3	73	2852.845
A0A0R0CU61	17	2398.0156
A0A0R2P8F3	19	2669.4919
A0A0R2PFJ7	90	2599.4932
A0A0R2PMK7	51	2519.9197
A0A0R2PV35	89	2622.1794
A0A0R2QCF3	53	2248.0964
A0A0R2QH41	89	2622.1794
A0A0R2QM17	36	2220.2124
A0A0R2QPV0	89	2622.1794
A0A0R2QZI4	93	2666.3386
A0A0R2RI35	29	2689.8635
A0A0R2RRM8	19	2467.8889
A0A0R2SN20	67	2690.5684
A0A0R2SVI2	89	2622.1794
A0A0R2U6R1	89	2622.1794
A0A0R2XD05	19	2467.8889
A0A0R3C374	100	2475.9175
A0A0R3CQ33	19	2684.8972
A0A0R3DLK6	86	2573.0461
A0A0R3JS14	4	2080.8005
A0A0R3L252	10	2773.2051
A0A0R3LCP3	20	2778.6946
A0A0R3LCR8	70	2649.8557
A0A0R3LYE3	5	2347.9563
A0A0R3M9W1	35	2670.0186
A0A0R3MMQ8	63	3133.656
A0A0R3N3E9	53	2761.1711
A0A0R4J4H8	17	3031.0969
A0A0S1UK89	28	3882.7312
A0A0S1URP8	100	2379.2795
A0A0S1UWT1	34	2274.4419
A0A0S1XBC4	90	2506.968
A0A0S1YQV9	26	2766.8059
A0A0S2FAN7	54	2829.2515
A0A0S2FRN1	32	2620.0344
A0A0S2G7M6	75	2515.3025
A0A0S2JHQ8	68	2760.7703
A0A0S2NZV3	26	2924.7209
A0A0S2P002	43	4187.4556
A0A0S2P088	33	3926.7344
A0A0S2P9U1	88	2342.2893
A0A0S2PBQ0	86	2551
A0A0S2S780	37	2143.9114
A0A0S3AS57	33	2556.1997
A0A0S3FIH6	74	2571.2368
A0A0S3UBN2	73	2530.8389
A0A0S4KE28	33	2562.1421
A0A0S4MXN0	74	2412.3127
A0A0S4NTP3	9	2421.1313

A0A0S4P4S7	25	2322.3696
A0A0S4PIT6	60	2377.1702
A0A0S4PQL1	74	2412.3127
A0A0S4QTN7	91	3676.5066
A0A0S6UWR9	20	2753.4941
A0A0S6VXA0	48	2198.0403
A0A0S7ALL5	37	2388.8462
A0A0S7AN66	61	2809.9683
A0A0S7BW82	54	2486.0117
A0A0S7YLD5	59	2502.5916
A0A0S8B4Z3	56	2489.6062
A0A0S8EKQ4	5	2497.2681
A0A0S8EL39	13	2370.5176
A0A0S8IDN0	81	2516.0901
A0A0S9CMV1	49	2736.377
A0A0S9DCH0	53	2486.3362
A0A0S9DNH2	5	2654.748
A0A0S9DWR5	58	4329.5654
A0A0S9KM05	32	4483.9521
A0A0S9M311	83	2474.7239
A0A0S9PEN9	44	2682.2561
A0A0S9Q9E5	40	2656.907
A0A0S9QPS4	58	6049.6016
A0A0S9R7Q3	64	2425.7764
A0A0S9RAU0	29	2638.5027
A0A0T0MLA0	55	4693.6787
A0A0T1Q6H1	37	2297.9639
A0A0T1QDX5	89	2297.8586
A0A0T1QEB7	36	2367.7207
A0A0T1S683	13	3841.5962
A0A0T1SCL4	85	2350.103
A0A0T1SJX2	68	3604.8457
A0A0T1SL12	98	2250.1238
A0A0T1U9U5	25	2252.3784
A0A0T1ULF9	76	2982.3577
A0A0T1UM92	34	3848.0215
A0A0T1WPD0	87	2712.1086
A0A0T2IK19	92	3916.8027
A0A0T2KUG9	94	2352.8052
A0A0T2L2H1	46	2722.0916
A0A0T2MCU7	58	2986.6206
A0A0T5PLD9	32	2471.5012
A0A0T5VRL9	14	2419.9324
A0A0T5ZXI6	21	4530.1509
A0A0T6ALV9	24	2702.5132
A0A0T6LP89	29	3974.344
A0A0T6LRD4	83	3692.0386
A0A0T6LXE1	49	2558.6238
A0A0T6UQT1	31	2750.731
A0A0T6YD22	32	2334.4929

A0A0T6YP47	32	2381.0452
A0A0T6YV65	32	2334.4929
A0A0T6ZA30	5	2343.04
A0A0T6ZU64	33	2443.1719
A0A0T7D0Q1	34	2549.2283
A0A0T7DE67	34	2598.0803
A0A0T7DJF3	98	2513.5334
A0A0T7ETW5	58	2554.2944
A0A0T7FNB3	55	3001.1704
A0A0T7FU31	78	2604.0073
A0A0T7GMG4	96	3108.8438
A0A0T7H874	18	3049.3757
A0A0T7JLE7	96	2826.748
A0A0T7JYV2	3	3010.5002
A0A0T7K5J3	50	2595.0115
A0A0T7NNF6	15	2829.8293
A0A0T7YD17	26	2804.4751
A0A0T7YEA9	29	3063.2419
A0A0T7Z081	55	2828.95
A0A0T7ZHF0	44	2894.5557
A0A0T8AC09	45	1731.9624
A0A0T8B005	93	2993.6218
A0A0T8B4P4	64	2924.5225
A0A0T8BKP6	20	2499.7524
A0A0T8BNH2	27	2936.957
A0A0T8BU07	5	2780.5286
A0A0T8BX03	96	2826.0735
A0A0T8CHH2	90	2875.8115
A0A0T8EH97	38	2976.4937
A0A0T8EII7	45	2605.0691
A0A0T8F0P2	98	3015.6304
A0A0T8II41	23	2820.8286
A0A0T8JGS2	75	2794.9187
A0A0T8JTT4	30	3004.0022
A0A0T8KCR7	93	3053.8982
A0A0T8M6A5	27	2633.3989
A0A0T8N3C8	91	1918.8129
A0A0T8PG43	48	2939.5999
A0A0T8PUS0	14	2725.9578
A0A0T8QCX9	35	2010.1698
A0A0T8QFQ2	41	2883.6218
A0A0T8SM74	25	3111.3984
A0A0T8SYL5	97	2811.3223
A0A0T8THB7	90	2599.97
A0A0T8TLC8	96	2922.9321
A0A0T8U444	38	2529.6245
A0A0T8UQJ1	33	2966.8984
A0A0T8VXI9	51	1136.7755
A0A0T8WD98	12	1000.6816
A0A0T8WZJ2	17	2643.9348

A0A0T8XAA3	44	3002.752
A0A0T8XSW7	47	1425.0347
A0A0T8XSY3	38	1110.2513
A0A0T9AER4	66	5899.5156
A0A0T9AH70	32	4500.7109
A0A0T9GUQ6	48	1215.594
A0A0T9IAX2	88	2828.2104
A0A0T9JWZ0	19	2497.3123
A0A0T9K4S5	6	3376.9302
A0A0T9K572	96	4637.7588
A0A0T9KSD8	7	4661.8145
A0A0T9MR01	43	4340.6274
A0A0T9MYM8	74	2274.134
A0A0T9Q4C1	2	2633.3044
A0A0T9QJD0	15	2829.8293
A0A0T9QSJ4	63	2777.8171
A0A0T9S6E5	91	2708.9438
A0A0T9T467	7	2586.6621
A0A0T9T6C9	80	2750.8049
A0A0T9TWD4	31	2716.624
A0A0T9UY74	18	2774.95
A0A0U0ASS4	99	3051.3997
A0A0U0B443	77	2814.0054
A0A0U0BFN0	42	3026.3511
A0A0U0BIG0	16	2927.9216
A0A0U0C9H4	4	2968.3984
A0A0U0CTK4	99	2765.0806
A0A0U0DGH1	36	2977.0193
A0A0U0F4G5	12	2767.5725
A0A0U0FKQ8	83	1709.6259
A0A0U0FVD0	86	2949.6558
A0A0U0FWW9	73	2986.6475
A0A0U0GY98	58	2490.9041
A0A0U0HLV8	85	1281.6858
A0A0U0KAV4	39	2773.541
A0A0U0KIB7	97	2459.7114
A0A0U0KVE4	14	2861.4285
A0A0U0KVE8	19	2971.8474
A0A0U0LRY2	87	2785.9204
A0A0U0MQ66	12	3011.3462
A0A0U0PAY4	73	2823.5847
A0A0U0WYT0	87	7136.0835
A0A0U0XYC0	79	7163.2769
A0A0U0Z2D9	65	1653.985
A0A0U1A3Y3	75	6629.2319
A0A0U1AB91	54	2749.6956
A0A0U1AD04	85	2766.8281
A0A0U1ASY1	48	2772.4268
A0A0U1AT11	33	2935.1826
A0A0U1AVQ1	16	2801.5579

A0A0U1B050	56	2673.4124
A0A0U1B430	34	2682.5559
A0A0U1BB08	55	6844.6108
A0A0U1BBI9	49	6778.7759
A0A0U1BE00	22	1182.4957
A0A0U1BIX4	24	6602.7466
A0A0U1NND8	38	2601.4663
A0A0U2PBX4	75	2176.053
A0A0U2XFH3	92	2947.3601
A0A0U2ZC99	60	2663.3374
A0A0U3EGQ1	72	2451.8188
A0A0U3EHY6	70	2827.7266
A0A0U3FRQ6	58	2421.8132
A0A0U3HG46	53	3691.5896
A0A0U3KF18	90	2215.1855
A0A0U3KJP0	52	2578.553
A0A0U3L7K8	56	2999.5071
A0A0U3LCB8	48	2369.0681
A0A0U3LHT9	74	3604.7493
A0A0U3LL56	87	2967.4734
A0A0U3LW38	53	2464.5564
A0A0U3N3Y9	29	2645.769
A0A0U3N6M7	38	3917.9087
A0A0U3P0Q0	89	3563.626
A0A0U3PI65	25	2225.53
A0A0U3QTC1	61	2915.9419
A0A0U3QV55	73	4121.4004
A0A0U3R8G3	51	2252.6565
A0A0U3RIE4	62	4143.8232
A0A0U3SAF1	29	4246.9546
A0A0U4B2Z9	53	3045.7839
A0A0U4C9Z2	85	2402.9082
A0A0U4GCW9	35	4208.0854
A0A0U4GRN2	88	2754.2275
A0A0U4W872	47	2826.6487
A0A0U5BL20	65	2529.6301
A0A0U5BSL5	34	2893.6611
A0A0U5FP09	76	2706.2554
A0A0U5H4F0	33	2836.4844
A0A0U5HFY8	61	2186.0173
A0A0U5HHD9	33	3345.8564
A0A0U5IDY0	47	2655.6873
A0A0U5J538	97	2319.3833
A0A0U5JDH6	77	2682.793
A0A0U5JEL6	98	3741.4875
A0A0U5M6X4	90	4082.8513
A0A0U5MJM8	8	2472.0593
A0A0U5NTI6	95	2749.3948
A0A0U5NVN5	5	2579.8682
A0A0U9H595	23	2013.7435

A0A0V0PQ32	94	2288.5781
A0A0V1R2M8	81	2375.1907
A0A0V2F7J8	76	4317.106
A0A0V8GEY5	6	1978.7225
A0A0V8GHV7	23	2362.615
A0A0V8HGU8	36	2042.1954
A0A0V8HU92	21	2200.7017
A0A0V8I648	73	2614.2856
A0A0V8IVW0	39	2895.4395
A0A0V8SNK2	70	4791.6064
A0A0V8TAB5	64	2761.3931
A0A0V9IX77	55	2586.5051
A0A0V9RYC4	16	2413.2988
A0A0W0YT16	41	2525.9854
A0A0W1BNW5	3	2736.4136
A0A0W1BP02	2	2372.3674
A0A0W1D8C6	75	2777.8376
A0A0W1DQ54	67	2424.6626
A0A0W1F394	63	2368.1045
A0A0W1KW42	9	2678.6987
A0A0W1KWY9	40	2564.7729
A0A0W1KX11	3	2514.8401
A0A0W1L3I4	28	2742.447
A0A0W1L3T4	41	2476.8093
A0A0W1ND10	73	3839.0483
A0A0W1NGA5	82	2230.6619
A0A0W1NHN6	78	4260.0601
A0A0W1NKP4	40	2243.002
A0A0W1NQW9	74	4639.2007
A0A0W7WMQ7	100	2715.446
A0A0W7X383	56	2308.7434
A0A0W7XBG1	84	2802.6509
A0A0W8ADG0	57	2528.7561
A0A0W8EKX1	54	2525.5999
A0A0W8EVK2	54	2666.948
A0A0W8I5T1	37	2428.4565
A0A0W8IIB6	18	2462.9666
A0A0W8IMR8	70	2519.6318
A0A0W8JD77	95	2553.3145
A0A0X2PCT3	27	2346.9985
A0A0X3R7W8	44	4908.9399
A0A0X3RDA4	38	4422.9448
A0A0X3S6R1	67	3274.2131
A0A0X3SEI7	15	2279.3799
A0A0X3SKD1	46	2371.9841
A0A0X3SP08	29	4735.4585
A0A0X3SV59	29	4603.5469
A0A0X3U6F4	32	3457.3779
A0A0X3V0H3	77	4123.7388
A0A0X3VQR3	58	2539.3191

A0A0X3W714	10	3974.0261
A0A0X3W9T3	35	2827.7539
A0A0X3WHC3	47	2493.1785
A0A0X3WSQ3	85	2488.8855
A0A0X3WTY9	32	2230.0149
A0A0X3WVZ5	65	3873.7236
A0A0X3XDC3	89	4437.627
A0A0X3XGT4	95	3084.009
A0A0X3XP64	9	4041.0874
A0A0X3XPM3	90	4204.4966
A0A0X3XQQ6	2	2364.0647
A0A0X4JTP1	3	2635.5869
A0A0X6HAV7	24	2432.7009
A0A0X7A059	58	2417.1926
A0A0X7JGE3	8	4460.1587
A0A0X7JPM1	72	4826.4346
A0A0X8LK10	12	2727.8367
A0A0X8RL12	20	2677.3752
A0A0X8SJP0	10	2611.6001
A0A0X8X3L2	13	2449.7354
A0A0X9B453	3	2767.6475
A0A0X9CC82	64	2825.8162
A0A0X9UAT9	81	2645.8203
A0A0Y0C405	53	2950.8674
A0A0Y0KCV2	75	2636.3118
A0A0Y0M4F4	52	2606.8984
A0A0Y0P6F3	29	2396.6299
A0A0Y1UNU7	30	2699.4084
A0A0Y3JN85	47	2773.2847
A0A0Y4EJZ0	7	2513.1204
A0A0Y4F8K9	88	2741.9849
A0A0Y9CRS5	84	2603.5701
A0A0Z5BZ97	29	2631.2573
A0A0Z5Q194	4	2976.7578
A0A0Z6YG58	86	2966.0234
A0A100CLM4	52	2656.8564
A0A100HNZ0	56	2391.7234
A0A100J4D6	55	3090.4084
A0A100J4D8	71	3019.7776
A0A100J537	64	3955.0042
A0A100J6F7	98	4689.6338
A0A100JCY1	47	2868.0747
A0A100JPB7	55	3090.4084
A0A100JPU0	36	2865.6514
A0A100JTN4	30	2683.3318
A0A100JTZ6	62	4258.1865
A0A100JXZ0	14	4206.8921
A0A100VQ06	68	2202.7449
A0A100Y2V7	25	2232.6829
A0A100Y3R7	12	2495.3774

A0A100Y7K5	14	3663.4031
A0A100YA05	79	2256.793
A0A100ZF69	18	2920.1836
A0A101AMM3	57	2382.8105
A0A101BU84	24	2956.3108
A0A101EQH9	67	2223.0017
A0A101I5U2	5	2428.7092
A0A101IMH3	88	2296.8462
A0A101JF42	62	4951.4551
A0A101JFL3	57	4021.6101
A0A101JT74	74	2718.8672
A0A101KYQ6	1	2250.5488
A0A101L4W0	28	2602.5015
A0A101LBH8	29	2899.4827
A0A101M6I5	72	2679.5537
A0A101N010	73	3966.5261
A0A101N9T5	38	2316.8672
A0A101NCB0	10	2291.196
A0A101NDF9	12	2284.1128
A0A101NFG4	24	2967.4758
A0A101NKE4	62	3979.6748
A0A101NSI8	61	2277.1377
A0A101NXW0	100	4193.2603
A0A101P7I9	95	2508.4014
A0A101PJZ1	77	2209.8726
A0A101Q455	6	2293.4185
A0A101QFE1	56	3050.6519
A0A101QHN0	97	2391.1677
A0A101QJH3	90	2538.0623
A0A101QKT1	50	2326.9407
A0A101R1A6	45	4187.3105
A0A101R2P9	11	2288.186
A0A101RD42	13	2879.3672
A0A101RI91	36	2210.7192
A0A101S675	80	2456.4846
A0A101S8C8	89	4072.5286
A0A101SET1	11	4408.271
A0A101SG54	28	2261.8428
A0A101SYK5	74	4170.7515
A0A101SZN6	21	4001.7627
A0A101T0Q8	80	2758.4924
A0A101TCI3	67	2292.8757
A0A101TH24	56	2835.2205
A0A101U0K9	37	4160.1411
A0A101U6D1	82	2517.5623
A0A101V084	55	2287.2815
A0A101V4H0	1	4001.0361
A0A101XZP8	17	2210.7053
A0A104NM65	74	2708.2297
A0A108U516	32	2620.0344

A0A109CDY6	26	2424.5205
A0A109CSG1	76	2595.6218
A0A109CYL7	91	2240.6641
A0A109II49	66	2447.4529
A0A109J7P9	83	2376.3083
A0A109QK50	56	2124.7336
A0A109WPC7	64	2535.5588
A0A109YN89	38	2423.0581
A0A110AVA9	6	6710.793
A0A110E8P6	100	2704.8535
A0A110RL77	3	3005.7551
A0A111FXJ5	61	2932.8188
A0A111XY92	4	2993.2329
A0A117DMM0	98	2440.7278
A0A117EB11	29	2482.9587
A0A117IUM8	90	4036.2764
A0A117KA82	59	3821.2896
A0A117KEY2	95	2420.3708
A0A117L2T0	56	2191.6211
A0A117L4W2	71	2464.1365
A0A117L8X6	77	2124.9185
A0A117M3L0	1	2533.9583
A0A117MJG3	43	2971.4399
A0A117NTJ8	84	2203.3323
A0A117P3R8	79	4124.229
A0A117P4A6	1	3960.4111
A0A117PCI5	37	2299.5872
A0A117PRT8	86	2429.9526
A0A117PTH4	79	4320.2871
A0A117Q144	90	2231.5408
A0A117Q2M6	49	2287.7825
A0A117Q6Z6	93	4278.1333
A0A117QH84	1	3995.7378
A0A117QJQ4	22	4235.2935
A0A117QML6	55	3990.613
A0A117QPQ7	13	2212.7664
A0A117R0S1	51	2232.2236
A0A117R8V6	67	3138.479
A0A117RCC8	70	4200.1309
A0A117RXI0	82	3002.4641
A0A117SXL6	15	2221.0469
A0A117V8E6	26	2659.4092
A0A117V914	13	2609.1133
A0A119CYV6	62	2533.9607
A0A120F783	53	2276.3325
A0A120F862	33	4953.2632
A0A120F8N8	9	2615.7925
A0A120FLV9	94	2521.0564
A0A121JEF9	99	2788.2217
A0A124C423	29	2482.9587

A0A124GVZ2	39	3977.1143
A0A124GX52	80	2456.4846
A0A124H6G3	16	3551.3364
A0A124HFX4	98	3319.0017
A0A124HPG9	33	3926.7344
A0A124HPM6	20	2819.7239
A0A124HVR2	35	2462.7102
A0A124HZ19	82	3962.688
A0A124ID14	84	4123.396
A0A125T0U2	7	2583.7773
A0A126PGE0	81	2454.6592
A0A126Q4N7	77	2750.6501
A0A126Y5X3	23	3884.575
A0A126Y788	98	2494.3296
A0A126YDG3	32	2230.0149
A0A126YS63	6	2406.4387
A0A126Z4Y4	33	4047.7163
A0A127A2M4	94	3135.2319
A0A127CMC6	36	2784.7734
A0A127D520	92	2656.3318
A0A127N547	56	2629.7317
A0A127SA86	100	2364.478
A0A127VGJ3	77	2334.9929
A0A132BZI3	7	2588.7373
A0A132N7E2	10	2575.7073
A0A134DEC1	10	2749.5273
A0A135IMR7	14	2375.1335
A0A136H420	81	2785.4563
A0A136I0T0	33	2761.5837
A0A136I1I5	37	2645.9602
A0A136JUN2	57	2403.6714
A0A136P200	61	2731.7629
A0A136PLI8	38	4739.9551
A0A136PQ79	90	3778.3528
A0A136PR14	61	2597.4458
A0A136PSV5	98	4389.2847
A0A136PT97	100	2499.2783
A0A139C0C4	14	2616.2803
A0A139M7S9	81	2587.5811
A0A139ME62	41	2478.241
A0A139MES3	31	1154.6525
A0A139MFT5	39	2567.5808
A0A139MFY5	92	2545.2117
A0A139MLS4	30	2233.2903
A0A139MRB6	100	2773.5005
A0A139MY13	22	2775.4443
A0A139N452	65	2799.0488
A0A139NB07	48	4503.6143
A0A139NE14	76	2799.6008
A0A139NFW8	79	3082.8979

A0A139NTA1	88	2949.7031
A0A139NW12	19	2738.7253
A0A139P1Y0	68	2743.6487
A0A139P5P8	64	2713.6294
A0A139PC59	29	3013.0471
A0A139PFS0	87	2763.4946
A0A139PHH0	71	1624.3822
A0A139PLL6	40	2609.5874
A0A139PVD8	96	1876.1902
A0A139Q539	52	2597.8103
A0A139Q647	98	2450.9143
A0A139QFA8	68	3033.3792
A0A139QFH1	7	2672.2461
A0A139QJB7	64	1580.5232
A0A139QSZ8	56	2620.6094
A0A139QTD8	19	3359.9094
A0A139QXT6	13	2618.7429
A0A139R2H8	74	1694.4476
A0A139R3I5	54	2430.1663
A0A139R6K5	88	2841.6733
A0A139R9R9	81	2763.0288
A0A139RE39	29	2771.2537
A0A139RP68	89	2772.9138
A0A140JXI1	29	2843.5442
A0A140JXI4	88	2887.407
A0A140JXI6	20	2751.9185
A0A140JXI7	58	2602.9209
A0A142HL66	31	2478.3379
A0A142HV74	60	2382.3347
A0A142KE92	6	2539.218
A0A142KH94	46	2281.6501
A0A142LEP2	98	2158.3782
A0A142Z2D1	31	2465.6262
A0A143BYV7	85	2992.3137
A0A143C0M6	100	4041.3643
A0A143CBS3	60	4464.5117
A0A143XQ59	78	2647.2588
A0A143Z9N1	26	2821.1531
A0A144MT99	99	2528.2532
A0A146G1V6	82	2807.1394
A0A146G4C1	41	2620.2434
A0A146G7S6	48	2588.0476
A0A146GB03	38	3680.8738
A0A146GB87	25	2707.4143
A0A146GBB3	42	3456.4473
A0A146GCV4	91	2682.0701
A0A147DHV1	80	2029.7609
A0A147DK95	23	2362.615
A0A147E1P0	97	2798.3359
A0A147EWX1	25	2487.385

A0A147HV64	39	2914.2659
A0A147IX49	60	2847.5662
A0A147J2G4	81	2900.4302
A0A147JDD4	84	2838.6782
A0A147KBW9	52	2037.4073
A0A147KFJ2	11	2464.1431
A0A147KMZ8	7	4338.8657
A0A148KN47	19	2414.2092
A0A149PEU0	23	2796.6299
A0A149PG68	2	3197.2852
A0A149W1J7	80	2421.1572
A0A150HHU3	49	2774.0596
A0A150JWH8	88	2736.5667
A0A150KLU3	63	2672.9609
A0A150KUW4	62	2656.7563
A0A150L0E6	78	2535.6899
A0A150LBY8	12	2744.5457
A0A150LGF5	10	2606.0933
A0A150LIB8	31	2679.2207
A0A150LW25	46	2796.8398
A0A150LXU1	66	2959.9631
A0A150LYB2	34	2778.9231
A0A150M9Y2	73	4033.0039
A0A150MKW7	61	2541.1208
A0A150NE90	32	2419.6514
A0A150NKB0	36	2780.9382
A0A150NUT2	88	2770.5
A0A150PSM8	61	2340.6587
A0A150PV36	75	2578.0625
A0A150PZ21	50	2673.9319
A0A150Q700	59	2590.7239
A0A150QHI3	48	2673.4976
A0A150QY19	56	2663.7864
A0A150REQ2	73	2804.2607
A0A150RSE8	65	2664.6699
A0A150RWY2	77	2862.011
A0A150S4Q9	32	2671.9014
A0A150SAJ0	11	2654.9905
A0A150SQJ4	81	2597.6814
A0A150SS85	36	2659.4302
A0A150TFS4	17	2572.571
A0A150TYH0	31	2651.7288
A0A150UJJ5	72	2606.8823
A0A150VR81	93	2218.4084
A0A150VRJ4	58	4458.5439
A0A150YZ37	54	2147.1692
A0A150YZH1	65	2229.1594
A0A150Z5A9	85	2382.1255
A0A151BU23	21	4091.2935
A0A151C116	9	3457.8811

A0A151CU35	75	2656.2285
A0A151D612	20	2308.5222
A0A151D8A3	49	3037.7124
A0A151DH08	63	2243.145
A0A151DI30	48	2307.1653
A0A151DN79	58	3171.9045
A0A151FDK9	23	2852.2476
A0A151FUL3	84	2798.9038
A0A151QVT5	35	1924.9617
A0A151QXB7	4	2356.4021
A0A151REC3	5	2558.1846
A0A151RKM9	81	3328.26
A0A151UAQ5	8	2418.2817
A0A154IS38	8	2389.7781
A0A154KSN9	65	2661.7512
A0A154L8Z0	66	2750.3125
A0A154MP58	46	2088.5613
A0A154MPI7	3	3178.7534
A0A154MSG3	47	3000.2639
A0A154MUI7	38	2319.345
A0A154NB93	36	2738.8286
A0A154QHN7	34	2734.2634
A0A154R397	76	2448.2009
A0A154UZW3	50	2670.6343
A0A154VLE0	62	2763.1064
A0A155TTI4	95	3442.76
A0A155V2V5	44	1382.5541
A0A157TCL9	68	2567.9329
A0A157TG10	25	2476.8762
A0A157TWG0	76	4848.0371
A0A157TYJ0	73	2529.946
A0AC59	8	2288.2261
A0AF66	69	2644.1504
A0AF81	56	3754.1663
A0AFB1	39	2683.5027
A0AG26	52	2872.1414
A0JZ86	32	2690.3547
A0K0K0	71	2967.1956
A0KK20	41	2637.2026
A0LR48	7	4641.8203
A0NVK9	4	2416.4641
A0R2K0	36	4642.2354
A0XZR8	38	2748.531
A0XZU6	16	2306.3267
A0YUE1	75	3031.9529
A1C3J9	22	2815.4866
A1JNB7	18	2987.4001
A1R103	39	2366.5493
A1R8U7	12	4234.5483
A1S5F3	62	2841.9358

A1SNN0	78	2607.6333
A1SQJ7	44	3152.8762
A2QGL6	4	2364.2134
A2QID8	21	3777.2971
A2RHQ9	93	3628.3032
A2RJU6	65	1019.6784
A2RJU7	30	1162.4318
A2RJZ1	80	1127.4987
A2RK29	48	2916.3335
A2RLY4	53	2764.1516
A3CN02	20	2542.5576
A3D1N8	30	2367.0874
A3DFD0	66	2570.9089
A3JMJ0	60	2535.3564
A3JYS5	16	2315.1541
A3KIN3	31	4270.1567
A3KK08	31	2939.6995
A3PJW4	72	2436.4502
A3TGZ0	45	2553.8616
A3UQH3	18	2526.0437
A3V9E7	39	3780.8391
A3VMT2	54	2595.7173
A3WL20	50	2623.9663
A3YHU8	85	2534.1714
A4AF25	67	2645.8511
A4AFR4	62	4187.0742
A4AK24	51	4392.5225
A4BJP2	38	2530.6763
A4C562	53	2670.7737
A4F947	32	2526.8369
A4FH46	61	2516.0095
A4FKW1	30	2368.7192
A4U0J3	92	2550.9282
A4U5G3	26	2565.5681
A4WRN3	61	2392.1287
A4X1R1	89	3776.1663
A4X576	15	4799.6143
A4X939	73	2278.3735
A4XIG7	73	2334.2168
A4YNI1	83	2782.3218
A4YUN3	27	2851.9705
A5CM23	34	2429.5615
A5CPC2	64	2586.2439
A5CT94	75	2841.0264
A5EJ08	36	2939.4998
A5EQE2	83	2585.1187
A5FAA5	99	2544.219
A5IL97	67	2223.0017
A5KN03	42	2776.5251
A5KVQ8	46	2549.7317

A5UZB6	47	2454.0725
A5ZMW4	48	2968.5625
A6AMQ2	69	2648.5586
A6CVW9	55	2995.8267
A6DGU2	38	2814.1023
A6DLV2	77	2831.5913
A6DUB8	85	2559.7422
A6EHL7	92	2401.5771
A6GCT8	43	2508.1343
A6LNI1	32	2798.2283
A6LTH5	83	2564.8555
A6M001	4	3001.9155
A6M245	82	2452.6096
A6T9X1	29	2536.3438
A6UD86	65	2446.3101
A6V4K6	11	3790.4805
A6W3B1	24	2065.425
A6WF35	88	4594.3091
A6X2M0	16	2463.0676
A7HN90	5	2695.9124
A7N5G4	79	2741.1277
A7NGQ8	46	2434.4507
A7ZAD8	80	2691.1016
A8AZ19	8	2578.4292
A8GA02	99	2735.1794
A8GJM5	9	2608.4397
A8L4K2	7	2853.7092
A8LUQ2	67	4729.3657
A8LZF3	2	3749.3047
A8R876	11	2825.27
A8S1H9	71	2324.1755
A8TVR1	90	2491.1663
A8WAC9	74	2057.626
A9AYR3	24	2464.9536
A9AYT4	53	2621.7292
A9BFQ9	14	2426.885
A9D1X4	7	2872.7356
A9F279	88	2571.0388
A9F5S4	44	2691.147
A9GNP8	52	2938.6362
A9L506	82	2310.8286
A9VS04	5	2500.3748
A9WAY6	45	2503.2756
B0C9B0	95	2491.957
B0K276	84	2716.3589
B0KCV1	76	2098.6162
B0KCW7	84	2716.3589
B0KDF9	29	2097.7722
B0N3K6	53	2488.9795
B0N8V4	37	3166.6091

B0RIF2	6	4479.8232
B0T4Z7	60	3519.8044
B1FT15	89	2730.7571
B1IAA0	74	2970.6975
B1KHD2	88	2745.0547
B1VL16	97	3618.1648
B1VQ99	63	4083.8042
B1VSM7	88	2226.7151
B1VW56	100	2421.3877
B1VWD4	73	2750.3557
B1VZ46	3	4476.6729
B1XZK8	8	2711.1819
B2J7D8	30	9268.2451
B2JNV8	90	2488.7898
B2TFW7	47	2728.0364
B3Q0Q7	93	2408.1694
B3Q9I7	13	2801.9221
B3VE09	87	2707.3877
B4AJV7	32	2537.6392
B4AK23	69	2834.5984
B4ALA4	42	2873.5447
B4AMR5	82	2813.04
B4V9A8	84	6620.5527
B4VCY4	15	2139.6074
B5FAU8	80	2691.6602
B5GET9	61	3988.6096
B5GF53	13	2278.1707
B5H6C9	57	2784.5564
B5HAV3	29	4196.5093
B5HHQ0	78	2206.1221
B5HNI5	44	3970.6189
B5HXI9	90	4743.8682
B5I181	27	2510.7585
B5I7D1	57	2373.4773
B5IQL4	15	2579.8772
B5JPY7	85	2813.7742
B5JPZ5	93	3003.4539
B5M9E4	40	2734.1328
B5WE49	98	2731.02
B5WNC7	37	2839.4275
B5XWQ4	34	2401.2344
B5XXA4	77	3063.895
B5YAN1	75	2570.1062
B5YCI2	89	3803.4941
B5ZQU8	29	2318.9348
B6AXQ7	21	2658.1633
B6G7Y7	57	2517.3215
B6QX09	27	2273.0071
B6ZKM3	89	3323.4092
B7GM33	81	2435.4473

B7GU31	54	3236.9683
B7IEC2	86	2732.6287
B7JD71	65	3043.7463
B7LVM8	100	2608.6919
B7QXQ6	75	2476.7395
B7VTD4	95	2828.0974
B7Z536	21	2634.1763
B8A1R1	65	2332.6201
B8CL86	7	2513.2534
B8CYA8	92	2129.3499
B8D213	2	2300.6267
B8DVE3	16	3695.0566
B8E0Q1	12	2529.1958
B8E1X9	41	3711.783
B8EBW4	24	2456.9231
B8HA17	49	2611.2434
B8HD08	94	2940.6084
B8HM35	71	2651.2476
B8I5U2	3	2537.4016
B8M2Y0	61	2520.0913
B8P2C6	12	2432.6763
B9HYG4	19	2568.1311
B9JAE6	51	2396.2583
B9JSR2	33	2462.7544
B9K076	92	2238.3184
B9KSP3	90	2312.1091
B9L147	90	2732.8962
B9MNR1	99	2344.0525
B9RAJ2	20	2497.8125
B9RAJ3	66	5935.228
B9REF8	35	2377.613
B9REG8	91	1872.8601
B9REG9	73	2543.5337
B9REH3	60	2277.0281
B9REH5	31	2032.9163
B9RI70	20	2599.8154
B9RI71	55	2786.9084
B9RM06	1	2500.4478
B9RWJ7	56	2758.4133
B9RXP7	84	2429.7251
B9S3R8	31	2830.1101
B9S3R9	13	3023.4355
B9SAQ1	57	4111.6807
B9SAQ2	39	2774.2869
B9SAQ3	9	3771.7087
B9SAQ4	52	4451.1421
B9SAQ5	65	1108.7123
B9SAQ6	6	4106.7129
B9SY45	67	2839.0159
B9T4F7	51	2604.8213

B9V8P5	78	2193.4839
C0FVV0	22	3253.1914
C0PT85	69	2510.9409
C0X123	54	2673.4795
C0X4F7	19	2742.3943
C0X5J2	54	2749.6956
C0ZYY4	5	2582.6665
C1ATZ8	76	3392.2695
C1C5U9	55	2979.0327
C1CXP6	74	2521.0173
C2E3G3	72	2888.5903
C2EKY3	61	2514.4978
C2EMN9	13	2805.6641
C2F9E3	79	2540.3635
C2FAB9	97	2705.3745
C2G3Q4	46	2597.8035
C2JTN8	20	2596.3235
C2K0R7	93	4366.5615
C2LSG6	35	3093.7043
C3JLK0	40	2569.5962
C3M8X6	73	2398.1318
C4ILL3	61	2792.3721
C4L0E9	27	2777.0459
C4L1S4	81	2037.4928
C4L1S9	11	2354.5906
C4L9V0	17	2702.4585
C4LD42	8	2596.875
C4LLI8	49	3668.9653
C4RCW7	29	3586.7158
C4RJD4	43	4440.3486
C4VL63	11	2728.8247
C4VNB6	96	2879.4668
C4Z6T5	82	2592.8389
C5BYX0	83	2756.1482
C5CHI5	95	2510.2576
C5ENY1	22	3386.6204
C5EP49	24	3284.3469
C5JB25	69	2542.427
C5JB26	78	2532.8547
C5WI66	94	2440.3464
C5XFD2	73	2867.2725
C6A200	95	2288.6572
C6AWP7	34	2402.6816
C6CZG8	10	2958.8318
C6D9L1	75	3033.2698
C6DGK6	15	2832.4822
C6PVA7	35	2768.3921
C6R4W1	67	2684.5215
C6W5Y0	62	2135.5842
C6WCL5	27	4067.8618

C6WCW9	78	2720.7717
C6Y1A3	91	2548.2563
C7BJX2	93	2771.6089
C7D674	8	2729.0452
C7IQT1	42	2446.0269
C7PAW2	95	2300.2151
C7PXI2	85	3935.1184
C7Q825	35	4237.9097
C7Q8K7	9	2555.2468
C7QZ99	50	3519.5603
C7QZW4	98	2801.7476
C7RFE4	81	2620.2417
C7XXG7	25	2804.2139
C8S0J3	23	2770.8845
C8SYI3	9	2464.2327
C8TAN5	85	2624.3889
C8WXU5	28	2735.3257
C8XJ34	18	4696.5864
C9D1B8	77	1332.1416
C9QH22	72	2663.1301
C9XUV9	72	3258.9133
C9XZL5	49	2876.5713
C9Y3J9	52	2841.0923
C9Y5F8	86	2482.8013
C9YC48	55	2659.6646
C9Z448	43	4248.0498
C9ZAQ5	95	2842.7363
C9ZB09	32	3023.5713
C9ZEJ6	87	4210.1553
D0D2N8	88	2572.2153
D0KIH4	38	3079.3391
D0KKZ7	56	2297.2224
D0VEC8	8	2267.9194
D0VLH9	33	2016.3722
D0VYR5	99	2532.7651
D0VYR6	97	2575.3386
D0VYR8	86	2567.0142
D0VYR9	96	2522.043
D0VYS0	56	2531.7749
D0VYS1	23	2568.5122
D0X5D9	75	2477.616
D1A1R2	48	2873.8838
D1A786	27	2514.1892
D1AC41	68	2434.1726
D1ANN6	60	2770.9353
D1APC8	81	2626.6724
D1APK8	100	2649.9084
D1AQC4	91	2909.3071
D1AWL3	83	2594.502
D1BC10	75	2752.2056

D1BCW8	62	3710.9126
D1BWB0	37	3948.2148
D1BX60	62	2937.7612
D1C7U8	22	3003.3923
D1CGH4	18	2671.4004
D2AUF2	34	2295.8953
D2B261	9	5547.2891
D2BAN2	48	4050.637
D2BAN4	31	2416.2512
D2BDF5	59	2436.4768
D2BKB9	7	2680.8782
D2BKE1	57	2570.4846
D2C6W2	67	2223.0017
D2EH44	26	2874.5518
D2PL17	29	2352.3154
D2PL35	7	3971.1606
D2PR30	84	2826.5776
D2Q1I7	27	2368.2485
D2Q763	99	3599.2788
D2QBR1	7	2597.6794
D3AAZ8	94	2797.7842
D3ADZ0	66	3705.0398
D3AL50	31	2641.8604
D3F6P8	87	3771.9102
D3FS09	62	2232.8591
D3HB41	50	2832.4146
D3P3S6	96	2621.5088
D3P5V9	22	2349.5073
D3P757	47	2564.385
D3P779	40	2501.093
D3PLV5	24	2433.6392
D3PQW3	61	2809.9683
D3Q2J6	42	2414.552
D3Q414	4	2304.7375
D3R7U0	79	3818.1367
D3RPI3	83	2876.8735
D3T6M2	32	2177.2725
D3Y2V4	93	2146.5203
D4DZX8	94	2571.2515
D4E282	89	2721.4265
D4F1U7	9	2700.594
D4HVI8	44	2752.0481
D4IYQ1	76	2424.2031
D4JEZ1	84	2662.8511
D4JG79	97	2952.804
D4K1K7	37	3008.0688
D4KAE7	1	3197.2351
D4KCX2	99	2653.6074
D4LC32	63	2749.5601
D4LKX5	78	2738.2888

D4LQI8	26	2843.9453
D4MVD3	34	2816.9407
D4MZ70	76	2813.0432
D4YI50	37	1649.1952
D4ZIJ5	61	2509.2842
D5AU75	45	2734.6726
D5BF76	72	3079.717
D5DZI8	28	1940.4937
D5E2L2	16	3157.0786
D5HDE6	48	2813.0662
D5HDJ9	65	2995.9436
D5KQK3	4	2558.6394
D5KX75	7	2871.0867
D5ULE7	82	4862.0845
D5VFQ0	7	2463.6685
D5WGC6	55	2417.6658
D5WLR1	84	2728.7419
D6A0U2	1	3833.103
D6A2J4	53	2274.6123
D6A3T6	27	4253.3921
D6A5X6	96	2292.0337
D6ABL3	21	3651.804
D6ABM2	32	2737.7932
D6AEP8	81	2370.2588
D6AI79	75	2195.084
D6AQT5	61	4811.459
D6B2A8	83	2402.741
D6ETZ7	34	3202.0361
D6EU92	1	4180.0459
D6EXV5	21	4183.9302
D6K1J5	72	2308.1272
D6K754	92	2322.4788
D6KER2	74	2213.8025
D6TBS0	41	2792.0862
D6U006	3	2767.6738
D6U0N6	13	2337.876
D6X9X8	33	2339.0457
D6Y5B2	75	5798.1211
D6Y646	28	4262.8291
D6Y6J1	98	4441.5376
D6YBP6	16	2389.6838
D6YBP8	86	2438.5044
D7A385	65	2925.7903
D7AS23	84	2092.9233
D7AXP9	98	3549.9255
D7AZG0	76	4561.0938
D7B2H4	45	2517.4277
D7C0L6	88	2563.8237
D7C1E5	80	5062.0635
D7C5W5	14	2301.6643

D7CRB8	40	3127.6572
D7MI42	28	2578.606
D7RTU9	3	2574.6467
D7UV05	47	3248.1729
D7UV53	67	2930.3342
D7UW26	91	2568.8994
D7UW38	40	2917.9463
D7UWE1	4	2891.1299
D7UWY0	33	2555.0242
D7UXB4	11	2818.5864
D7VDX2	69	3131.7773
D7VE52	13	2473.5435
D7VPW1	74	2551.4263
D8FNI9	59	2826.9009
D8LGS1	35	5520.6104
D8LQW6	10	4509.1064
D8S991	54	4411.397
D8UQ06	62	2497.5403
D9R0D8	85	2855.7139
D9SVH0	1	2055.7173
D9SZB9	4	3897.2646
D9SZT8	52	4921.8022
D9T3K2	88	2450.9187
D9T8D4	49	2394.1631
D9TCC7	45	4058.9216
D9TIB4	17	2385.8228
D9TR57	64	2093.0522
D9UP56	15	4044.355
D9UYK1	95	4742.3525
D9VHM8	6	2473.7437
D9WNM9	85	2261.895
D9X810	61	3349.1528
D9XB25	10	3985.4851
D9XHQ6	63	3341.2275
D9XPF0	69	4136.1885
D9XZ76	85	3639.616
D9Y101	88	2235.2839
D9Y1S6	15	4162.4268
E0MSZ2	41	2414.146
E0NHM3	28	2794.5369
E0PDF8	66	2969.6299
E0PFZ9	60	3842.6199
E0Q968	20	3593.0784
E0RA06	37	2235.0684
E0RB30	70	2707.3948
E0RCR1	26	2866.1802
E0RE68	4	2123.0046
E0RJV2	48	3108.7878
E0RL55	84	2763.1533
E0RQX8	55	2500.9075

E0RXJ5	23	2443.4299
E0SEG5	57	2657.71
E0SEG6	21	2675.9453
E0SEH6	69	2726.085
E0X9H4	61	2043.3827
E1IGK6	15	2188.9075
E1LMW8	24	2823.2341
E1LQA9	41	2648.9856
E1UKU7	71	2576.2825
E1UTL1	13	2824.0562
E1UTQ7	85	2712.8486
E1VSX4	88	2180.7002
E2NPA7	54	2691.127
E2PVV7	16	3565.9094
E3CIV2	38	2724.8325
E3E9Z4	22	2228.7427
E3EAC3	21	2136.428
E3F031	9	2330.2625
E3FN28	80	2416.3926
E3FSZ5	35	2575.6338
E3H2Z7	23	2629.2332
E3IUS0	36	3186.1233
E3UMV6	26	2707.0994
E4LNR8	22	2746.3027
E4LRI3	67	3337.0498
E4LT50	27	3230.2803
E4LW40	31	4008.9695
E4LW77	88	3631.3965
E4LXN4	77	2994.6387
E4LYB4	45	2609.3933
E4LYQ9	30	3510.8538
E4LZ66	10	3002.137
E4MZV6	22	2149.3125
E4N3R8	7	4709.8081
E4N5Z2	75	2487.0894
E4NBD1	68	4727.7515
E4Q361	29	2434.5894
E4Q7Z7	18	2389.0571
E4S6B1	73	2360.4109
E4SGY9	3	2368.9607
E4WGS6	50	2778.752
E5B1F9	41	2747.4126
E5YL48	4	2678.8777
E6K1F8	27	2402.9094
E6NU37	37	2418.6326
E6RMI2	11	2436.5864
E6S7C2	49	2603.5708
E6TUY6	4	2289.2527
E6U3G5	13	2000.1248
E6VGW0	28	2867.2104

E6XES3	16	2595.5649
E7FHY4	42	2180.7761
E7FMU7	85	2742.0952
E7FS70	6	2915.7742
E7RC93	22	2183.4812
E8JUK5	39	3084.8311
E8LUA8	71	2774.677
E8M8E2	49	2487.6343
E8N3Y4	35	2540.3257
E8N6X0	7	2435.262
E8U905	78	2773.5684
E8UQS3	76	2098.6162
E8URA9	29	2097.7722
E8UXX5	74	3335.4873
E8VZV1	55	2769.8435
E8W143	6	4351.0425
E8W810	33	2317.0457
E8W9K4	35	2277.1326
E8X6A1	26	3102.5862
E9SVV9	58	2842.4749
E9UUK0	14	2588.7153
E9UXK4	67	2965.5488
F0GXD3	31	3110.7258
F0LAM7	33	2412.5759
F0M5E8	84	2529.2087
F0MA10	61	3051.259
F0SBK6	23	2583.0613
F0YZT2	5	2810.3579
F0Z422	22	3247.6301
F1JZ12	77	2201.7629
F1LL52	83	2248.521
F1TE14	89	2718.0439
F1YKW9	23	4551.0825
F1YXP4	15	3139.9124
F1YZM4	81	2939.4807
F1YZM7	66	4478.3306
F1Z0S2	83	2664.8909
F1ZUP6	78	2110.2803
F2AFP4	99	2398.3032
F2G9T0	96	2642.9204
F2JPA6	53	3355.0149
F2JX03	19	2207.2229
F2N7E4	64	2640.854
F2NA43	28	3007.9985
F2R8J7	10	2494.4868
F2R8K2	55	3162.0769
F2RGF6	74	2352.6785
F2RKF5	79	3554.0325
F2RLS6	30	2529.4517
F3BJ64	98	2661.8411

F3BMW9	61	2393.7322
F3NAM3	68	2832.6304
F3NEX3	48	2253.0012
F3NIF5	72	2367.7773
F3NIG0	43	4633.6655
F3Y9S9	91	2609.8269
F3YCS4	32	2706.7297
F3ZH12	48	2212.6909
F3ZIC4	98	4104.814
F4AIE0	27	2771.7458
F4BL34	39	2809.8103
F4BLB9	58	2903.804
F4C2M4	84	2486.8857
F4F3X7	34	2434.7034
F4FD86	100	2392.812
F4FEA7	99	4250.2114
F4FEZ0	1	5162.9209
F4FFK1	18	3879.7412
F4GH96	8	3854.9756
F4GLK2	14	2543.8022
F4H7E6	14	4665.7993
F4KVL5	7	2833.822
F4LRK3	27	2667.4602
F4MXF8	42	2671.887
F4MXN9	81	3527.1033
F4N5B9	61	2614.6211
F4NJN0	14	3421.7461
F4NPV6	90	2714.615
F4QIE2	50	2603.2622
F4QNW2	11	2418.1528
F4QNW3	89	2420.2888
F4QTA0	95	2250.1311
F4XR96	68	2569.8926
F5JK61	96	2421.4846
F5U4N6	19	2800.1567
F5WYI5	54	2430.1663
F5X2P1	27	2365.2625
F5X4E2	70	2455.8411
F5XGJ5	74	2690.791
F5XLS1	72	2713.4673
F5Y9D3	36	2415.6687
F5YLG7	50	2714.5176
F5YNN0	32	2685.8611
F5YNT1	74	2680.698
F5YNT7	31	2695.5676
F5Z6I3	74	2763.3435
F5ZGD5	68	2829.8203
F6BL86	69	2061.166
F6CUI3	86	2165.7629
F6DJP6	76	2092.5115

F6FR56	83	3716.9661
F6FS57	69	2789.4531
F6H7V7	62	3002.4731
F6IDL9	97	2888.5247
F7K4L0	100	2321.5908
F7KF50	22	3259.9253
F7NRC1	63	2624.0706
F7RR92	78	2274.1614
F7UBG1	53	2514.7065
F7X9K8	42	2372.8125
F7ZA00	14	2685.907
F8A202	16	4261.437
F8A798	64	2976.4856
F8CBN7	52	2608.3298
F8F2L0	3	2652.8394
F8F615	77	2242.7085
F8F6C7	99	1812.8186
F8I8K7	54	2707.637
F8IE65	10	2483.6531
F8JXM9	76	2656.0176
F8K1W6	96	4117.4292
F8L746	32	3123.2886
F9EGJ8	4	2649.76
F9HIS5	1	3437.6321
F9HL45	21	3011.0405
F9LWP8	69	3470.1064
F9P3I4	50	3861.4763
F9TCL2	97	2422.6235
F9U6X5	43	2658.5105
F9U737	51	2263.5322
F9ULH8	41	2425.4954
F9XYD9	62	2580.0598
F9Y846	9	2330.2625
G0AX44	53	2396.9246
G0FQQ5	95	2703.2634
G0FRP6	54	2484.6338
G0FZ95	19	2670.8999
G0G5T9	41	2924.4722
G0GFK4	7	2418.7227
G0L4C6	84	2807.0784
G0M2J6	41	2527.5073
G0PQW9	72	3157.886
G0PUU1	85	2820.4238
G0PVZ2	90	4017.5764
G0Q2R3	49	2260.2112
G0Q381	32	3660.7434
G0Q482	12	4482.6138
G0Q8E5	4	2392.7791
G0V3V5	72	2078.407
G1C1T5	45	2555.2234

G1Y4C7	98	2245.3606
G2DX76	9	2431.5012
G2G8K4	8	3813.981
G2GDH5	60	4233.6304
G2GLL5	74	2288.0266
G2GLZ6	37	3132.3599
G2LE13	33	3600.3748
G2MRY3	27	2120.2168
G2MUZ4	50	2105.7107
G2NB62	84	2191.0718
G2NE89	89	2380.5757
G2NFR6	77	2403.3718
G2NJG8	35	4429.9336
G2NPU1	93	3215.03
G2PEP9	53	2301.1853
G2PWX8	73	2360.4109
G2RNI7	26	2438.3315
G3EGK5	70	2371.613
G3LY19	78	2752.9116
G4F057	49	2673.0198
G4HKQ4	84	2048.7175
G4NQU7	6	2661.5972
G4NR65	37	2744.9863
G4NYI0	25	2746.8364
G4NYJ9	89	2625.8792
G4RAB5	68	3103.2258
G4XU74	28	2158.0464
G5I9K8	7	2795.979
G5IC88	29	2685.3782
G5IHC4	67	2864.9758
G5IMS5	9	2248.2041
G5JTB7	6	3562.27
G5RMN3	62	607.2469
G5RMN4	2	1234.678
G5ZW36	70	2649.2166
G6F9T1	78	2618.7705
G6FAV8	58	2572.1506
G6FBI5	98	2719.075
G6FFS4	13	2795.3979
G7D1R8	94	2436.2937
G7DLD6	91	2584.4609
G7EF52	38	2409.3782
G7EHU0	8	2519.4973
G7ET08	36	2631.2834
G7F3X3	50	2359.9514
G7F6V7	23	2652.031
G7G5P6	78	2846.0344
G7G6E9	29	2540.6489
G7M6D5	66	2477.0295
G7VTE8	26	2267.54

G7W3A7	79	2121.6533
G7ZEM2	8	2370.9373
G7ZH49	13	2410.3296
G7ZIC3	96	2459.27
G7ZID5	18	2586.2717
G8ARQ1	25	2570.7852
G8AWC9	35	2410.4688
G8AWD9	83	2647.9758
G8AWE1	50	2441.8049
G8NVF9	1	2212.5964
G8NWT0	93	2938.0754
G8P195	16	2696.7114
G8P4C7	79	2496.425
G8P4F6	40	2809.6653
G8P915	51	2559.6069
G8QYI0	24	2401.9841
G8S7I1	57	4342.4341
G8S7Q9	74	4095.3005
G8SB50	26	6260.0229
G8SC15	71	4737.1294
G8TSK9	3	2902.3792
G8TT01	54	2707.637
G9A0Q1	86	2262.4019
G9BYB8	86	2373.8289
G9IS31	50	2668.6707
G9IS39	8	2609.697
G9RQQ0	36	2623.7554
H0BCI1	38	2172.4824
H0BDP8	3	2409.3521
H0BE14	94	3578.887
H0BIK3	53	2560.6594
H0BLZ7	54	2949.3782
H0FZK7	20	2347.835
H0HC94	76	2388.5969
H0K1S0	67	3360.9536
H0QPX1	68	2574.8533
H0S301	38	2851.6465
H0S7F0	21	3547.5874
H0S8U3	95	2507.6482
H0SMM9	69	2816.6292
H0T1Z3	75	2934.168
H0T5R6	52	2644.9902
H0TI04	12	2801.4219
H0TP06	51	2693.8328
H1Q7A1	28	4145.895
H1QA68	15	2277.2659
H1QQV6	53	2958.5464
H1QRE5	27	4180.9136
H1X6D4	95	2809.2065
H1Y3B9	69	2652.1182

H2CFD7	19	2377.3833
H2JI37	68	2676.6394
H2JLX3	33	3926.7344
H2JLX8	1	4194.8896
H2JP17	68	2349.7812
H2JXT2	73	2545.6128
H2K5D1	56	2813.9421
H3MMQ0	98	2619.9695
H3MW96	64	2608.584
H3R9U2	31	2689.5828
H3RE30	55	2787.1282
H3RIM2	34	3500.8354
H3ZEG5	33	2549.8867
H4FDK8	21	2419.9099
H5T5C4	98	2634.1355
H5T6U3	86	2700.6519
H5WVY6	21	2469.6514
H5XAW9	23	2668.8896
H5XJU1	1	3873.2332
H5YQE7	47	2546.3538
H6CD97	74	2721.9351
H6CNP0	14	2794.322
H6CP27	71	2117.2793
H6CRB9	55	2698.1895
H6N9L4	11	2277.3936
H6NMQ2	29	2249.7683
H6PBK3	72	2897.8821
H6SRJ1	5	2403.562
H7GEV0	27	2088.6626
H8E6T3	35	2778.6541
H8FXJ8	37	2594.4275
H8GDM6	1	3353.3313
H8H0B5	87	2605.0383
H8MU62	30	2502.4053
H8XVY6	65	2444.1597
H9CGR5	34	2542.4851
H9U9K9	5	2628.3511
H9UL14	13	4590.5039
H9ZGD8	90	2956.0796
H9ZGD9	68	2952.9502
H9ZGE0	67	2840.4436
H9ZGE1	95	2818.5769
H9ZGE2	4	2913.7134
H9ZUT0	45	2105.9158
I0BCR1	91	1865.5223
I0BJW8	61	2235.0896
I0G3J4	65	2588.6394
I0GV58	20	2644.5405
I0HF78	8	3570.0708
I0HIP5	60	4255.8911

I0HYI5	85	2114.3311
I0IGW5	22	2721.6665
I0JQY5	10	2216.4407
I0K7N3	95	3016.707
I0KZ53	14	4221.0312
I0L5G8	17	2096.6958
I0L7W2	20	2265.9624
I0L805	87	4403.6514
I0L9J4	67	2572.9119
I0LCR6	56	3792.23
I0R7E1	5	3127.6824
I0UW37	76	2661.8352
I0V7N4	98	3400.0908
I0YK64	22	2402.3179
I1AVY5	39	2555.0178
I1D3B4	6	3119.9194
I1E031	18	2443.6433
I1HU83	69	2552.1267
I1NUA2	18	2421.5591
I2C178	26	2708.197
I2CBS4	65	2909.0823
I2F6Y5	83	2404.6208
I2GLH4	63	2478.325
I2IRY4	83	2597.3494
I2QQE8	56	2540.2898
I2RVU7	16	2604.1257
I2SQZ8	90	2714.615
I2UCB9	89	2725.5481
I2W777	49	3035.5161
I2X071	19	2767.5786
I2X5G8	62	2763.9365
I3AIN0	65	2636.9917
I3QIG4	44	2613.9546
I3SYX2	90	2472.1663
I3U0Z7	65	2731.5396
I3VS74	78	2062.9062
I3VXG7	10	2074.3691
I3XDD0	97	2278.2229
I3Y5S0	69	2191.957
I3YH25	74	2494.5183
I4B8U7	11	2200.8682
I4ERL5	78	2848.7012
I4VUG3	8	2318.7222
I4VWU8	31	2701.2285
I5BYR6	75	2318.3
I5CAT0	21	2484.5188
I6APM1	13	2422.4182
I6APS1	60	2317.3276
I6ARU5	18	2481.4783
I6AYS5	28	2510.5164

I6ZYM4	58	2574.6262
I7CL63	14	4184.4941
I7CPF4	100	4600.6919
I7DLX2	52	2342.6284
I7EAL6	28	2326.2222
I7EUV9	18	2431.8127
I7IVF0	11	2713.0522
I7J5U3	54	2148.4905
I7KHU7	56	1670.2749
I7KI36	42	1993.8092
I7LD22	33	2786.5564
I7LPV0	48	588.4423
I7LS21	16	2597.7759
I8R1J8	30	2072.7378
I8TQ36	84	2418.6111
I9DRN0	6	2849.3789
I9KXA9	62	2732.8892
I9NFG2	59	2353.825
J0BEL1	4	2389.4587
J0KUK3	95	2327.0066
J0N0T0	20	2952.2732
J0NGX7	77	2784.6245
J0P229	62	2655.9656
J0V2T6	10	2450.8962
J0VWU7	26	2383.9465
J1ACA0	30	2476.042
J1HVE4	44	2695.1426
J1HX71	29	2676.5581
J1NZZ1	95	2993.2463
J1TBL4	90	2341.8059
J2H761	33	3323.6074
J2JX81	30	3092.5559
J2KWY4	1	2369.0042
J2LTU4	47	2662.7588
J2R701	53	2384.8938
J2VR00	96	2605.8801
J2WDV1	35	2715.0312
J2ZSL2	28	3083.6443
J3ANZ0	9	2609.0854
J3C6P0	65	2343.5596
J3L6Q8	52	2599.4553
J4XBG7	65	2549.228
J4XRR1	83	2640.0857
J5HAL3	14	3075.3679
J5MQG5	16	2375.8762
J5U9U2	37	2602.5576
J5UTN3	16	2365.0713
J7IJ95	8	2215.4536
J7KFN3	13	2373.6279
J7L8F2	93	2810.1416

J7LBN1	83	4396.6108
J7LI95	95	2336.74
J7LN09	48	2199.7769
J7LYT0	6	4237.2134
J8RCF0	5	2528.8452
J9A1G5	6	2607.1609
J9HP47	6	2368.5383
J9XU85	16	3695.0566
J9Z024	15	3133.769
J9ZZW8	23	2717.6755
K0A8J9	50	2030.2019
K0D3E2	91	2398.3931
K0D5L8	1	2666.9575
K0EER5	78	2718.6519
K0JRE9	61	5993.9126
K0JWV1	32	3803.3777
K0K6D9	49	3243.303
K0KB51	31	2478.6069
K0PLM8	72	2392.1775
K0YU62	16	2720.1614
K1UFA6	54	3946.0203
K1UXI9	18	2527.5984
K2GR21	87	2480.2612
K2IR40	74	2449.093
K2LMK5	89	2722.8494
K2M7D3	86	2572.4893
K2N268	70	2477.313
K2PNL2	60	2949.8821
K2PXX1	88	3656.4463
K2Q1B7	84	2571.627
K2QB67	58	2838.696
K2QBJ7	66	2861.877
K2QF48	87	2684.5813
K3XH76	100	2312.8584
K4D5D2	31	2501.666
K4I4U1	67	2370.1824
K4K173	85	2520.1987
K4KIJ3	82	2367.5774
K4QAX1	29	2420.8667
K4QVT5	62	2311.1868
K4R1I7	26	2989.1138
K4R9P9	40	2327.7112
K5CYK6	29	2596.4468
K5TC37	89	2582.7151
K5USK6	4	2624.3606
K5VIP2	89	2582.7151
K6PJ73	77	2538.9749
K6QJK5	54	2574.9041
K6QPI5	78	2742.2651
K6QWU0	48	2748.7568

K6R4L1	41	2726.3918
K6RDR9	59	2560.4331
K6RPI9	28	2780.5371
K6RUM4	87	2525.959
K6TJ93	14	2447.2983
K6VWJ1	4	2392.199
K6WUZ5	3	2804.8882
K6YVQ1	63	2792.9419
K6ZHI5	68	2477.9509
K7AIP5	54	2863.7749
K7PES2	65	2440.3232
K7QWH8	92	4545.0146
K7R8A6	22	2047.1837
K7S4S9	43	2488.8982
K7VST0	4	2540.3694
K7W2F0	55	2355.3474
K7ZX81	88	2820.7278
K8A1Z8	5	2762.5671
K8A4I0	80	3071.1628
K8ADK6	68	2933.8782
K8AJR8	32	2810.8816
K8ALA8	49	1353.1639
K8ASY6	47	1880.0646
K8AW43	26	2629.2571
K8AZ31	85	2723.5757
K8B0Z4	28	2768.5559
K8B4Z9	65	2921.6179
K8B7R2	28	2812.6458
K8BAR2	35	2801.3638
K8BI51	71	2008.5338
K8BQP1	27	2838.7366
K8BWT5	12	2751.5845
K8BYF6	93	1180.0078
K8C0I1	8	2818.3821
K8C261	9	2727.6809
K8C913	59	2703.5139
K8CBQ1	19	2810.9182
K8CMR9	22	1372.436
K8CT21	66	2765.645
K8CTC7	82	2752.4167
K8CVG7	81	2783.8953
K8D7Z9	69	2803.4138
K8E1P0	19	2606.7214
K8GQK7	9	2705.1709
K8QBV8	53	2595.5266
K8QQB4	70	2736.2544
K9CYL1	66	2666.4175
K9DZC8	79	2366.6111
K9E351	55	2671.0745
K9TDJ7	65	2561.9243

K9WM42	99	2694.2153
K9WQ83	80	2489.4189
K9XAN6	63	2272.9497
K9XKL8	69	2687.5183
L0A0G1	60	2525.0374
L0DCM3	86	2654.8398
L0EBS9	38	2103.9434
L0EHU9	32	2255.5974
L0EK01	43	2407.0408
L0EKA4	5	2352.4011
L0ELF5	37	4305.4292
L0IIV7	73	2080.1484
L0K7D4	19	2495.5022
L0LQR9	8	2578.865
L0M412	20	2360.6426
L0NI99	36	2406.4902
L1KDW9	54	2297.1499
L1L4D1	88	2915.6753
L1L7Z9	21	4253.8696
L5N1X7	47	2151.2844
L7EYW0	69	3614.6511
L7F4B6	32	3023.5713
L7F5W9	34	2750.9583
L7FBS5	1	2817.7468
L7FDA7	29	2467.0696
L7FHS6	87	4823.0601
L7LMB8	70	3109.387
L7RVW5	5	2422.7136
L7T8J0	14	2420.4766
L8DCH1	10	2511.3838
L8ENY0	78	4253.603
L8EVL9	14	2614.3367
L8F638	36	4642.2354
L8J8Z6	74	2511.2664
L8K5M4	88	2649.8105
L8P7A3	21	3912.9238
L8PCP8	30	2938.2087
L8TQ75	96	2900.4946
L8XKA1	63	2347.8022
L9P9G7	99	2733.9631
L9PE61	14	2410.1931
L9PHR2	84	2953.0542
M0Q9T2	9	2700.594
M1BC60	68	2540.5605
M1MT62	61	2613.1074
M1NQJ6	23	2488.8848
M1QQC9	76	2625.7222
M1Z429	28	2219.8755
M1ZM06	35	5201.3262
M2NN97	76	2471.457

M2NRX8	55	2832.7083
M2PZ93	76	2981.7769
M2Q4V4	46	3759.3101
M2V479	66	2429.6379
M2X794	2	2786.7312
M2XRQ3	94	5425.7549
M2YAZ1	35	2503.6433
M2YJT2	90	2347.7
M2YLB3	93	2022.4824
M2ZB83	69	2637.5657
M2ZG92	86	2953.2686
M3BYH3	29	3622.2366
M3BZA6	3	4261.0283
M3D101	97	2257.4307
M3D2W6	47	4232.9834
M3D3N7	47	3230.4468
M3DHJ5	60	4170.832
M3ED66	18	4093.0967
M3EIG2	11	1817.9996
M3EK39	44	4238.0796
M3FUY6	23	2839.417
M3VGR2	62	4958.9731
M4F2P8	22	2489.3945
M4KUV0	64	2588.1985
M4KVI0	83	2723.501
M4KYJ7	96	2605.4177
M4MZB5	42	2372.8125
M4NMY0	33	2477.1296
M4YXZ5	93	2400.5918
M4YYI1	88	2700.406
M4Z8X1	42	2692.5278
M4ZDN8	3	3255.9131
M5A7J9	12	2565.6658
M5B8Z3	55	2671.1655
M5BYZ4	57	1451.744
M5C0E3	57	1397.1877
M5C1D8	71	2717.2986
M5C6H3	19	4087.5483
M5DXZ5	39	2541.5984
M5QUM2	85	2816.9827
M7MR34	82	2845.0186
M7N622	50	2253.1384
M7R6A1	52	2622.6843
M8AF35	69	2352.7986
M8CQP7	13	2660.4678
M8CQU4	93	2877.875
M8CYT6	76	2135.6453
M9R842	55	2779.749
M9RMG1	57	2798.3936
M9TW44	67	2347.5007

M9TXM8	54	4330.9341
M9U0R4	55	2769.8435
M9U478	79	2303.4451
M9ZC55	2	2715.0715
N0CP03	63	2356.1372
N0CZM8	22	3799.0571
N0D4R4	88	2378.4705
N0D4U4	65	2981.0918
N0D629	52	2207.5974
N0DY37	84	4006.1069
N1JQ25	98	2417.7781
N1JSG8	24	2933.9209
N1UX26	78	2428.3
N1V5H2	13	3033.4705
N6V0Q4	15	2404.6953
N6W1U8	93	2678.0686
N9VN24	99	2651.3904
O08324	71	2349.4934
O24524	18	2766.1194
O61594	72	2739.2717
Q07HP5	20	2734.614
Q084Z6	95	2694.3911
Q08IT7	43	2715.5281
Q08S20	61	2358.6953
Q08YK7	10	2411.5576
Q091M8	58	2789.5928
Q0FHD6	33	2345.5127
Q0GMU3	16	2144.6421
Q0R5R7	98	2764.8779
Q0SHX5	49	3550.1836
Q11NH0	27	2497.4976
Q12601	27	3220.4529
Q12KZ0	16	2918.1125
Q13A13	83	2788.1584
Q13PT6	72	2257.3521
Q14QP8	27	2754.5833
Q167E6	30	2638.8257
Q1AUD7	51	3912.8267
Q1CY46	44	2581.4634
Q1CYU5	32	2481.0759
Q1GM35	90	2385.115
Q1GUD6	47	2290.6768
Q1J2J3	96	2782.8223
Q1J5L4	87	2450.4363
Q1J655	92	2777.1907
Q1JFR5	58	2493.3872
Q1JGD8	92	2777.9158
Q1JKS5	59	2487.019
Q1JLA4	74	2788.3677
Q1MBM6	81	2431.9819

Q1R4L5	59	2775.0027
Q1R7Y4	36	2844.0718
Q1Z7Q5	96	2765.5647
Q21EM1	83	3006
Q21KX3	54	2660.1975
Q21ZF1	66	2178.1255
Q21ZG0	88	2537.8853
Q28R01	43	2585.2131
Q2GA89	64	2480.4102
Q2GAY4	83	2492.3892
Q2ITY8	16	2803.9626
Q2K441	13	2388.1777
Q2S749	76	2595.6548
Q2UL94	84	2123.2383
Q2WAV2	53	2283.3599
Q2WGB4	94	2162.4348
Q38WG1	77	2838.5671
Q3J2E9	88	2303.0808
Q3XXN2	53	2730.4587
Q3Y0A1	35	2680.9197
Q3Y0A9	45	2717.5278
Q3Y0M8	49	4213.8203
Q42618	93	2823.2998
Q43014	8	2777.2153
Q45FX7	2	1531.1562
Q47PF5	47	4724.5596
Q47RE2	26	2508.0691
Q47XU7	33	2835.8877
Q48S46	78	2421.1841
Q48SY1	74	2788.3677
Q4WBS7	99	3326.4375
Q51723	42	2180.7761
Q53EH2	1	2055.7173
Q53W75	89	2093.4204
Q53WW9	11	2948.4392
Q59976	39	4043.9121
Q5FJD3	9	2807.1831
Q5KUY7	24	2814.9199
Q5KXG4	46	2482.8445
Q5UB04	42	3256.47
Q5WBD7	66	3357.6824
Q5WKT0	38	2700.908
Q5WL98	58	2861.8533
Q5XAU7	10	2278.7825
Q5XBM4	74	2788.3677
Q60026	38	2085.4885
Q608B9	5	2478.2478
Q63F48	73	2707.4697
Q6AKE8	84	3061.457
Q6CYW8	19	3094.6697

Q6MSD6	40	2399.5449
Q6N915	41	2542.4861
Q6QGY5	78	2601.5549
Q700B1	94	2752.928
Q746L1	50	2114.8711
Q7CV27	7	2432.1204
Q7MG41	38	2522.5049
Q7NYK2	33	2746.9575
Q7X3Y0	34	2429.5615
Q7Y073	77	2462.2205
Q82BV6	64	2964.4324
Q82CT5	14	3898.5369
Q82M59	44	2271.2615
Q86D78	87	2667.3145
Q870B6	61	4213.2329
Q89H18	98	2937.6123
Q89L91	97	2474.8657
Q89UP6	48	2440.9216
Q8CJM3	12	2446.8979
Q8DNA9	33	2737.9585
Q8DRA9	4	2830.717
Q8EMS8	58	2720.8398
Q8ES64	27	2780.6448
Q8GEB0	17	2096.2292
Q8GEB1	43	2136.6375
Q8GEB2	94	2078.1487
Q8GEB3	89	2093.4204
Q8GEB4	17	2096.2292
Q8GHE5	37	2096.2388
Q8GKQ8	53	2578.9971
Q8RCQ8	21	2085.8708
Q8T0W7	60	2516.7881
Q8X214	43	2540.938
Q92LX2	42	2372.8125
Q93X78	24	6058.9087
Q97M15	36	2466.4146
Q99Z97	71	2734.4502
Q9A6F8	28	4353.5747
Q9C122	40	5134.0396
Q9CFI7	7	2577.8315
Q9CFL0	49	2768.4556
Q9CJ31	94	2577.8574
Q9EXF8	12	2572.3713
Q9F3B7	58	4133.6777
Q9F3I3	21	4183.9302
Q9GSE6	5	2758.6992
Q9HIL6	29	3440.5295
Q9K440	14	3213.0505
Q9KBK3	5	2159.3984
Q9L2L6	3	2993.73

Q9L794	34	2127.6272
Q9LAV5	26	2508.0691
Q9RA58	93	2080.407
Q9RA61	50	2114.8711
Q9SPK3	64	3299.1436
Q9UUQ3	33	5080.8496
Q9X9R4	94	1112.3882
Q9ZNN7	92	2050.1206
R0A635	83	2328.5332
R0E6N0	6	4431.21
R0FFY1	66	2399.2354
R0GZG2	88	2384.0881
R1CUK8	88	2097.3843
R1FKF0	53	2872.1953
R1FT37	37	4027.5835
R1GAE9	27	2415.9185
R1GBW0	6	2671.2559
R1I2E4	72	2743.7522
R1IFP3	3	2840.7107
R4G036	67	2754.1804
R4LKY4	92	4522.9785
R4LMQ4	53	4072.5757
R4LWC4	80	4200.3618
R4PXL6	30	2896.9771
R4SR38	50	2989.0557
R4SWC3	88	2127.6843
R4T6P4	82	2305.7849
R4TBK3	75	2594.4258
R4THI3	19	3794.3513
R4YH72	85	2624.3889
R4YWU4	6	2662.9807
R5DYS0	91	2893.9236
R5EQ88	48	2728.7415
R5JYX8	44	4730.6108
R5N4U6	40	2760.4241
R5SYI5	60	2644.9487
R5TRK9	38	2656.6543
R5TWR6	9	2190.6941
R5V1N6	65	2817.2344
R5WJG7	31	2654.9241
R5ZQZ5	48	2669.5801
R6NN85	25	3229.2688
R6PXX2	69	2981.7173
R6VC17	94	2949.2488
R6ZYM6	91	2779.1699
R7BJP0	50	2582.3235
R7D3D1	71	2971.2903
R7DU43	19	3125.2322
R7H2Z3	86	3078.8767
R7I469	80	3156.6731

R7RUN9	18	2124.7554
R7XT60	53	2499.6909
R8HJ90	5	2528.8452
R8KT44	84	2488.7146
R8Q5X0	72	2615.1345
R8TNK1	84	2488.7146
R8V3Y2	35	2638.655
R9CE34	69	2490.7207
R9G8Q2	89	2915.5498
R9GHS9	100	2799.8503
R9GWA4	99	2625.0762
R9JK27	72	2745.8982
R9PFT7	38	4211.2227
R9U0C2	35	2686.2788
R9U0D6	77	2613.9128
S0AM59	90	2599.0491
S0FIW2	3	2282.0198
S0FYL9	69	2104.3159
S2LVQ2	92	2526.0681
S2NFY0	78	2742.2651
S2P6U4	70	2523.7698
S2VME4	60	3202.8208
S2YBY3	86	4054.5757
S2YRJ9	6	4136.752
S2YRW1	87	3608.542
S2YZB7	38	2387.6865
S3AKQ3	95	2448.1895
S3AU36	34	2349.2917
S3B776	35	2778.6541
S3C2X1	89	5244.4575
S3FCI8	89	2021.0663
S3FEZ0	46	2315.3149
S3H9L5	4	2346.4973
S3Z732	18	2779.8621
S4A001	27	5121.4092
S4MGR2	21	3472.3267
S4MYQ7	62	4149.5879
S4N9R4	11	3900.2419
S4XIQ3	54	2840.1848
S4XJZ7	31	2651.7288
S4XL86	61	2340.6587
S4YMW4	52	2316.478
S5AK99	96	2642.9204
S5ASB4	69	2494.3643
S5C7J4	87	2671.981
S5S0W9	92	2409.9861
S5U834	30	2734.8472
S5UVC9	35	4112.0605
S5V2I9	84	2859.8845
S5VXZ2	15	2272.6169

S6EUE8	65	2749.2322
S6EVI6	22	2805.9868
S6EVS8	1	1383.947
S6F9A3	63	963.1382
S6FR19	14	2585.1101
S6GNS4	99	2589.4468
S7U581	74	2710.0178
S7XIH6	3	4371.7871
S9QAM8	94	2080.5647
S9QDH2	23	2597.4519
S9RDB0	80	2523.7927
S9RLG1	95	2400.2007
S9SHG7	14	2568.1355
T0BPF3	100	2280.4753
T0HTG3	90	2574.5698
T0IT97	68	2673.562
T0K8N3	22	2622.54
T0PWK0	59	2667.4561
T0TF90	63	2936.0334
T0TSU1	73	3001.6426
T0U0H1	83	2135.5078
T0U295	50	2216.0422
T0U8Z8	97	1944.7198
T0UE82	95	2661.6062
T0UEH1	63	706.0971
T0ULY1	18	1790.8433
T0VJW6	9	3908.8286
T0VMS9	22	2819.2324
T0VQA6	37	2813.6355
T1NU52	64	2459.1592
T1VQ26	75	2602.1558
T1ZGJ3	36	3058.1404
T2KKC3	26	2362.5059
T2RZY0	30	2368.7192
T2S6V3	26	2543.9138
T5I764	90	2599.2363
T5KMS4	40	2564.3237
U0FGF9	18	2590.3914
U1HA43	69	2514.009
U1J1N0	54	2310.1199
U1J6A7	19	2674.2974
U1JXP4	50	2395.979
U1JYQ2	12	2709.9363
U1K5V5	23	2652.031
U1L837	48	2512.8689
U1LD59	76	2083.3318
U1M199	2	2333.533
U1M1A3	48	2020.7882
U1RC45	4	3937.1921
U1Y804	21	2660.2432

U2A5Y3	31	3835.0481
U2CBP5	58	2734.3496
U2EIJ2	37	2807.9446
U2H7J0	71	2364.9155
U2KX49	92	3198.7834
U2T8E4	35	3679.1804
U2T8H7	19	3514.5215
U2URX1	42	2738.2034
U2YMR8	10	2724.3508
U2ZB65	92	2462.5454
U3AQY2	60	2541.6633
U3TK74	58	2396.002
U4EAX8	65	2818.9683
U4KIY0	25	2847.1614
U4QC20	27	2578.8408
U4R6Q3	74	2680.9729
U4ZZN7	43	2526.4216
U5DDI2	16	2486.1321
U5KJR1	96	2776.7688
U5VW16	75	2445.5095
U5W6M1	45	4166.1128
U5WB57	47	3998.7261
U6C7K7	18	2375.8601
U6EME9	57	2584.5012
U6EMH2	72	2715.8584
U6EQP7	21	3030.675
U6ET99	33	1046.194
U6SRQ3	62	2201.1626
U7DA26	37	2939.6223
U7G846	35	2449.1934
U7QKM3	52	2847.1816
V0ARF9	9	2618.4924
V3K6Z5	47	2560.8757
V4IGK2	20	2277.5771
V4IND8	58	2490.9805
V4KKK5	65	3897.9119
V4LAH2	63	3062.9746
V4MY52	4	2391.7358
V4P7X7	68	2717.615
V4PF31	78	2522.4177
V4PU71	34	2617.7742
V4PUN6	94	2652.2141
V4UC97	72	2958.1487
V5R1L0	2	2632.3813
V5W6I4	25	2452.3477
V5WKT4	51	3018.8145
V5X0Q1	20	2219.0886
V5X1T6	77	2086.0303
V6F0S1	23	2590.5767
V6F7V4	92	2550.9282

V6FCW6	94	2752.9307
V6FP83	43	2792.0283
V6JX71	76	2757.9636
V6JZT7	25	4281.8354
V6KM14	17	3517.0994
V6KM89	20	2180.7546
V6KP04	41	4235.8169
V6L2T1	32	2167.3457
V6T323	97	2081.4617
V6UJ24	75	2195.084
V6UK54	21	3651.804
V6UKL6	66	2834.4343
V7BWU2	76	2628.7014
V7EGD6	68	2461.1987
V9HHQ1	90	2448.8047
V9VGK4	34	2643.717
V9WHC2	59	2389.7495
V9XM14	67	2186.8799
V9XQ07	22	2023.0677
V9XQW2	76	2252.5776
V9XR34	5	2441.2915
V9XST0	67	1809.8289
V9ZZ66	56	2621.5542
W0A629	39	2853.731
W0BUD8	94	2858.6438
W0BWR5	3	3443.9006
W0BZB0	67	2836.571
W0C065	65	2965.4644
W0FLD1	45	2877.5596
W0FQM2	44	3020.1147
W0HUE2	76	2298.8767
W0IHU8	3	2388.3203
W0IYZ5	83	2634.0952
W0J0Z0	86	2322.4399
W0J296	72	2946.3542
W0J651	78	2419.4399
W0LHR5	57	2604.0322
W0LJ28	97	2630.7185
W0V5Z2	16	2670.6763
W0Z7Z9	47	3595.6104
W0ZB74	28	2677.0452
W1B1K5	33	2591.6797
W1B355	79	2546.3777
W1BBP5	59	2870.7136
W1DLQ7	34	3133.5034
W1DNG5	38	983.4854
W1DQF6	58	2184.865
W1DVS7	82	814.212
W1DXI4	62	2792.1526
W1E580	40	2146.9729

W1E5P8	39	2486.4546
W1E6M2	9	2618.4924
W1EPA2	33	2591.6797
W1EPJ8	40	2146.9729
W1EU18	96	2352.6265
W1G240	25	2514.8809
W1GLR7	57	2612.6833
W1GZC2	33	2591.6797
W1H6G4	70	2893.6157
W1HE52	90	1427.874
W1HGY6	35	2932.1414
W1HHQ1	57	2528.7561
W1HPW0	33	2591.6797
W1I0E0	13	2628.7036
W1I155	19	2881.885
W1KV84	54	2396.6965
W1KXX8	11	2384.1311
W1RQB9	42	2893.2312
W1RVK6	64	2458.2224
W1SK95	49	2191.376
W2EFT1	56	5351.6143
W2EJ94	61	2570.3057
W2EKL3	97	7926.4609
W2ESQ6	95	2429.1516
W2F073	81	2154.8506
W2F0A8	80	2272.9797
W2F271	89	2597.2522
W2U3C5	77	2092.6191
W2VAQ7	48	3630.7957
W3Y128	72	2971.405
W4BBB3	69	2192.8445
W4N6E1	27	3647.2578
W4Q343	17	2168.7913
W4QKR4	12	2142.5632
W4QZ14	91	2212.8936
W4R1E9	40	2206.6597
W4V2R1	93	2547.2976
W5VYM2	65	2753.4668
W5W1A4	48	2296.8999
W5WEG0	66	3816.4414
W5WHJ9	93	3355.6553
W6CG73	15	2241.0935
W6K116	20	2454.7688
W6LTU9	96	2572.6499
W6M302	66	2493.9058
W6M8D9	23	2900.79
W6RG33	26	2748.8416
W6TLA4	83	2328.1348
W6W1F3	22	2967.1121
W6WWY6	14	2475.2275

W7IPT8	15	2763.1221
W7IVY8	16	3796.7317
W7NGV8	18	2813.2236
W7SV29	94	2465.8987
W7TCZ5	43	4398.9951
W7TGA6	10	3357.8269
W7VBV3	89	4007.6421
W7VGI0	25	4924.6147
W7VLW8	86	2543.3369
W7VM47	69	3755.9456
W7VRE1	43	3337.9355
W7W731	60	2412.1509
W8EZI0	24	2331.7532
W8I1S6	35	2642.2998
W8SAR2	19	919.3439
W8UFH6	35	2932.1414
W8UJL1	19	2661.9041
W8UVM0	33	2591.6797
W8XWK7	81	2587.1548
W8YRW3	85	2080.7742
W9ADI0	77	2002.7377
W9BGS1	29	2536.3438
W9EAC3	10	2074.3691
W9EEC0	78	2062.9062
W9FP75	81	2370.2588
W9FS44	21	3651.804
W9FVS7	61	4811.459
W9FXL9	26	2734.0659
W9G2W6	34	2262.1497
W9GB96	73	2338.3499
W9GDW6	14	2702.4287
W9GTD4	99	4293.6724
W9VIM8	27	2710.3071
X0N079	7	2656.791
X0R5P1	2	2236.7544
X2H6V1	30	2743.6311
X4ZXV2	56	2176.5847
X5K473	41	2657.6372
X5NUA2	56	2673.4124
X6L011	30	2366.4707
X7EHA7	95	2642.7739
X7F492	73	2585.1729
X8HBB2	85	3080.4121
X8ITU6	5	3004.0801
Z5DUF2	15	2624.3865
Z5XSU4	13	2595.116
Z5XXT5	37	2594.0867
